# Transcriptomic and ChIP-sequence interrogation of EGFR signaling in HER2+ breast cancer cells reveals a dynamic chromatin landscape and S100 genes as targets

**DOI:** 10.1186/s12920-019-0477-8

**Published:** 2019-02-08

**Authors:** Miguel Nava, Pranabananda Dutta, Nathan R. Zemke, Robin Farias-Eisner, Jaydutt V. Vadgama, Yanyuan Wu

**Affiliations:** 1 Division of Cancer Research and Training, Department of Medicine, Charles R. Drew University of Medicine and Science, 1731 East 120th Street, Los Angeles, CA 90059 USA; 2 Jonsson Comprehensive Cancer Center and David Geffen School of Medicine, University of California, Los Angeles, CA USA; 3 Molecular Biology Institute, University of California, Los Angeles, USA

**Keywords:** HER2, Breast Cancer, EGFR, Epigenetics, Chromatin, Next generation sequencing

## Abstract

**Background:**

The Human Epidermal Growth Factor Receptor (EGFR/HER1) can be activated by several ligands including Transforming Growth Factor alpha (TGF-α) and Epidermal Growth Factor (EGF). Following ligand binding, EGFR heterodimerizes with other HER family members, such as HER2 (human epidermal growth factor receptor-2). Previously, we showed that the EGFR is upregulated in trastuzumab resistant HER2 positive (HER2+) breast cancer cells. This study is aimed to determine the downstream effects on transcription following EGFR upregulation in HER2+ breast cancer cells.

**Methods:**

RNA-sequence and ChIP-sequence for H3K18ac and H3K27ac (Histone H3 lysine K18 and K27 acetylation) were conducted following an Epidermal Growth Factor (EGF) treatment time course in HER2+ breast cancer cells, SKBR3. The levels of several proteins of interest were confirmed by western blot analysis. The cellular localization of proteins of interest was examined using biochemically fractionated lysates followed by western blot analysis.

**Results:**

Over the course of 24 h, EGFR stimulation resulted in the modulation of over 4000 transcripts. Moreover, our data demonstrates that EGFR/HER2 signaling regulates the epigenome, with global H3K18ac and H3K27ac oscillating as a function of time following EGF treatment. RNA-sequence data demonstrates the activation of immediate early genes (IEGs) and delayed early genes (DEGs) within 1 h of EGF treatment. More importantly, we have identified members of the S100 (S100 Calcium Binding Protein) gene family as likely direct targets of EGFR signaling as H3K18ac, H3K27ac and pol2 (RNA polymerase II) increase near the transcription start sites of some of these genes.

**Conclusions:**

Our data suggests that S100 proteins, which act as Ca2+ sensors, could play a role in EGF induced tumor cell growth and metastasis, contribute to trastuzumab resistance and cell migration and that they are likely drug targets in HER2+ breast cancer.

**Electronic supplementary material:**

The online version of this article (10.1186/s12920-019-0477-8) contains supplementary material, which is available to authorized users.

## Background

Human Epidermal Growth Factor Receptor-2-positive (HER2+) breast cancer is one of the four major molecular sub-types of breast cancer. HER2 is a classical receptor tyrosine kinase (RTK) and its kinase activity is stimulated by heterodimerization with other ligand bound HER family members, such as EGFR/HER1 [1–3]. Treatment for HER2+ breast cancer includes the use of trastuzumab, a monoclonal antibody that binds to the HER2 extracellular domain and inhibits downstream signaling [4]. Primary or acquired resistance to trastuzumab has been a major challenge for clinical management of this disease. Resistance to trastuzumab may involve intrinsic alternations in HER2 receptor (e.g. deletions of the regions coding the trastuzumab binding site); loss of antibody-dependent cell-mediated cytotoxicity (ADCC); intracellular alterations in HER2 downstream signaling; and crosstalk between receptors and signaling pathways leading to activation of other HER family receptors, such as EGFR [5].

EGFR binds to its cognate ligand EGF (Epidermal Growth Factor) which induces receptor tyrosine phosphorylation and promotes cross talk between EGFR family members and other signaling pathways. Aberrant EGFR activity has been shown to play a key role in therapeutic resistance [5, 6]. A previous study from our laboratory demonstrated that HER2+ breast cancer cells resistant to trastuzumab had higher levels of EGFR relative to controls, which we hypothesized could amplify downstream signaling and enhance migration [7]. Although the EGFR signaling cascade has been studied extensively, several aspects still remain elusive.

The effects on transcription following activation of EGFR have been studied in several cancer and non-cancer cell types and with the use of different methodologies [8–14]. With the use of microarray technology it was demonstrated that HeLa cells (cervical cancer cells) stimulated with EGF exhibited waves of transcription within a time course of 8 h [8]. Stimulation of non-cancer breast cells, MCF10A with EGF activated intracellular signal transduction pathways that lead to the induction of genes involved in cell motility, in addition to the activation of immediate early genes (IEGs) [8, 9]. The same group also showed that EGF-regulated mRNA’s in MCF10A cells were modulated post-transcriptionally by immediately down-regulated microRNAs (ID-miRs), immediately up-regulated microRNAs (IU-miRs) and delayed up-regulated microRNAs (DU-miRs) [9, 13].

More recent publications have investigated EGFR-modulated transcription utilizing Next Generation Sequencing (NGS) technologies [12, 14]. Interestingly, immediate early genes (IEGs) are relieved of the “pause” step in transcription by the recruitment of the Integrator complex following EGF treatment of HeLa cells [12]. The Integrator complex is also involved in EGF stimulation dependent biogenesis of enhancer RNAs (eRNAs) [14]. Due to these results and more recent ones, the Integrator complex has emerged as a novel putative drug target in melanoma cell line, A375 and non-small cell lung adenocarcinoma cell line, A549 [15]. However, despite these findings, the effects on transcription following long term EGF stimulation of EGFR (> 20 min) and its underlying chromatin basis remains unexplored in HER2+ breast cancer cells.

In this study, we used Next Generation Sequencing (NGS) technologies to determine the fluctuations in gene expression following EGF treatment of SKBR3 (HER2+ breast cancer cells). We found that over 4000 transcripts are modulated by 2-fold or more during a 24 h EGF time course. In addition, we observed oscillations in H3K18ac and H3K27ac during the same period. Surprisingly, regardless of when transcript levels peak, all activated genes gain H3K18ac and H3K27ac within 1 h post EGF treatment, have a decrease in those marks 6 h post-EGF treatment and regain those 24 h post-EGF treatment. Lastly, we identified S100 genes, which have been reported to contribute to tumorigenic processes [16], as likely direct targets of EGFR/HER2 signaling. S100 genes gradually increase in expression during an EGF treatment time course, gain H3K18ac and H3K27ac marks near their promoters and some also gain pol2 within 20 min of EGF treatment in HeLa cells.

## Methods

### Cell lines

The human breast cancer cell line SKBR3 (HTB-30) was obtained from American Type Culture Collection. Monolayer cultures of the cells were maintained in DMEM/F12 medium with 10% fetal bovine serum. The cell line overexpressed the HER2/c-erb-2 (HER2) gene product. The SKBR3/100–8 line was generated from SKBR3 by clonal selection after long term adaptation in maintained in growth medium containing 100 μg/ml of trastuzumab in our Laboratory. The clone was confirmed to be through clone selection and confirmed remaining HER2 overexpression and insensitive to trastuzumab treatment [7].

### Western blot

Protein lysates were prepared using EBC modified buffer (50 mM Tris-Cl (pH 8.0), 150 mM NaCl, 0.5% NP-40) containing Thermo Scientific Protease and Phosphatase Inhibitor Tablets (A32959). The following antibodies were used: phospho-EGFR (Tyr1068) (Cell Signaling Technology, Cat. 3777S), EGFR (Cell Signaling Technology, Cat. 4267S), phospho-AKT (Cell Signaling Technology, Cat. 9271S), AKT (Cell Signaling Technology, Cat. 9272S), phospho-ERK1/2 (Thr202/Tyr204) (Cell Signaling Technology, Cat. 4370S), ERK1/2 (Cell Signaling Technology, Cat. 4695S) and β-actin (Santa Cruz Biotechnology, sc-69,875).

### Quantitative-PCR

RNA was isolated using Trizol (ThermoFisher Scientific, Cat. 15,596,026). 1μg of RNA was used to construct libraries using iScript (Bio-Rad, Cat. 1,708,840). 1/20th of reaction mix from constructed libraries was used in each qPCR reaction using iTAC Universal SYBR Green Supermix (Bio-Rad, Cat. 1,725,122). Primers used for qRT-PCR can be found in Additional file 1: Table S3.

### RNA-seq

The media from subconfluent SKBR3 cells was changed from 10% FBS DMEM to 0.2% FBS DMEM 18–20 h prior to the beginning of the EGF treatment time course. EGF was added to a final concentration of 50 ng/mL for indicated time intervals. RNA was isolated using Trizol (ThermoFisher Scientific, catalog number 15596026). Nanodrop and Qubit Fluorometric instruments were used to determine RNA concentrations. 1μg of RNA was used to construct libraries with KAPA mRNA Hyperprep Kit (Roche KK8580). Libraries were sequenced on an Illumina Hiseq 3000 instrument.

### RNA-seq analysis

Biological replicates (*n* = 2) mRNA-seq (single end 50 bp) reads were aligned to hg19 using default parameters of Tophat2 (version 2.1.0) and Bowtie2 (version 2.3.2). Samtools (version 0.1.18) was used to convert SAM to BAM files. FPKM values were generated using default parameters for Cuffdiff (version 2.1.1). Only FPKM values greater than 0.5 were considered for further analysis. *P*-values for RNA-seq are reported as the output files indicate (which are based on the Jensen-Shannon metric).

### ChIP-PCR

The same protocol for ChIP-seq was followed up to the purification and quantitation of ChIP DNA (see below). At that point, equal mass of ChIP and input DNA was used in a qPCR reaction. Enrichment was determined by dCt relative to input. Primers used in qPCR can be found in Additional file 1: Table S3.

### ChIP-seq

The media from subconfluent SKBR3 cells was changed from 10% FBS DMEM to 0.2% FBS DMEM 18–20 h prior to the beginning of the EGF treatment time course. EGF was added to a final concentration of 50 ng/mL for indicated times. Following EGF treatment time course, formaldehyde was added to a final concentration of 1% and incubated for 10 min at 37 °C. Following PBS washing, cells were scraped and washed with 1 mL of PBS containing protease inhibitors (Roche). Cells were resuspended in lysis buffer at a ratio of 5 × 10^6^ cells per 100ul of lysis buffer. 150ul of cell lysate was used in chromatin immunoprecipitation with a given antibody. 10ul of cell lysate was saved for use as input. 3μg of antibody (H3K18ac (EMD Millipore, catalog 07–354) and H3K27ac (Active Motif, catalog 39,135)) was used per ChIP. Following washes and elution, immunoprecipitated material was reverse crosslinked overnight at 65 °C. Samples were treated with RNase A for 30 min at 37 °C and then with Proteinase K for 2 h at 56 °C. DNA was recovered using phenol/chloroform extraction and precipitation. Qubit Fluorometric instrument was used to quantify concentration of recovered DNA. 1 ng of DNA was used to construct libraries with KAPA Hyper Prep Kit (Roche KK8502). Libraries were sequenced on an Illumina Hiseq 3000 instrument.

### ChIP-seq analysis

Biological replicates (*n* = 2) single end 50 bp reads were aligned to hg19 using default parameters of Bowtie2 (version 2.3.2). Only reads that aligned to a unique position in the genome with no more than two sequence mismatches were retained for further analysis. Peaks were identified with MACS2 (version 2.1.1) using default parameters (q-value (minimum FDR) was 0.01 and *p*-value < .0005). Q-values are calculated from *p*-values using Benjamini-Hochberg method. MACS2 output Bedgraph files were converted to BigWig using bedGraphtoBigWig. And BigWig files were converted to Wiggle files for use in CEAS with bigWigtoWig (http://hgdownload.soe.ucsc.edu). Integrated Genome Browser (IGB) was used to view Bedgraph, BigWig and Wiggle files (http://bioviz.org/igb/). Average H3K18ac and H3K27ac enrichment near the TSS was determined using Cis-regulatory Element Annotation System (CEAS) (http://liulab.dfci.harvard.edu/CEAS/). Bedtools (version 2.26.0) intersect option was used to determine overlapping peaks between H3K18ac or H3K27ac peaks at different time points.

### Transcription factor binding site (TFBS) query

Homer (version 4.9) (homer.ucsd.edu) was used to find enriched motifs from − 300 to + 50 bp (default settings) of each cluster in the RNA-seq data set. Only motifs that were enriched with a *p*-value < .01 are reported.

## Results

### EGFR signaling and expression of immediate early genes (IEGs) in SKBR3 cells

**Fig. 1:**
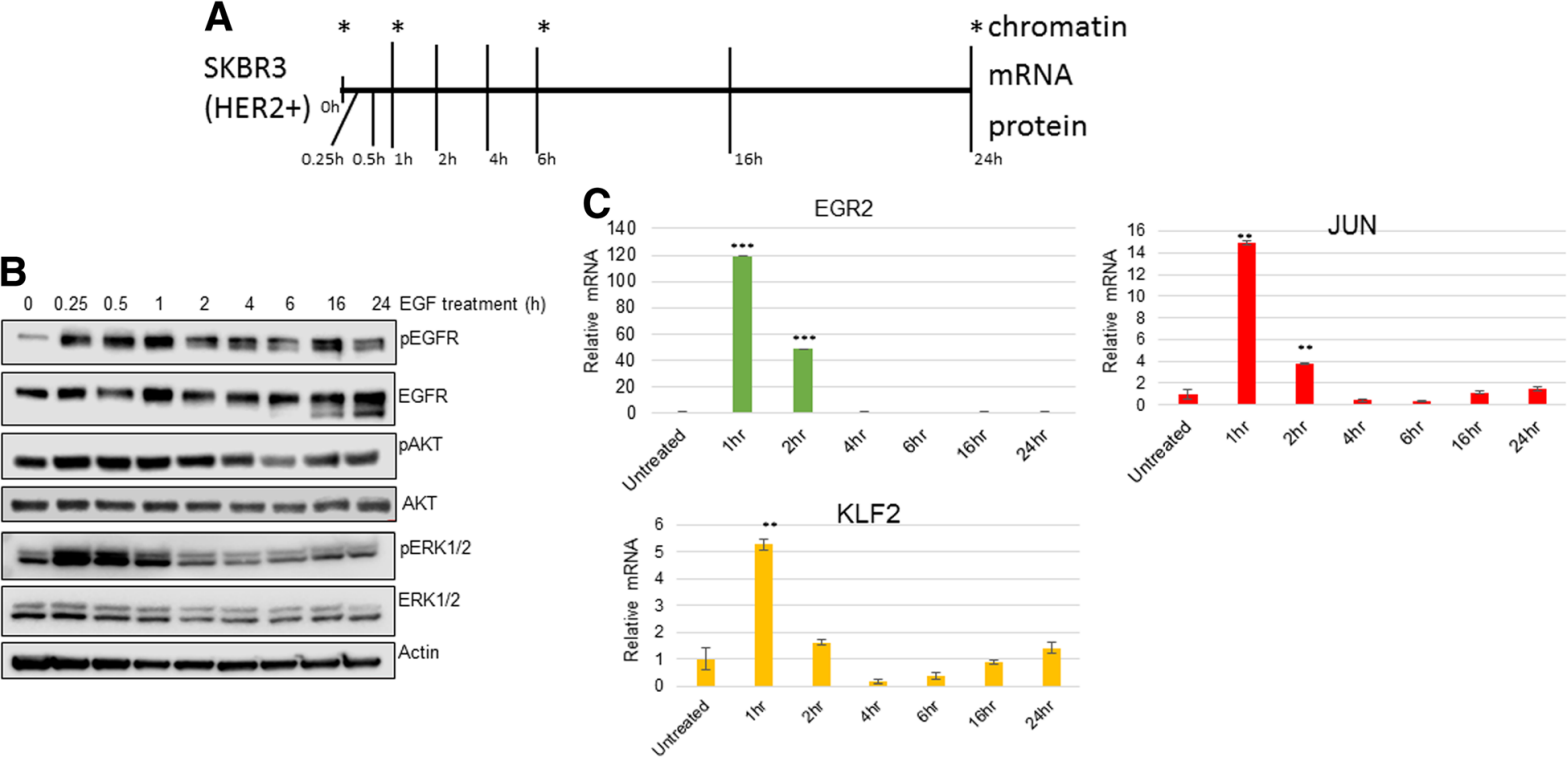
Schematic of experimental outline and EGF time course. **a** EGF was added to HER2+ SKBR3 cells for indicated times. Total RNA, protein or chromatin (*) was harvested for the indicated times. **b** Abundance of proteins involved in EGFR signaling were immunoblotted (*n* = 2 repeats). **c** Levels of known immediate early genes (IEGs) were assayed by qPCR during an EGF time course (*n* = 3 repeats). *p-value < 0.1, **p-value < .01, ***p-value < .001 one-sided t-test for significant difference between Untreated and EGF treated samples

In order to determine the cellular dynamics following EGF treatment (50 ng/mL) in serum starved SKBR3 cells, we extracted RNA, protein and chromatin at similar time points (Fig. 1a). EGFR signaling is initiated by ligand activated receptor heterodimerization with HER2, HER3 (human epidermal growth factor receptor-3) or HER4 (human epidermal growth factor receptor-4) and subsequent MAPK (mitogen-activated protein kinase) phosphorylation. Therefore to analyze these events, we subjected protein lysates to immunoblotting. EGFR phosphorylation increased within 15 min of EGF treatment and this was consistent with a concomitant increase in pAKT (activated AKT/protein kinase B) and pERK1/2 (activated Extracellular signal-regulated kinase 1/2 (ERK1/2)) (Fig. 1b). EGFR phosphorylation remained higher than baseline levels throughout the 24 h EGF time course. However, pAKT and pERK1/2 levels returned to basal levels by 2 h and 1 h post-EGF treatment, respectively. The transient activation of pAKT and pERK1/2 following EGFR stimulation is consistent to previously published data for similar experiments conducted in normal mammary epithelial cells, MCF10A [17]. However, unlike similar experiments conducted in MCF10A cells, SKBR3 cells treated with EGF have a longer duration of higher than basal levels of pERK1/2 and maintain nearly equal levels of EGFR throughout the duration of the time course.

We next determined the regulation of IEGs in SKBR3 cells following EGF treatment. As has been observed in HeLa and MCF10A cells [8], the treatment of SKBR3 cells with EGF resulted in a robust induction of *EGR2 (Early growth response protein 2)*, *JUN (Jun Proto-Oncogene)* and *KLF2* (*Krüppel-like Factor 2*) (Fig. 1c) As the name for this group of genes suggests, they peaked in expression immediately after EGF treatment (1 h post) and rapidly decreased at later time points, falling to below basal levels by 4 h post-EGF treatment. Interestingly, *EGR2*, *JUN* and *KLF2* levels stabilized back to basal levels at later time point’s post-EGF treatment (16 h–24 h).

### mRNA-sequencing (RNA-seq) of SKBR3 cells treated with EGF

**Fig. 2:**
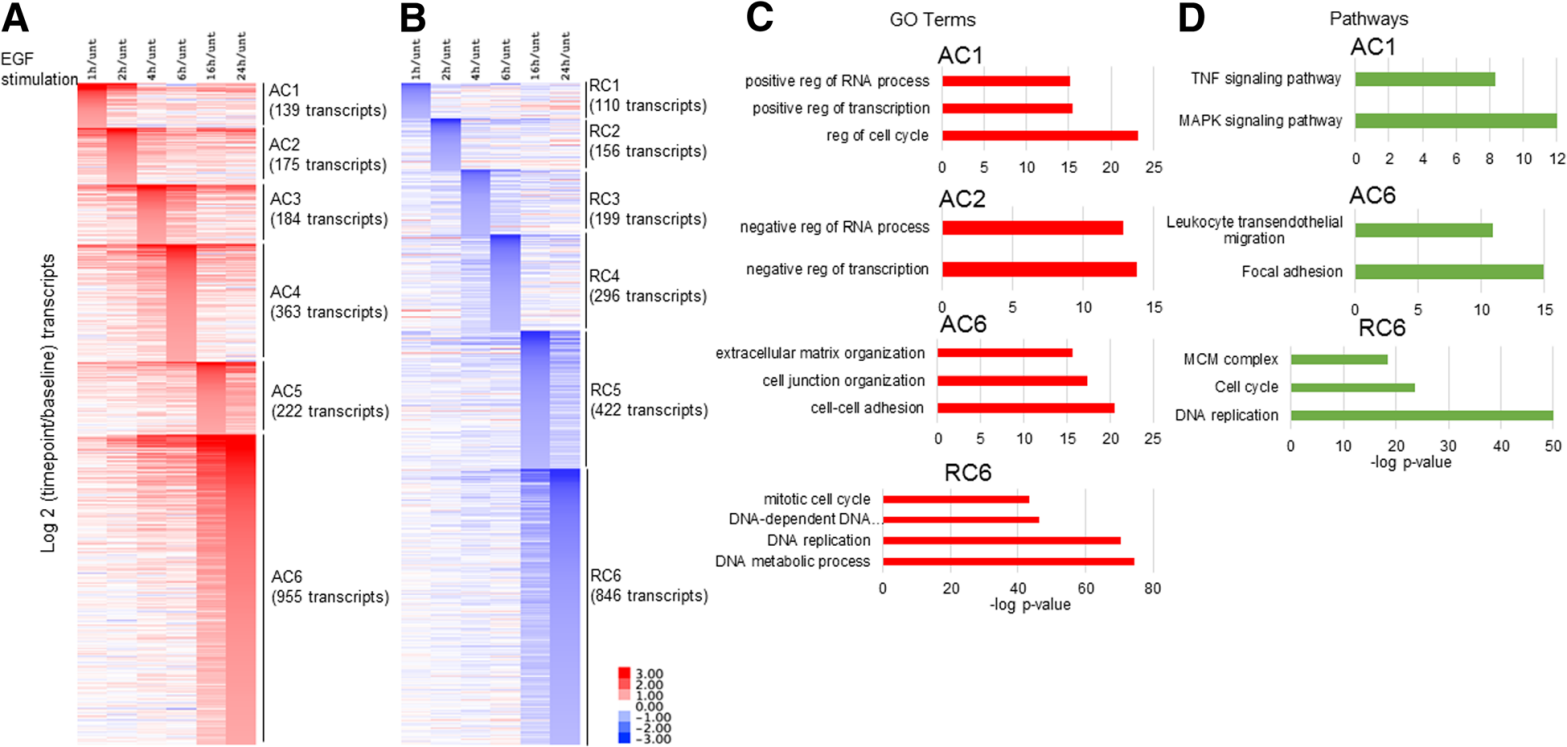
EGF RNA-seq time course and GO terms. **a** EGF was added for indicated times in serum starved SKBR3 cells. RNA-seq was conducted. Fragments Per Kilobase of transcript per Million mapped reads (FPKM) values were generated using Cufflinks. And transcripts whose expression was induced or **b** repressed to at least twice (in biological replicates) the baseline level were graphed according to when they reached their peak or summit in expression AC = activated cluster RC = repressed cluster. Only FPKM values of greater than 0.5 were kept. The number of each transcript in each cluster is indicated in parenthesis. **c** Gene ontology (GO) terms and **d** KEGG Pathways as reported by HOMER

Previous EGF time course experiments to determine global changes in transcription utilized tiling arrays and/or limited their investigation to earlier time points (< 8 h) [8, 10–12]. Therefore to expand our current knowledge, we explored gene expression in SKBR3 cells following EGF treatment utilizing mRNA-sequencing (RNA-seq). Serum starved SKBR3 cells were treated with EGF (50 ng/mL) and RNA was isolated at time 0, 1 h, 2 h, 4 h, 6 h, 16 h and 24 h post-EGF treatment (Fig. 1a). Following alignment and transcript quantification, transcripts with Fragments Per Kilobase of transcript per Million mapped reads (FPKM) > 0.5 and those that were differentially expressed by 2-fold or more compared to untreated SKBR3 cells in biological replicates were plotted in a heatmap according to their peak expression or repression time (Fig. 2a and b). In total, 2038 transcripts increased in expression by 2-fold or more compared to untreated SKBR3 cells during the 24 h EGF time course (Fig. 2a and Additional file 2: Table S1). We subdivided these transcripts into six clusters of activated clusters (AC) 1–6, based on their peak expression time (Fig. 2a). On the other hand, 2029 transcripts reduced in expression by 2-fold or more compared to untreated SKBR3 cells during the 24 h EGF time course (Fig. 2b and Additional file 3: Table S2). These transcripts were also subdivided into six clusters of repressed clusters (RC) 1–6, based on their peak repression time (Fig. 2b). All clusters of genes were statistically significant (*p*-value < 0.001) when compared to transcript expression in untreated serum starved SKBR3 cells (Additional file 4: Figure S1).

### Early activated transcripts (< 2 h)

Activated cluster 1 (AC1) contains 139 transcripts that peaked in expression 1 h post-EGF treatment (Fig. 2a). Gene ontology (GO) and Kyoto Encyclopedia of Genes and Genomes (KEGG) pathway analysis of AC1 transcripts confirmed that EGFR stimulation activated transcripts involved in cell cycle function, gene regulation and MAPK and TNF signaling pathways (Fig. 2c and d). AC1 contains transcripts that have been previously classified as IEGs (*FOS (Fos Proto-Oncogene), JUN, NR4A1 (Nuclear Receptor Subfamily 4 Group A Member 1)* and *EGR1 (Early growth response protein 1)*) and DEGs (*KLF2 (Kruppel Like Factor 2), Dual Specificity Phosphatase 1, 4, 5, and 8 (DUSP1, DUSP4, DUSP5 and DUSP8*)) (Additional file 2: Table S1) [8, 12]. *ZFP36*, also known as TTP (zinc finger protein 36 homolog) was also in AC1. ZFP36 has previously been shown to regulate IEGs post-transcriptionally by promoting the degradation of IEGs like *FOS* [8]. Therefore, it is likely that in HER2+ SKBR3 cells, ZFP36 is also an attenuator of EGFR signaling at the post-transcriptional level. In short, a 1 h EGF treatment of SKBR3 cells activated genes that are known to promote and antagonize MAPK signaling.

AC2 contains 175 transcripts, whose activation peaked 2 h post EGF treatment, and these genes are known as transcriptional repressors (Fig. 2a, c and Additional file 2: Table S1). Examples of these transcripts are *MAFF (MAF BZIP Transcription Factor F), ID1 (DNA-Binding Protein Inhibitor ID-1), Kruppel-Like Factor 10, 4, 7 (KLF10, KLF4, KLF7),* and *EZH2 (Enhancer Of Zeste 2 Polycomb Repressive Complex 2 Subunit)*. MAF (MAF BZIP Transcription Factor) proteins are basic leucine zipper (bZIP) family of transcription factors that have activator and repressor functions in various tissues [18]. The function of MAFF in EGFR signaling remains largely unexplored, although ectopic expression of MAFF inhibited EGF driven reporter activity [8]. Kruppel-like factor (KLF) family of transcription factors are known downstream targets of EGFR signaling and, like MAFF, function as both activators and repressors through their interactions with histone modifying complexes such as p300/CBP (E1A binding protein p300/ CREB-binding protein), CtBP (C-terminal-binding protein) and Sin3A (Histone Deacetylase Complex Subunit Sin3a) [8, 19].

### Late activated transcripts (24 h)

AC6 contains 955 transcripts and these transcripts play a role in extracellular matrix organization, cell junction organization and cell-cell adhesion (Fig. 2a, c and Additional file 2: Table S1). The transcripts in AC6 are indicative of cellular migration and focal adhesion pathways activation (Fig. 2d). Some of the genes in AC6 are known EGFR targets, such as *MMP9 (Matrix Metallopeptidase 9), ITGA5 (Integrin Subunit Alpha 5), KRT17(Keratin 17)* [8, 10, 20]. However, some have never been described as downstream EGFR targets, such as Claudin (CLDN) family members *CLDN9* and *CLDN12*. Claudin proteins are known to localize to tight junctions, however some Claudin members are known to promote migration and metastasis [21]. *FHL2* (Four-and-a-half LIM domains protein 2) was one of the most differentially expressed genes at 24 h post-EGF treatment, with an initial increase in expression 2 h post-EGF treatment (Additional file 2: Table S1). FHL2 is known to be a modulator of transcription that also has additional roles in promoting signal transduction and cell migration [22]. Wingless-Type MMTV Integration Site Family, Member 9A (*WNT9A)* followed the same trend as *FHL2*, initially increasing in expression at 2 h post-EGF treatment (Additional file 2: Table S1). WNT9A, a non-canonical WNT, is known to play role in morphogenesis, development and proliferation inhibition, but its function in HER2+ breast cancer is unknown [23–25]. Lastly, 10 S100 family of genes (12 transcripts) peaked in expression 24 h post-EGF treatment, but some had initial differential gene expression as early as 4 h post-EGF treatment (Fig. 5). Regulation of *S100* genes will be discussed below.

### Late repressed transcripts (24 h)

RC6 contains 846 transcripts and these transcripts play a role in cell cycle and DNA replication (Fig. 2b,c and Additional file 3: Table S2). The genes in RC6 are functional components in MCM (minichromosome maintenance) complex formation and DNA replication initiation (Fig. 2d). *MCM2, MCM3, MCM4, MCM5, MCM6* and *MCM7* are all repressed 24 h post-EGF treatment. In addition to MCM transcripts, *PCNA (Proliferating cell nuclear antigen), CDC25A (M-phase inducer phosphatase 1), CDC45 (Cell Division Cycle 45), E2F Transcription factor 1 and 2 (E2F1, E2F2),* and *CDT1* (DNA replication factor) are also in RC6. EGFR signaling has been known to decrease ^3^H-Thymidine incorporation in EGF treated breast cancer cells, including SKBR3 cells [26]. This is probably due to the potent activation of *CDKN1A* (p21), an inhibitor of G1 Cyclin Dependent Kinases (CDKs) [27, 28]. *CDKN1A* peaked in expression 4 h post-EGF (i.e. AC3) and remained higher than baseline levels throughout the EGF time course. Therefore, we have identified the cell cycle genes that are repressed as a result of EGF treatment.Table 1 summarized those genes regulated by EGF. Additional files 2 and 3: Table S1 and Table S2 lists all genes modulated by EGF treatment.

**Table 1:** Summary of genes regulated by EGF

AC1	AC2	AC3	AC6	RC2	RC5	RC6
FOS	MAFF	CDKN1A	MMP9	CPNE1	MAX	MCM2
JUN	ID1		ITGA5			MCM3
NR4A1	KLF4		KRT17			MCM4
EGR1	KLF7		CLDN9			MCM5
DUSP1	KLF10		CLDN12			MCM6
DUSP4	EZH2		FHL2			MCM7
DUSP5	KDM3A		WNT9A			PCNA
DUSP8	KDM6B		S100A5			CDC25A
ZFP36			S100A2			CDC45A
			S100A3			E2F1
			S100A10			E2F2
			S100A6			CDT1
			S100A13			
			S100A4			
			S100A7			
			S100P			
			S100A16			

### Chromatin immunoprecipitation (ChIP) H3K18ac and H3K27ac

In order to explore the underlying chromatin dynamics following EGF treatment of SKBR3 cells, we conducted ChIP-qPCR and assayed H3K18ac and H3K27ac enrichment near the transcription start site (TSS) of three regulated genes. We decided to specifically assay H3K18ac and H3K27ac enrichment because (1) they are marks that are exclusively deposited by co-activator paralogs P300/CBP [29], (2) these marks are enriched in transcriptionally active sites [14, 30], (3) these marks increase following activation [12, 31], (4) H3K27ac has previously been shown to increase near EGF activated genes [12] and (5) H3K27ac is a marker of super-enhancers [32].

**Fig. 3:**
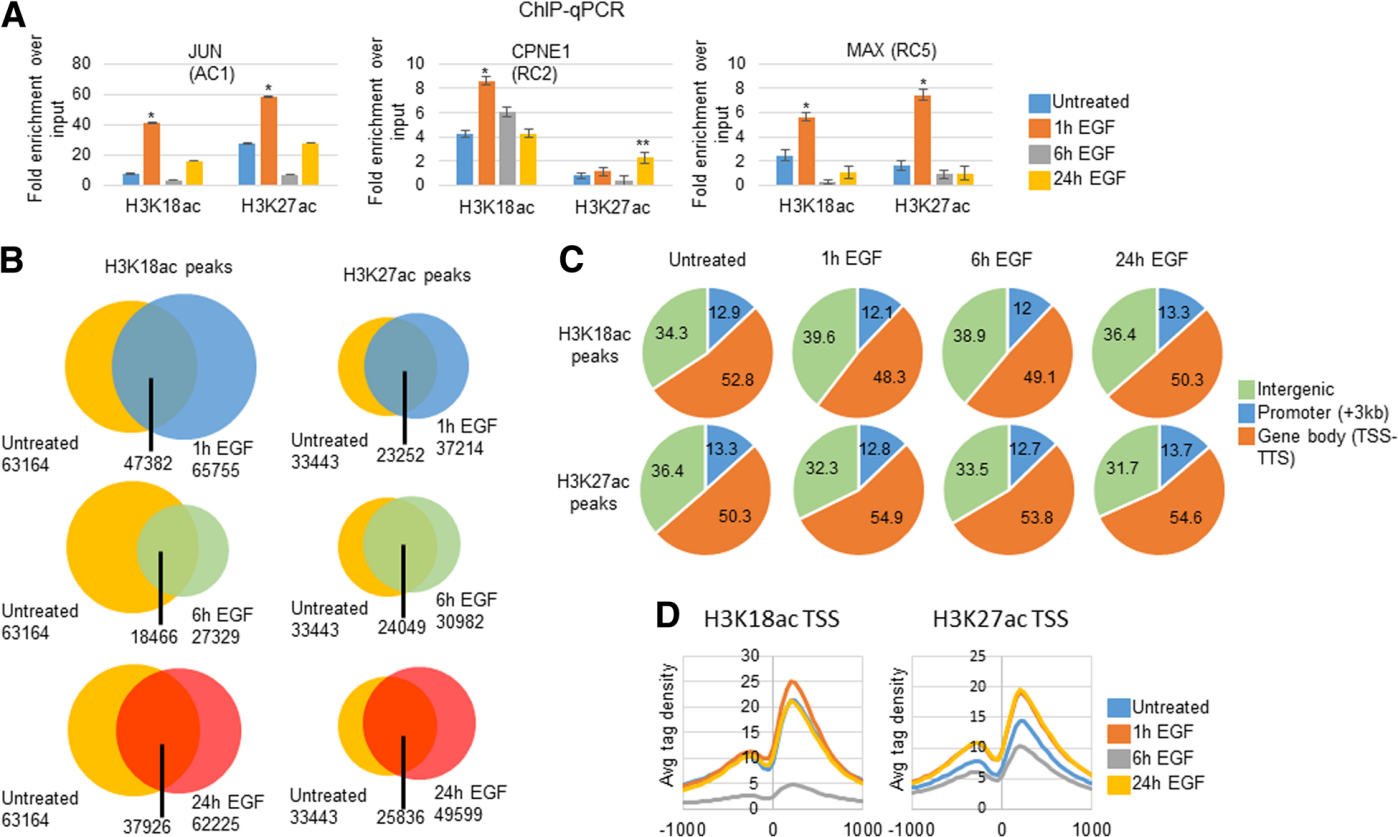
H3K18ac and H3K27ac were mapped post EGF treatment**. a** EGF was added for indicated times in serum starved SKBR3 cells. Chromatin was subjected to ChIP as indicated in the protocols (−/+SD). Enrichment was determined by using primers near the TSS of indicated genes. *p-value < 0.1 and **p-value <.01 t-test for significant difference between Untreated and EGF treated samples. **b** Total ChIP-seq peaks were counted for each condition and overlapping peaks were identified for each timepoint within each ChIP. **c** The location of each peak was determined using CEAS (sitepro) option. **d** H3K18ac and H3K27ac signals were mapped at all TSS by timepoint

We first assayed H3K18ac and H3K27ac at a classic IEG, *JUN* (Fig. 3a). H3K18ac increased 1 h post-EGF treatment when compared to untreated cells. By 6 h post-EGF treatment, H3K18ac fell below H3K18ac levels in untreated cells. H3K18ac levels rebounded above basal levels 24 h post EGF treatment. The oscillation of H3K18ac following EGF treatment was recapitulated by H3K27ac levels near the JUN TSS (Fig. 3a). H3K27ac levels also increased 1 h post-EGF treatment compared to untreated cells, decreased below basal levels at 6 h post-EGF treatment and returned to near basal levels at 24 h post-EGF treatment.

We then assayed H3K18ac and H3K27ac near the TSS of *CPNE1 (Copine-1)*, a repressed gene in RC2 (Fig. 3a). Surprisingly, H3K18ac increased near the TSS 1 h post-EGF treatment and fell to near basal levels by 24 h post-EGF treatment. The CPNE1 TSS had minimal H3K27ac enrichment through the 24 h EGF time course.

Having observed H3K18ac increase near a repressed gene, we assayed H3K18ac and H3K27ac near another repressed gene, *MAX (myc-associated factor X)*, that peaked in repression 16 h post EGF treatment (RC5) (Fig. 3a). Interestingly, both H3K18ac and H3K27ac increased 1 h post-EGF treatment near the TSS of MAX. H3K18ac and H3K27ac enrichment decreased dramatically at 6 h and remained low at 24 h post-EGF treatment.

### ChIP-sequence (ChIP-seq) H3K18ac and H3K27ac

**Fig. 4:**
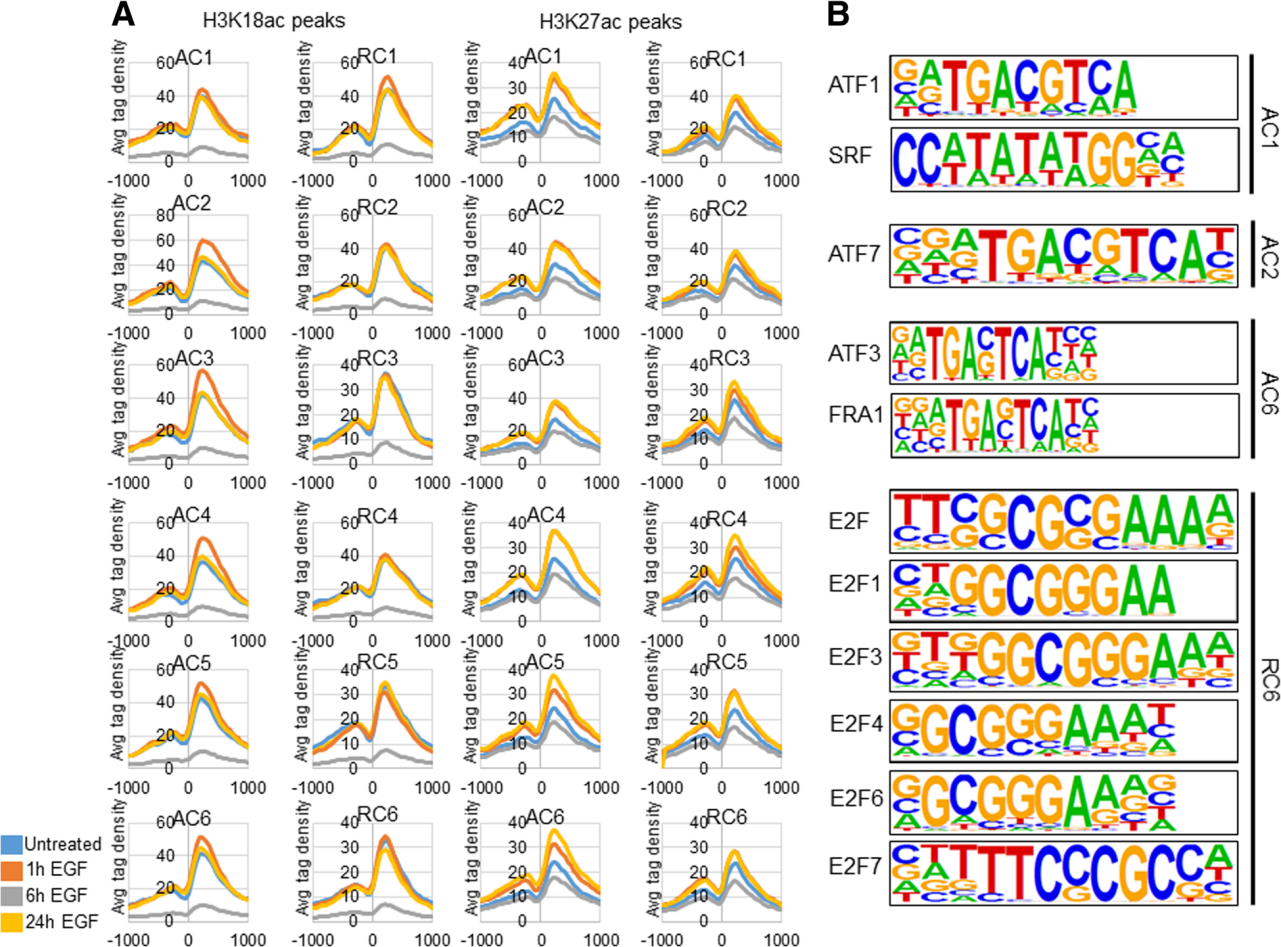
H3K18ac, H3K27ac and transcription factor binding sites (TFBS) near TSS of clusters. **a** Genes in each cluster from Fig. 2 were subjected to CEAS analysis. **b** Motif search results as indicated by HOMER search from -300 bp to + 50 bp of each cluster. All motifs shown were enriched at *p*-value <.01

The vast majority of ChIP-seq experiments in EGF treated cells have focused on IEGs and immediately activated enhancers [12, 14]. Therefore, we decided to conduct ChIP-seq for H3K18ac and H3K27ac and to look at all genes (Fig. 3b, c, and d). There were over 60,000 H3K18ac peaks in SKBR3 cells at every time point except at 6 h post-EGF treatment (Fig. 4b and Table 2). Moreover, the majority of H3K18ac peaks were shared between untreated cells and all times post-EGF treatment (Fig. 4b and Table 1). Most H3K18ac peaks that were present following EGF treatment were not at new sites.

**Table 2:** Summary of number of peaks in each ChIP-seq

	H3K18ac peaks	H3K27ac peaks
0 h	63,164	33,443
1 h	65,755	37,214
6 h	27,239	30,982
24 h	62,225	49,599

H3K27ac enrichment has previously been analyzed in EGF treated HeLa cells [12, 14]. HeLa cells are cervical adenocarcinoma cells and the location of H3K27ac could vary significantly from cell type to cell type [32]. Therefore, we decided to analyze H3K27ac in SKBR3 cells treated with EGF (Fig. 3b and c). There were over 30,000 H3K27ac peaks at every time point assayed. Like for H3K18ac peaks, most H3K27ac that were present following EGF treatment were not at new sites. Although at 6 h there were the fewest H3K27ac peaks, there was no stark discrepancy in H3K27ac peak numbers during the time course as there was for H3K18ac.

The majority of H3K18ac and H3K27ac peaks in untreated cells were in gene bodies (defined as TSS to transcription termination site (TTS)), 52.8 and 50.3% of total peaks, respectively (Fig. 3c). In general, a greater proportion of H3K18ac peaks remained at intergenic sites and a lower proportion of H3K18ac peaks in gene bodies in EGF treated samples compared to untreated cells (Fig. 3c). In contrast to H3K18ac peaks, H3K27ac peaks decreased at intergenic regions and increased in gene bodies in all EGF treated samples compared to untreated cells (Fig. 3c). Lastly, H3K18ac and H3K27ac peaks did not change significantly at promoters (defined as < − 3 kb from TSS) during the EGF time course.

We next analyzed H3K18ac and H3K27ac levels and distribution at all TSS. Untreated cells exhibited high H3K18ac levels at ~ + 200 bp (Fig. 3d). This peak increased at 1 h post-EGF treatment, dramatically decreased at 6 h and rebounded to baseline levels at 24 h. There was also a much smaller H3K18ac peak at ~ − 250 that was similar in height for untreated cells and cells treated for 1 h and 24 h. Cells treated for 6 h had the lowest H3K18ac levels throughout the − 1000 to + 1000 window.

Similar to H3K18ac enrichment at the TSS of all genes, H3K27ac increased at 1 h from − 1000 to + 1000, but with similar peak locations to H3K18ac at ~ − 250 and ~ + 200. These H3K27ac peaks decreased at 6 h to below H3K27ac peaks in untreated cells. Similar to H3K18ac peaks, H3K27ac peaks rebounded from their 6 h levels at 24 h. Unlike H3K18ac, cells treated for 24 h had higher H3K27ac at all TSS than untreated cells. H3K27ac signal at 1 h and 24 h post-EGF treatment were similar at all TSS.

### H3K18ac and H3K27ac at regulated genes

Having observed that H3K18ac and H3K27ac increased at activated and repressed genes (Fig. 3a), we analyzed H3K18ac and H3K27ac enrichment near the TSS of all regulated genes (Fig. 4a). H3K18ac and H3K27ac ChIP-seq data closely resembled ChIP-qPCR data at *JUN*, *CPNE1* and *MAX* (Figs. 3A and Additional file 5: Figure S2). Regardless of peak time, all activated clusters gained H3K18ac and H3K27ac near the TSS by 1 h post-EGF treatment. AC2 genes had the highest H3K18ac peak near + 200 bp at 1 h, followed by AC3 genes. Cells treated for 6 h had the lowest H3K18ac at all clusters. Surprisingly, genes in RC1 had a slight increase in H3K18ac at 1 h compared to untreated cells and the peak at ~ + 200 bp was higher in RC1 than the one in AC1. All other repressed clusters had no significant difference in H3K18ac between untreated cells and those that were treated with EGF for 1 h and 24 h.

As has been previously shown [14], IEGs (AC1) gained H3K27ac within 1 h of EGF treatment (Fig. 4a). Like H3K18ac peaks at regulated genes, cells treated for 6 h had the lowest H3K27ac levels near the TSS at all regulated genes (Fig. 4a). Unlike H3K18ac at regulated genes, H3K27ac levels were clearly higher near the TSS of all regulated genes at 1 h and 24 h post-EGF treatment. Interestingly, H3K18ac and H3K27ac trends were similar in that they either increased or stayed near baseline levels at 1 h, decreased at 6 h and at 24 h were either similar to baseline levels or higher than baseline levels. However, in general, H3K27ac peaks were higher near activated genes compared to repressed genes (Fig. 4a). Interestingly, and like H3K18ac, RC1 genes gained more H3K27ac at 1 h and 24 h than AC1 genes at the same time points.

### Enriched motifs

Co-activator paralogs P300/CBP are recruited to genomic sites through their interactions with sequence specific proteins (i.e. transcription factors) or through their bromodomain. Once recruited, P300/CBP can deposit acetylation marks on histones (e.g. H3K18ac and H3K27ac) and non-histone proteins [33]. Therefore, to determine what transcription factor binding sites are present at the various regulated clusters of genes we analyzed the window from − 300 to + 50 of all regulated genes by cluster (Fig. 4b). AC1, AC2 and AC6 all contained transcription factor binding sites for transcription factors known to be immediately downstream of EGFR signaling, such as Activating Transcription Factor 1, 3, 7 (ATF1, ATF3, ATF7), SRF (C-Fos Serum Response Element-Binding Transcription Factor), and FRA1 (Fos-related antigen 1) (Fig. 4b). As expected, based on the gene ontology data of RC6 (Fig. 2c), RC6 genes were enriched for E2F family binding sites. Even though they were not the most significantly enriched at all clusters, every cluster contained binding sites for transcription factors known to be downstream of EGFR signaling (Fig. 4b and Additional file 6: Figure S3). One possibility is that EGFR signaling promotes the recruitment of P300/CBP to every regulated gene regardless of whether there is consequential transcription.

### EGF activates S100 genes

**Fig. 5:**
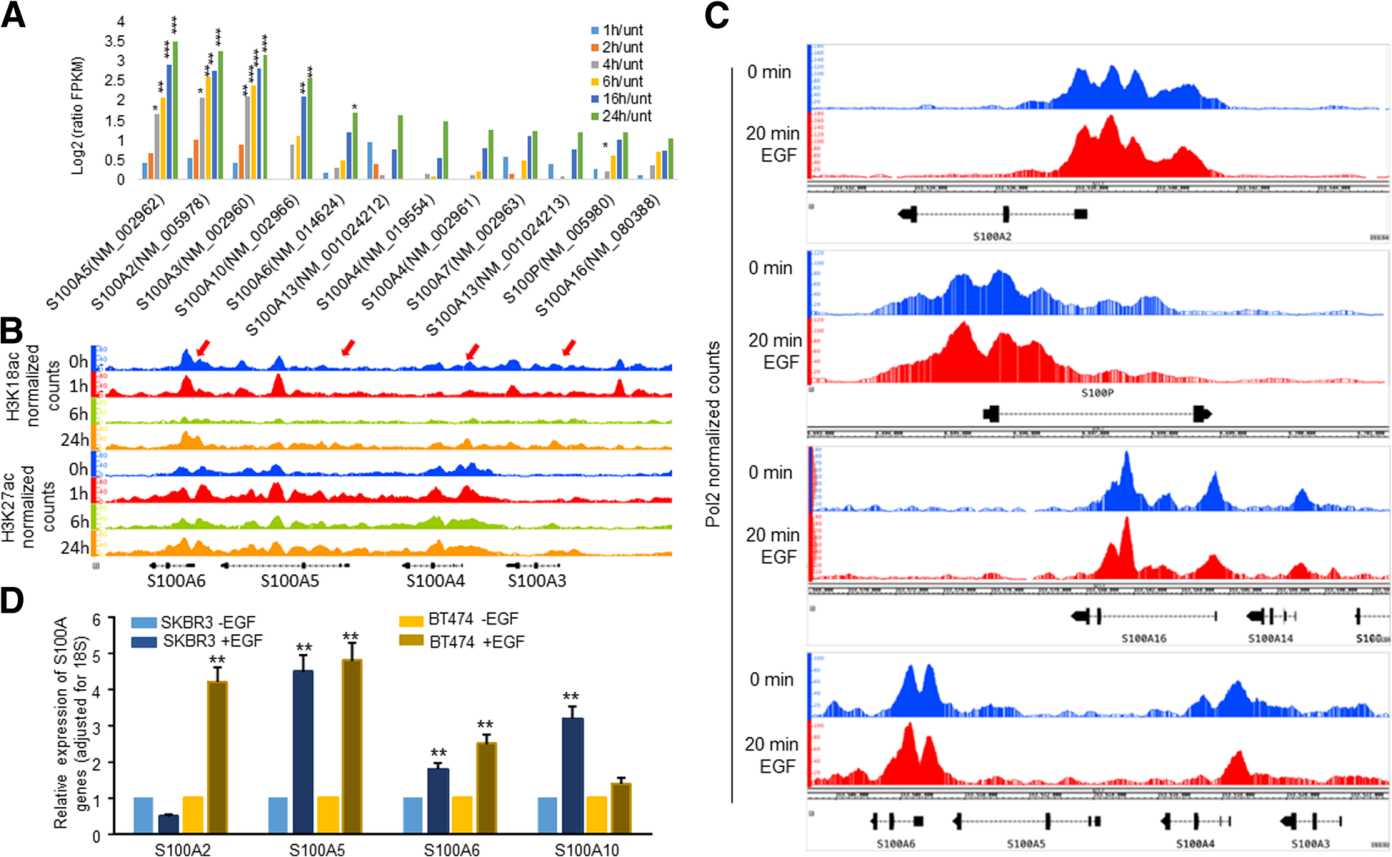
EGF upregulates S100 gene family. **a** Bar graphs are log2 ratios of (timepoint/baseline). *p-value < 0.1, **p-value < .01, ***p-value < .001 as reported by Cufflinks **b.** IGB browser view at locus containing S100A6, S100A5, S100A4 and S100A3. Red arrows indicate locations near annotated TSS for S100 genes. **c** Pol2 enrichment at S100 genes before and 20 min after EGF treatment in HeLa cells using data from Gardini et al. [12]. **d** SKBR3 and BT474 cells were serum starved for 16 h and then treated with or without EGF for 24 h. RNA from those cells were used to examine the indicated the levels of S100A genes by RT-qPCR. The bars indicated relative levels of those S100A genes adjusted for 18S. Each bar was mean of three values plus standard deviation. **P < 0.01 compared to non-EGF treatment

Looking closer at AC6, we observed that EGF activated several *S100* gene family transcripts from *S100A5*, *S100A2*, *S100A3*, *S100A10*, *S100A6*, *S100A13*, *S100A4*, *S100A7*, *S100P* and *S100A16* (Fig. 5a). S100 proteins are Ca^2+^ sensors that function in tumorigenic processes such as cell migration, metastasis, proliferation and immune evasion [16]. All S100 transcripts in AC6 increased gradually during the EGF time course. *S100A2* became activated 2-fold by 2 h post EGF treatment, while transcripts from *S100A5* and *S100A3* became activated 2-fold or more by 4 h post EGF treatment. The rest of the *S100* transcripts reached the cutoff (i.e. 2-fold over untreated cells) after the 4 h time point.

Out of 21 *S100* genes, 17 are found on chromosome 1 in a 2 Mb region known as the epidermal differentiation complex (EDC) [16]. We looked closer at H3K18ac and H3K27ac in a genomic region containing *S100A6*, *S100A5*, *S100A4* and *S100A3* (Fig. 5b). H3K18ac levels increased in the *S100A6* gene body at 1 h, even though H3K18ac peaks remained similar near the TSS. There was an overall decrease in H3K18ac at 6 h throughout the genome. H3K18ac levels recovered to near basal levels at the *S100A6* TSS at 24 h. In contrast to H3K18ac, H3K27ac levels increased dramatically at the TSS and in the *S100A6* gene body within 1 h and stayed elevated throughout the EGF time course. The annotated *S100A5* TSS contained low H3K18ac levels at all time points but did contain a noticeable H3K18ac peak within the gene body in untreated cells and cells treated for 1 h. On the other hand, H3K27ac levels increased robustly throughout the *S100A5* coding region within 1 h post-EGF treatment and remained elevated during the time course. H3K18ac levels at and near *S100A4* were similar in untreated cells and cells treated for 1 h and 24 h. In contrast, H3K27ac levels at and near *S100A4* increased at 1 h and remained elevated compared to H3K27ac levels in untreated cells.

Given the concomitant increase in *S100* gene family transcripts and histone acetylation levels we hypothesized that *S100* genes are direct EGFR signaling transcriptional targets. In order to address this we analyzed previously published ChIP-seq data for pol2 [12]. Pol2 increased in the gene body and/or near the TSS at *S100A2*, *S100P*, *S100A16* and *S100A6* within 20 min of EGF treatment in HeLa cells (Fig. 5c). Lastly, pol2 increased near the TSS of *S100A2* and *S100P* in the sense and anti-sense direction.

To confirm our finding from sequencing analysis we separately treated SKBR3 and BT474 (another HER2-positive breast cancer cell line) with EGF and conducted RT-qPCR. The data showed that *S100A5*, *S100A6* and *S100A10* were all upregulated upon EGF treatment (Fig. 5d). We could not confirm upregulation of *S100A2* by EGF in SKBR3, but it was significantly upregulated in BT474 (Fig. 5d). We also did not confirm activation of *S100A3* in any of the cell lines (data not shown).

### S100A genes and Trastuzumab resistance

**Fig. 6:**
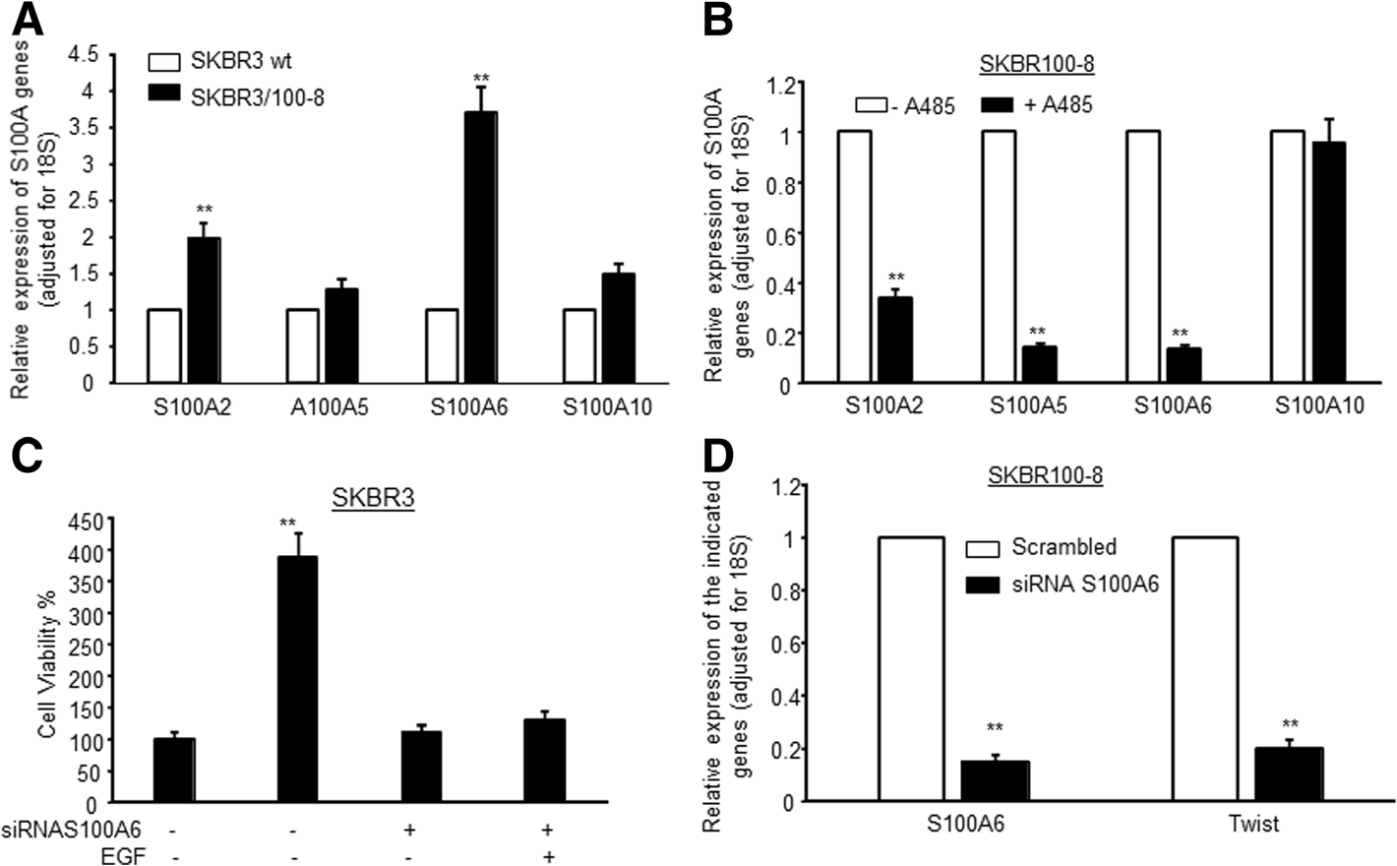
S100A genes are associated with trastuzumab resistance**. a** The levels of the indicated S100A genes in wild type SKBR3 (SKBR3 wt) and trastuzumab resistant cells, SKBR3/100–8 were examined by RT-qPCR. The bars indicated relative expressing levels of the indicated S100A genes adjusted for 18S. Each bar indicated mean of three values plus standard deviation. **P < 0.01 compared to the levels of the indicated S100A genes in SKBR3 wt. **b** SKBR3/100–8 cells were treated with 10 μM of A485 for 24 h and the levels of the indicated S100A genes were determined by RT-qPCR. The bars indicated relative expressing levels of the indicated S100A genes adjusted for 18S. Each bar indicated mean of three values plus standard deviation. **P < 0.01 compared non-treated cells. **c** SKBR3 cells were plated in 96 well plate and treated with siRNA S100A6 for 2 days, and then 20 μg of EGF were added into culture for additional 24 h. Cells viability of each condition was measured by MTT assay. The bars were mean of 6 values plus standard deviation. **P < 0.01 compared to no EGF and sRNA treatment. **d** SKBR3/100–8 cells were treated with or without siRNA S1006 for 72 h and then RNA was extracted. The levels of S100A and TWIST1 were examined by RT-qPCR. The bars indicated relative expressing levels of S100A6 and TWIST1 genes adjusted for 18S. Each bar indicated mean of three values plus standard deviation. **P < 0.01 compared non-siRNA treated cells (Scrambled)

To examine the contribution of *S100* genes in HER2-overexpressing breast cancer cells resistance to trastuzumab, we determined the levels of S100 genes in a trastuzumab resistant cell line SKBR3/100–8. SKBR3/100–8 was generated from SKBR3 through clonal selection in our laboratory. Besides acquired resistance to trastuzumab, SKBR3/100–8 cells display an EMT phenotype and express higher levels of EGFR when compared to parental SKBR3 cells [7]. SKBR3/100–8 cells express significantly higher levels of *S100A2* and *S100A6* when compared to parental SKBR3 cells. SKBR3/100–8 cells also expressed 1.5-fold higher levels of S100A10 relative to controls (Fig. 6a).

Our ChIP-seq data showed that EGF treatment induced higher expression levels of S100A genes and this coincided with an increase in H3K18ac and H3K27ac. Specifically, H3K18ac and H3K27ac levels increased at the TSS of S100 genes and in the S100A6 gene body within 1 h and stayed elevated throughout the EGF time course. To determine whether P300/CBP activity had an effect on the expression of S100 genes in HER2-overexpressing breast cancer cells, we treated SKBR3/100–8 cells with A-485, a selective small-molecule inhibitor of P300/CBP acetylase activity, and examined expression of S100 family genes by RT-qPCR. The data showed that inhibition of P300/CBP activity decreased expression of *S100A2*, *S100A5* and *S100A6* significantly in SKBR3/100–8 (Fig. 6b). The level of *S100A10* was not affected by inhibition of P300/CBP.

Since *S100A6* consistently demonstrated higher levels in all three lines we tested, we sought to determine the role of S100A6 in HER2-overexpressing breast cancer cells and in EGF/EGFR cell signaling through siRNA knockdown experiments. First we treated SKBR3 cells with an siRNA directed against *S100A6* and then treated the cells with EGF. The data showed that knockdown of *S100A6* itself did not affect cell growth, however, it did inhibit EGF-induced cell growth (Fig. 6c).

Next, we conducted *S100A6* knockdown in trastuzumab resistant cells, SKBR/100–8. Knockdown of *S100A6* decreased EMT driver, Twist1 (Fig. 6d). This suggests a role for *S100* genes, specifically S100A6, in the migratory phenotype observed in HER2-overexpressing breast cancer cells with acquired trastuzumab resistance.

## Discussion

Prior explorations into EGFR mediated modulation of transcription utilized outdated technologies or limited analysis to early time points following EGFR stimulation [8, 10, 11, 13]. Furthermore, previous investigations utilized cell lines such as HeLa, MCF10A and human keratinocytes that are not dependent on EGFR and HER2 for survival, as these cell lines are insensitive to trastuzumab treatment [34, 35]. In contrast, SKBR3 (HER2+) cells are sensitive to trastuzumab and rely on HER2 for survival [34, 36]. This makes SKBR3 cells a better model system to study clinically relevant EGFR signaling.

The analysis contained herein implemented NGS technologies to interrogate the transcriptome and chromatin landscape of SKBR3 cells following EGF treatment. We found that EGFR stimulation, over a carefully conducted EGF time course, regulated over 4000 transcripts, with similar numbers becoming activated (2038) or repressed (2029) by 2-fold or more relative to untreated cells. We observed, as has been previously shown, a wave of transcriptional activation and repression following EGF treatment (Fig. 2a and b). Our analysis of the RNA-seq data further expanded on current knowledge by searching for fluctuations in transcript abundance (and not gene expression), which better captures mRNA diversity [11].

EGF treatment of SKBR3 cells activated both immediate early genes (IEGs) and delayed early genes (DEGs) within 1 h, consistent with previous work on other cell lines. The transient activation of pAKT and pERK1/2 following EGFR stimulation is consistent to previously published data for similar experiments conducted in normal mammary epithelial cells, MCF10A. However, unlike similar experiments conducted in HeLa and MCF10A cells [17], SKBR3 cells treated with EGF have a longer duration of higher than basal levels of pERK1/2 and maintain nearly equal levels of EGFR throughout the duration of the time course.

Previous experiments showed that EGF stimulation of MCF10A activated *DUSP5* [8]. In HeLa cells, EGF treatment activated *DUSP1, DUSP2* and *DUSP5*. DUSP (dual specificity phosphatase) family of proteins dephosphorylate phospho-serine, phospho-threonine and phospho-tyrosine residues on their substrates and they are known to antagonize MAPK signaling [37]. In addition, *ZFP36* (ZFP36 Ring Finger Protein) and *ZFP36L1 (ZFP36 Ring Finger Protein Like1)* also became activated within 1 h. ZFP36 and ZFP36L1 are known to destabilize AU-rich element-containing (ARE) transcripts and ZFP36 has previously been shown to promote *FOS* degradation [8]. In addition, there is evidence that some DEGs, such as the DUSP (Dual-specificity phosphatase) proteins, attenuate EGFR signaling and it is conceivable that in HER2+ SKBR3 cells the same is true. Therefore, it is likely that similar transcriptional dynamics exist in SKBR3 and HeLa cells during the early phase of EGFR stimulation. In short, the RNA-seq data for AC1 are consistent with previously published data sets but add to the body of literature of IEGs downstream of EGFR stimulation.

KDM3A (Lysine Demethylase 3A) and KDM6B (Lysine Demethylase 6B) are demethylases involved in removing repressive H3K27 methyl marks [38], and they clustered into AC2 (Additional file 2: Table S1). Given the robust induction of H3K27ac at activated and repressed genes it is possible that KDM6B plays a role in removing H3K27 methyl marks prior to the deposition of H3K27 acetylation marks. Mapping genome-wide H3K27 methyl modifications remains uninvestigated in EGF treated SKBR3 cells. This is made more interesting by noting that *EZH2* also clustered into AC2 (Additional file 2: Table S1). EZH2 (Enhancer Of Zeste 2 Polycomb Repressive Complex 2 Subunit) is a component of the Polycomb Repressive Complex 2 (PRC2), which is involved in depositing H3K27me3 (histone H3 lysine 27 trimethylation) marks and therefore silencing chromatin [39]. Therefore, determining the genome-wide localization of KDM3A, KDM6B and EZH2 during EGFR stimulation could provide insights into additional mechanisms of gene regulation during EGFR signaling.

The transcripts in AC6 play a role in extracellular matrix organization, cell junction organization and cell-cell adhesion (Fig. 2c). EGF is known to induce cell migration in several cell lines, including HeLa, MCF10A and human keratinocytes. Our RNA-seq data adds to the number of genes downstream of EGF that could be involved in cell migration. *WNT9A*, a gene in AC6, is induced over 2-fold relative to untreated cells within 2 h of EGF treatment. WNT9A is known to affect morphogenesis in several species and in different cell types [40–42]. In addition, *WNT9A* is expressed at much higher levels in JIMT1 (a trastuzumab resistant cell line) compared to SKBR3 cells (a trastuzumab sensitive cell line) (data not shown).

An interesting group of transcripts in AC6 belong to the *S100* gene family (Additional file 2: Table S1). EGF induced transcripts, by 2-fold or more relative to untreated cells, of 10/21 *S100* genes encoded in the human genome. The increase in H3K18ac and H3K27ac levels following EGFR stimulation was consistent with chromatin regulation near the S100-gene-containing locus on chromosome 1 (Fig. 5b). Moreover, the recruitment of pol2 to several *S100* gene family members within 20 min of EGF treatment in HeLa cells, suggests that some *S100* genes are direct EGFR targets (Fig. 5c). Furthermore, our data confirmed that *S100A6* and *S100A2* were significantly increased in trastuzumab resistant breast cancer cells, SKBR3/100–8 (Fig. 6a). Inhibition of P300/CBP by A485 significantly downregulated *S100A2*, *S100A5* and *S100A6* in SKBR3/100–8 (Fig. 6b). Trastuzumab resistance can develop from increased HER family receptor signaling. Trastuzumab reduces HER2-mediated signaling, but it may not inhibit signaling from other HER2 family receptors’ crosstalk, such as EGFR (HER1) /HER2 [43]. In contrast to HER2-negative breast cancer, HER2-positive breast cancer expresses higher levels of EGFR [44] and exhibits increased signaling from HER family receptors, such as EGFR, as well as EGFR/HER2 crosstalk [45]. These are some of the molecular mechanisms that contribute to trastuzumab resistance. Data from our laboratory showed an increased expression of EGFR in HER2-overexpressing breast cancer cells that had acquired resistance to trastuzumab [7]. Altered EGFR signaling in tumors could result in phenotypic changes and transcriptional changes which could promote EMT, and increase cell proliferation, invasiveness and increased motility [46]. Breast cancer cells undergoing EMT also exhibit a drug and trastuzumab resistance phenotype [7, 47–49]. S100A6 has been suggested to promote EMT through β-Catenin in a pancreatic cancer cell line [50]. We also showed in this study that knockdown of *S100A6* by siRNA in SKBR3/100–8 also decreased the expression of EMT driver, Twist1 (Fig. 6d). RNAi mediated depletion of *S100A6* also inhibited EGF-induced cell growth (Fig. 6c). The data suggests that EGF mediated induction of *S100* genes could be a mechanism by which HER2-overexpressing breast cancer cells develop resistance to trastuzumab. *S100A6* could be potential target for treating HER2 and EGFR positive breast cancer.

Although we did not assess migratory characteristics, it is well documented that EGFR stimulation causes a migratory phenotype in various cell lines [8, 9]. The transcriptional profile illustrated in our data suggests that this is also the case in SKBR3 cells. This is interesting because of the strong association observed between Epithelial-Mesenchymal-Transition and trastuzumab resistance [51, 52]. Moreover, the multidrug resistance phenotype is enhanced in breast cancer cells that have activated MAPK/ERK pathways [53]. Interestingly in our experiments, pEGFR remained higher than baseline levels throughout the 24 h period, even though pERK1/2 and pAKT were back to baseline levels by 1–2 post-EGF treatment, suggesting that other pathways could become activated by EGFR signaling.

In addition, S100 genes are known to be overexpressed in certain cancers and have been the focus of research groups interested in developing novel drug targets [16]. To that end, paquinimod and tasquinimod have been tested as small molecule inhibitors of S100A8 and S100A9. Tasquinimod has demonstrated promising results, as phase II and phase III clinical trials have indicated positive relapse free survival (RFS) in prostate cancer patients [54, 55]. Additional research on the function of S100 proteins in HER2+ breast cancer disease must be conducted and is currently underway in our laboratory.

Our H3K18ac and H3K27ac ChIP-seq data, demonstrates for the first time that the chromatin landscape has been assessed in an EGF treatment time course of HER2+ breast cancer cells. H3K18ac increased near the TSS of all activated genes and at RC1 within 1 h, suggesting that regardless of when genes peaked in expression, the chromatin landscape was rapidly altered (Fig. 4a). On the other hand, H3K27ac levels increased at all regulated genes and remained higher than H3K27ac levels in untreated cells at 1 h and 24 h post-EGF treatment (Figs. 3d and 4a). This strongly suggests that chromatin regulation does not account for all of the fluctuations in gene expression observed in EGF treated SKBR3 cells. To that end, other groups have demonstrated that microRNAs play a role in modulating mRNA’s in EGF treated MCF10A cells [9, 13]. Nonetheless, studying the role of microRNAs in EGF treated SKBR3 cells using NGS technologies remains unexplored and might provide key differences of post-transcriptional regulation of gene expression. Moreover, other groups have shown that gene expression in EGF treated cells is regulated at the “pause” step in transcription [12, 15]. Therefore, it would be interesting to explore pol2 recruitment in EGF treated SKBR3 cells. Lastly, given the oscillations in H3K18ac and H3K27ac during the EGF time course, it would be insightful to know the genome-wide locations of P300/CBP, the acetylases that deposit those marks.

## Conclusion

We report here the first study to examine the modulation of gene regulation by EGFR/HER2 in a clinically relevant HER2+ breast cancer cell line. Dysregulated gene expression is a hallmark of cancer [56]. Oncogenic signaling directly affects chromatin structure, as our data has demonstrated [57]. The implementation of NGS technologies to the field of cancer biology has provided mechanistic insights into how cancer cells differ from normal cells. This has facilitated the discovery of novel drug targets. Trastuzumab resistance is a clinical reality, with many patients facing recurring breast cancer disease [5, 6]. In addition, HER2+ breast cancer disease is discovered in more advanced stages in African-American women at a point when trastuzumab therapy is less effective [58, 59]. Therefore, our results have added to the list of likely drug targets, some of which we are already pursuing. Targeting S100 proteins may prevent EGF induced tumor cell growth and metastasis and can complement trastuzumab therapy.

## Additional files

Additional file 1: **Table S3.** Primers used in this study. (DOCX 11 kb)

Additional file 2: **Table S1.** Log2 ratios of activated genes listed by cluster. (PDF 2233 kb)

Additional file 3: **Table S2.** Log2 ratios of repressed genes listed by cluster. (PDF 2231 kb)

Additional file 4: **Figure S1.**Genes differentially expressed by cluster. A. Distributions of FPKMs plotted as boxplots for transcripts induced 2-fold (AC = activated cluster) B. Distributions of FPKMs plotted as boxplots for transcripts repressed 2-fold or more (RC = repressed cluster). ***p-value < .001 two-sided t-test for significant difference between Untreated and EGF treated samples. (TIF 158 kb)

Additional file 5: **Figure S2.** EGFR signaling modulates chromatin at activated and repressed genes. IGB browser views of ChIP-seq H3K18ac and H3K27ac data at loci containing JUN, CPNE1 and MAX. Red arrows indicate annotated TSS. (TIF 213 kb)

Additional file 6: **Figure S3.** Clusters contain motifs for TF known to be downstream of EGFR signaling. Motifs results as indicated by HOMER search from -300 bp to + 50 bp of each cluster. All motifs shown were enriched at *p*-value <.01. (TIF 230 kb)

## Abbreviation

AC: Activated gene cluster
ADCC: Antibody-dependent cell-mediated cytotoxicity
ARE: AU-rich element-containing
ATF1, ATF3, ATF7: Activating Transcription Factor 1, 3, 7
bZIP: Basic leucine zipper
CDC25A: M-phase inducer phosphatase 1
CDC45: Cell Division Cycle 45
CDKN1A (p21): Inhibitor of G1 Cyclin Dependent Kinases (CDKs)
CDT1: DNA replication factor
ChIP: Chromatin Immunoprecipitation
ChIP-seq: ChIP-sequence
CLDN 9 and CLDN12: Claudin (CLDN) family members 9 and 12
CPNE1: Copine-1
CtBP: C-terminal-binding protein and Sin3A (Histone Deacetylase Complex Subunit Sin3a)
DEGs: Delayed early genes
DU-miRs: Delayed up-regulated microRNAs
DUSP: Dual-specificity phosphatase
DUSP1, DUSP4, DUSP5 and DUSP8: Dual Specificity Phosphatase 1, 4, 5, and 8
E2F1 and E2F2: E2F Transcription Factor 1 and 2
EGF: Epidermal Growth Factor
EGFR/HER1: The Human Epidermal Growth Factor Receptor
EGR1: Early growth response protein 1
EGR2: Early growth response protein 2
EMT: Epithelial–mesenchymal transition
eRNAs: Enhancer RNAs
EZH2: Enhancer Of Zeste 2 Polycomb Repressive Complex 2 Subunit
FHL2: Four-and-a-half LIM domains protein 2
FOS: Fos Proto-Oncogene
FPKM: Fragments Per Kilobase of transcript per Million mapped reads
FRA1: Fos-related antigen 1
GO: Gene ontology
H3K18ac: Histone H3 lysine 18 acetylation
H3K27ac: Histone H3 lysine 27 acetylation
H3K27me3: Histone H3 lysine 27 trimethylation
HER: Human epidermal growth factor receptor
HER2: Human epidermal growth factor receptor-2
HER3: Human epidermal growth factor receptor-3
HER4: Human epidermal growth factor receptor-4
ID1: DNA-Binding Protein Inhibitor ID-1
ID-miRs: microRNAs
IEGs: Immediate early genes
ITGA5: Integrin Subunit Alpha 5
IU-miRs: Immediately up-regulated microRNAs
JUN: Jun Proto-Oncogene
KDM3A: Lysine Demethylase 3A
KDM6B: Lysine Demethylase 6B
KEGG: Kyoto Encyclopedia of Genes and Genomes
KLF2, KLF10, KLF4, KLF7: Krüppel-like Factor 2, 10, 4, 7
KRT17: Keratin 17
MAF: MAF BZIP Transcription Factor
MAFF: MAF BZIP Transcription Factor F
MAPK: Mitogen-activated protein kinase
MAX: Myc-associated factor X
MCM: Minichromosome maintenance
MMP9: Matrix Metallopeptidase 9
NGS: Next Generation Sequencing
NR4A1: Nuclear Receptor Subfamily 4 Group A Member 1
p300/CBP: E1A binding protein p300/ CREB-binding protein
pAKT: Activated AKT/protein kinase B
PCNA: Proliferating cell nuclear antigen
pERK1/2: Activated Extracellular signal-regulated kinase 1/2
Pol2: RNA polymerase II
PRC2: Polycomb Repressive Complex 2
RC: Repressed gene cluster
RFS: Relapse free survival
RNA-seq: mRNA-sequencing.
RTK: Receptor tyrosine kinase
S100: S100 Calcium Binding Protein
SRF: C-Fos Serum Response Element-Binding Transcription Factor
TFBS: Transcription factor binding site
TGF-α: Transforming Growth Factor alpha
TSS: Transcription start site
WNT9A: Wingless-Type MMTV Integration Site Family, Member 9A
ZFP36: ZFP36 Ring Finger Protein
ZFP36: zinc finger protein 36 homolog
ZFP36L1: ZFP36 Ring Finger Protein Like1
