# Supplementary material for: Transcriptomic and ChIP-sequence interrogation of EGFR signaling in HER2+ breast cancer cells reveals a dynamic chromatin landscape and S100 genes as targets
Source: BMC Med Genomics. 2019 Feb 8;12:32. doi: 10.1186/s12920-019-0477-8 (PMC6368760; doi:10.1186/s12920-019-0477-8)
Supplement: Supplementary file 1 — Table S3. Primers used in this study. (DOCX 11 kb) [file 12920_2019_477_MOESM1_ESM.docx]

**Table S3. Primers**

| **qRT-PCR Primers** | **ChIP-PCR Primers** |
| --- | --- |
| EGR2_F1 GGCCCCTTTGACCAGATGAA  EGR2_R1 AGCTGCTGGGATATGGGAGA JUN_F1 GAGCTGGAGCGCCTGATAAT JUN_R1 CCCTCCTGCTCATCTGTCAC KLF2_F1 CCTGCAGGAGCGCTGG KLF2_R1 GCCCCATGGACAGGATGAAG | JUN_ChIP_F1 CCTCCATCAGAGGTTGCGAG JUN_ChIP_R1 CATTGTGGGCTGACGTCTTG CPNE1_ChIP_F1 AAGCACAATTCGCTCCTTCC CPNE1_ChIP_F2 AGGTAAGCACAATTCGCTCCT MAX_ChIP_F1 TAGGCGCAAAACGGAACCA MAX_ChIP_R1 ATGCTGCCTCTCTTTGGTGT |
