# Supplementary material for: Transcriptomic and ChIP-sequence interrogation of EGFR signaling in HER2+ breast cancer cells reveals a dynamic chromatin landscape and S100 genes as targets
Source: BMC Med Genomics. 2019 Feb 8;12:32. doi: 10.1186/s12920-019-0477-8 (PMC6368760; doi:10.1186/s12920-019-0477-8)
Supplement: Supplementary file 2 — Table S1. Log2 ratios of activated genes listed by cluster. (PDF 2233 kb) [file 12920_2019_477_MOESM2_ESM.pdf]

Table S1. Log2 ratios of activated genes listed by cluster

test_id	gene_id	1h/unt	2h/unt	4h/unt	6h/unt	16h/unt	24h/unt	cluster *
NM_006732	FOSB	8.20748	7.37221	2.84045	1.21167	1.72840	1.89995	ac1
NM_005252	FOS	5.39741	1.49265	-0.09619	-0.95748	0.79899	0.95226	ac1
NM_001030287	ATF3	5.22447	4.93065	1.54751	-0.02266	1.05025	1.01112	ac1
NM_001901	CTGF	4.95262	4.00000	-0.22628	-1.06682	0.31178	0.34731	ac1
NM_004417	DUSP1	4.82365	1.31705	-0.01297	-0.43676	1.07638	1.19329	ac1
NM_001206486	ATF3	4.48427	3.14029	0.43609	-0.35113	1.75718	2.23903	ac1
NM_173157	NR4A1	4.22600	4.15110	-2.01998	-2.84760	-0.48419	-0.41286	ac1
NM_001964	EGR1	4.19605	1.67689	-0.39799	-1.09553	1.01387	1.06230	ac1
NM_002228	JUN	4.14591	1.22957	-0.06722	-0.24198	0.39961	0.65681	ac1
NM_001674	ATF3	4.00645	3.56881	0.56000	-1.11532	0.83482	1.26626	ac1
NM_003407	ZFP36	3.81197	2.00524	0.35682	-0.35199	1.15798	1.41611	ac1
NM_001300	KLF6	3.71439	2.73436	0.82004	0.90372	0.75869	0.69575	ac1
NM_001554	CYR61	3.48899	2.88671	0.91816	0.62920	-0.05045	0.17613	ac1
NM_016270	KLF2	3.43936	1.60606	-1.11743	-0.67142	0.38720	0.49273	ac1
NM_001955	EDN1	3.22013	1.91671	1.48260	1.25421	0.57809	-2.03529	ac1
NM_014330	PPP1R15A	3.20811	2.77461	1.83615	1.66663	1.34838	1.73804	ac1
NR_039872	MIR4721	3.12879	2.39804	1.13333	2.35320	1.69657	2.13678	ac1
NM_004419	DUSP5	2.99285	2.87683	1.39199	0.96867	2.16123	2.33204	ac1
NM_000594	TNF	2.88590	2.21140	1.92058	0.72994	1.49450	1.43518	ac1
NM_173354	SIK1	2.80713	1.00491	-0.35299	-0.35486	-0.29585	-0.48984	ac1
NM_001242487	KIAA0913	2.74333	2.49111	1.41243	0.63393	1.66189	1.40858	ac1
NM_001185094	NIT1	2.70403	0.70459	2.46757	0.48342	0.42682	0.74675	ac1
NM_001164783	BCKDHA	2.58739	1.83323	1.41502	1.97719	2.23843	0.73197	ac1
NM_002229	JUNB	2.56388	1.87807	0.52641	0.08764	1.72410	2.09654	ac1
NM_005676	RBM10	2.27677	2.15084	1.86062	1.26631	2.01992	2.00461	ac1
NR_000006	SNORD21	2.27618	0.82273	-0.15923	0.83829	1.06451	0.85471	ac1
NM_003955	SOCS3	2.21314	0.60488	0.03123	-0.10122	1.42283	1.96750	ac1
NR_002995	SNORA76	2.17777	1.23612	1.08237	1.01887	0.62089	1.91958	ac1
NM_001098504	DDX17	2.17308	1.69889	0.88175	1.15772	0.29055	1.15294	ac1
NM_002928	RGS16	2.17035	0.19398	-0.27072	-0.75339	-0.26607	-0.55005	ac1
NM_001171135	ZBED1	2.15855	1.99487	1.68469	1.09571	0.02400	1.58492	ac1
NM_017920	URGCP	2.13117	1.49796	0.50800	-1.82110	-0.05979	0.17909	ac1
NM_004729_1	ZBED1	2.11351	1.88979	0.41400	0.66108	0.84709	0.67874	ac1
NM_001025236	TSPAN4	2.09463	0.32158	1.39436	1.21589	-0.14861	0.75577	ac1
NM_173475	DCUN1D3	2.07734	1.59285	0.31408	0.10916	0.36411	0.46289	ac1
NM_001032391	LCMT1	2.02086	1.22345	0.44960	1.86973	1.77351	1.38951	ac1
NM_032322	RNF135	2.01169	0.98389	0.95647	0.06939	0.85788	0.99793	ac1
NM_004729	ZBED1	2.01114	0.70308	-0.35092	-0.58243	0.10991	-0.92807	ac1
NM_203418	RCAN1	1.98151	1.96926	-0.05881	-0.43677	-0.40169	-0.31166	ac1
NM_004907	IER2	1.97442	1.18788	0.25278	0.08483	0.83554	1.16610	ac1
NM_001130020	ATP6V0A1	1.97075	-0.50251	-1.01789	0.74219	0.71018	1.19207	ac1
NM_004040	RHOB	1.93363	1.34061	-0.87195	-0.94586	-0.58833	-0.57001	ac1
NR_045022	C17orf76-AS1	1.86577	-0.33629	0.26551	1.57394	1.29194	0.03900	ac1
NM_014470	RND1	1.85680	1.30021	-0.48790	-0.39887	0.15818	0.25332	ac1
NR_002910	SNORA71B	1.84093	1.19680	1.42955	1.74116	0.17214	1.04956	ac1
NM_001256355	UBE2L3	1.83835	-0.73535	0.16973	-1.41772	0.83659	0.00729	ac1

NM_001242369	C9orf7	1.82262	0.16600	-0.33933	1.52540	1.21952	1.52789	ac1
NM_001128855	GTPBP3	1.81187	1.01361	-0.55093	-0.63651	0.49339	0.71738	ac1
NM_001005502	CPM	1.76123	0.24616	0.27502	1.68281	0.30703	0.43125	ac1
NM_015675	GADD45B	1.74860	0.68881	-0.49350	-0.76864	0.61645	0.83523	ac1
NM_007326	CYB5R3	1.69880	1.10073	0.86736	-3.87714	-2.92646	-4.14664	ac1
NM_052901	SLC25A25	1.68764	1.21115	0.65730	-0.10720	0.29462	0.37018	ac1
NM_025079	ZC3H12A	1.68062	1.59415	0.29944	-0.05326	0.78355	1.10896	ac1
NM_003897	IER3	1.67760	1.19292	0.76313	0.35799	1.27981	1.36574	ac1
NM_002167	ID3	1.67191	1.10631	0.23381	0.31179	1.46426	1.62900	ac1
NM_001956	EDN2	1.66999	0.86197	-0.49259	-0.97815	-2.90185	-2.68497	ac1
NM_020307	CCNL1	1.65588	0.95785	-0.01434	-0.02117	0.03737	0.21899	ac1
NM_001146020	TJAP1	1.63849	1.39195	0.78711	0.51438	1.49866	1.55018	ac1
NM_173208	TGIF1	1.63197	0.34322	0.03589	-0.05574	0.71962	0.62390	ac1
NM_006622	PLK2	1.59241	0.82354	0.18736	0.34282	-0.73812	-0.98539	ac1
NM_013368	SERTAD3	1.59166	-0.03037	-0.21371	-0.11188	0.14589	0.04592	ac1
NM_001131023	PEX5	1.57017	0.09195	1.00798	0.06031	1.49894	0.98994	ac1
NM_003670	BHLHE40	1.56715	0.89752	0.94604	0.57405	0.63717	0.63947	ac1
NM_006763	BTG2	1.54835	1.12549	0.44436	-0.28042	0.31945	0.23108	ac1
NM_001005911	IP6K2	1.54399	1.17078	0.77169	0.73708	-0.16230	0.16918	ac1
NM_001135663	RAB7L1	1.52170	1.18711	0.72751	1.26014	1.25065	0.76830	ac1
NM_001171135_	ZBED1	1.50010	1.27714	0.57293	-0.16385	0.10353	-0.38579	ac1
NM_001113361	TBC1D14	1.49947	0.60755	1.07463	1.37076	0.75177	0.88312	ac1
NM_004197_8	STK19	1.48283	1.21014	0.75585	1.30550	0.91744	1.15206	ac1
NM_181454	MRPL55	1.47152	1.05252	0.90876	1.11908	0.73909	1.43827	ac1
NR_037421	MIR3648	1.42878	1.35608	0.92474	0.36721	0.82157	0.95782	ac1
NM_001159767	BZW2	1.42567	-0.08169	-0.74268	0.25936	-0.27798	-0.67488	ac1
NM_001202468	ZMYND11	1.42328	0.16236	0.75215	0.83012	0.95312	0.65639	ac1
NR_003068	SNORD88B	1.42232	0.31209	0.56216	0.19178	-1.19730	0.54941	ac1
NM_001164817	ACIN1	1.41108	-0.20203	0.59654	1.06218	0.37348	0.70452	ac1
NM_203414	C17orf81	1.38636	0.75180	0.91344	0.40013	1.10047	1.16505	ac1
NM_018087	TMEM48	1.37783	1.02028	0.71369	0.82615	1.36137	0.83667	ac1
NM_052827	CDK2	1.35481	-0.31639	-0.43791	0.63413	0.43553	-0.79134	ac1
NM_001195328	RAB9A	1.31274	1.03596	1.12545	0.73734	1.03787	0.77863	ac1
NM_178234	TUSC3	1.31214	0.78053	-0.10691	0.21752	-0.50900	1.24928	ac1
NM_001113525	ZNF276	1.30380	0.85522	0.59728	0.95637	0.12350	0.26224	ac1
NR_024203	ECD	1.29947	0.99527	-0.72446	0.82353	-0.44852	-0.70443	ac1
NM_194278	C14orf43	1.28880	0.11951	0.37847	0.58548	0.22244	0.29249	ac1
NM_033486	CDK11B	1.27705	-2.52913	0.10441	0.78120	-0.69493	0.96860	ac1
NM_001127585	ING4	1.27344	-1.31734	-0.51300	0.00124	-0.61866	-0.41052	ac1
NM_024111	CHAC1	1.26213	0.44393	0.09148	-0.02828	0.23161	0.70646	ac1
NM_002467	MYC	1.26030	1.12727	0.75456	0.46457	-0.25034	-0.22824	ac1
NR_026705	VTRNA1-3	1.25647	-0.25173	0.45675	-0.21797	-0.12577	1.07293	ac1
NM_018386	PCID2	1.24167	1.07496	-0.06598	-0.50925	1.03583	-1.21892	ac1
NR_036581	LOC10028925	1.24121	0.82707	-0.20795	-0.21757	0.13866	0.28107	ac1
NR_036518	C17orf62	1.23987	1.21766	-0.95885	-1.00972	1.11839	-0.34847	ac1
NM_020218	ATXN7L3	1.23521	0.48149	0.48517	-0.37460	0.01429	0.71003	ac1
NM_032036	IFI27L2	1.22712	-0.41711	0.15779	0.55881	-0.64315	0.51320	ac1

NM_001200018	NAT6	1.22209	1.14579	-0.12511	-0.68348	0.22864	0.03720	ac1
NR_029472	SNRPC	1.21684	0.74069	0.81213	0.53952	0.35903	-0.39618	ac1
NM_001171819	PPARD	1.21332	0.67837	0.58043	-0.32531	0.56665	0.11274	ac1
NM_004420	DUSP8	1.21262	1.01374	-0.74363	-0.87438	0.38316	0.72876	ac1
NM_030952	NUAK2	1.20050	0.28705	-0.09638	-0.26633	-0.01945	0.07294	ac1
NM_001098272	HMGCS1	1.18923	0.90756	0.23354	0.19614	0.50268	-0.08798	ac1
NM_001394	DUSP4	1.18706	1.06842	0.93154	0.68721	0.95966	0.93133	ac1
NM_001078175	SLC29A1	1.18431	0.95822	0.38606	0.90653	-0.50797	-1.68572	ac1
NR_033334	TMEM70	1.16485	0.40373	-0.86977	0.30893	-0.16395	-0.32481	ac1
NR_027438	IP6K2	1.16316	0.57869	0.16700	-0.26743	0.57271	0.39201	ac1
NR_045106	C15orf44	1.15267	0.94509	0.33419	0.68984	0.65691	0.33845	ac1
NM_001162997	C17orf110	1.15066	0.70075	0.73450	0.40702	0.51833	0.98983	ac1
NR_002967	SNORA31	1.14685	0.28750	-0.28291	1.04197	-0.60050	0.80046	ac1
NR_040055	LOC10050668	1.14622	0.34940	-0.15764	0.25555	-1.41038	-0.77697	ac1
NM_133263	PPARGC1B	1.14265	-0.30682	0.55600	0.84351	-1.21604	-1.35742	ac1
NR_023318	ADPGK	1.13986	-0.03266	0.80675	-0.10494	0.83684	0.56547	ac1
NM_130474	MADD	1.13969	0.51254	-0.98238	0.17320	1.00004	0.90747	ac1
NM_001242892	CSDE1	1.12876	0.85901	-0.16303	1.12490	0.75607	0.42106	ac1
NM_001145714	SAE1	1.12117	-0.09336	0.05407	-0.25648	-1.19817	0.42809	ac1
NM_001135553	MKNK1	1.12089	-0.43206	-0.44266	-0.97271	-0.45603	0.68403	ac1
NR_002986	SNORA60	1.11918	0.24661	0.89820	-0.67776	-0.41288	-0.93379	ac1
NM_003591	CUL2	1.11765	0.52075	0.71127	0.82546	0.91174	-0.30739	ac1
NM_198309	TTC8	1.11316	0.83478	0.58375	0.24832	-0.53882	-0.16230	ac1
NR_002585	SNORA52	1.10765	0.20238	-0.59498	1.00416	-1.30145	0.16249	ac1
NM_016003	WIPI2	1.10707	0.68431	0.20084	0.24352	0.87908	0.88493	ac1
NM_001136575	LANCL1	1.10681	0.08746	0.31860	1.06611	0.03062	-0.53843	ac1
NM_001198869	CAPN1	1.10599	-0.06342	-0.22862	-0.64581	0.33722	0.36306	ac1
NM_130443	DPP3	1.09180	-0.69277	-0.17391	-0.06352	0.67811	-0.47296	ac1
NM_001164410	CDK5	1.08985	0.44645	-0.19805	0.46698	0.32034	0.17819	ac1
NM_138362	FAM104B	1.08709	0.72653	0.54829	0.62113	-0.48537	-0.61064	ac1
NM_001244701	ZFP36L1	1.08670	0.19574	0.35381	0.20183	0.49920	0.37697	ac1
NM_201569	SMG7	1.07423	-0.52828	0.81113	0.24852	0.42118	0.43500	ac1
NM_203344	SERTAD3	1.07283	0.23941	-0.66174	-0.34237	0.66910	0.88290	ac1
NM_001171628	VEGFA	1.06842	1.06745	1.03444	0.99333	0.75569	0.87510	ac1
NM_017885	HCFC1R1	1.05953	0.22620	-0.32104	0.04973	0.27865	0.01107	ac1
NM_134427	RGS3	1.05703	-0.31928	-1.45626	-1.50702	0.69006	0.98997	ac1
NM_032711	MAFG	1.04799	-0.72366	-0.49529	-0.96348	-1.31300	-0.88883	ac1
NR_002590	SNORA41	1.03962	-0.86925	-1.50550	0.87103	-1.27832	-0.23473	ac1
NM_016272	TOB2	1.02869	0.86372	0.21682	0.04737	0.42559	0.64002	ac1
NR_000015	SNORD55	1.02836	0.49688	0.26933	0.72800	-0.12598	0.74148	ac1
NM_001077493	NFKB2	1.02834	0.18993	0.49744	0.06247	0.03972	0.07643	ac1
NR_037591	AK2	1.02763	-0.41101	-2.16068	0.39124	-1.10992	0.03151	ac1
NM_001184721	GYG1	1.02379	-0.18405	-0.12083	-0.23846	-1.00209	-2.48923	ac1
NM_001136007	FXYD3	1.01267	0.31058	0.01435	0.37485	-0.09469	0.64047	ac1
NM_182811	PLCG1	1.00500	0.63266	-1.10312	-1.73687	0.47376	0.29245	ac1
NM_001164759	PRKAR1B	1.00048	0.64329	0.56856	-0.44472	-0.27230	-1.64455	ac1
NM_001657	AREG	1.93242	4.35282	4.24423	3.66677	3.50798	3.05570	ac2

NR_030367	MIR637	3.58939	3.90053	3.30402	1.58153	3.27547	3.75576	ac2
NM_003246	THBS1	2.75802	3.78096	3.15742	2.87815	0.91985	0.69065	ac2
NM_001199622	NCOA7	2.91763	3.59513	1.22571	-0.12498	0.97928	0.91560	ac2
NR_002958	SNORA17	0.13447	3.27179	0.95429	-0.59179	3.13158	2.14728	ac2
NM_001161573	MAFF	2.58520	3.15375	1.58337	0.38880	0.99916	1.18074	ac2
NM_001126337	TUFT1	2.33854	3.09285	0.50071	0.41434	1.50572	1.51298	ac2
NM_005967	NAB2	0.57572	3.03885	2.02241	1.17735	1.70905	1.86948	ac2
NM_139351	BIN1	2.59401	2.98606	2.78799	1.41392	2.61882	2.33288	ac2
NM_001146030	SEMA7A	1.07660	2.83026	2.24657	1.69005	1.89307	2.27894	ac2
NR_000019	SNORD34	2.29429	2.72924	1.17795	-0.03096	0.35811	1.54338	ac2
NM_005985	SNAI1	1.47010	2.72917	1.70974	0.75518	2.31797	2.64348	ac2
NM_013376	SERTAD1	1.89502	2.62579	1.77535	1.11024	1.31747	1.58752	ac2
NM_001254738	RND3	1.24298	2.61877	0.03688	0.63559	-0.36433	0.50762	ac2
NM_012323	MAFF	2.15071	2.60851	0.85478	0.61297	1.97391	2.35867	ac2
NM_005655	KLF10	1.32353	2.59313	0.80316	0.76844	0.99934	0.75473	ac2
NM_014583	LMCD1	1.05838	2.55247	1.51189	1.53853	-0.29925	-0.23625	ac2
NM_004431	EPHA2	1.08915	2.45036	1.99012	1.49955	1.63856	2.09136	ac2
NM_016545	IER5	1.76319	2.39765	1.50647	1.02290	1.27780	1.56679	ac2
NM_018948	ERRFI1	1.39728	2.34929	1.40276	0.98120	1.74612	1.20081	ac2
NM_001128920	MARK3	2.19040	2.31760	2.15968	1.59469	1.97648	2.04733	ac2
NM_033027	CSRNP1	2.16746	2.30595	1.36162	0.96824	1.01378	1.35775	ac2
NM_080648	APEX1	2.16835	2.28285	1.04334	1.79787	-2.65447	0.48102	ac2
NM_001143964	TBC1D7	1.21888	2.21259	1.69557	0.83928	1.72247	2.03706	ac2
NM_181353	ID1	1.34559	2.17507	-0.19426	0.71742	1.45259	1.36769	ac2
NM_152998	EZH2	1.80937	2.13337	1.56093	2.07146	0.82622	-0.11766	ac2
NM_012118	CCRN4L	1.04214	2.10657	1.57672	1.05728	0.08784	0.33044	ac2
NM_003131	SRF	1.48531	2.08443	1.44144	0.55683	0.51108	0.92003	ac2
NM_001205252	RNF223	1.75660	2.07264	0.45135	0.11907	0.65790	0.84595	ac2
NM_031308	EPPK1	1.47880	2.04626	1.38304	0.45610	0.39540	0.44729	ac2
NR_002920	SNORA8	0.81086	2.04419	0.65529	1.98373	0.07677	1.81446	ac2
NM_001080424	KDM6B	1.01230	2.01625	0.67461	0.04656	0.80847	0.92263	ac2
NM_182685	EFNA1	1.49048	1.98021	1.18335	0.64023	0.41204	1.28145	ac2
NM_014779	TSC22D2	0.36202	1.97878	1.12690	1.08009	0.71632	0.61794	ac2
NM_025194	ITPKC	0.71338	1.97647	1.37323	0.65369	0.97699	1.32345	ac2
NM_183373	PXDC1	1.04038	1.91343	1.27548	0.23688	0.33869	0.62369	ac2
NM_001111018	NAV2	0.42031	1.91165	1.39468	0.11830	-0.28508	-0.85067	ac2
NR_000013	SNORD42B	-0.79409	1.90080	0.85342	1.42197	0.49564	0.29886	ac2
NM_015404	DFNB31	1.38554	1.88742	0.55224	0.39882	1.78852	1.81440	ac2
NM_005239	ETS2	1.03592	1.88222	1.02589	0.83426	0.46942	0.47956	ac2
NM_001135638	PIP5K1A	0.90972	1.87588	0.21994	-1.47630	-0.53880	0.91858	ac2
NM_021960	MCL1	1.18361	1.87177	0.97905	0.89886	1.01990	0.78879	ac2
NM_018195	C11orf57	0.24864	1.84508	1.25326	1.59873	1.35997	0.79485	ac2
NR_045962	KRT8	-0.60378	1.82599	-0.63921	0.88953	-0.72998	1.03122	ac2
NM_003954	MAP3K14	0.56457	1.81258	1.75476	0.92382	0.44277	0.48642	ac2
NM_005253	FOSL2	1.08991	1.80005	0.87036	0.47116	1.24241	1.32827	ac2
NM_025195	TRIB1	1.34327	1.79394	1.26004	1.11782	1.71319	1.58715	ac2
NM_000527	LDLR	1.58920	1.79278	1.36251	0.82111	1.34625	1.56787	ac2

NM_003457	ZNF207	1.48783	1.77531	0.44999	0.19874	-0.63556	-0.51131	ac2
NM_002996	CX3CL1	0.91472	1.77198	1.67703	0.75115	0.94110	0.65214	ac2
NM_017632	CDKN2AIP	1.65159	1.76184	0.41036	0.03243	-0.66265	-0.78524	ac2
NM_004809	STOML1	1.09084	1.75826	1.23986	1.33990	0.90753	-0.30829	ac2
NM_001163673	WDR81	1.09331	1.73953	1.25175	0.41163	0.35399	0.84704	ac2
NM_001025579	NDEL1	0.11626	1.73935	1.39928	1.43620	-0.09762	-1.03290	ac2
NM_006403	NEDD9	1.50219	1.73248	0.11241	0.30092	0.82899	0.76893	ac2
NM_001195056	DDIT3	1.49294	1.73150	0.18789	-0.61474	0.47224	0.21418	ac2
NM_201995	SF1	1.53461	1.72246	0.77372	1.36290	0.86245	-0.86571	ac2
NM_004561	OVOL1	0.94588	1.70547	-0.03716	-0.24404	1.08409	1.18854	ac2
NR_028502	MIR22HG	1.24548	1.70509	0.54529	0.32008	0.17237	0.48567	ac2
NM_147187	TNFRSF10B	0.73735	1.69457	1.52479	1.68188	1.48817	1.63655	ac2
NM_199248	CACNB1	0.34161	1.68956	1.46434	0.83983	1.02196	0.70052	ac2
NM_002357	MXD1	0.60643	1.67765	-0.11214	0.86014	1.29466	1.20911	ac2
NM_014806	RUSC2	0.90142	1.66486	0.47917	-0.26469	0.80484	1.29510	ac2
NM_001130677	C17orf96	1.07192	1.66226	0.92499	-0.07122	0.14225	0.17023	ac2
NR_028322	LOC10013228	-0.14064	1.65338	0.70025	0.30681	1.14291	1.54270	ac2
NM_001621	AHR	0.13792	1.64922	1.23851	1.31448	0.84762	0.63009	ac2
NM_001102421	ZFAND5	0.26055	1.63809	-0.06951	0.92388	0.89376	0.68081	ac2
NM_181093	SCYL3	0.13900	1.61534	1.25764	1.26603	0.16622	-0.46414	ac2
NM_002165	ID1	0.79970	1.59224	0.38160	0.45766	0.89003	1.00368	ac2
NR_027160	C17orf76-AS1	1.42574	1.58893	1.03186	0.63015	1.11230	1.18618	ac2
NM_171997	USP2	0.54550	1.58491	1.23393	0.13721	-0.11319	-0.06602	ac2
NM_001142414	CRCP	0.61387	1.58057	0.92782	0.97678	-0.07428	0.32739	ac2
NM_001047160	NET1	0.72675	1.57938	1.54629	1.02535	0.21966	0.13961	ac2
NM_178815	ARL5B	0.61391	1.57056	0.98363	0.71000	0.58364	0.31398	ac2
NM_025201	PLEKHO2	0.64659	1.53019	0.55380	0.23257	1.02352	1.34316	ac2
NM_175742	MAGEA2	0.28018	1.52239	1.33621	1.01538	0.55573	0.67811	ac2
NM_004073	PLK3	0.72678	1.51245	1.42056	0.87347	1.40392	1.47946	ac2
NM_001173977	LRRC16A	0.78881	1.49428	1.08329	0.23395	0.13233	0.80302	ac2
NM_001195802	LDLR	0.96570	1.49415	0.66093	0.11810	-0.42755	0.23023	ac2
NM_177433	MAGED2	1.36445	1.48387	1.14873	0.97327	0.86930	-0.62043	ac2
NM_172016_4	TRIM39	0.34866	1.48340	1.14848	0.47077	-0.22916	-0.35466	ac2
NM_001135636	PIP5K1A	0.65008	1.48168	0.97787	0.58604	0.59677	0.22720	ac2
NR_002912	SNORA67	0.74095	1.47593	0.98804	1.31766	1.01971	-0.19761	ac2
NM_005354	JUND	1.23257	1.47283	0.35259	-0.56746	0.64278	0.88364	ac2
NM_145253	FAM100A	0.87002	1.46176	0.89082	0.36678	0.15676	0.46148	ac2
NM_001199455	BRD2	1.06359	1.45921	0.36876	-0.13852	0.25616	-0.09690	ac2
NM_004235	KLF4	0.97879	1.45510	0.83338	0.35255	0.05247	-0.11288	ac2
NM_148415	ATXN2L	1.10158	1.44722	0.96116	1.02617	1.04354	1.17127	ac2
NM_182739	NDUFB6	-0.88833	1.43495	-0.12940	0.23972	-0.03085	-0.35323	ac2
NR_039969	MIR5047	0.49743	1.43227	1.15788	0.52686	1.14438	0.32626	ac2
NM_001146684	RNF222	1.07675	1.42875	0.75962	0.34149	-0.02602	0.24012	ac2
NM_002908	REL	0.41563	1.42143	-0.09640	-0.02765	0.40110	0.08985	ac2
NM_001100594	SNRK	0.78498	1.41416	0.79666	0.14564	0.21638	0.05229	ac2
NM_133467	CITED4	1.24929	1.40834	1.09474	0.65288	0.39437	0.51348	ac2
NM_014572	LATS2	0.74477	1.40565	0.71594	0.40272	0.33976	0.14267	ac2

NM_005168	RND3	0.74482	1.40481	0.91078	0.58843	0.83244	0.42612	ac2
NR_027712	PIP5K1P1	0.50887	1.38139	0.78240	0.23449	0.33789	0.38234	ac2
NM_025154	SUN1	0.40169	1.37236	0.61047	0.90573	0.54044	-0.37874	ac2
NM_014567	BCAR1	0.10827	1.37137	-0.00408	-0.38842	-0.46850	1.25056	ac2
NM_006734	HIVEP2	0.08898	1.37125	0.56979	0.86469	0.65433	0.43359	ac2
NM_001102420	ZFAND5	0.59841	1.36831	1.27564	0.93844	0.96329	0.84420	ac2
NM_015508	TIPARP	0.33634	1.36172	0.14251	0.28613	0.59459	1.06230	ac2
NR_027782_1	PLEKHM1	1.32338	1.36060	0.87668	1.23487	0.72396	-0.02243	ac2
NM_001165414	LDHA	0.79129	1.35896	0.88919	1.24108	-0.08229	-0.10197	ac2
NR_003035	SNORA16A	0.56551	1.35734	0.49119	0.35881	1.06944	0.65266	ac2
NM_020127	TUFT1	0.65902	1.35653	0.16453	-0.72618	-0.18757	0.21977	ac2
NM_003709	KLF7	0.32875	1.35367	0.56640	1.05372	1.31914	1.14718	ac2
NM_173211	TGIF1	0.44954	1.35315	0.62567	1.23784	0.82129	-0.36487	ac2
NM_032521	PARD6B	0.29903	1.34219	0.54189	0.70185	0.57722	0.71572	ac2
NM_183419	RNF19A	-0.56165	1.30536	0.25336	-0.29513	0.84649	0.11445	ac2
NM_003447	ZNF165	0.61754	1.30147	0.46206	0.06272	0.47265	0.68468	ac2
NM_000623	BDKRB2	0.66417	1.28271	-0.01477	-0.61616	-0.86494	-1.73352	ac2
NM_181726	ANKRD37	1.03633	1.28140	-0.32795	0.19608	0.26286	0.38088	ac2
NM_016605	FAM53C	0.83783	1.27645	0.24640	-0.17688	0.28451	0.56200	ac2
NM_001135101	CRELD2	0.65163	1.27460	0.46929	1.05685	1.13510	0.64213	ac2
NR_002992	SNORA7B	0.36152	1.27360	0.67368	0.64560	1.21838	0.97022	ac2
NM_025090	USP36	0.34337	1.27178	0.60778	0.30993	-0.57539	-0.48653	ac2
NR_002959	SNORA18	0.32729	1.27114	1.16555	0.63867	1.12764	0.73447	ac2
NM_212469	CHKA	0.55504	1.26856	0.72243	-1.23434	0.86893	0.81983	ac2
NM_001033059	AMD1	-0.50248	1.26018	0.83866	-0.53511	-1.31483	-1.19713	ac2
NM_001002914	KCTD11	0.42200	1.25211	1.01757	0.55842	0.62510	1.03555	ac2
NM_001163323	CCDC120	-1.70605	1.24716	1.15756	0.34066	0.65941	0.73773	ac2
NR_002987	SNORA61	0.84597	1.24637	0.40215	0.42348	0.43896	-0.03702	ac2
NM_203417	RCAN1	0.99315	1.24263	-0.91728	-1.79480	0.63943	-1.72497	ac2
NM_001105205	RUSC1	0.46540	1.23756	-0.10543	0.87752	0.11546	0.74946	ac2
NM_001256721	CCT4	-0.24517	1.22875	1.21958	0.76908	0.07005	-0.76429	ac2
NM_001198719	RBBP7	1.00222	1.22398	1.08421	1.01140	-0.00662	-0.19092	ac2
NM_001170687	MIB2	0.63758	1.21577	0.57375	0.49746	0.86431	0.64670	ac2
NM_173828	RELL2	0.58822	1.20464	0.13883	-0.98676	0.97972	0.85241	ac2
NM_003921	BCL10	0.60030	1.20217	0.88056	0.96900	1.15483	1.12987	ac2
NM_001146322	GRAMD3	0.58730	1.19618	-0.05789	0.67878	0.49477	-0.49564	ac2
NM_001144062	COPB1	0.64190	1.17374	-1.30956	0.76673	0.03172	-0.28243	ac2
NR_027762	RBM34	1.02329	1.16130	0.77543	0.71869	1.01361	0.59749	ac2
NM_022374	ATL2	0.58053	1.16125	0.24074	0.22114	0.67629	0.47329	ac2
NM_198220	SNRPB2	0.69765	1.15622	-0.50100	0.66190	-0.45849	-0.86830	ac2
NM_176814	ZNF800	-0.10090	1.15097	0.50958	0.77122	-0.00554	-0.07964	ac2
NM_173074	PIGF	1.13748	1.15045	0.62757	-0.49139	-1.29223	-0.68460	ac2
NM_006494	ERF	0.51519	1.14360	0.10156	-0.55091	0.41110	0.72155	ac2
NM_001253850	VTCN1	0.89761	1.13868	0.71954	0.64481	-2.09958	-1.35468	ac2
NM_001206987	METTL23	0.64858	1.13051	0.57289	0.70605	-0.43854	0.05240	ac2
NM_080829	FAM65C	0.30615	1.12779	0.90387	-0.01631	-0.07920	-0.12673	ac2
NM_018032	LUC7L	0.59167	1.11511	0.70020	0.79087	0.00311	0.25737	ac2

NM_001008727	ZNF121	0.49107	1.10968	0.21486	0.97629	0.18925	0.25467	ac2
NM_033182	FBXO44	1.02743	1.09683	0.65931	0.39311	0.20319	1.08725	ac2
NR_039891	MIR3064	-0.42365	1.09253	-0.21946	-0.74159	0.15478	0.74391	ac2
NM_206836	ECI2	0.74636	1.09144	0.25236	0.96121	0.67432	-0.38417	ac2
NM_018941	CLN8	0.20019	1.08919	0.83234	0.40675	0.14555	0.51084	ac2
NM_031304	DOHH	0.83895	1.08446	1.00295	0.85900	-0.15580	0.81771	ac2
NM_017865	ZNF692	-0.53251	1.08437	-0.77914	-0.92561	0.38718	0.97066	ac2
NR_002580	SNORA3	-0.14684	1.08307	-0.10964	-0.70017	-0.52382	-0.38433	ac2
NR_037985	TTC19	0.25718	1.08164	-0.03354	0.19818	0.71159	-0.40030	ac2
NM_001802	CDR2	0.48490	1.07774	0.73223	0.56599	0.22372	-0.04440	ac2
NM_001167820	BLCAP	0.26715	1.07650	0.15396	0.39693	-0.35827	-0.30454	ac2
NM_003842	TNFRSF10B	0.37890	1.07420	0.76282	0.31836	0.60496	0.84153	ac2
NM_001017369	MSMO1	-0.76983	1.07384	-2.05320	-0.05277	0.66378	-1.27025	ac2
NM_174886	TGIF1	0.86696	1.07156	0.46006	0.61920	0.78140	-0.77991	ac2
NM_001190847	CNOT4	0.05096	1.06774	-0.45753	-0.46934	-0.65011	-0.18163	ac2
NM_177401	MIDN	0.16104	1.06191	0.54102	0.04961	0.43334	0.78865	ac2
NM_001199875	SERF2	0.84411	1.05507	0.48965	0.25018	0.21148	0.53685	ac2
NM_015483	KBTBD2	0.25413	1.05205	0.42080	0.40759	0.54511	0.24487	ac2
NM_182556	SLC25A45	0.86287	1.05105	-0.87362	0.69835	-0.03550	1.02270	ac2
NM_022913	GPBP1	-0.01343	1.05088	0.85100	0.32214	0.71909	0.21227	ac2
NM_001093756	C5orf44	0.79691	1.04717	0.08898	0.31483	0.71154	-0.22926	ac2
NM_001001522	TAGLN	0.41612	1.04703	0.54100	0.15740	0.72281	-0.38398	ac2
NR_036542	CARS	-0.38598	1.04320	-0.84512	0.53135	-0.07860	0.40866	ac2
NM_153210	USP43	0.54371	1.02865	-0.09714	-0.18403	-0.02330	-0.05996	ac2
NM_006145	DNAJB1	0.78821	1.02589	0.01004	-0.33326	0.64855	0.81918	ac2
NM_005920	MEF2D	0.80463	1.02286	-0.79901	-0.90447	0.48172	0.73064	ac2
NM_001162895	KIAA0040	-0.23046	1.01937	-0.32609	-1.16647	-1.80115	0.46682	ac2
NR_039983	LOC729737	-0.27140	1.01577	0.47899	-0.27583	0.44739	0.87759	ac2
NM_001077665	AGAP6	0.03970	1.00280	0.15338	0.49536	-0.03127	-0.06891	ac2
NM_001146688	KDM3A	0.47937	1.00245	0.57306	0.70745	0.78410	0.41584	ac2
NM_005966	NAB1	-0.14352	1.00118	0.97302	0.84607	0.32236	0.21548	ac2
NR_028326	LOC10013316	-0.24484	1.00051	0.52714	-0.00188	0.29516	0.82633	ac2
NM_213622	STAMBP	-0.53618	1.00010	0.59053	0.38819	-0.21352	-0.06081	ac2
NM_002982	CCL2	1.86819	3.01789	3.99958	3.51487	2.02618	1.74864	ac3
NM_021101	CLDN1	0.87072	2.72065	3.60777	3.36769	1.67663	1.37433	ac3
NM_001124	ADM	2.35693	2.45085	3.54236	3.08884	1.75886	2.24432	ac3
NM_001945	HBEGF	0.97461	2.94775	3.35873	2.29367	1.28263	1.23177	ac3
NM_001220778	CDKN1A	-0.49513	1.85975	3.21857	2.66877	1.87218	3.01886	ac3
NR_003078	SNORD110	1.78746	1.74851	3.17682	2.06322	0.74363	2.15524	ac3
NM_032865	TNS4	1.02804	2.51097	3.12687	2.42480	1.78485	1.80313	ac3
NM_001220777	CDKN1A	1.15278	2.49115	3.11536	2.38055	2.67450	2.53998	ac3
NM_006290	TNFAIP3	2.89335	2.43170	3.09172	2.79384	2.00462	1.21781	ac3
NM_000389	CDKN1A	0.82764	1.93327	3.03939	2.08739	1.74249	2.76403	ac3
NM_172087	TNFSF13	2.64977	0.71690	2.80098	2.26396	0.52533	1.05896	ac3
NM_020317	C1orf63	0.75863	2.03818	2.79558	1.94282	1.44664	1.93900	ac3
NM_024616	C3orf52	0.72159	2.36179	2.71855	2.60343	1.13495	1.12982	ac3
NM_032641	SPSB2	1.94541	0.82399	2.68104	1.49577	1.34123	2.26493	ac3

NM_007350	PHLDA1	0.64979	2.34166	2.65276	2.08113	2.40187	2.44784	ac3
NM_003899	ARHGEF7	0.98553	2.56315	2.62055	2.56272	0.15533	2.60869	ac3
NM_182966	NEDD9	1.24815	1.81864	2.53800	2.33589	0.05243	0.49147	ac3
NM_006465	ARID3B	0.24146	1.91348	2.37544	2.13939	1.39762	1.55584	ac3
NM_001122607	RUNX1	1.54852	2.13924	2.37275	2.30028	1.28337	1.14842	ac3
NM_138959	VANGL1	0.83588	0.27076	2.33617	1.97455	0.63398	0.56471	ac3
NM_016655	GABPB1	1.06152	1.58236	2.23306	1.30201	1.96909	1.34152	ac3
NM_002608	PDGFB	0.23348	0.64443	2.16655	1.80156	1.66059	1.92889	ac3
NM_001253815	GPR172A	-0.93305	1.47713	2.16128	0.70637	1.25091	1.08129	ac3
NR_046368	HP07349	1.00896	1.01978	2.14994	0.72713	1.60973	0.92812	ac3
NM_005902	SMAD3	-0.09305	0.68192	2.12870	1.69740	1.49330	1.52990	ac3
NM_145041	TMEM106A	0.30943	1.50309	2.11887	1.50679	0.53550	0.59181	ac3
NM_014395	DAPP1	-0.18698	0.66842	2.09292	1.78211	1.01480	1.20792	ac3
NM_007372	DDX42	0.77773	0.56950	2.07327	1.77894	0.65593	0.61339	ac3
NR_028436	UBE2L3	2.03819	0.63715	2.05834	1.71680	0.26891	1.14820	ac3
NM_080796	DIDO1	1.25399	1.68126	2.04641	1.80277	1.92635	1.56024	ac3
NM_133640	MED22	1.35177	-0.52731	2.03918	1.61743	1.15970	-0.07592	ac3
NM_001168388	CITED2	0.40696	1.41636	2.00578	1.98487	-0.04533	-0.76331	ac3
NM_001099285	PTMA	0.61201	-2.80123	2.00240	-0.57242	0.13450	1.57311	ac3
NM_138373	MYADM	1.00038	1.49605	1.99796	1.45167	0.16072	0.39415	ac3
NM_201555	FHL2	-0.14866	0.77449	1.96674	1.86568	1.15620	1.45448	ac3
NM_012099	CD3EAP	1.15019	1.33502	1.95242	1.94275	-0.10392	0.07487	ac3
NM_001136004	MICAL3	0.42914	0.76956	1.94340	1.70379	0.02307	-0.44850	ac3
NM_007318	PSEN1	0.00310	1.45720	1.93017	1.78868	1.28153	1.37990	ac3
NM_001421	ELF4	-0.32009	0.30531	1.90462	1.24259	0.40745	1.36298	ac3
NM_003378	VGF	0.71904	1.56613	1.89909	1.28838	0.69758	1.41197	ac3
NM_145725	TRAF3	1.24320	0.78838	1.87549	1.61471	1.12204	0.34784	ac3
NM_033657	DAP3	1.60346	1.32468	1.87487	1.44161	0.49729	1.19851	ac3
NM_152222	RELT	0.39348	1.67137	1.87074	1.23842	0.34645	0.74807	ac3
NM_001079519	FAM177A1	0.76695	0.99733	1.86698	1.56352	1.78835	1.56380	ac3
NM_153690	FAM43A	0.28081	0.58403	1.85464	1.58166	1.02491	1.60065	ac3
NM_002999	SDC4	0.25667	1.32292	1.85121	1.55792	0.92513	1.00040	ac3
NM_182480	COQ6	1.15999	1.41054	1.84592	-0.38048	0.42045	0.25880	ac3
NM_003981	PRC1	1.47579	1.52829	1.82389	1.64673	1.11856	0.71590	ac3
NM_022767	AEN	0.56205	1.60294	1.81494	1.35103	0.50185	0.57981	ac3
NM_206890	DIP2A	1.46699	0.62320	1.81193	0.78094	1.19566	1.13776	ac3
NM_001256540	NOP16	1.20930	0.40942	1.80784	1.60637	0.62845	-0.06162	ac3
NM_003844	TNFRSF10A	0.51870	1.40171	1.80173	1.60270	0.34581	0.35364	ac3
NM_001134338	RNF24	-0.64163	0.77521	1.79642	1.05603	-0.36587	-0.63049	ac3
NM_023940	RASL11B	-0.01262	0.33927	1.74417	0.88975	-0.75676	-1.00842	ac3
NM_001206696	IRF6	0.30096	0.77653	1.71912	1.15110	0.12183	0.71406	ac3
NM_033016	PDGFB	0.27448	0.40589	1.69582	1.45039	1.06824	1.20878	ac3
NM_014344	FJX1	-0.18394	0.67645	1.69419	1.58840	-0.38902	-0.91368	ac3
NR_003189	SYS1-DBNDD2	1.12607	1.02703	1.68682	1.17831	-0.11318	0.76928	ac3
NM_001080470	ZNF697	0.04099	0.60546	1.68355	1.63160	0.45387	0.05179	ac3
NM_015641	TES	0.38435	0.72607	1.66572	1.52424	1.22262	1.03038	ac3
NM_000611	CD59	1.27929	0.89577	1.64365	0.69181	1.33486	1.06374	ac3

NM_001013842	C8orf58	0.54146	1.19320	1.64348	1.31037	1.60583	0.80884	ac3
NM_001145301	RARA	0.02721	0.43937	1.64170	1.15081	0.90426	1.20520	ac3
NR_002976	SNORA44	1.36700	0.96805	1.61794	1.32982	-2.08258	0.18722	ac3
NM_001080125	CASP8	1.01011	0.33153	1.60188	1.44039	1.00020	0.91470	ac3
NM_001204410	SEC14L1	1.11212	0.94241	1.59042	1.08377	-0.96130	-0.08462	ac3
NM_001143843	TMEM106C	0.42233	-0.89884	1.58301	1.07881	-0.63928	-1.10376	ac3
NM_003740	KCNK5	0.13929	0.80732	1.58220	0.90935	0.22919	0.02391	ac3
NM_032482	DOT1L	0.23463	0.78552	1.57809	1.11839	0.51485	0.63040	ac3
NM_022763	FNDC3B	0.32124	-0.41334	1.56882	1.34478	0.38450	1.05932	ac3
NM_020148	SPIRE1	0.00259	1.00982	1.56301	1.55191	0.14673	-0.20998	ac3
NM_023938	C1orf116	0.30285	0.71070	1.56013	1.40425	1.27080	1.55306	ac3
NM_021724	NR1D1	-0.23698	0.60308	1.51754	0.69329	0.63753	0.36526	ac3
NM_018387	STRBP	1.44213	1.02816	1.51682	0.66087	1.00611	1.22717	ac3
NM_001204193	SRP19	-0.40739	0.41100	1.50396	1.29542	0.66043	0.62553	ac3
NM_001924	GADD45A	-0.61562	0.77026	1.50136	1.24711	0.20585	0.26403	ac3
NM_001242339	PFKP	0.86772	1.38777	1.43640	0.70879	-0.26283	0.34375	ac3
NM_004936	CDKN2B	0.30847	1.20261	1.41871	1.33605	0.79870	0.74085	ac3
NM_003376	VEGFA	0.79425	1.36777	1.41508	0.87011	-0.08361	0.78748	ac3
NM_001083113	ZGPAT	-2.07696	0.35716	1.41493	1.10066	-0.02954	-1.29505	ac3
NM_003088	FSCN1	0.45270	1.04626	1.40311	1.00243	0.92505	1.03588	ac3
NM_173344	ST3GAL1	0.17646	0.44330	1.39535	0.88225	0.49549	0.46784	ac3
NM_014550	CARD10	-0.06491	0.43438	1.39387	1.14287	0.91577	1.25526	ac3
NM_001134758	ARFRP1	0.35385	0.84287	1.39355	0.38877	-1.11745	0.49577	ac3
NM_001142588	NFYC	1.29932	0.73799	1.38440	-0.11067	0.73135	0.69137	ac3
NM_001099753	SYBU	0.91717	0.60326	1.38116	0.48246	-0.03869	0.74717	ac3
NM_001242481	EIF1AD	-1.74290	-0.88743	1.37545	-0.60734	0.45080	0.06404	ac3
NM_001202233	NR4A1	-0.10341	1.03597	1.37459	0.76309	0.70496	0.79065	ac3
NM_012101	TRIM29	0.26465	0.81874	1.36890	1.21953	1.20455	1.27992	ac3
NM_004636	SEMA3B	0.10607	-0.61099	1.36436	0.17872	0.31422	0.14595	ac3
NM_001164313	ZBTB24	0.62736	0.93556	1.36337	1.00059	0.06467	-0.63945	ac3
NM_001199694	SLC4A2	0.14884	0.14467	1.33582	0.15737	0.42217	1.12162	ac3
NR_024052_6	HCG18	0.68590	-0.06678	1.32919	0.99586	0.92148	1.05342	ac3
NM_001165412	NFKB1	-0.51265	0.85651	1.32736	1.22064	-0.08092	-1.40911	ac3
NM_006749	SLC20A2	0.00266	0.74517	1.32656	1.00220	-0.07670	-0.00303	ac3
NM_004083	DDIT3	1.22897	1.05660	1.32651	0.49218	0.69022	0.38216	ac3
NM_006047	RBM12	-0.50000	0.62436	1.32313	0.56015	0.26683	0.40657	ac3
NM_001127231	AUTS2	0.52768	-0.29231	1.32111	0.84072	0.62074	-0.18989	ac3
NR_000007	SNORD73A	-0.67456	-1.45599	1.31731	-0.50013	-1.28019	0.77902	ac3
NM_002473	MYH9	0.05162	0.84471	1.30705	1.05579	0.35210	0.39456	ac3
NM_007026	DUSP14	0.16631	0.80641	1.30287	0.94256	0.48239	0.72354	ac3
NM_015000	STK38L	0.24386	1.20426	1.30206	0.87937	-0.01247	-0.06098	ac3
NM_001039481	ETNK1	0.00060	0.76034	1.29372	0.92263	0.48186	0.55893	ac3
NM_000346	SOX9	0.78968	0.82928	1.28324	1.03670	1.08247	0.90778	ac3
NM_000161	GCH1	0.23464	0.97330	1.27013	1.15593	0.23900	-0.49498	ac3
NM_001002814	RAB11FIP1	0.02624	0.43758	1.26312	1.06049	1.18187	0.89522	ac3
NM_001080450	BEND3	-0.07443	0.45921	1.26235	1.22042	-1.20587	-1.68682	ac3
NM_001197114	IQCJ-SCHIP1	0.07839	0.98615	1.25087	1.07507	0.81511	0.41716	ac3

NM_001020658	PUM1	0.52505	-0.33581	1.24246	0.92230	0.98618	0.65533	ac3
NM_012296	GAB2	0.07870	-0.14808	1.23815	0.15223	0.76929	0.71435	ac3
NM_006832	FERMT2	0.12970	1.10265	1.23713	1.00860	0.63166	0.27915	ac3
NM_007242	DDX19B	1.02184	0.87037	1.23630	0.77223	0.49584	0.56691	ac3
NM_001160305	SETD6	0.38165	0.39343	1.23227	0.95325	0.38447	0.44056	ac3
NM_021630	PDLIM2	0.16450	0.30805	1.22042	1.00922	0.73257	1.20460	ac3
NM_001195302	C16orf57	0.67431	0.55228	1.21728	1.12460	0.83479	1.02236	ac3
NM_001130921	RABL2B	0.76159	0.35881	1.21485	0.21255	-0.24841	-0.80309	ac3
NM_004756	NUMBL	0.20302	0.50315	1.20864	0.55455	0.78008	1.19390	ac3
NM_001135023	ELMOD3	-0.34169	-0.48448	1.19325	0.90504	-1.35546	-0.05171	ac3
NR_015441	LOC652276	0.18550	0.79479	1.18907	1.13767	0.34775	0.42644	ac3
NM_001204137	MBD1	-0.43796	0.42682	1.17334	-0.41665	0.37980	-0.42314	ac3
NM_001008801	ZNF468	0.07144	0.64536	1.17196	0.47038	-0.32166	0.37115	ac3
NM_001100119	XRCC3	1.14723	-1.31368	1.17190	-0.88722	0.38451	-0.39686	ac3
NM_153341	RNF19B	0.48770	0.71246	1.16866	0.75582	0.59645	0.90330	ac3
NM_032998	DEDD	0.78405	0.80604	1.16527	1.02423	0.91639	0.71161	ac3
NM_006079	CITED2	0.32953	0.12245	1.16337	0.81169	-0.00736	0.34044	ac3
NM_005911	MAT2A	0.53861	1.04869	1.16276	0.73134	0.21669	0.13371	ac3
NM_001142391	SLC10A3	-0.01465	0.15689	1.16269	1.08247	-0.64161	-0.31883	ac3
NM_022658	HOXC8	-0.02928	0.25742	1.15872	1.13668	-0.15125	-0.38551	ac3
NM_001199119_	TRIM39-RPP2	1.07407	0.89937	1.15793	1.09270	0.37676	0.25743	ac3
NM_177455	BHLHA15	0.38977	-0.08708	1.14897	0.63698	0.50748	1.14773	ac3
NM_030803	ATG16L1	-0.03903	-1.18365	1.14309	0.70076	-0.20928	-1.01270	ac3
NM_001077494	NFKB2	0.54352	0.99390	1.13807	0.49449	0.47791	1.11928	ac3
NM_030927	TSPAN14	0.06301	0.44226	1.13782	1.07386	0.67081	0.79532	ac3
NM_000418	IL4R	-0.14672	0.08151	1.13250	1.07345	0.69613	0.92987	ac3
NM_052899	GPRIN1	0.06176	0.53179	1.13206	0.49090	0.80629	1.10182	ac3
NM_170713	RASSF1	0.03600	0.42011	1.12913	0.76801	-0.06591	0.52095	ac3
NR_037428	MIR3655	-0.27623	0.11914	1.12729	-0.06227	-0.13090	0.62538	ac3
NM_004531	MOCS2	0.08860	-0.49382	1.12625	0.81086	0.83293	0.86046	ac3
NM_001201339	SAFB	0.58817	0.78743	1.12304	0.83871	0.76393	0.13172	ac3
NM_003998	NFKB1	0.39955	0.49696	1.11637	1.03100	-0.23622	-0.20434	ac3
NM_001134422	CDV3	0.32628	0.50189	1.11482	0.79373	0.75339	1.00511	ac3
NM_005415	SLC20A1	0.38922	0.94039	1.10418	0.79233	-0.19279	-0.49126	ac3
NM_006887	ZFP36L2	-0.02530	0.67595	1.10388	0.85996	0.47582	0.24365	ac3
NM_012063	DNM1L	0.53377	0.13885	1.10355	0.22332	-0.86898	0.07032	ac3
NM_001163509	DCAF4	0.79480	0.67738	1.09744	0.79479	-0.57135	0.06329	ac3
NR_027247	LOC10013033	0.53471	0.69347	1.09449	1.08449	0.82328	0.98566	ac3
NM_152398	OCIAD2	-0.14517	0.31846	1.09226	0.98518	0.65691	0.74626	ac3
NM_001005328	OR2A7	0.17248	-0.24685	1.09212	-0.03236	0.44172	0.61441	ac3
NM_178582	HM13	0.18120	-0.06532	1.09145	0.98264	0.34782	-0.00527	ac3
NM_004760	STK17A	-0.14110	0.80143	1.08751	1.01735	0.96509	0.86936	ac3
NM_152271	LONRF1	0.51955	0.84623	1.08572	0.94626	0.98609	0.69435	ac3
NM_001032381	PQBP1	0.19153	0.28459	1.08562	-0.36288	0.02700	0.76097	ac3
NM_018455	CENPN	1.03122	0.75860	1.08154	0.85079	-0.29044	0.21188	ac3
NM_001001479	SLC35E4	0.28760	0.32988	1.07999	0.73495	0.60938	0.85976	ac3
NM_001014837	CUTA	0.56988	0.23254	1.07961	0.68600	0.65161	0.27752	ac3

NR_037675	PRR24	0.87701	0.94111	1.06832	0.61110	0.82623	0.44525	ac3
NM_001145443	PFKFB3	-0.03723	0.67975	1.06811	1.05410	0.29554	0.80974	ac3
NR_001457	SNORD38B	1.06214	0.51035	1.06297	0.55131	0.65673	0.29862	ac3
NM_001195446	SRSF7	-0.55288	0.49255	1.06151	0.95628	0.36847	-0.04990	ac3
NM_001144944	MYL12B	0.03291	-0.73160	1.05860	0.85270	0.89637	-0.90151	ac3
NM_001111077	EZR	0.31592	0.70691	1.05492	0.68240	0.10320	0.15704	ac3
NM_001130688	HMGB2	-0.13443	-0.35209	1.05461	0.14151	0.19534	-0.46333	ac3
NM_203472	SELS	-0.31659	0.27037	1.05346	-0.04343	-0.65293	-0.61594	ac3
NM_201997	SF1	0.60994	0.62633	1.05020	0.08455	-0.45535	-0.05075	ac3
NM_198490	RAB43	-1.14582	-0.05815	1.03809	0.10577	0.90369	0.89331	ac3
NR_037654	MTHFS	0.30590	0.51096	1.03687	0.82329	0.28370	0.15661	ac3
NM_002717	PPP2R2A	0.07952	0.30238	1.03505	0.87405	0.65958	0.62394	ac3
NM_015530	GORASP2	0.17489	0.51527	1.03136	0.72070	0.80353	0.93683	ac3
NM_005088_1	AKAP17A	0.60146	1.01817	1.03101	-0.50626	0.55398	0.35276	ac3
NM_005336	HDLBP	0.92853	-0.11562	1.02944	0.84941	0.40634	0.27746	ac3
NM_001136258	SGMS2	0.39580	0.88729	1.02815	0.29848	0.31740	-0.00724	ac3
NM_001127204	HMOX2	0.42641	0.23583	1.02549	0.59663	0.52180	0.42836	ac3
NM_139279	MCFD2	0.51031	0.75828	1.02371	0.10340	0.17887	0.23302	ac3
NR_024438	LOC648740	0.50817	0.71853	1.01705	0.85388	0.67071	0.92003	ac3
NM_020762	SRGAP1	-0.05055	0.17989	1.01557	0.34852	0.44164	0.26816	ac3
NM_004730	ETF1	-0.44919	0.71845	1.01449	0.66890	-0.77497	-0.02310	ac3
NM_001614	ACTG1	0.41017	0.61996	1.01440	0.92886	0.52490	0.78593	ac3
NM_006253	PRKAB1	0.18257	0.60830	1.00703	0.78880	0.37469	0.61419	ac3
NM_139239	NFKBID	0.48398	0.96084	1.00600	0.28172	0.25388	0.46774	ac3
NM_004815	ARHGAP29	-0.40587	0.35717	1.00440	0.89275	0.51671	0.44524	ac3
NM_014405	CACNG4	-0.00716	-0.03376	1.00370	0.75659	0.71011	0.83120	ac3
NM_004281	BAG3	0.13838	0.60104	1.00108	0.61558	0.78473	0.99737	ac3
NM_012424	RPS6KC1	0.41130	-0.11819	1.00103	0.37821	0.88075	0.93470	ac3
NR_046330	TUBGCP2	0.27287	-0.05537	1.00027	0.99290	0.80508	0.44455	ac3
NM_006528	TFPI2	2.98699	5.06204	5.83256	6.18758	5.66790	5.34493	ac4
NM_000598	IGFBP3	0.58085	1.87057	3.84735	4.44213	3.94812	3.55967	ac4
NM_001142601	SPHK1	1.50176	1.12849	3.75766	4.34123	3.64768	3.66233	ac4
NR_036260_1	MIR1184-3	2.17789	-0.17386	1.50360	3.98547	-1.24868	0.46433	ac4
NM_001204860	TCEB1	2.79128	0.74621	2.32448	3.84427	1.87965	1.45675	ac4
NR_030345	MIR614	1.35867	2.97249	3.04157	3.25558	2.58612	2.10363	ac4
NM_015515	KRT23	0.39304	1.75648	2.97644	3.19266	2.82824	2.62441	ac4
NM_001123378	ADSL	2.73343	0.64235	1.41233	3.18327	1.25149	-0.30882	ac4
NM_001242482	EIF1AD	2.15847	2.85331	2.29883	3.02646	2.72441	2.45701	ac4
NM_134324	TARBP2	1.83150	-0.38762	1.92808	2.84214	2.35030	1.02801	ac4
NR_024204	LINC00152	0.23166	1.01034	2.38820	2.83517	1.44610	1.57572	ac4
NM_000565	IL6R	0.33993	0.66190	2.30022	2.69782	1.63006	1.01889	ac4
NM_001199784	SDCBP2	-0.42212	0.33253	1.96520	2.68562	2.19778	2.63709	ac4
NM_005243	EWSR1	1.01668	0.76041	1.21133	2.68353	-1.29042	-0.71356	ac4
NM_001039492	FHL2	0.49214	1.38031	2.43456	2.67511	2.15003	2.40073	ac4
NM_001201370	RNF7	1.05320	-0.58139	-0.17900	2.66071	1.04280	1.30710	ac4
NM_144495	PQBP1	1.28023	0.16603	1.62658	2.63341	1.01687	0.43566	ac4
NM_001199697_	BAG6	0.48922	0.66873	0.77863	2.63230	1.81729	1.61457	ac4

NM_001013442	EPGN	0.70411	2.25858	1.98189	2.59196	2.16448	1.58125	ac4
NM_013390	TMEM2	-0.34390	1.32865	1.96389	2.57723	1.23562	1.20089	ac4
NM_001199873	B4GALT3	2.29943	1.59985	2.00087	2.55713	1.79457	1.77949	ac4
NR_033192	CCDC59	1.11531	2.50062	2.26389	2.51061	0.62775	0.38221	ac4
NM_001145160	TPM4	0.85485	1.43301	2.02301	2.50932	0.67059	0.06288	ac4
NM_001130850	CAB39	-1.88896	0.80276	2.08118	2.48579	0.36212	-1.22369	ac4
NM_004998	MYO1E	0.20539	0.69652	1.93001	2.42255	1.35271	1.05771	ac4
NM_005242	F2RL1	0.29045	1.17421	2.19412	2.40083	1.29170	1.21263	ac4
NM_014488	RAB30	0.91663	2.16305	2.08694	2.39923	0.11652	-0.08065	ac4
NM_198256	E2F6	0.39402	0.13920	1.56320	2.37073	-0.00602	0.59522	ac4
NR_033675	VDAC2	1.75037	2.12880	0.06522	2.36355	-2.08249	1.12405	ac4
NM_001008897	TCP1	0.90867	1.66560	1.03564	2.36334	-0.21388	-0.03326	ac4
NM_001174120	ZFYVE27	0.66572	0.12301	2.34163	2.34295	1.01369	1.88467	ac4
NM_001003931	PARP3	0.52122	2.16316	1.84038	2.33885	1.28968	0.75736	ac4
NM_025179	PLXNA2	-0.26962	0.43264	2.13162	2.31714	1.41798	1.56336	ac4
NM_001253875	UXS1	-0.53420	0.55311	2.00020	2.31476	1.05742	-0.24079	ac4
NM_001993	F3	0.73596	1.89062	1.76670	2.31462	0.68288	0.45102	ac4
NM_001198806	C9orf30	1.32333	0.75146	1.97701	2.28229	0.03517	1.05736	ac4
NM_201517	H2AFV	1.76321	1.54108	1.17188	2.27107	1.13742	1.00328	ac4
NM_001173534	DGCR2	1.87384	-1.11330	1.21457	2.26355	-0.17215	0.56602	ac4
NM_003125	SPRR1B	0.32192	1.07494	1.72438	2.26271	1.93767	1.94226	ac4
NM_001251979	RCAN3	0.88329	0.99794	1.50364	2.18014	0.55151	1.37082	ac4
NM_198948	NUDT1	0.79274	1.22113	0.59401	2.17697	-0.46380	1.26329	ac4
NM_130830	LRRC15	0.23880	0.55003	1.40752	2.13719	0.45665	1.30027	ac4
NM_001195630	MLLT10	1.97296	-0.61041	1.19461	2.11482	1.38538	0.41819	ac4
NM_012081	ELL2	0.12690	1.07773	1.52398	2.10757	2.10139	1.86123	ac4
NM_014325	CORO1C	0.28899	0.80452	1.67351	2.10668	0.81293	0.37489	ac4
NM_198435	AURKA	1.98783	0.80411	0.14117	2.09694	0.57484	1.53805	ac4
NM_003379	EZR	1.20082	2.01229	2.04260	2.08734	1.88894	1.52813	ac4
NM_001034024	DMAP1	1.27338	1.85201	1.45388	2.03507	1.47302	1.23708	ac4
NR_000020	SNORD33	1.67530	0.00926	1.47474	2.02819	0.59002	0.67026	ac4
NM_152523	CCNYL1	0.52865	0.94835	1.66666	2.00548	0.66019	0.28857	ac4
NM_001242915	ZFAND6	-0.64371	0.40150	0.05775	2.00308	0.50196	0.10766	ac4
NM_003051	SLC16A1	0.21833	0.21300	0.89623	2.00183	0.59525	0.55072	ac4
NM_015412	C3orf17	1.15193	1.56958	0.94313	1.98030	0.96620	0.71378	ac4
NM_005139	ANXA3	0.37974	0.57613	1.64838	1.97538	1.85696	1.74415	ac4
NM_001039671	YIF1B	1.17677	-1.98747	-1.56696	1.97226	0.92846	-0.34552	ac4
NM_001135811	FAM60A	0.24737	1.06493	1.44353	1.95297	0.78379	0.16235	ac4
NM_018393	TCP11L1	1.27004	1.05134	1.18611	1.94442	1.46718	0.28932	ac4
NM_016476	ANAPC11	0.73904	0.90907	1.05456	1.93155	0.66741	1.88819	ac4
NM_199001	C9orf169	-0.62174	-0.05613	1.19732	1.90808	0.22518	0.92881	ac4
NM_001037872	REV1	1.66704	1.55637	0.98251	1.88578	1.44097	1.36554	ac4
NR_038830	PIN1	0.81438	0.92195	0.05933	1.88543	0.07162	-1.07185	ac4
NM_006033	LIPG	0.27195	0.64381	1.49309	1.87276	1.60929	1.63876	ac4
NM_001100423	SPATS2L	1.08095	0.96899	1.50837	1.86876	1.26239	1.25296	ac4
NM_001202547	SYNJ2BP-COX	1.58624	0.33726	0.73660	1.86843	0.58517	0.83115	ac4
NM_021238	FAM60A	1.16921	0.39376	0.65406	1.86703	-0.82777	-0.19385	ac4

NM_001159287	TPI1	-0.14060	1.18073	-0.03005	1.86077	-0.07646	-0.03420	ac4
NR_045724	CLTB	0.58696	1.56848	1.58035	1.83960	0.56057	0.94105	ac4
NM_024518	ULBP3	0.60729	0.87404	1.68189	1.83853	0.11261	0.95096	ac4
NM_133463	AMZ1	0.08830	0.42364	1.69726	1.83824	0.53067	0.33725	ac4
NM_005953	MT2A	0.62670	0.76723	1.32045	1.83244	1.70520	1.61656	ac4
NM_030922	NIPA2	1.41864	1.49750	1.31430	1.81316	1.27711	0.63517	ac4
NM_001153552	SCOC	1.63899	1.26122	1.37658	1.81000	0.46150	0.17677	ac4
NM_203350	ZRANB2	1.60099	1.31237	1.65250	1.80672	0.69318	0.99552	ac4
NM_145693	LPIN1	-0.18916	0.34677	1.30353	1.79721	0.56449	0.45289	ac4
NM_194283	DNAJC21	0.81295	0.03452	0.39117	1.79477	0.31730	0.34558	ac4
NM_016639	TNFRSF12A	0.21906	0.90738	1.76254	1.78836	1.34636	1.58981	ac4
NM_182762	MACC1	-0.07984	1.56630	1.54642	1.78143	0.55688	0.47718	ac4
NM_001779	CD58	0.65153	0.96650	1.12405	1.75754	1.13764	0.49013	ac4
NR_030318	MIR589	-0.80293	1.00613	0.58156	1.75687	-0.51319	-0.15113	ac4
NM_170662	CBLB	0.23252	0.18878	1.33005	1.74922	1.60339	1.37963	ac4
NM_006457	PDLIM5	0.09296	0.32756	1.22024	1.74660	1.07355	0.89775	ac4
NM_024586	OSBPL9	0.18096	0.20512	1.25613	1.74316	0.59693	1.05486	ac4
NM_001030006	AP2B1	0.51393	0.88971	0.08322	1.72718	-0.27136	1.02594	ac4
NM_021643	TRIB2	-0.02836	0.13955	1.19551	1.72706	1.11343	1.31407	ac4
NM_000676	ADORA2B	0.19533	0.24607	1.62863	1.72265	0.96152	0.89284	ac4
NM_172177	MRPL42	-0.15348	-0.46275	0.57635	1.70442	0.20256	0.30010	ac4
NR_024206	LINC00152	0.13172	0.44631	1.47078	1.70396	0.64246	0.88205	ac4
NM_002897	RBMS1	0.09249	0.27874	0.99118	1.69893	0.48594	0.40853	ac4
NM_001113496	42987	0.15895	0.75729	1.42268	1.68091	-1.41932	-0.58694	ac4
NR_037458	MIR3687	0.52588	1.41095	1.29018	1.67878	-0.76935	1.30287	ac4
NM_004507	HUS1	0.39415	-0.21567	0.89568	1.67461	0.85498	0.59649	ac4
NM_001256403	SLMO2	0.68448	1.20330	0.92454	1.67034	-0.32410	0.77164	ac4
NM_001038493	DLX1	0.59272	1.30956	0.91345	1.66938	1.12292	0.39410	ac4
NM_016224	SNX9	0.03438	0.37937	1.38800	1.66874	1.29268	0.93212	ac4
NM_001184718	TIPARP	1.24354	1.44517	1.40901	1.66858	1.00923	0.60288	ac4
NM_001005242	PKP2	0.11866	0.22479	1.10162	1.66113	0.74886	0.42079	ac4
NR_024373	LOC541471	0.04876	0.24376	1.21281	1.65566	1.04027	1.22806	ac4
NM_152386	SGPP2	-0.47762	-0.21942	1.38388	1.65466	1.21927	0.39787	ac4
NM_001079537	TRAPPC6B	1.04729	1.53213	0.89271	1.65447	1.41873	1.15361	ac4
NM_007200	AKAP13	0.33450	0.38779	1.55249	1.64301	1.25878	0.65145	ac4
NM_004093	EFNB2	0.49304	1.18608	1.23236	1.62936	-0.03398	-0.18330	ac4
NM_002940	ABCE1	0.82632	0.69828	0.65598	1.61737	-0.31494	-0.40920	ac4
NM_014787	DNAJC6	0.34761	0.26809	1.02305	1.60900	0.22375	-0.56794	ac4
NM_177423	PPFIA1	-0.08127	0.21283	1.12150	1.60587	1.32197	0.70505	ac4
NM_001198681	LEPROT	0.70217	0.35439	0.69287	1.59593	1.15364	0.24499	ac4
NM_012154	EIF2C2	0.22237	0.78027	1.41103	1.59570	1.12248	1.13895	ac4
NM_172373	ELF1	1.35263	1.37408	0.87265	1.59416	1.27237	1.12843	ac4
NM_014220	TM4SF1	0.39136	0.78462	1.27689	1.56525	1.09110	1.07308	ac4
NM_001135768	PVR	0.73975	0.79223	0.98836	1.56301	0.38384	0.62504	ac4
NM_005547	IVL	0.16000	0.74151	1.46674	1.55638	0.96079	1.18238	ac4
NM_001166269	HAUS4	1.39012	1.01154	0.97794	1.55409	1.30372	0.43345	ac4
NM_001025237	TSPAN4	-1.39609	0.91334	-0.25788	1.54803	-0.86030	1.32076	ac4

NM_182931	MLL5	0.86303	0.70679	0.46781	1.53553	0.86881	0.51962	ac4
NM_016289	CAB39	-0.14434	0.59129	0.99965	1.53215	1.10225	1.03018	ac4
NM_014584	ERO1L	-0.12686	0.45267	0.97222	1.52721	1.21804	1.21491	ac4
NM_175075	C8orf42	0.35599	0.56445	0.32365	1.51943	0.50070	0.06665	ac4
NM_003174	SVIL	0.15250	0.42420	0.70491	1.51278	1.05084	0.80839	ac4
NM_001256183	ANKRD11	1.26750	1.12590	1.43606	1.50492	-0.67820	1.46118	ac4
NM_147188	FBXO22	0.99136	0.32276	0.07034	1.50218	0.33112	-1.30935	ac4
NM_005737	ARL4C	0.14193	0.72288	1.34382	1.50047	-0.19987	-0.49560	ac4
NM_001243754	C7orf49	0.96816	1.46870	1.47498	1.49856	0.31492	0.84559	ac4
NM_001256532	MSTO1	0.17743	0.18493	0.40541	1.49841	-2.21711	0.56599	ac4
NM_031453	FAM107B	-0.07432	0.33013	1.02292	1.49831	1.02590	0.56688	ac4
NM_015472	WWTR1	0.57938	0.56352	0.27887	1.49640	1.32962	0.99173	ac4
NM_152330	FRMD6	-0.02538	0.17862	1.05542	1.49440	0.65450	0.47492	ac4
NM_001036645	HHLA3	1.10755	0.23642	0.09724	1.48998	0.31006	0.58917	ac4
NM_018482	ASAP1	0.13995	0.13177	0.96429	1.48820	0.56532	0.18393	ac4
NM_178336	MRPL52	0.43936	0.20197	0.35999	1.47968	1.21873	-0.67733	ac4
NM_001012732	DCTD	-1.45604	0.34243	1.01170	1.47774	0.38394	1.17036	ac4
NM_015002	FBXO21	0.46847	0.22184	0.89663	1.47747	0.00889	-0.60854	ac4
NR_037873	ZHX1	-0.54790	0.14673	0.37261	1.46932	0.82200	-0.31826	ac4
NM_022845	CBFB	-0.13645	0.97878	1.36208	1.46858	-0.71343	-0.44071	ac4
NM_152858	WTAP	-0.41695	-0.70056	0.70349	1.46632	-0.82788	-1.55918	ac4
NM_001254752	UQCRB	0.07015	0.31932	0.26866	1.46109	1.07239	0.84110	ac4
NM_016071	MRPS33	0.43881	1.10748	0.73614	1.46081	-0.26182	-0.89559	ac4
NM_002184	IL6ST	-0.16984	0.71358	1.05331	1.45026	0.73864	1.10036	ac4
NM_001118890	GLRX	0.32783	0.53594	0.90006	1.44789	1.36202	1.22954	ac4
NM_181359	IL6R	-1.78150	0.06218	0.82666	1.44311	-1.36908	0.89990	ac4
NM_001243349	SH3D19	-0.93866	0.51387	1.32006	1.44091	1.12398	0.75794	ac4
NM_017453	STAU1	0.44166	0.50094	1.21478	1.43894	1.07902	0.99780	ac4
NM_001098832	FAM104A	0.00006	1.14343	0.97123	1.43603	-0.58236	0.10937	ac4
NM_033668	ITGB1	-0.09720	0.73522	0.74743	1.43354	1.00063	0.95665	ac4
NM_015368	PANX1	0.16597	0.38786	0.81935	1.42997	0.49535	0.26061	ac4
NM_001620	AHNAK	-0.06633	0.54687	1.02410	1.42906	1.11957	0.88124	ac4
NM_001164213	LRCH1	0.41600	0.58980	1.37430	1.42533	1.15950	0.87747	ac4
NM_005569	LIMK2	0.39473	-0.11645	-0.84191	1.41341	-0.19982	-0.76353	ac4
NM_020347	LZTFL1	0.37216	0.32982	0.63168	1.40766	0.96161	0.60851	ac4
NM_005434	MALL	0.06904	0.19710	0.95585	1.40367	0.74675	0.73530	ac4
NM_000144	FXN	0.49471	0.07517	0.60093	1.40137	-0.51938	-0.10389	ac4
NR_037897	FLJ38109	0.17209	0.12280	0.85426	1.39727	0.64394	0.49949	ac4
NM_001168239	ABL2	0.61378	1.14734	0.78605	1.39577	1.23456	1.24639	ac4
NM_004177	STX3	0.24062	0.65232	1.21749	1.38292	0.44736	0.09307	ac4
NM_007082	RABL2A	0.69809	0.54698	1.19430	1.38107	-0.59277	-0.59144	ac4
NM_001130824	SUPT5H	0.46900	0.60768	0.48402	1.37872	0.86283	0.74050	ac4
NM_080746	RPL10L	-0.07491	-0.50631	-0.00385	1.37853	0.53689	-0.04635	ac4
NM_012294	RAPGEF5	-0.12352	0.42605	0.90129	1.37725	0.52208	0.24895	ac4
NM_002526	NT5E	0.03363	0.12409	-0.41728	1.37662	0.99710	1.11130	ac4
NM_002278	KRT32	0.58952	0.09931	0.32469	1.37140	0.29811	0.72596	ac4
NM_006868	RAB31	0.09833	0.08688	0.80694	1.37087	1.21734	1.03526	ac4

NM_001166420	HDAC8	-0.08195	1.31237	0.69634	1.37055	-0.42813	0.05912	ac4
NM_145791	MGST1	0.62249	0.37222	-1.05724	1.37017	-1.44182	-0.67285	ac4
NR_003940	SNORD80	0.52684	0.73494	-0.23111	1.36784	0.59331	-0.40751	ac4
NM_175839	SMOX	0.58705	0.83789	0.87589	1.36513	1.14195	0.99721	ac4
NM_024482	GMEB1	0.64164	1.31540	1.07836	1.36332	0.78458	0.53507	ac4
NR_030754	MIR622	0.19964	0.17931	0.67573	1.35523	0.07450	0.09526	ac4
NM_003359	UGDH	-0.10256	1.08511	1.11738	1.35445	0.56520	0.67314	ac4
NM_000381	MID1	0.25006	0.21849	0.40254	1.35015	-0.18062	-0.82045	ac4
NM_031483	ITCH	-0.11629	0.41144	1.01279	1.34332	0.70114	0.49268	ac4
NM_001098209	CTNNB1	0.03174	0.33233	0.78646	1.33444	1.22700	1.23685	ac4
NM_006806	BTG3	0.21950	0.38098	1.03072	1.32787	-0.26821	-0.69309	ac4
NM_019610	RBMXL1	0.07458	1.25090	1.16372	1.32700	0.42718	0.17555	ac4
NM_021168	RAB40C	1.20965	0.19387	0.20930	1.32334	0.64618	0.32544	ac4
NM_016500	CXorf26	0.22436	0.62852	1.03676	1.31845	-0.03666	-0.17340	ac4
NM_139164	STARD4	-0.02294	0.57393	0.92933	1.31809	1.06935	0.87888	ac4
NM_001256310	GLS	-0.02084	0.16475	0.78454	1.31203	0.51320	0.63013	ac4
NM_000224	KRT18	0.31085	0.18484	0.71228	1.31165	0.08117	-0.05793	ac4
NM_001136131	APP	0.37484	-0.86998	-0.18919	1.31060	-0.72295	-0.09612	ac4
NM_001042559	EIF4G2	-0.22175	0.14633	0.98009	1.31054	0.04763	-1.12653	ac4
NM_145065	PELI3	0.29694	0.01331	1.13672	1.30870	0.97443	0.97989	ac4
NM_001135700	YWHAZ	0.03526	-0.00937	0.90890	1.30777	-0.57116	-0.20711	ac4
NM_001207069	BZW1	-0.19315	0.69543	1.15933	1.30536	0.35894	0.16827	ac4
NM_133496	SLC30A7	0.29234	0.66061	1.03774	1.30457	0.30955	0.55140	ac4
NM_003373	VCL	0.01265	0.71051	1.21169	1.30164	-0.03221	-0.21488	ac4
NM_001142620	STRA6	-0.76394	0.15442	1.05121	1.29229	0.74474	0.71710	ac4
NM_003831	RIOK3	0.09277	0.49642	0.83282	1.29066	0.78636	0.76006	ac4
NM_198552	FAM89A	0.02403	0.25938	0.90116	1.28977	0.33905	0.17127	ac4
NM_000344_1	SMN1	0.79032	0.93595	1.04745	1.28646	0.68648	0.47815	ac4
NM_181889	UBE2D3	0.80446	0.66271	0.69801	1.28578	0.90911	0.73134	ac4
NR_015395	LOC541471	-0.47208	0.11125	0.79392	1.28087	0.95340	1.08503	ac4
NM_001098638	RNF169	0.22019	0.18799	0.51020	1.27629	0.89096	0.47945	ac4
NM_001128219	VGLL4	0.16336	0.00412	0.67259	1.27305	0.51620	1.04924	ac4
NM_004457	ACSL3	0.80328	0.16325	0.43798	1.27042	0.80749	-0.58499	ac4
NM_001004127	ALG11	-0.09638	0.53976	0.47856	1.26891	0.61982	0.31776	ac4
NM_024576	OGFRL1	-0.34471	0.08985	0.65803	1.26749	0.24118	0.25198	ac4
NM_007099	ACP1	-0.33341	0.26473	-0.62942	1.26650	0.32973	-0.28703	ac4
NM_014755	SERTAD2	0.33386	1.00854	1.19958	1.26248	0.81977	0.39983	ac4
NM_001135592	RPS27A	0.68429	-0.83068	0.69219	1.26137	-0.19961	-0.47607	ac4
NM_004078	CSRP1	0.22855	0.73433	1.19991	1.26010	0.30804	0.56770	ac4
NM_001227	CASP7	-0.02561	0.06731	0.72343	1.25669	0.97342	0.70076	ac4
NM_032239	LARP1B	0.26345	0.38072	0.93137	1.25636	-0.46271	-0.59028	ac4
NM_020824	ARHGAP21	-0.25360	0.27715	0.88702	1.25565	0.47170	0.08447	ac4
NM_182491	ZFAND2A	0.23587	0.86218	1.14323	1.25454	-0.25652	-0.07464	ac4
NM_000859	HMGCR	0.36116	0.79277	0.84515	1.25396	0.49312	0.47335	ac4
NM_172218	SPAG1	0.37001	0.64659	0.65191	1.25149	0.47331	-0.40113	ac4
NM_001031628	SMAGP	0.59519	-0.01269	1.18252	1.25010	0.56581	0.39002	ac4
NM_003898	SYNJ2	0.48134	0.46624	0.90771	1.25001	0.82670	0.76784	ac4

NR_033721	DNAJC19	-0.81853	0.06587	0.61891	1.24200	-0.33194	0.16787	ac4
NM_003615	SLC4A7	-0.55651	0.57877	0.78751	1.24075	1.04147	0.99798	ac4
NM_017633	FAM46A	-0.24915	-0.41429	1.06032	1.24038	0.21111	-0.09835	ac4
NM_206837	OSCP1	0.81112	0.54087	0.59102	1.23953	-0.40735	-0.45188	ac4
NM_006625	SRSF10	0.59717	0.72029	0.81679	1.23697	-0.91549	-0.80056	ac4
NM_001005741	GBA	-1.65353	-1.47183	0.38562	1.23334	0.52801	0.12827	ac4
NM_032148	SLC41A2	0.08902	0.41146	0.90010	1.23061	0.58665	0.42604	ac4
NM_139046	MAPK8	-0.13351	-0.95061	1.10808	1.22992	-0.98873	-0.27310	ac4
NM_002623	PFDN4	0.40592	-0.00605	0.41628	1.22839	1.00442	1.14395	ac4
NM_022073	EGLN3	0.31142	0.31666	0.79647	1.22793	0.11916	-0.01392	ac4
NM_005494	DNAJB6	0.40041	0.80166	0.87581	1.22631	0.81186	0.46049	ac4
NM_153207	AEBP2	0.21630	0.74377	0.80331	1.22556	0.31818	0.49437	ac4
NM_018654	GPRC5D	0.81348	0.71687	0.30977	1.22402	-0.76551	-0.75982	ac4
NM_005882	MAEA	0.65266	1.04593	0.76600	1.22231	-1.19236	-0.30188	ac4
NM_007118	TRIO	0.02893	0.23647	0.58877	1.21917	0.95122	0.48746	ac4
NM_014452	TNFRSF21	-0.06771	0.12051	0.94093	1.21794	1.11427	0.95581	ac4
NM_012334	MYO10	-0.11281	0.11761	0.71001	1.21750	1.12312	0.87163	ac4
NR_024110	SBDSP1	-0.14919	0.64110	0.72615	1.21274	-0.40459	-0.18725	ac4
NR_038957	LOC10050671	1.04114	-0.37854	-0.85500	1.21137	-0.15996	-0.18571	ac4
NM_001252385	TNIP1	-0.68449	-0.35650	1.00896	1.21051	1.04251	1.10585	ac4
NM_001498	GCLC	0.32219	0.47129	0.92362	1.21035	-0.04424	-0.17737	ac4
NM_016441	CRIM1	0.01582	-0.18739	0.40453	1.20990	0.70992	0.78779	ac4
NR_026844	ANKRD36BP1	-0.08349	0.54870	0.59743	1.20980	0.41168	-0.03466	ac4
NM_001135095	FNDC3B	-0.23703	0.39247	-0.31816	1.20929	1.16968	0.46898	ac4
NM_016355	DDX47	0.15215	0.38346	1.07354	1.20920	0.28161	0.03043	ac4
NM_001136025	PLS3	0.08440	0.75997	0.79079	1.20826	1.19112	1.07139	ac4
NR_033926	LOC644961	0.59173	0.77937	1.16700	1.20662	0.77562	0.90114	ac4
NM_145254	TMEM170A	0.40597	0.67801	0.94628	1.20056	0.20216	0.04945	ac4
NM_001146160	TATDN1	0.31885	-0.85639	-0.80833	1.20032	-0.48907	-1.02810	ac4
NM_001550	IFRD1	0.26489	0.85587	1.04612	1.19922	0.06208	0.01039	ac4
NM_001142418	MORF4L2	0.79554	-1.07860	1.06708	1.19712	0.62696	-0.42613	ac4
NR_026911	RPL21P28	0.68939	0.23016	0.82824	1.19256	0.79634	0.09178	ac4
NM_001100623	PLEKHB2	1.02654	0.98843	0.97128	1.19045	0.63820	0.37292	ac4
NM_001128627	SPIRE1	0.06925	-0.39966	0.58368	1.18994	-0.00477	-0.03777	ac4
NM_183323	PAIP1	0.08481	-0.22894	-0.43459	1.18445	-0.99226	-1.57327	ac4
NM_016447	MPP6	-0.12759	0.21313	0.64373	1.18389	-0.06021	-0.46055	ac4
NM_139281	WDR36	-0.14920	0.33657	0.55240	1.18187	-0.05620	-0.49079	ac4
NM_012309	SHANK2	0.20162	0.48580	0.51552	1.18069	0.53855	0.64214	ac4
NM_001002258	ATP5G3	0.45652	-0.51264	0.21967	1.17774	-0.78497	0.28201	ac4
NM_207042	ENSA	0.95212	0.09374	0.49046	1.17742	-0.04405	-0.00764	ac4
NM_014043	CHMP2B	0.11832	0.35718	0.49885	1.17418	0.05801	0.20296	ac4
NM_004728	DDX21	0.01836	0.52881	0.74460	1.17302	-0.03369	-0.25149	ac4
NM_015131	WDR43	0.21965	0.92040	0.88233	1.16824	-0.17393	-0.52834	ac4
NM_001256293	KRT8	0.51804	-0.15174	0.24938	1.16684	0.38720	0.16311	ac4
NM_018360	TXLNG	-0.43816	0.70143	0.74137	1.16681	-0.03775	-0.52582	ac4
NM_003567	BCAR3	0.18751	0.52427	1.14751	1.16569	0.88068	0.91719	ac4
NM_005399	PRKAB2	-0.20744	0.09566	0.82549	1.16448	0.66000	0.57630	ac4

NM_001015882	DNAJC25	0.73075	0.94725	0.11524	1.16329	-0.18371	-0.47143	ac4
NM_015106	RAD54L2	0.06487	0.00334	1.11285	1.16046	0.01157	-0.15783	ac4
NM_153698	C9orf21	0.14456	0.73472	0.78676	1.15788	0.26487	-0.00065	ac4
NM_001418	EIF4G2	0.19036	0.46441	0.62626	1.15542	0.48568	0.06408	ac4
NM_012482	ZNF281	0.21052	0.92829	0.73315	1.15255	1.03732	0.87958	ac4
NM_001145412	NFE2L2	-0.60861	1.01876	0.34869	1.15029	0.15308	0.48318	ac4
NM_001032	RPS29	0.84453	0.49599	0.19822	1.14952	0.80754	0.85310	ac4
NM_145902	HMGA1	0.12471	0.24230	0.56275	1.14679	0.75475	0.46107	ac4
NM_032509	MAK16	0.32161	0.81441	0.66078	1.14502	-0.24420	-0.50661	ac4
NM_001242508	OSBPL1A	-0.41397	-0.32010	-0.54046	1.14133	0.36950	-0.07008	ac4
NM_001079812	DIAPH1	0.03035	0.19562	0.95254	1.14104	0.72192	0.59407	ac4
NM_001130481	VRK2	0.72514	0.66959	-0.21410	1.13342	-0.11506	-0.10775	ac4
NM_001008485	SLC41A3	-1.01061	-0.22566	-0.14803	1.12889	-0.69069	-0.01068	ac4
NM_005797	MPZL2	0.06071	0.27296	0.51999	1.12886	0.53736	0.61487	ac4
NM_005387	NUP98	0.45159	0.65060	1.12144	1.12827	-0.20946	-1.33369	ac4
NM_001177880	SYT12	0.41683	-0.37611	0.23228	1.12615	1.01727	0.80017	ac4
NM_004396	DDX5	0.29965	0.63157	0.89818	1.12568	-0.28089	-0.41588	ac4
NM_001423	EMP1	-0.01180	0.45397	0.83943	1.12500	0.84440	0.82338	ac4
NM_001134423	CDV3	-0.14631	0.30038	0.07057	1.12183	-1.04180	-1.45516	ac4
NM_005746	NAMPT	0.20158	0.73779	0.87296	1.12175	0.14269	-0.06855	ac4
NM_001195101	PTP4A2	-0.02358	0.29917	0.35852	1.11660	-0.53980	-0.85624	ac4
NM_014377	DNAJC2	-0.09544	0.73458	0.70745	1.11639	0.21669	0.43208	ac4
NM_001007098	SCP2	0.12092	0.43494	0.54277	1.11591	1.06441	0.85738	ac4
NM_001008895	CUL4A	0.52645	0.65992	0.74018	1.11569	0.53928	0.33672	ac4
NM_003467	CXCR4	0.34076	0.17623	0.56252	1.11354	-0.64546	-0.29250	ac4
NM_015465	GEMIN5	0.08946	0.30658	0.92545	1.11295	-0.28999	-0.69814	ac4
NM_030674	SLC38A1	0.14842	0.16788	0.78129	1.11057	0.78691	0.38205	ac4
NM_001142618	STRA6	-1.18752	-0.59969	-0.30200	1.10992	0.91912	0.77801	ac4
NR_038354	WDR20	-0.48324	0.13024	0.50010	1.10735	0.09523	-0.15918	ac4
NM_144726	RNF145	-0.57025	0.07228	0.71364	1.10713	0.23080	0.23128	ac4
NM_001013399	CCNC	-0.60162	0.38108	0.15439	1.10647	-0.01611	-0.18186	ac4
NM_002886	RAP2B	0.22951	0.96803	1.08593	1.10350	-0.08661	-0.12077	ac4
NM_001080123	PRNP	0.32703	0.28645	0.21649	1.10065	0.21139	-0.11613	ac4
NM_004925	AQP3	-0.06240	-0.07818	0.85895	1.10044	0.97087	0.88385	ac4
NM_001170804	LARP4	-1.53537	0.37654	-0.34399	1.10006	0.11210	-0.79754	ac4
NM_024422	DSC2	-0.11285	0.73295	0.63168	1.09985	1.01159	0.52128	ac4
NM_024315	C7orf23	0.24253	0.40303	0.76928	1.09706	0.23398	-0.03177	ac4
NM_181468	EIF6	0.61531	0.31978	0.74515	1.09432	0.06167	0.54465	ac4
NM_001001890	RUNX1	-0.01685	0.13905	0.76877	1.09300	0.26821	0.23676	ac4
NR_036652	LOC648809	0.22511	0.26210	1.06176	1.09121	0.86645	0.92883	ac4
NR_036510	UROD	0.16999	0.44881	-0.51953	1.09017	-0.90380	0.17980	ac4
NM_000416	IFNGR1	-0.02625	0.71981	1.03800	1.08900	0.68688	0.55728	ac4
NM_001033925	TIAL1	0.41552	0.55580	1.07720	1.08728	0.22495	-0.18489	ac4
NM_022041	GAN	0.43623	0.88085	0.86690	1.08561	0.46489	0.34477	ac4
NM_152663	RALGPS2	-0.21101	0.13079	0.19275	1.08500	0.90897	0.73014	ac4
NM_005788	PRMT3	0.51705	0.33546	-0.00219	1.08453	0.14961	0.15171	ac4
NM_020360	PLSCR3	0.17254	0.30836	0.56803	1.08439	0.91338	1.04076	ac4

NM_001010938	TNK2	-0.07591	-0.00235	-0.52959	1.08394	0.77787	0.77442	ac4
NM_144609	CCDC43	0.26436	0.56704	0.49885	1.08100	-0.56092	0.02001	ac4
NM_001166271	SPATA13	0.11669	0.16920	0.76359	1.07737	0.71028	0.42267	ac4
NM_002823	PTMA	0.10900	0.62262	-0.70780	1.07528	-0.95856	-1.97394	ac4
NM_005648	TCEB1	0.29277	-0.15346	0.01266	1.07101	-0.55709	-0.76090	ac4
NM_001032296	STK24	0.27811	0.52780	0.97841	1.06777	0.90747	0.79207	ac4
NM_001018006	TPM1	0.20718	0.28572	0.86193	1.06758	-0.16297	-0.36872	ac4
NM_006066	AKR1A1	0.33487	-0.26420	0.13636	1.06757	-0.13896	0.65874	ac4
NM_001167870	ATP5SL	0.92215	-0.79686	-0.36191	1.06553	-0.47266	-0.24003	ac4
NM_001136232	SEC13	-0.03645	0.68198	0.19574	1.06540	0.43703	0.33987	ac4
NM_020143	PNO1	0.33548	0.32768	0.60122	1.06539	-0.33161	-0.64483	ac4
NM_004520	KIF2A	0.01685	0.10365	-0.04261	1.06531	0.45493	0.22790	ac4
NM_138730	HMGN3	0.88231	0.92683	0.99049	1.06469	0.18162	0.20166	ac4
NM_005358	LMO7	0.01202	0.31461	0.66626	1.06455	0.45256	-0.62863	ac4
NM_003242	TGFBR2	0.04241	-0.89141	0.46071	1.06313	0.27423	0.34207	ac4
NM_198700	CELF1	-0.41939	-1.04294	0.17546	1.06236	0.18879	-0.30616	ac4
NM_021982	SEC24A	0.13795	0.40775	0.73684	1.06177	0.80722	0.70426	ac4
NM_003414	ZNF267	0.12785	0.51990	0.67933	1.05937	0.37616	0.63009	ac4
NM_152400	C4orf32	0.10677	0.23635	0.54062	1.05791	0.03339	-0.11326	ac4
NM_003442	ZNF143	-0.00511	-0.10488	0.61783	1.05609	0.84411	0.70614	ac4
NM_001159675	SYNCRIP	0.77673	0.69761	0.50991	1.05277	-0.72513	-0.56271	ac4
NM_015055	SWAP70	0.04879	0.47939	0.67303	1.05063	0.50364	0.34672	ac4
NM_001135732	TOM1	0.28729	0.51518	0.11864	1.04991	-0.15765	0.81794	ac4
NM_016028	SUV420H1	-0.00973	0.35700	0.58018	1.04956	0.46003	0.21981	ac4
NM_004504	AGFG1	0.36093	0.52004	0.84599	1.04952	0.41744	0.12182	ac4
NM_015878	AZIN1	0.47894	0.79634	0.86087	1.04547	0.67058	0.54373	ac4
NM_013316	CNOT4	0.68509	-0.75540	-0.09674	1.04323	0.50555	-0.52116	ac4
NM_002524	NRAS	0.04645	0.17720	0.64821	1.04278	0.70186	0.33396	ac4
NM_022455	NSD1	0.16464	0.31406	0.96623	1.03967	0.56241	0.51816	ac4
NM_003584	DUSP11	0.04070	0.25539	0.58644	1.03891	0.26947	0.18143	ac4
NM_020954	RNF213	-0.12975	-0.11560	0.69026	1.03873	-0.22050	-0.10251	ac4
NM_001134432	AZI2	0.30403	0.68477	-0.02984	1.03702	0.58154	-0.08688	ac4
NM_031216	SEH1L	0.27879	0.29185	0.61915	1.03590	0.05347	-0.47605	ac4
NM_005900	SMAD1	0.32657	0.25454	0.22585	1.03191	0.77490	-0.21736	ac4
NR_027716	H2AFJ	0.32047	-0.66038	-0.40982	1.03011	-0.10977	-0.16844	ac4
NM_032299	DCUN1D5	0.17572	0.08079	0.43337	1.02956	-0.09731	-0.44503	ac4
NM_024429	PRKAG2	0.27681	0.44263	0.70773	1.02849	-0.03148	-0.26969	ac4
NM_025076	UXS1	0.76916	-0.48220	0.34152	1.02600	-0.05078	0.73500	ac4
NM_001035235	SRA1	0.44913	-0.14239	0.33566	1.02554	-0.33347	-0.44018	ac4
NM_018566	YOD1	-0.05314	0.81463	0.75241	1.02495	0.74812	0.62072	ac4
NM_001102426	TBC1D8	0.01835	0.19019	0.79965	1.02472	0.26574	0.32047	ac4
NM_175742_1	MAGEA2	0.47512	0.72368	0.86600	1.02426	-0.04956	0.64142	ac4
NM_001197122	IRF3	0.09316	0.14846	0.24642	1.02326	0.54834	0.84051	ac4
NM_001948	DUT	-0.17912	-0.51633	0.03350	1.02283	-1.56535	-3.00555	ac4
NM_001008657	TCOF1	0.04869	0.39136	0.87810	1.02111	0.92470	0.78607	ac4
NR_004390	SNORA57	-0.22667	0.71265	-0.98243	1.01972	0.32652	-0.15405	ac4
NM_003902	FUBP1	0.23183	0.31266	0.64288	1.01594	0.66322	0.44340	ac4

NM_032667	BSCL2	0.20588	-1.35078	-0.74774	1.01504	-0.02090	0.87552	ac4
NM_017634	KCTD9	0.04196	0.55900	0.58889	1.01492	0.66824	0.22883	ac4
NM_015176	FBXO28	0.19257	0.35433	0.46553	1.01440	0.30963	0.10966	ac4
NM_001243985	RELA	-0.25212	0.06360	-0.09525	1.01392	0.24168	-0.55812	ac4
NM_016021	UBE2J1	0.06943	0.31335	0.66905	1.01390	0.50921	0.39091	ac4
NM_153488	MAGEA2B	0.25781	0.47640	0.89307	1.00942	0.39967	0.73960	ac4
NM_001042697	ZSWIM7	0.19501	-0.16060	-0.36477	1.00815	-0.92492	-0.64933	ac4
NM_024524	ATP13A3	-0.44900	0.21631	0.47701	1.00796	0.35946	0.30762	ac4
NM_003253	TIAM1	-0.07859	-0.04520	0.73106	1.00786	0.38437	0.07764	ac4
NM_001009894	C12orf29	-0.11568	0.36741	0.55389	1.00440	-0.03013	-0.03875	ac4
NM_001177591	PPP2R2A	0.21124	0.63793	0.01305	1.00346	-0.41407	-1.28629	ac4
NM_145014	HYLS1	0.66862	0.15564	0.31577	1.00223	0.32708	-0.96741	ac4
NM_030780	SLC25A32	0.18596	0.84142	0.83639	1.00218	-0.08503	-0.20820	ac4
NM_001142428	MORF4L2	-0.00536	0.53876	0.07161	1.00201	0.24348	-2.36453	ac4
NM_015092	SMG1	-0.35538	0.48407	0.62947	1.00146	0.19959	0.02340	ac4
NM_014240	LIMD1	-0.09077	-0.08292	0.93049	1.00013	-0.12817	0.00711	ac4
NM_007097	CLTB	2.58594	2.40061	3.49588	3.89189	4.45473	4.30184	ac5
NM_033382	SEC14L2	-0.02491	0.73773	1.98432	2.60974	3.52619	2.84013	ac5
NR_027783	SAT1	2.29330	2.88838	1.91770	2.17245	3.31950	3.22607	ac5
NM_024729	MYH14	2.83873	-0.11435	-0.13222	2.33398	3.10199	0.75744	ac5
NM_017527	LY6K	0.07838	-0.49497	1.35288	1.33556	3.10115	2.97038	ac5
NM_013451	MYOF	0.79296	1.21389	1.43486	2.46692	3.05210	2.25983	ac5
NM_001172569	MYD88	1.65743	2.17729	1.27414	0.60435	2.74993	2.52356	ac5
NM_001025252	TPD52	-1.06739	-0.45263	0.66290	-0.09917	2.70422	2.63757	ac5
NM_005230	ELK3	-0.24095	-0.05635	1.59264	2.03686	2.65455	2.62177	ac5
NM_007219	RNF24	0.61707	1.19238	2.15094	2.19480	2.58892	2.50243	ac5
NM_012258	HEY1	0.85684	0.03175	1.35249	1.52127	2.57247	2.33979	ac5
NM_001174101	PRR7	1.66661	1.86167	0.30144	1.61346	2.53457	2.50582	ac5
NM_130898	CREB3L4	0.85725	0.68859	1.50561	1.15152	2.41798	0.81523	ac5
NM_012114	CASP14	0.32851	-0.13739	0.09878	0.43386	2.40475	2.37791	ac5
NM_012250	RRAS2	0.43528	0.83369	1.41488	2.23504	2.36749	2.36317	ac5
NM_001128628	PAK6	0.53083	1.77171	0.75828	0.13137	2.31802	2.08620	ac5
NR_001456	SNORD38A	0.78204	1.75705	0.84529	2.05405	2.25440	-1.05343	ac5
NR_002575	SNORA27	1.10397	1.78131	-1.15541	-0.14518	2.21627	1.19579	ac5
NM_001243186	PIM1	0.69358	0.47166	0.54917	0.67903	2.21177	2.02050	ac5
NM_016445	PLEK2	0.09714	0.31116	1.48009	1.63027	2.20878	2.12433	ac5
NM_198149	SHISA4	0.89934	0.20387	0.81005	0.71482	2.19256	1.65072	ac5
NM_001205315	STEAP4	-0.03469	1.04414	1.28769	2.05905	2.19119	1.99540	ac5
NM_032421	CLIP2	-0.46285	-0.07423	0.60857	0.38331	2.16832	2.12591	ac5
NM_017729	EPS8L1	1.01795	2.00937	1.97234	2.02959	2.15633	1.89247	ac5
NM_001199535	TGIF2-C20ORI	1.80707	1.34136	1.22231	0.98235	2.12695	0.97273	ac5
NM_058229	FBXO32	-0.14921	0.06015	0.59724	0.87483	2.11278	2.06273	ac5
NM_018704	CTTNBP2NL	0.21624	0.69358	1.00219	1.09828	2.11214	1.91821	ac5
NR_046325	KDM5B-AS1	0.19213	0.38686	0.41694	0.35960	2.09147	1.79670	ac5
NM_001031847	CPT1A	1.59172	0.51556	0.64934	0.28151	2.07619	1.94339	ac5
NR_026875	NEURL3	0.39983	1.03625	1.09165	0.57113	2.02786	1.97310	ac5
NM_005811	GDF11	0.07065	0.15202	0.41395	0.56767	2.02078	1.99116	ac5

NM_001080855	PXN	-0.07675	0.39535	1.40637	1.20153	1.99637	1.92442	ac5
NM_001102594	DTX2	0.18834	-0.14856	-0.02140	0.03172	1.98462	1.92371	ac5
NM_001135821	FDPS	0.27582	-0.08645	0.44452	0.44691	1.97761	1.62677	ac5
NM_001136153	ATF6B	1.62012	1.77465	1.72230	1.00587	1.96325	-0.29904	ac5
NR_033961	LOC729862	0.18550	0.40097	0.23591	0.78946	1.94545	1.92234	ac5
NM_024887	DHDDS	-0.47837	-0.12843	-0.03763	0.29264	1.92879	0.62421	ac5
NM_021575	AP2S1	0.45956	0.57170	1.38021	1.84233	1.89881	0.80283	ac5
NR_000024	SNORD46	1.36700	-1.11350	0.84458	-0.74097	1.88697	-0.09978	ac5
NM_001199040	STRA6	-0.57203	-0.72440	1.42861	1.64380	1.86638	0.85743	ac5
NR_027780	HMGXB4	1.25240	0.20349	-0.06394	0.95820	1.86024	0.34537	ac5
NM_003456	ZNF205	0.56397	1.08526	1.21599	0.35362	1.85680	1.78969	ac5
NM_001178080	STAT6	-0.52315	1.48782	0.98536	1.30019	1.84840	-1.16186	ac5
NM_001203258	MTA1	1.74830	1.41135	-1.07212	1.43286	1.82688	0.57565	ac5
NM_001042758	SRGAP2	-0.21290	0.29192	0.08377	0.05237	1.82575	1.70599	ac5
NM_001017526	ARHGAP8	1.09474	0.25378	1.19314	-0.34608	1.82356	1.40668	ac5
NM_016816	OAS1	0.93978	0.64618	0.75185	1.35210	1.80930	1.35847	ac5
NM_001164165	GPR75-ASB3	1.12114	1.40878	0.07740	0.75871	1.80314	0.66229	ac5
NM_004354	CCNG2	0.22995	-0.46259	-0.70659	0.02090	1.77979	1.50064	ac5
NM_002688	42983	0.04844	0.05084	0.47360	0.62201	1.77459	0.66700	ac5
NM_003234	TFRC	0.63566	1.08426	0.31029	0.87923	1.76666	1.47962	ac5
NM_004087	DLG1	-0.17095	1.60478	0.76926	1.03777	1.74808	0.90009	ac5
NM_007045	FGFR1OP	-0.03653	1.18017	0.77394	0.90090	1.74390	0.50623	ac5
NM_145267	C6orf57	0.92351	0.27890	0.01544	-0.19206	1.74199	1.46607	ac5
NR_033257_1	XAGE1E	0.57171	-0.37869	1.00323	1.02771	1.73946	1.63351	ac5
NM_001014834	PAK4	0.92065	0.38095	-0.98972	0.65695	1.73154	0.56192	ac5
NM_001244390	NUDT2	0.79852	1.48126	1.15810	0.77868	1.72152	-0.04737	ac5
NM_001079675	ETV4	0.75788	0.02898	-0.00564	-0.00841	1.71817	1.37177	ac5
NM_033201	C16orf45	-0.05764	-0.21739	0.16628	0.69352	1.70437	1.52782	ac5
NR_023345	CNKSR1	-0.21148	0.61916	-0.15440	0.80021	1.69558	-1.08384	ac5
NM_018370	DRAM1	0.12014	0.14812	0.53274	0.62349	1.69540	1.28774	ac5
NM_001142798	C17orf49	1.34080	0.26095	1.43985	0.01837	1.67011	0.70009	ac5
NM_174908	CCDC50	0.16919	0.13954	0.59322	1.05496	1.66626	1.30629	ac5
NM_138638	CFL2	0.08673	-0.61708	0.44149	-0.93042	1.66315	1.22939	ac5
NM_001009934	DNASE1L1	0.26201	0.01325	0.99352	0.54539	1.63127	1.27297	ac5
NM_001190274	FBXO11	-0.25255	0.53168	0.59990	0.82225	1.59116	1.28033	ac5
NM_178452	DNAAF1	0.24130	0.10870	0.88227	1.01975	1.58334	1.50319	ac5
NR_033251_1	XAGE1C	0.20963	0.31931	0.53486	0.70345	1.57834	1.49226	ac5
NM_032631	HDGFRP2	1.16387	-0.51178	-0.20116	-0.86716	1.57764	0.60988	ac5
NM_006617	NES	-0.32078	0.04632	1.26263	1.37125	1.56647	0.86960	ac5
NM_001164722	PTAFR	0.02618	-0.03279	0.94113	1.08574	1.54187	1.48674	ac5
NM_001105539	ZBTB10	-0.44105	1.04031	0.65982	0.29132	1.54018	1.30018	ac5
NM_001040100	SPTSSB	0.09416	-0.29061	0.02731	-0.03521	1.53984	1.02377	ac5
NM_001190708	MTRNR2L10	0.48686	0.05849	0.18007	0.03283	1.53849	1.47357	ac5
NM_001206559	POLR1D	1.00700	0.22469	0.54608	0.41286	1.53764	1.39012	ac5
NM_181349	SMURF1	0.62265	0.88659	1.04144	0.96837	1.53237	1.27399	ac5
NM_016451	COPB1	0.26089	0.34786	0.65109	1.40681	1.52702	1.50958	ac5
NM_001167671	LPP	-0.33130	-0.21001	-0.50956	1.01636	1.52500	1.22277	ac5

NM_001206947	PICALM	0.44143	0.23383	0.76773	1.33899	1.52399	-0.15889	ac5
NM_004468	FHL3	-0.18894	0.19153	1.44057	1.38906	1.52215	1.04873	ac5
NM_006526	ZNF217	0.19209	0.55355	0.63207	0.58294	1.52019	1.33049	ac5
NM_001195203	EEF1D	1.09620	0.43762	0.76347	0.86996	1.49610	1.16745	ac5
NM_014690	FAM131B	-0.66592	-0.19402	0.40030	-0.20608	1.48877	0.24312	ac5
NR_003089	OTUB1	0.78490	0.91906	1.46823	0.22078	1.48759	1.23328	ac5
NM_014688	USP6NL	0.27301	1.36864	-0.14800	0.34333	1.47443	0.31298	ac5
NR_024543	SNHG7	0.73912	-0.11880	1.19481	1.30503	1.45462	0.95640	ac5
NM_001001788	RAET1G	0.36872	-0.41875	0.12793	0.28604	1.44507	1.24880	ac5
NM_007085	FSTL1	0.01836	0.17717	1.15604	1.31098	1.44223	1.41994	ac5
NM_199437	PRDM10	0.48634	0.34349	0.08205	1.14015	1.43319	1.08721	ac5
NM_001001924	MTUS1	0.22016	0.40126	0.29324	0.66693	1.42102	1.02270	ac5
NM_139266	STAT1	1.04830	0.53282	0.80215	0.46590	1.41631	0.90927	ac5
NM_003083	SNAPC2	0.32343	0.25063	0.69099	0.80274	1.41015	1.02980	ac5
NM_002518	NPAS2	0.06582	-0.02873	0.75151	1.14664	1.40739	1.40247	ac5
NM_001085481	MAP1LC3B2	0.36225	0.34789	0.35295	0.61997	1.38297	1.35121	ac5
NM_001128914	PCBP2	0.23525	0.25338	0.27949	0.25830	1.38034	1.28298	ac5
NM_001105248	TMC5	0.00062	0.14275	-0.05988	0.03693	1.37875	1.34870	ac5
NM_005764	PDZK1IP1	0.90729	-0.21356	0.18508	-0.23852	1.37173	0.49409	ac5
NM_002583	PAWR	-0.00862	0.70097	1.20164	1.18121	1.36950	1.32164	ac5
NM_000693	ALDH1A3	-0.01768	0.30798	0.85330	1.17360	1.36100	1.17520	ac5
NM_016246	HSD17B14	0.45068	-0.27840	-0.50373	0.27736	1.34269	1.28192	ac5
NM_002483	CEACAM6	0.09347	-0.09770	0.14780	0.58277	1.33675	1.15465	ac5
NM_001077397	IRF2BP2	0.85542	1.32025	1.01894	0.76072	1.33254	1.19924	ac5
NM_001077241	SLC25A45	-0.67530	-1.13115	1.28834	-0.08776	1.32727	0.46154	ac5
NM_015299	KHNYN	0.02322	0.10549	0.09963	0.29566	1.32540	1.20643	ac5
NM_001250	CD40	0.83057	0.00550	-0.01006	0.36677	1.32447	0.98001	ac5
NM_005057	RBBP5	1.17979	0.40931	0.75902	1.20136	1.32088	0.39414	ac5
NM_001174156	SAMD8	0.13434	0.81176	0.99191	1.13547	1.31984	1.28663	ac5
NM_020169	LXN	-0.07924	0.25592	0.09200	0.12486	1.31950	1.04317	ac5
NM_018372	LRIF1	0.73009	1.12605	0.78986	0.77089	1.31574	1.15757	ac5
NM_025185	TANC2	-0.05290	0.09860	0.67264	0.57737	1.30932	0.85842	ac5
NR_027692	PHF1	0.71200	0.17022	0.07380	0.15069	1.30664	1.05625	ac5
NM_176783	PSME1	-0.02587	0.26094	0.84580	0.33407	1.30432	1.06251	ac5
NM_033240	PML	0.35133	-0.13289	-0.05331	-0.39808	1.30419	1.19868	ac5
NM_015100	POGZ	0.37536	0.04995	0.67890	0.50418	1.29799	0.48325	ac5
NM_006618	KDM5B	0.12805	0.70154	1.09534	0.92959	1.29714	1.03616	ac5
NM_006551	SCGB1D2	-0.68165	0.41138	0.22333	1.11726	1.29128	0.86018	ac5
NM_015107	PHF8	0.48470	1.19460	0.26516	0.92167	1.28842	1.23793	ac5
NM_001039573	SEC14L1	-0.00697	-0.01072	0.57281	0.64653	1.28687	1.16605	ac5
NM_201559	FOXO3	0.65443	0.89705	0.67831	1.00458	1.27889	0.69262	ac5
NM_002845	PTPRM	-0.12634	0.29401	0.11990	-0.08254	1.27554	1.10908	ac5
NM_203364	CAPRIN1	-0.02706	-0.17724	0.03784	0.25875	1.25837	0.57370	ac5
NM_001677	ATP1B1	0.03029	0.25811	0.78784	1.22646	1.25784	1.17457	ac5
NM_014035	SNX24	0.25737	0.16381	0.53417	1.08844	1.25277	1.02942	ac5
NM_017836	SLC41A3	-0.20066	-0.65592	-0.29073	-1.46280	1.24842	0.85415	ac5
NM_139201	GIT2	0.47341	0.25901	0.66545	0.74201	1.24737	1.23525	ac5

NM_173076	ABCA12	-0.11705	0.25284	0.66594	0.68256	1.24476	1.20774	ac5
NR_028272	NEAT1	-0.34583	1.01203	0.52800	0.29403	1.24240	1.21286	ac5
NM_138368	AP5B1	0.05360	0.92751	0.19753	-0.31067	1.24110	1.07826	ac5
NR_033251_2	XAGE1C	0.33691	-0.30980	0.63364	0.29661	1.24079	0.91347	ac5
NM_001130867	ERCC2	-0.64868	0.07285	-0.33004	0.44779	1.23953	1.04217	ac5
NM_003358	UGCG	-0.26097	0.33907	0.57254	0.63435	1.23497	1.21532	ac5
NM_198447	GOLT1A	0.10340	-0.15305	0.37991	0.92393	1.23241	1.19669	ac5
NM_001081675	KLHL38	-0.10119	-0.60069	-0.33229	-0.13610	1.23017	0.66745	ac5
NM_181887	UBE2D3	-0.00763	1.09058	0.61540	1.09850	1.22936	1.03611	ac5
NM_001007553	CSDE1	0.11012	0.47191	0.22344	0.89164	1.22935	0.91986	ac5
NM_018474	PLK1S1	0.07882	0.43655	0.36166	0.48476	1.22619	0.53277	ac5
NM_002780	PSG4	-0.30666	0.51581	0.60225	1.05847	1.22540	0.85522	ac5
NM_153221	CILP2	-0.00306	0.30538	0.88933	0.85682	1.22426	1.19286	ac5
NM_015313	ARHGEF12	0.08254	0.47670	-0.67965	-0.12629	1.21490	-0.63131	ac5
NM_003330	TXNRD1	0.08180	-0.08696	0.63927	0.50279	1.21344	1.20233	ac5
NM_006480	RGS14	0.38214	-0.26402	-0.16163	0.14740	1.21110	1.18816	ac5
NM_005197	FOXN3	0.84374	0.97130	-0.06953	0.19357	1.20895	1.07646	ac5
NM_001242394	SYTL3	0.59482	0.27903	-0.61932	0.08016	1.20759	0.68454	ac5
NM_001079803	GAA	0.53955	0.26942	0.34446	0.27945	1.20246	0.66489	ac5
NM_004635	MAPKAPK3	-0.06643	-0.31510	0.07761	0.05244	1.20227	0.89669	ac5
NM_000636	SOD2	-0.21667	-0.05131	-0.15744	0.31813	1.18993	0.50824	ac5
NM_001128208	LEPROTL1	0.06717	-0.31773	0.25995	0.30029	1.18507	0.79569	ac5
NM_004830	MED23	0.29663	0.27253	0.16250	0.53400	1.18503	0.45251	ac5
NM_001003698	RREB1	0.95577	-0.49712	-0.32939	0.40067	1.18434	0.38042	ac5
NM_001100599	ZNF707	0.88528	0.79810	0.56647	0.86454	1.18324	1.06715	ac5
NR_034178	SRGAP2P2	0.31736	0.05769	0.08758	-0.35857	1.18210	0.99202	ac5
NM_144985	CDH24	0.06356	-0.66672	0.04516	-0.99647	1.17963	0.94098	ac5
NM_001142397	CTIF	-1.33924	0.82372	0.62324	-0.11490	1.17929	0.67476	ac5
NM_005933	MLL	0.69214	-0.52410	0.75866	1.01380	1.17868	0.75768	ac5
NM_005137	DGCR2	0.34377	-0.15987	0.77827	-1.06181	1.17705	0.92867	ac5
NM_021199	SQRDL	0.01629	0.04545	0.78100	0.99158	1.17563	1.06646	ac5
NM_020778	ALPK3	-0.04063	0.00197	0.36084	0.32608	1.17272	1.09454	ac5
NM_012110	CHIC2	0.69160	0.96622	1.14224	0.90292	1.16897	1.08556	ac5
NM_004839	HOMER2	0.02038	-0.44634	0.32410	0.50373	1.16732	0.61174	ac5
NM_172352	CD46	-0.05504	0.32360	0.52314	0.20590	1.16429	0.88579	ac5
NM_172353	CD46	0.26493	-0.10641	0.48396	-0.02955	1.16188	0.95893	ac5
NM_003344	UBE2H	0.03525	0.42973	0.70023	0.91358	1.16122	1.07559	ac5
NM_001025356	ANO6	0.52999	0.78969	1.06040	0.66408	1.15742	1.10748	ac5
NM_001242911	ZFAND6	0.36677	0.61052	0.81361	0.31010	1.15723	1.08586	ac5
NM_020397	CAMK1D	0.18634	-0.16560	0.18029	0.15311	1.15321	0.89497	ac5
NM_001198868	CAPN1	0.51169	0.17940	1.03351	0.35302	1.14798	1.06932	ac5
NM_001025235	TSPAN4	0.05765	-1.46012	-0.91831	-1.76219	1.14797	-1.01724	ac5
NM_033340	CASP7	0.28715	0.38193	0.54818	0.90457	1.14782	1.13432	ac5
NM_015601	HERC4	-0.19177	0.01778	0.12216	0.33605	1.14517	0.96993	ac5
NM_203403	LURAP1L	-0.05248	0.03985	-0.44519	0.01920	1.14396	1.02088	ac5
NM_003226	TFF3	0.58350	0.14356	0.65517	0.44535	1.13827	0.94893	ac5
NM_001160154	MGAT4A	-0.43664	0.13424	-0.11131	0.06822	1.13332	0.73465	ac5

NM_003406	YWHAZ	0.73153	0.29689	-0.02411	0.36486	1.12758	0.32373	ac5
NM_138396	42803	0.59064	-0.07787	-0.07853	-0.02980	1.12642	0.97717	ac5
NM_001127695	CTSA	-0.03121	0.18148	-0.21252	-0.34820	1.12462	0.89736	ac5
NM_001146082	MBOAT7	-0.39032	-0.06494	-0.17456	-0.96823	1.11581	0.92985	ac5
NM_002561	P2RX5	-0.21302	-1.51737	0.14548	-0.04699	1.11257	0.72571	ac5
NM_001141980	TP53BP1	0.54819	0.48632	-0.35306	0.39254	1.11220	1.02103	ac5
NR_028287	GABARAPL3	0.15795	0.72886	1.08085	0.68553	1.11142	0.95026	ac5
NM_001032384	PQBP1	1.05320	0.79110	0.13492	0.09589	1.10941	-0.04804	ac5
NM_021994	ZNF277	-0.25430	0.11948	0.28531	0.33647	1.10896	0.80715	ac5
NM_018110	DOK4	-0.13896	-0.07099	0.20640	0.29088	1.10858	1.08913	ac5
NM_014766	SCRN1	0.75099	0.24388	0.76152	0.44758	1.10458	0.81176	ac5
NM_139248	LIPH	0.05108	-0.03686	0.51094	0.66522	1.08975	1.06014	ac5
NM_001166693	AFF1	-0.09420	-0.09927	-0.72335	0.25074	1.08973	0.58381	ac5
NM_014326	DAPK2	0.09849	0.03188	0.62865	0.94312	1.08968	1.05162	ac5
NM_001040649	ACP1	-0.29661	-0.03025	0.23892	-0.31999	1.08785	0.66982	ac5
NM_001040098	MOSPD3	0.20296	0.40322	0.08120	0.10884	1.08639	0.85510	ac5
NM_005899	NBR1	0.42617	0.09425	-0.24229	-0.08417	1.08338	0.86974	ac5
NM_014646	LPIN2	-0.11392	0.00026	-0.23391	-0.08910	1.08258	0.86980	ac5
NM_016223	PACSIN3	-0.58929	-0.52843	-0.34598	-0.74437	1.07727	-0.37240	ac5
NM_012304	FBXL7	-0.18476	0.14316	0.36925	1.05311	1.07569	0.72289	ac5
NM_001244262	HBP1	-0.23841	-0.20193	0.17711	0.23547	1.07364	0.94598	ac5
NM_001198852	FOXJ3	-0.77982	0.41679	0.01357	0.43839	1.06658	-0.04862	ac5
NM_005534	IFNGR2	0.07427	0.02154	0.78281	0.89238	1.05904	1.04653	ac5
NM_032582	USP32	0.05359	0.36058	0.70525	0.77464	1.05737	0.80588	ac5
NM_004331	BNIP3L	-0.05871	0.28529	0.56795	0.89121	1.05384	0.87436	ac5
NM_032627	SSBP4	0.24265	0.04455	-0.07064	0.12442	1.05348	0.71033	ac5
NM_033394	TANC1	0.03706	-0.40956	0.12820	0.11380	1.05171	0.47683	ac5
NM_001144073	CHORDC1	0.25178	0.27148	-0.21012	0.83465	1.05113	0.79259	ac5
NM_022751	FAM59A	0.23746	-0.15211	0.75585	0.64276	1.04922	0.17446	ac5
NM_001252231	SEC24A	0.66093	0.37265	1.00585	0.76034	1.04677	0.68911	ac5
NR_026954	LOC90246	-0.26132	-0.36634	-0.55141	-0.48235	1.04675	0.79457	ac5
NM_015085	RAP1GAP2	0.04795	-0.69352	0.02217	0.23354	1.04517	0.83070	ac5
NM_005188	CBL	0.05590	0.31133	0.72914	0.56992	1.04297	0.74597	ac5
NM_138957	MAPK1	0.52278	0.16725	0.23528	0.02896	1.04126	0.93706	ac5
NM_018710	TMEM55A	-0.36200	-0.59703	0.09760	0.66277	1.03747	0.89789	ac5
NM_001624	AIM1	-0.10261	0.05520	0.64824	0.90864	1.03520	0.96348	ac5
NM_001166159	NDUFS2	-0.87135	-0.96788	0.71500	-0.02786	1.03518	0.07223	ac5
NM_001184985	WNK1	-0.02190	0.02174	0.25443	0.77164	1.03329	0.43077	ac5
NM_145320	OSBPL3	-0.08954	0.94497	0.77534	0.87875	1.02577	-0.37398	ac5
NM_001130145	YAP1	0.35714	0.10505	0.86270	0.79856	1.02497	0.71833	ac5
NM_001243403	CLEC16A	0.27605	0.10720	0.39462	0.66687	1.02426	0.76383	ac5
NM_001199216	MAG	0.05727	0.32275	1.01977	0.88054	1.02358	0.53760	ac5
NM_007110	TEP1	0.00353	0.10344	0.34043	0.26467	1.02317	0.99372	ac5
NR_045513	NT5C	-0.42056	-0.33951	0.20669	0.03750	1.02032	0.34645	ac5
NM_006165	NFRKB	0.53796	0.28413	0.86452	-0.11991	1.01610	0.79880	ac5
NM_001242831	ELOVL5	0.38957	-0.39542	0.04321	0.45101	1.01159	0.91978	ac5
NM_001530	HIF1A	-0.04463	0.11307	0.46957	0.11416	1.00652	0.81504	ac5

NM_006408	AGR2	0.03114	0.10766	0.48309	0.72985	1.00586	0.89064	ac5
NM_001197026	PLEKHA8	-0.13785	0.08998	0.24091	0.81864	1.00515	0.98673	ac5
NM_032167	SNX29	-0.03433	-0.12221	-0.26658	-0.45819	1.00269	0.93655	ac5
NM_000228	LAMB3	0.58963	1.18264	3.13238	3.94826	5.42877	5.98909	ac6
NM_002205	ITGA5	-0.13532	0.91129	2.36277	2.82640	5.13885	5.77630	ac6
NM_004994	MMP9	-0.30424	-0.04083	0.68423	1.31959	4.68248	5.66238	ac6
NM_000424	KRT5	0.58772	0.70022	2.02694	2.40154	3.92538	4.69680	ac6
NM_001450	FHL2	0.24508	1.03821	2.92425	3.10516	4.12521	4.60930	ac6
NM_001130040	SHC1	-0.06353	0.95270	1.69476	1.23592	3.91281	4.41280	ac6
NM_001085	SERPINA3	0.12647	-0.09082	1.19192	1.72616	3.33581	4.32892	ac6
NM_139314	ANGPTL4	0.43521	1.03631	3.02407	2.81909	3.85706	4.21950	ac6
NM_001323	CST6	0.54347	1.04322	1.96152	2.06357	3.38951	4.20190	ac6
NM_002004	FDPS	0.77091	0.46343	1.05108	0.40260	3.37467	4.17528	ac6
NM_001013837	MAD1L1	3.88847	3.80775	3.48730	2.99802	4.14664	4.17434	ac6
NM_001170718	BCAR1	0.28340	1.06829	2.03098	0.65935	2.80907	4.09715	ac6
NM_001143962	CAPN8	0.16598	0.26649	1.27882	2.09457	3.84000	4.07615	ac6
NM_005555	KRT6B	0.63169	0.97012	2.44008	2.63436	3.70427	4.03585	ac6
NM_003364	UPP1	-0.03392	1.44680	2.63644	3.02611	3.68213	4.02128	ac6
NM_005928	MFGE8	0.53689	0.66524	1.02178	1.73020	3.75334	3.95817	ac6
NM_000422	KRT17	2.55920	3.32065	3.89666	3.41423	3.74628	3.93458	ac6
NM_014417	BBC3	1.96844	2.60035	1.27700	0.62077	3.47912	3.91631	ac6
NM_002512	NME2	-1.21260	0.92839	1.80503	-0.75955	2.41247	3.90498	ac6
NM_003395	WNT9A	0.18434	2.29886	3.08732	2.67584	2.66397	3.74098	ac6
NM_001165413	SERPINE1	0.41608	0.85735	1.39528	1.38475	2.73410	3.72300	ac6
NM_198541	IGFL1	0.72414	0.97318	2.31467	2.86819	2.31683	3.61683	ac6
NM_019034	RHOF	0.23733	0.09131	1.48277	1.49605	3.08452	3.54919	ac6
NM_002962	S100A5	0.42606	0.68124	1.67239	2.08263	2.92997	3.49106	ac6
NM_004864	GDF15	1.02777	1.69776	1.65206	1.25115	3.25322	3.48316	ac6
NM_001039667	ANGPTL4	-0.99511	1.41651	3.09147	2.35267	2.98674	3.46423	ac6
NM_002659	PLAUR	0.59231	2.03851	2.92029	2.59018	3.16979	3.41985	ac6
NM_002970	SAT1	0.85097	1.32243	1.56850	1.75064	3.21714	3.39377	ac6
NM_003254	TIMP1	-0.19831	-0.05039	0.78799	1.62842	2.89613	3.35176	ac6
NM_002658	PLAU	1.89510	3.13889	2.96771	2.82271	2.87353	3.33612	ac6
NM_002543	OLR1	-0.08202	0.48377	0.53949	1.94234	2.90206	3.30681	ac6
NM_001039999	FAM83G	0.16885	0.95529	2.38031	2.17112	3.03255	3.28437	ac6
NM_005978	S100A2	0.56402	1.03245	2.07527	2.61789	2.77317	3.26875	ac6
NM_001456	FLNA	0.32638	0.31614	1.12551	0.90506	2.81696	3.21913	ac6
NM_002254	KIF3C	-0.04188	-0.21394	0.92846	0.81840	2.80873	3.19196	ac6
NM_134270	SMTN	0.72926	1.93710	2.51680	2.64445	2.73230	3.17849	ac6
NM_000201	ICAM1	0.53447	1.58072	2.77001	2.57744	2.96001	3.16609	ac6
NM_002960	S100A3	0.43369	0.88715	2.11399	2.38775	2.83631	3.15544	ac6
NM_001134368	SLC6A6	0.89983	1.91834	2.74607	2.81395	3.05784	3.15492	ac6
NM_001039775	AIM1L	-0.17930	-0.00228	0.73615	0.04819	2.22006	3.11553	ac6
NM_001126050	HDGF	-0.05657	0.44194	0.96748	2.00280	2.60809	3.07081	ac6
NM_001136485	C11orf86	0.05448	-0.14154	0.30585	-0.04594	2.73650	3.04507	ac6
NM_001114309	ELF3	0.90567	0.59549	0.02739	0.52475	2.44758	3.04357	ac6
NM_021727	FADS3	-0.01622	0.24872	1.06861	1.46426	2.30840	3.00521	ac6

NM_004714	DYRK1B	0.33828	0.36413	-0.54594	-0.57963	2.17949	2.99581	ac6
NM_001195532	SPTAN1	2.23922	1.35270	1.24083	0.87280	2.95524	2.99481	ac6
NM_018166	FAM176B	0.44737	0.32632	0.86453	0.88149	2.53823	2.97826	ac6
NM_020895	GRAMD1A	1.01379	1.14504	1.36555	1.52068	2.57969	2.97193	ac6
NM_001143906	TRAFD1	1.35095	1.60028	1.44135	1.02709	2.74042	2.95880	ac6
NM_000484	APP	0.44753	1.06920	2.07338	1.92674	2.85412	2.94326	ac6
NM_002318	LOXL2	0.18337	0.18931	1.14924	1.28494	2.59976	2.90660	ac6
NM_024527	ABHD8	0.36990	0.24687	1.14824	1.06171	2.53431	2.90648	ac6
NM_005195	CEBPD	1.35844	0.75040	0.14404	-0.18319	2.14043	2.89265	ac6
NM_016233	PADI3	-0.04021	-0.30329	0.11096	0.18782	2.53790	2.89206	ac6
NM_001216	CA9	0.08502	-0.20889	0.26822	0.56145	2.24039	2.87602	ac6
NM_003710	SPINT1	-0.68767	-0.80526	-0.07398	-0.29332	2.01743	2.85651	ac6
NM_001198616	MAP7	2.14884	2.29885	2.36770	2.29434	2.25766	2.84515	ac6
NM_007049	BTN2A1	1.68149	1.28635	2.17873	1.95623	2.61535	2.84455	ac6
NR_030767	ANKRD13D	-0.80337	0.61472	0.71945	-0.09954	2.42421	2.84197	ac6
NM_001127617	ALDOA	0.47934	-0.13284	2.66117	0.51328	0.04440	2.83840	ac6
NM_002204	ITGA3	-0.08661	0.15941	1.30178	1.48775	2.32796	2.82438	ac6
NM_001005376	PLAUR	0.47427	0.03733	1.08688	0.82250	2.39837	2.81946	ac6
NM_000725	CACNB3	-0.02847	-0.39138	0.57615	0.62111	2.58879	2.80931	ac6
NM_001540	HSPB1	0.41870	0.29682	0.37368	0.25380	2.25217	2.80517	ac6
NM_012429	SEC14L2	0.02850	0.62327	2.25989	2.63234	2.49311	2.79384	ac6
NM_133471_2	PPP1R18	0.37325	1.45635	1.80134	1.20215	2.52340	2.77395	ac6
NM_016352	CPA4	0.06550	0.23908	1.09670	2.07272	2.33404	2.77121	ac6
NM_001184940	C9orf25	0.83905	0.88214	0.94050	1.52717	1.75774	2.75951	ac6
NM_001130852	CBLC	0.05273	0.03410	0.79401	0.62041	2.08264	2.74916	ac6
NM_003979	GPRC5A	0.77025	1.89301	2.64446	2.41297	2.22718	2.74406	ac6
NM_014365	HSPB8	0.28767	0.94971	1.64265	1.33670	1.89818	2.73699	ac6
NM_007105	SLC22A18AS	0.27922	-0.01983	0.87291	1.58027	2.54107	2.73314	ac6
NM_203385	RNH1	-0.50237	2.50330	0.67884	2.19688	1.70975	2.72908	ac6
NM_006097	MYL9	1.16033	0.88161	1.44201	0.98602	1.87801	2.72079	ac6
NM_138768	MYEOV	0.02345	0.57311	1.77760	1.90384	2.35359	2.71283	ac6
NM_001252393	TNIP1	0.34889	-0.46194	0.97219	1.64948	2.01341	2.71024	ac6
NM_032899	FAM83A	-0.05071	-0.33383	0.21223	0.68194	1.94489	2.69841	ac6
NM_025182	FAM214B	0.11924	0.37842	0.27488	-0.02559	2.17703	2.69466	ac6
NM_005026	PIK3CD	0.08575	0.27356	1.68033	1.94944	2.62830	2.69207	ac6
NM_020210	SEMA4B	0.90712	0.18623	0.69145	0.88388	2.56836	2.68971	ac6
NM_001251	CD68	-0.15733	-0.06885	0.55052	0.92225	2.19347	2.68349	ac6
NM_003186	TAGLN	0.99386	1.81977	2.20313	2.00088	2.63489	2.67536	ac6
NM_001201552	ZNF821	-0.48006	-0.05850	-0.00314	0.08382	2.21144	2.66865	ac6
NM_001008661	CCBL2	-0.18172	2.58948	2.22253	1.83287	1.79635	2.66608	ac6
NM_002990	CCL22	-0.26216	0.44245	1.77221	2.25589	2.47558	2.66209	ac6
NM_005451	PDLIM7	0.23535	0.54343	1.45064	1.75102	2.24679	2.63042	ac6
NM_005115	MVP	0.28626	-0.37152	-0.56595	0.02637	1.99980	2.62886	ac6
NM_004838	HOMER3	0.48046	0.19611	0.52545	0.93238	2.04981	2.61929	ac6
NM_182565	FAM100B	0.14745	0.36005	0.15981	0.35449	2.20246	2.60739	ac6
NM_197955	C15orf48	0.13536	0.30791	0.84750	1.43008	2.30676	2.59464	ac6
NM_002966	S100A10	-0.09872	0.02203	0.90506	1.10822	2.09216	2.58036	ac6

NM_173086	KRT6C	0.53686	0.46560	1.24695	1.55829	2.23723	2.57109	ac6
NM_207006	FAM83A	0.01035	-0.08519	0.29328	0.72557	1.85008	2.56243	ac6
NM_001135190	ARAP1	0.94839	1.05930	-0.16940	-0.78251	1.93663	2.56225	ac6
NM_206833	CTXN1	0.18512	0.27079	1.10576	1.19062	2.42000	2.56205	ac6
NM_001197126	IRF3	-1.49719	1.25677	0.90629	0.53814	0.64225	2.53361	ac6
NM_001243756	PXN	-0.25914	0.27149	0.69148	0.86182	2.08143	2.53287	ac6
NM_002411	SCGB2A2	0.06757	-0.15835	0.08152	0.50387	2.16958	2.52670	ac6
NM_032525	TUBB6	0.01468	0.85810	2.28425	2.47045	2.25195	2.52406	ac6
NM_001145715	KPNA7	-0.23176	0.73606	1.33648	1.65487	2.30162	2.50298	ac6
NR_026644	UBAC2	-0.86089	-0.64915	0.61142	-0.02991	1.99228	2.49813	ac6
NM_012129	CLDN12	0.81750	0.71675	1.22848	1.94293	1.35062	2.48253	ac6
NM_153832	GPR161	0.00623	0.13695	1.74254	1.71558	2.21068	2.48120	ac6
NM_001243156	TAF1C	1.49780	0.17687	2.06862	1.87752	1.96832	2.47639	ac6
NM_006000	TUBA4A	0.38669	0.48973	0.74350	0.50609	1.87652	2.47523	ac6
NM_175884	CCDC71L	0.78123	1.56073	1.75895	1.71077	1.86272	2.46666	ac6
NM_198252	GSN	1.10663	-0.13965	0.81722	-0.35260	2.43290	2.45829	ac6
NM_022119	PRSS22	0.26549	0.86428	1.24943	0.86795	1.88949	2.45573	ac6
NM_014945	ABLIM3	0.02606	-0.08178	0.45631	1.03935	2.13074	2.45565	ac6
NM_001004128	QSOX1	0.03627	-0.07556	0.51274	0.78310	1.79132	2.45449	ac6
NM_145236	B3GNT7	0.03065	-0.31688	-0.27735	-0.06927	2.38275	2.45191	ac6
NM_017894	ZSCAN2	-0.07185	1.02022	0.05555	1.10786	1.22821	2.45124	ac6
NM_001005909	IP6K2	1.54680	1.89703	1.55220	1.47995	2.14604	2.44572	ac6
NR_024479	LOC10013172	-0.17603	-0.19158	0.37590	0.85519	1.76104	2.44300	ac6
NM_007246	KLHL2	1.41941	1.11585	0.23868	0.39493	2.36342	2.43888	ac6
NM_032488	CNFN	0.73266	0.21664	0.60341	1.05877	1.85603	2.43068	ac6
NM_012232	PTRF	-0.11381	1.09067	2.24651	2.14251	2.06460	2.43033	ac6
NM_001042610	DBNDD1	0.42705	0.39300	0.73769	1.24240	2.24161	2.42852	ac6
NM_001256662	TEAD2	0.27812	-0.43066	-0.08697	0.31898	2.19669	2.42826	ac6
NM_003311	PHLDA2	0.94100	1.71707	2.29499	2.25896	2.20803	2.42236	ac6
NM_021077	NMB	-0.01744	0.11506	0.93872	0.10912	2.14791	2.41526	ac6
NM_012257	HBP1	0.73090	1.18248	-0.93305	0.41085	2.28442	2.38663	ac6
NM_198580	SLC27A1	0.44800	0.16213	0.31633	0.34005	2.22128	2.38429	ac6
NM_005619	RTN2	0.18997	0.08068	0.98138	-0.06613	1.96345	2.38214	ac6
NM_005268	GJB5	0.87354	0.45582	1.54162	1.72772	2.01061	2.37909	ac6
NM_145899	HMGA1	0.08403	-0.20387	0.37222	0.58519	1.81765	2.36922	ac6
NM_032108	SEMA6B	-0.41626	-0.44580	-0.02752	-0.35483	2.27550	2.36130	ac6
NM_020982	CLDN9	0.24191	0.55162	1.58685	1.97581	2.08503	2.35883	ac6
NR_028406	FXYD5	0.24234	0.44993	0.71059	0.66722	1.53403	2.35782	ac6
NM_003043	SLC6A6	0.00704	-0.05048	0.77776	1.79438	2.30479	2.34764	ac6
NM_005141	FGB	0.07174	0.25339	-0.18524	0.47633	1.59043	2.34117	ac6
NR_003063	TUBA4B	0.63807	0.57230	0.78698	0.57917	1.93966	2.33520	ac6
NM_174894	TRAPPC5	0.32862	0.61412	1.13526	1.47157	2.27730	2.33360	ac6
NM_000935	PLOD2	0.01898	1.17262	0.96135	1.42115	2.20096	2.33045	ac6
NM_001128225	SLC39A13	1.87676	0.87972	0.58512	0.77162	1.15353	2.32188	ac6
NM_001135770	PVR	0.40504	0.98667	1.05821	0.85005	1.91970	2.31942	ac6
NM_003744	NUMB	1.87484	1.87602	2.26308	1.86060	1.36078	2.31422	ac6
NM_005817	PLIN3	0.01319	-0.05618	0.57424	0.54387	1.90438	2.31401	ac6

NM_213636	PDLIM7	0.30601	-0.04606	0.91541	1.14772	1.95944	2.30924	ac6
NM_001953	TYMP	0.63932	0.43308	0.47169	0.82500	1.70325	2.30265	ac6
NM_031286	SH3BGRL3	0.14070	-0.06446	0.27157	0.71816	1.64542	2.30101	ac6
NM_032515	BOK	-0.00364	0.88802	2.10972	2.05590	2.22025	2.29722	ac6
NM_145903	HMGA1	0.24480	0.17018	0.45413	0.44322	1.92970	2.29168	ac6
NM_001136017	CCND3	-0.62583	-0.80645	1.09083	1.00257	2.05418	2.28789	ac6
NM_018306	TMEM40	0.32970	1.16002	2.01970	1.80138	1.85534	2.28631	ac6
NM_024866	ADM2	1.43759	1.18304	1.07783	-0.08277	1.57501	2.28626	ac6
NM_001195071	TMEM184B	1.55337	1.95422	1.86771	1.66917	2.28293	2.28493	ac6
NM_002256	KISS1	0.11449	0.34538	0.76948	1.01697	1.70201	2.27908	ac6
NM_021149	COTL1	-0.05335	-0.01565	0.81127	0.95878	1.73180	2.27738	ac6
NM_172208_4	TAPBP	0.14337	1.50121	1.17506	1.12122	2.06777	2.26526	ac6
NM_001172896	CAV1	-0.17077	-0.92940	1.09752	0.37977	1.73837	2.26075	ac6
NM_005357	LIPE	0.35191	0.81251	0.16559	-0.13514	1.77112	2.25493	ac6
NM_000888	ITGB6	-0.00923	0.82945	1.22828	1.47114	2.13296	2.24309	ac6
NM_001145773	GPR56	-0.39580	-1.35538	0.53664	0.35007	0.89880	2.21360	ac6
NM_004566	PFKFB3	0.63867	1.08168	2.10246	1.84753	2.16209	2.20536	ac6
NM_001199954	ACTG1	0.48691	0.62860	1.68994	1.81901	1.57047	2.20386	ac6
NR_037719	C17orf61-PLS(	-0.29690	-0.89293	0.10218	0.09787	1.54496	2.19181	ac6
NM_016354	SLCO4A1	0.15696	0.54511	1.16968	1.26829	1.93971	2.18563	ac6
NM_001199	BMP1	-0.11967	-0.67680	0.45101	0.41207	1.59937	2.18233	ac6
NM_001242443	GRB7	-0.14895	0.71519	0.54070	0.12324	1.63601	2.17991	ac6
NM_001030002	GRB7	0.19606	0.52650	0.31478	-0.07021	1.95452	2.17842	ac6
NM_002314	LIMK1	-0.23997	-0.36005	0.35019	0.12168	1.58535	2.17507	ac6
NM_006736	DNAJB2	0.26803	0.75309	1.05910	0.33086	1.53715	2.15898	ac6
NM_002447	MST1R	0.05094	-0.14569	0.72299	0.69728	1.57906	2.15035	ac6
NM_001040197	AGTRAP	0.34216	-0.09609	0.07903	1.01372	1.24545	2.13543	ac6
NM_001136199	GRAMD1A	-0.04420	-0.11931	0.20342	-0.48607	1.84736	2.13389	ac6
NR_037688	ACTG1	1.09163	1.23495	1.47787	0.65817	2.00875	2.12889	ac6
NM_001253792	ZNF444	-0.33388	0.48292	-0.57604	-1.91948	1.80534	2.12158	ac6
NM_001135999	RUSC2	0.30775	1.88379	1.05644	0.01290	1.84119	2.11941	ac6
NM_001037125	UNKL	0.12866	-0.20622	-0.50934	-0.01242	1.94419	2.11908	ac6
NR_004380	SNORD104	1.64235	0.88459	1.34010	1.64204	1.51106	2.11277	ac6
NM_147782	CTSB	0.23949	0.92685	0.39081	1.67140	1.93301	2.11139	ac6
NM_033445	HIST3H2A	0.53252	0.26681	0.61703	0.90319	1.47759	2.09859	ac6
NM_004039	ANXA2	0.11700	0.33317	0.91824	1.34621	1.73454	2.09467	ac6
NM_001195200	CCDC107	0.31782	0.90549	1.24055	1.59678	0.00054	2.09155	ac6
NM_033008	PCBP4	0.52053	1.07849	1.08104	1.68763	1.08211	2.09112	ac6
NM_020888	KIAA1522	0.21439	0.10451	0.46274	-0.34495	1.56806	2.08777	ac6
NM_001142569	C1orf106	-0.34739	-0.34013	0.06007	-0.32570	1.74096	2.07916	ac6
NM_201649	SLC6A9	0.04395	0.63649	1.30034	0.76329	1.11399	2.07356	ac6
NM_001244888	AGAP1	1.43634	0.38780	1.39662	1.55034	1.97782	2.06576	ac6
NM_001075098	MOCS1	0.91034	0.55216	0.49349	1.29794	1.42385	2.06252	ac6
NM_014601	EHD2	0.05799	-0.22637	0.11185	-0.05073	1.80081	2.05894	ac6
NR_003942	SNORD76	0.11843	0.66671	1.66828	0.10344	1.10280	2.05737	ac6
NM_145901	HMGA1	-0.51509	-0.47626	0.22566	0.38740	1.47942	2.05349	ac6
NM_006383	CIB2	0.03081	-0.50703	-0.23142	0.00582	1.89076	2.05065	ac6

NM_033449	FCHSD1	0.03791	0.15059	0.95066	0.80934	1.69809	2.05013	ac6
NM_022842	CDCP1	-0.16690	-0.48255	0.72922	1.17663	1.73499	2.04879	ac6
NM_001795	CDH5	0.24606	1.14299	1.94177	1.94983	1.64695	2.04876	ac6
NM_014888	FAM3C	-1.24621	-0.01409	1.10694	1.04616	1.76266	2.04138	ac6
NM_001010917	GOLGA7B	0.03054	-0.09490	0.41915	0.55472	1.51767	2.03682	ac6
NM_198390	CMIP	0.18073	0.33186	1.52841	1.03490	1.80422	2.02164	ac6
NM_152672	OSTalpha	0.18548	-0.06789	0.06158	0.13910	1.51564	2.01963	ac6
NR_002968	SNORA34	0.78203	1.36953	1.12360	1.85724	0.75558	2.01879	ac6
NM_003612	SEMA7A	0.34740	1.83361	1.89083	0.51349	1.87146	2.01524	ac6
NM_019100	DMAP1	0.48770	0.54310	1.08896	0.66013	1.04522	2.00879	ac6
NM_182507	KRT80	0.42831	0.90764	1.50154	0.96534	1.67452	2.00562	ac6
NM_001242775	TMEM139	1.48327	-0.37551	0.99462	1.25863	0.43036	1.99983	ac6
NM_206900	RTN2	-0.62664	0.09090	-0.28533	0.26527	1.01695	1.99239	ac6
NM_023072	ZSWIM4	0.27609	0.94251	1.13615	0.37752	1.82390	1.98885	ac6
NM_133376	ITGB1	0.32994	-0.06063	0.88802	0.51050	0.84149	1.98858	ac6
NM_001145721	HOMER3	-0.31307	-0.39669	-0.40387	-0.88541	1.45518	1.97919	ac6
NM_000202	IDS	0.44560	0.66802	0.87664	1.54075	1.80991	1.97340	ac6
NM_003244	TGIF1	1.57401	1.18692	0.99345	0.87827	1.66737	1.96961	ac6
NM_182623	FAM131C	0.36441	-0.18798	0.37416	0.12481	1.30178	1.96241	ac6
NM_006084	IRF9	0.40484	0.53109	0.79119	0.59079	1.80628	1.95795	ac6
NM_003811	TNFSF9	1.49565	1.60453	0.12393	-0.23515	1.41060	1.95746	ac6
NM_002826	QSOX1	-0.03392	-0.04807	0.29886	0.37505	1.14353	1.95734	ac6
NM_015589	SAMD4A	0.11062	0.78331	1.69343	1.76277	1.83583	1.95447	ac6
NM_181746	CERS2	-0.42289	-1.25115	0.38526	0.66006	1.29733	1.95148	ac6
NM_001242409	FAM59A	-0.35231	0.88408	0.15138	0.06097	1.65431	1.94870	ac6
NM_001120	MFSD10	0.81393	0.64523	1.13744	0.88322	1.31524	1.94782	ac6
NM_006373	VAT1	-0.02256	-0.26979	0.04321	0.04657	1.56698	1.94484	ac6
NM_001958	EEF1A2	0.02819	-0.08388	0.12135	-0.17409	1.78707	1.94127	ac6
NM_022164	TINAGL1	0.23437	0.28322	0.77783	0.74622	1.45137	1.94005	ac6
NM_207368	FAM195B	0.48197	0.05764	0.36091	0.48250	1.49260	1.93182	ac6
NM_015610	WIPI2	1.50164	1.29423	0.68299	0.84760	1.19509	1.92932	ac6
NM_177417	KLC3	0.08722	0.08520	0.71602	0.59760	1.40226	1.92609	ac6
NM_001024736	CD276	-0.01255	0.16971	1.29946	1.15099	1.54890	1.92518	ac6
NR_033257	XAGE1E	0.14574	0.47114	0.29628	0.55863	0.91984	1.92443	ac6
NM_016526	BET1L	1.44079	1.11881	1.43412	1.76451	0.93377	1.92107	ac6
NM_021259	TMEM8A	0.09533	0.15046	0.50507	0.40604	1.57729	1.91932	ac6
NM_001834	CLTB	0.06835	0.09231	0.52196	0.62039	1.27632	1.91440	ac6
NM_152468	TMC8	0.19042	0.49739	1.21807	0.88476	1.36077	1.90896	ac6
NM_001243249	NPRL3	0.57828	0.81256	0.44889	0.67710	1.19698	1.90033	ac6
NM_001305	CLDN4	0.48608	0.99486	0.68022	0.29225	1.46303	1.89923	ac6
NM_012116	CBLC	0.18870	-0.04037	0.15460	0.19184	1.55928	1.89375	ac6
NM_005629	SLC6A8	0.32225	0.29736	0.44620	0.21021	1.01995	1.88478	ac6
NM_144606	FLCN	0.09431	0.36615	0.55792	0.36203	1.59468	1.88344	ac6
NM_182532	TMEM61	0.71301	-0.08893	0.13054	1.02615	1.38560	1.88014	ac6
NR_003573	ANXA2P2	0.07914	0.08424	0.84990	1.12331	1.62297	1.87730	ac6
NM_001164603	ASXL1	0.31020	0.59100	0.87179	1.03374	1.30494	1.87716	ac6
NR_001446	ANXA2P3	0.20301	0.11691	0.85843	1.07708	1.63217	1.87238	ac6

NM_005310	GRB7	0.01527	-0.11430	-0.25375	-0.00717	1.50190	1.87144	ac6
NM_014238	KSR1	0.06032	-0.14183	0.54374	0.59989	1.80444	1.86738	ac6
NM_001253881	MED27	0.37185	0.78302	-0.35257	1.65371	1.46479	1.86660	ac6
NM_145689	APBB1	-0.15995	-0.36069	-0.03957	0.29214	1.61844	1.86610	ac6
NM_001079880	PRKD2	-0.30483	-0.47290	-0.37738	-0.52464	1.56954	1.85826	ac6
NM_032575	GLIS2	0.35059	1.03362	1.22694	0.39220	1.54012	1.84542	ac6
NM_030935	TSC22D4	0.02555	0.15510	0.18708	-0.27883	1.43347	1.84515	ac6
NM_006877	GMPR	0.50589	-0.13298	0.77829	1.29260	1.77302	1.84013	ac6
NM_005514	HLA-B	0.17300	0.27133	0.62111	0.51725	1.42521	1.83649	ac6
NM_015379	BRI3	0.06940	0.01005	0.21378	-0.00930	1.24421	1.83553	ac6
NM_202470	GIPC1	0.20219	0.30293	0.29201	0.02284	1.32170	1.83506	ac6
NM_002842	PTPRH	0.30626	0.28038	1.01363	1.20762	1.45673	1.83448	ac6
NM_001206952	SLC16A3	0.11926	0.63576	0.72662	0.09036	1.51887	1.83413	ac6
NM_031450	C11orf68	0.91116	1.31718	1.34383	1.47157	1.63046	1.83306	ac6
NM_145109	MAP2K3	0.19070	0.17673	1.13561	1.26638	1.51201	1.83211	ac6
NM_024954	UBTD1	0.23166	0.05480	0.39855	0.18635	1.38280	1.82830	ac6
NM_003712	PPAP2C	0.69835	-0.51305	0.09097	0.63177	1.64024	1.82731	ac6
NM_004037	AMPD2	-0.12234	0.12888	0.41306	0.12818	1.42149	1.82569	ac6
NM_006314	CNKSR1	0.10428	-0.84481	-0.37689	-2.47103	-0.22041	1.82165	ac6
NM_138410	CMTM7	1.03370	0.58172	0.21687	0.09699	0.61756	1.81998	ac6
NM_013399	C16orf5	0.99517	1.18013	1.72912	1.05895	1.37762	1.81789	ac6
NM_001127198	TMC6	0.03811	-0.11393	0.60107	-0.10249	1.54716	1.81660	ac6
NM_001146310	C1orf86	0.25809	0.51012	0.57060	0.05441	1.36977	1.81247	ac6
NM_006747	SIPA1	0.13404	0.21237	0.09270	-0.27651	1.42902	1.80706	ac6
NM_020812	DOCK6	-0.21662	-0.17945	0.45351	0.45340	1.65873	1.80559	ac6
NM_018089	ANKZF1	1.17247	0.60786	0.77130	1.63449	1.75786	1.80520	ac6
NM_001037494	DYNLL1	0.03510	0.08217	0.36229	0.11938	0.72955	1.80449	ac6
NR_001562	ANXA2P1	0.04797	-0.07696	0.75109	1.02927	1.60476	1.80247	ac6
NM_003338	UBE2D1	-0.06259	-0.27727	1.12748	1.74878	1.61655	1.80036	ac6
NM_001145054	C2orf81	0.08172	0.02094	0.21518	0.19123	1.33842	1.79696	ac6
NM_003565	ULK1	0.05224	0.18204	0.36121	-0.33205	1.39952	1.79563	ac6
NM_001146345	PQLC1	0.56502	0.70606	1.53231	0.45883	1.51509	1.79031	ac6
NM_001204089	C1orf151-NBL	0.19332	0.10204	0.64050	0.46198	0.12702	1.78963	ac6
NM_032038	SPNS1	0.42313	-0.14488	0.60344	0.76469	1.01179	1.78468	ac6
NR_027064	PLAC2	-0.12706	-0.26792	0.01499	0.02726	1.69552	1.78098	ac6
NM_001113492	42987	0.05134	-0.24575	0.16044	-0.22825	1.26186	1.77508	ac6
NM_022778	CEP85	0.36900	0.80603	1.13090	0.73553	1.38350	1.77149	ac6
NR_003594_3	REXO1L2P	0.24823	0.77282	0.72755	1.08038	1.48850	1.77045	ac6
NM_181864	ACOT7	0.44010	0.73438	-0.02727	0.61128	1.36203	1.76667	ac6
NM_004207	SLC16A3	-0.11898	-0.16810	0.28153	0.53550	1.34879	1.76655	ac6
NM_032412	CYSTM1	0.20693	0.04910	-0.15391	0.04849	1.58624	1.76357	ac6
NM_003530	HIST1H3D	0.35992	-0.40261	-0.15694	0.06413	1.46573	1.76260	ac6
NM_001145064	GATSL2	0.44509	-0.04529	-0.08709	-0.05186	1.50229	1.76143	ac6
NM_018113	LMBR1L	-0.14647	-0.19023	0.14897	0.21243	1.46930	1.76087	ac6
NM_001035254	FAM102A	0.20571	0.29912	0.99878	0.80985	1.50200	1.75911	ac6
NM_004395	DBN1	0.09498	0.06839	0.42492	-0.05257	1.36813	1.75748	ac6
NM_001348	DAPK3	0.59637	1.30071	1.57075	0.97883	1.31113	1.75532	ac6

NM_001039920	ZNF384	0.33062	0.56969	1.36003	0.39379	0.99938	1.75366	ac6
NM_202494	GIPC1	0.26280	0.33887	0.52222	0.25221	1.15901	1.74997	ac6
NM_001035534	FAM129B	-0.13493	0.11384	0.30876	0.25744	1.12778	1.74724	ac6
NM_172390	NFATC1	-0.19737	0.06338	-0.14966	-0.43586	1.47694	1.74694	ac6
NM_022833	FAM129B	0.12112	-0.20060	0.38621	0.25053	1.24794	1.74649	ac6
NM_002305	LGALS1	0.19043	-0.35793	-0.00677	0.61948	1.00736	1.74431	ac6
NM_003900	SQSTM1	0.32991	0.63845	0.94683	0.82477	1.24973	1.74280	ac6
NM_033409	C20orf54	-0.20048	-0.35046	-0.06294	-0.12493	1.15852	1.74224	ac6
NM_003370	VASP	0.09656	0.60912	1.12026	0.70146	1.31295	1.73022	ac6
NM_001110556	FLNA	-0.05421	0.19091	0.64722	0.45475	1.09243	1.72900	ac6
NM_001196	BID	-1.37146	-0.00272	0.88780	0.65525	1.06494	1.72373	ac6
NM_198883	MTX1	0.71111	0.96837	1.19421	0.46240	1.21671	1.72193	ac6
NR_002454	LOC606724	0.05845	0.18355	0.89097	0.69334	1.23455	1.71829	ac6
NM_005860	FSTL3	0.15659	0.60958	1.19302	0.90731	1.37163	1.71581	ac6
NM_006142	SFN	0.05723	0.78760	1.60691	1.35981	1.37668	1.71418	ac6
NM_014624	S100A6	0.16682	-0.02410	0.30682	0.50314	1.19874	1.71081	ac6
NM_002953	RPS6KA1	0.32730	0.47002	0.66308	0.50507	1.53285	1.70638	ac6
NM_005194	CEBPB	0.21748	0.37203	0.38283	-0.04523	1.45916	1.70637	ac6
NM_000088	COL1A1	-0.12071	0.71929	0.82345	0.66264	1.49945	1.70566	ac6
NM_001164840	LYRM4	-0.05304	0.40273	0.75757	0.76508	0.39545	1.70468	ac6
NM_001098531	RAPGEF3	0.21132	0.57775	0.45468	0.39566	1.69698	1.70175	ac6
NM_001098416	HDAC7	-0.31622	0.39443	0.55520	0.49195	0.80379	1.69977	ac6
NM_001145524	YPEL3	0.15123	-0.18521	-0.27228	-0.15305	1.50814	1.69871	ac6
NM_005490	SH2D3A	0.02261	0.19521	0.77618	0.64650	1.21431	1.69769	ac6
NM_001011722	ARHGEF10L	-0.08515	-0.20608	-0.24541	-0.05949	1.39325	1.69643	ac6
NM_012326	MAPRE3	-0.02122	0.00915	0.02209	-0.09649	1.41398	1.69642	ac6
NR_003594	REXO1L2P	0.21645	0.69770	0.94604	0.96526	1.32580	1.68990	ac6
NM_025092	ATHL1	0.28928	0.73770	0.79578	0.47939	1.35660	1.68970	ac6
NM_153840	GPR110	-0.13458	0.05470	0.49367	0.75497	1.57878	1.68920	ac6
NM_203434	IER5L	0.63873	0.43616	0.34683	0.44273	1.43233	1.68844	ac6
NM_001145862	MTMR11	0.15416	-0.58132	0.19404	-0.20379	1.24380	1.68817	ac6
NM_001134870_	PPP1R18	0.08528	0.93918	1.12548	0.53815	1.34408	1.68617	ac6
NM_006270	RRAS	0.30241	0.02661	0.25447	0.27935	1.11690	1.68162	ac6
NM_004421	DVL1	0.05695	0.17365	0.46796	0.33039	1.19673	1.67834	ac6
NM_016525	UBAP1	0.48147	0.87019	0.82726	0.35517	1.58802	1.67752	ac6
NM_024832	RIN3	0.09939	-0.09722	-0.34046	0.21394	1.36604	1.67620	ac6
NM_001081492	KRT80	0.22886	0.54236	0.71722	0.55767	1.43716	1.67571	ac6
NR_030407	MIR671	-0.45787	0.64998	0.87047	0.08643	-0.30231	1.66955	ac6
NR_003594_4	REXO1L2P	0.17538	0.44208	0.78944	0.92643	1.22112	1.66891	ac6
NM_001243333	SEZ6L2	-0.94260	0.11001	-0.44112	0.38174	0.81156	1.66852	ac6
NM_001005915	ERBB3	0.17925	-0.17112	0.06122	0.41304	1.01523	1.66548	ac6
NR_026857	FLJ90757	-0.17100	-0.02252	0.02912	0.48325	1.48688	1.65856	ac6
NM_001204415	TINAGL1	-0.20837	0.04420	0.49580	0.14327	0.85219	1.65778	ac6
NM_001109974	SYNPO	-0.22794	-0.08188	0.34147	0.10032	1.29736	1.65705	ac6
NM_001145278	NECAP2	0.24787	0.12216	0.03852	-0.50286	1.30735	1.65390	ac6
NM_198275	MPZL3	-0.01081	0.70422	0.97809	0.93891	1.61919	1.65247	ac6
NM_020350	AGTRAP	-0.41735	-0.79851	-0.63861	0.36947	0.96285	1.65206	ac6

NM_001005731	ITGB4	0.02904	-0.00424	-0.11567	-0.22719	1.24352	1.64991	ac6
NM_134269	SMTN	0.14237	0.44812	1.02652	0.57321	1.39713	1.64982	ac6
NR_003022	SNORA59B	1.18939	-0.05966	0.12124	-0.34161	1.29773	1.64972	ac6
NM_001144888	BAIAP2	-0.43201	-0.61443	-0.27458	-0.06258	1.20528	1.64541	ac6
NM_001042678	RHOC	0.22744	-0.01521	0.39651	0.88792	1.26938	1.64435	ac6
NM_001206802	TP53I3	0.84000	-0.02808	0.31846	0.46744	1.07386	1.64292	ac6
NM_001024212	S100A13	0.95712	0.40056	0.12747	-0.31602	0.76497	1.64033	ac6
NM_001242825	FDPS	0.53080	0.71854	1.03807	1.22407	1.51693	1.63864	ac6
NM_001002020	PUS1	0.41267	1.27990	1.54868	0.08134	1.03718	1.63732	ac6
NM_019594	LRRC8A	1.13185	0.93128	0.75952	0.33422	1.43159	1.63550	ac6
NM_001007072	ZSCAN2	0.74247	-0.56049	0.31385	-0.10137	1.08336	1.63350	ac6
NM_177533	NUDT14	0.50352	0.24041	0.58920	0.23433	0.97846	1.63020	ac6
NM_032323	TMEM79	0.13644	0.01643	1.22975	1.48787	1.44479	1.62992	ac6
NM_004368	CNN2	0.46784	0.49745	0.66977	0.14477	1.22783	1.62975	ac6
NM_181652	PRDX5	0.08202	-0.96433	-0.09366	-0.17184	1.35727	1.62895	ac6
NM_014901	RNF44	0.05207	0.12430	0.15004	0.09116	1.24401	1.62314	ac6
NM_005224	ARID3A	0.14670	0.14826	0.14432	-0.19512	1.19034	1.61576	ac6
NM_173546	KLHDC8B	-0.23288	-0.20988	-0.66749	-0.70974	1.28792	1.61520	ac6
NM_134421	HPCAL1	-0.25198	-0.18865	0.62643	0.77154	1.21054	1.61375	ac6
NM_021229	NTN4	-0.01259	0.55031	0.19277	-0.02692	1.49704	1.61062	ac6
NM_003839	TNFRSF11A	0.34843	-0.00146	1.06465	1.37551	1.54419	1.60883	ac6
NM_000801	FKBP1A	-0.09755	-0.13376	0.42597	0.75479	1.05005	1.60560	ac6
NM_001178110	ZNF185	0.43435	-0.21063	0.30559	0.66210	1.33324	1.60523	ac6
NM_012286	MORF4L2	-0.13564	0.82696	0.54331	-1.34365	1.40464	1.60493	ac6
NM_005429	VEGFC	0.06500	0.29233	0.72119	1.21434	1.51191	1.60358	ac6
NM_001242442	GRB7	0.62818	0.88864	1.03874	0.64277	1.51332	1.60259	ac6
NM_006123	IDS	0.21972	-0.02877	0.45810	0.61753	1.24037	1.60259	ac6
NM_001126131	POLG	0.92461	1.38225	1.40145	1.06136	1.13777	1.60246	ac6
NM_001040118	ARAP1	0.43537	0.54704	-0.01302	0.23141	1.52982	1.59751	ac6
NM_138435	FAM83F	-0.33343	-0.29982	0.52605	0.64468	1.27291	1.59599	ac6
NM_002569	FURIN	-0.07735	0.03333	0.48739	0.48144	1.22550	1.59590	ac6
NM_019096	GTPBP2	-0.01157	0.06145	0.57025	0.33921	1.41744	1.59417	ac6
NM_002773	PRSS8	-0.00840	-0.14478	-0.05699	0.01029	1.21797	1.59301	ac6
NM_019050	USP53	-0.58742	0.90483	1.26276	1.21119	1.43109	1.59258	ac6
NM_152267	RNF185	0.96489	1.29519	0.67359	0.14069	1.49398	1.59245	ac6
NR_026690	ABHD11-AS1	-0.06458	-0.53096	-0.55395	-0.04409	0.90916	1.59236	ac6
NM_174869	IDH3G	0.78529	0.96881	0.58202	0.35273	1.55255	1.59074	ac6
NM_018957	SH3BP1	0.01639	-0.10467	0.43510	0.42114	1.48021	1.58934	ac6
NM_001178107	ZNF185	1.15946	-0.16992	0.32506	-0.43738	0.47616	1.58729	ac6
NM_004886	APBA3	0.24753	0.15672	0.06278	-0.27147	1.11192	1.58702	ac6
NM_138423	CASC4	0.00321	0.17715	-0.00179	-0.34037	0.96630	1.58369	ac6
NM_001031722_	ATAT1	-0.04521	0.23125	-0.02436	-0.32115	1.30721	1.58350	ac6
NM_003881	WISP2	0.14330	-0.00491	-0.13186	0.49495	1.54006	1.58345	ac6
NM_153247	SLC29A4	0.19186	-0.04918	0.03660	-0.12255	0.09781	1.58256	ac6
NM_021173	POLD4	0.18243	-0.07454	0.12683	0.36021	1.18504	1.58169	ac6
NM_001706	BCL6	0.33803	-1.48761	0.29230	-0.23343	1.55802	1.57964	ac6
NM_000435	NOTCH3	-0.03304	-0.01027	0.16445	-0.17753	1.22950	1.57703	ac6

NM_001986	ETV4	-0.33460	-0.19720	0.31676	0.63514	1.23325	1.57514	ac6
NM_020524	PBXIP1	-0.14235	-0.29061	-0.26104	-0.59481	1.36926	1.57503	ac6
NM_022822	KLC2	-0.21964	0.68608	-0.19660	0.61962	1.41494	1.57347	ac6
NM_001288_2	CLIC1	0.01822	0.01777	0.39512	0.45666	1.20217	1.57323	ac6
NM_001012727	AGPAT2	0.46750	-0.08682	-1.31167	0.29873	1.21459	1.57228	ac6
NM_001164475	SLC41A3	0.28885	0.53642	0.55634	0.81271	-0.00878	1.57193	ac6
NM_001204519	P2RX5	-0.52778	-0.58966	0.28578	-0.66789	-0.21293	1.56981	ac6
NM_015219	EXOC7	-0.48052	0.22343	-0.48767	-1.21306	1.46004	1.56869	ac6
NM_001784	CD97	-0.23050	-0.21294	0.43887	0.38920	1.17449	1.56733	ac6
NM_001198773	PI4KB	0.27692	0.24400	0.51398	0.26372	1.55642	1.56641	ac6
NM_002116_4	HLA-A	-0.04855	0.07565	0.33347	0.41434	1.23483	1.56330	ac6
NM_024698	SLC25A22	-0.37774	0.50636	0.89484	0.89815	0.57541	1.56307	ac6
NM_139161	CRB3	0.48698	0.46655	0.69984	0.16254	1.20154	1.55316	ac6
NM_001144070	ABCC3	-0.12577	-0.43279	0.08335	0.72228	1.35317	1.55289	ac6
NM_033239	PML	0.64613	-0.46067	0.27001	-0.16654	0.97527	1.55237	ac6
NM_001243177	ALDOA	0.14732	-0.12652	0.28010	0.37750	1.10807	1.55233	ac6
NM_184041	ALDOA	0.01614	-0.27434	0.16467	0.37871	0.98471	1.55015	ac6
NM_198799	BCAS4	0.10269	-0.11449	0.26096	0.70822	1.31460	1.54856	ac6
NM_033274	ADAM19	-0.04145	-0.05622	0.76326	0.52783	1.27869	1.54720	ac6
NM_009587	LGALS9	-0.39957	-0.46994	0.82505	1.08701	1.05325	1.54243	ac6
NM_202001	ERCC1	0.27460	-0.00499	0.27310	0.57239	1.16107	1.54161	ac6
NM_001252390	TNIP1	0.03539	-0.00522	0.41439	0.49887	1.08854	1.53810	ac6
NM_144779	FXYD5	-0.79710	-0.49884	-0.26194	0.56385	0.69791	1.53691	ac6
NM_018275	C7orf43	0.43232	0.67236	0.79005	0.62684	1.15400	1.53612	ac6
NM_174881	CRB3	0.31528	0.30637	0.32239	0.55775	1.09205	1.53605	ac6
NR_003594_2	REXO1L2P	-0.27639	0.74874	1.15306	1.04528	1.46439	1.53597	ac6
NM_024636	STEAP4	0.15306	0.26094	0.31856	-2.30177	1.49255	1.53457	ac6
NM_001242821	DEF8	0.09431	-0.06706	-0.05788	0.00252	1.04574	1.53122	ac6
NM_001031690	FAM131B	0.20255	-0.36400	0.41511	1.08883	0.89188	1.53017	ac6
NM_182919	TICAM1	0.46436	1.24544	0.85366	0.23113	1.06994	1.52787	ac6
NM_017607	PPP1R12C	0.01267	0.07796	0.04611	-0.13665	1.07268	1.52702	ac6
NM_017946	FKBP14	-0.03610	0.52913	0.57507	0.91467	1.32670	1.52653	ac6
NM_173626	SLC26A11	0.21223	0.17658	-0.10117	0.04852	0.07868	1.52640	ac6
NM_019032	ADAMTSL4	0.04017	0.24070	0.41811	0.10638	0.91323	1.52436	ac6
NR_003678	NCOR1P1	0.98512	0.24816	0.63436	0.46421	0.52120	1.52421	ac6
NM_001142576	IMPDH1	0.49336	0.02027	0.62892	-0.12197	1.13019	1.52376	ac6
NM_182642	CTDSP1	0.30834	0.01497	0.20980	-0.47136	1.01224	1.52335	ac6
NM_015326	SRGAP2	0.26708	0.72226	0.75645	0.20690	1.37033	1.52294	ac6
NR_030366	MIR636	0.16987	0.98152	0.18303	1.06070	-0.53909	1.52240	ac6
NM_001206654	NCOR2	0.43781	-0.65060	0.40740	-0.05662	1.01440	1.52085	ac6
NM_022153	C10orf54	-0.03115	-0.13486	0.28917	0.38248	1.18029	1.52035	ac6
NM_002360	MAFK	0.59960	1.17373	0.80437	0.30487	1.05884	1.52028	ac6
NM_024668	ANKHD1	0.42116	0.52890	1.20987	0.57910	1.21696	1.52016	ac6
NM_004556	NFKBIE	0.19016	0.72026	0.87919	0.32460	1.21379	1.51968	ac6
NM_002117_6	HLA-C	0.30299	0.25392	0.44022	0.33235	1.14689	1.51944	ac6
NM_004295	TRAF4	0.52054	0.77895	0.64166	0.45440	1.09155	1.51875	ac6
NM_014256	B3GNT3	-0.03355	-0.10233	0.27133	0.38146	1.30621	1.51735	ac6

NM_003155	STC1	0.04148	0.05301	0.21341	0.77458	1.02825	1.51604	ac6
NM_001163258	FAM63A	0.33669	0.89689	-0.94998	-0.09346	1.43645	1.51490	ac6
NM_133328	DEDD2	0.14833	0.04527	0.09170	0.17246	1.17665	1.51477	ac6
NM_005957	MTHFR	0.09082	0.21887	-0.00889	-0.52665	1.28289	1.51376	ac6
NM_001002857	ANXA2	0.08722	-0.38897	0.71305	0.49550	1.27359	1.51289	ac6
NM_004603	STX1A	-0.74038	1.00643	1.06314	0.90584	1.11616	1.51115	ac6
NM_001128833	ZBTB4	-2.33878	-0.57585	0.24380	-0.86203	1.29049	1.51034	ac6
NM_005586	MDFI	0.07149	0.27939	0.52970	0.48268	0.98162	1.50993	ac6
NM_001135729	TOM1	0.50672	0.37757	0.12883	-0.75518	1.26247	1.50949	ac6
NR_003594_8	REXO1L2P	0.03033	0.81796	0.83131	0.80907	1.08021	1.50678	ac6
NM_018004	TMEM45A	0.20102	-0.00931	0.37263	0.76681	1.38439	1.50562	ac6
NM_002210	ITGAV	-0.43372	0.34316	0.62058	1.36168	1.38773	1.50499	ac6
NM_183008	UBXN11	0.92376	0.31795	0.26076	-0.09449	0.78418	1.50474	ac6
NR_030775	SHISA4	-0.60891	-0.24441	-1.90667	-0.41498	0.74357	1.50432	ac6
NM_001083963	TDRKH	1.36968	1.17764	-0.22455	0.80014	-0.11878	1.50378	ac6
NM_002434	MPG	-1.15431	0.11465	-0.55561	0.11075	0.96160	1.50347	ac6
NM_001077198	ATG9A	-0.03156	-0.07146	-0.04506	-0.01601	1.17902	1.50303	ac6
NM_004878	PTGES	-0.05803	-0.20486	0.04280	0.17120	1.07964	1.50054	ac6
NM_001098637	PWWP2B	0.35906	0.33497	-0.40121	0.01412	0.84120	1.50001	ac6
NM_004433	ELF3	0.68959	0.50265	-0.30931	0.16629	1.14816	1.49997	ac6
NM_002502	NFKB2	-0.24457	0.38876	0.84795	0.40538	1.16044	1.49733	ac6
NM_001569	IRAK1	-0.14754	-0.21861	-0.17686	-0.20521	0.81332	1.49623	ac6
NM_001003696	ATP5J	0.17162	0.26426	1.07130	0.75457	1.01364	1.49589	ac6
NM_001136157	OTUD5	0.79826	1.03097	0.79586	0.17928	1.16560	1.49552	ac6
NM_001145063	GATSL1	0.02151	-0.16888	-0.40524	-0.53795	0.90552	1.49294	ac6
NM_002414_1	CD99	0.23966	-0.14365	0.28133	0.20591	1.17560	1.49217	ac6
NM_001780	CD63	0.06753	-0.10083	0.22585	0.63231	1.11953	1.48863	ac6
NM_183006	DLGAP4	0.23867	0.40311	0.69795	0.39386	1.26781	1.48638	ac6
NM_019554	S100A4	-0.05926	-0.61029	0.14449	0.09548	0.55744	1.48561	ac6
NM_152727	CPNE2	0.01580	-0.15644	0.15553	0.34513	1.37796	1.48405	ac6
NM_002741	PKN1	-0.10159	-0.25002	0.40579	0.09576	0.26038	1.48288	ac6
NM_007074	CORO1A	0.13853	0.05507	0.78982	0.83690	1.24128	1.48093	ac6
NM_002574	PRDX1	0.37086	-1.37763	-0.01905	0.49168	1.24608	1.48023	ac6
NM_001130028	CLK3	0.82612	1.27427	1.05904	0.74000	1.13266	1.47984	ac6
NM_001136218	TMEM51	-0.76432	-1.13576	0.91892	1.42821	0.21026	1.47866	ac6
NM_181304	MRPL52	0.09498	0.23517	-0.67684	1.06856	0.65498	1.47850	ac6
NM_001034841	ITPRIPL2	1.39155	1.43183	1.14990	0.46874	1.17186	1.47783	ac6
NM_025241	UBXN6	0.11279	-0.27210	-0.00552	-0.06400	0.88487	1.47728	ac6
NM_001199267	DGKZ	0.46097	-0.06746	0.87157	0.13389	0.54614	1.47616	ac6
NM_181462	MRPL55	-0.09201	-0.00709	-0.89571	-0.12442	0.61858	1.47498	ac6
NM_033386	MICALL1	0.00667	-0.02059	0.00261	-0.16234	1.19655	1.47492	ac6
NM_001161359	FCHO1	0.23570	0.05190	0.51732	0.60634	1.14148	1.47321	ac6
NM_030971	SFXN3	0.07915	-0.02548	0.57927	0.59582	1.21851	1.47218	ac6
NM_001127662	GSN	-0.22005	-0.04873	-0.16247	0.06885	0.91186	1.47015	ac6
NM_002284	KRT86	0.26548	0.14045	0.34120	0.55546	0.90377	1.46541	ac6
NM_015104	ATG2A	0.00993	-0.03456	-0.00221	-0.13909	1.20351	1.46518	ac6
NM_198332	STAT2	1.34782	-0.07757	1.14309	0.50601	1.33446	1.46385	ac6

NM_007145	ZNF146	0.36130	0.14796	0.35005	-0.49599	1.04320	1.46154	ac6
NM_001943	DSG2	-0.14850	-0.12810	0.24294	0.65286	1.33745	1.46098	ac6
NM_003768	PEA15	0.02556	0.15038	0.60549	0.57645	1.03891	1.45775	ac6
NM_001042679	RHOC	0.03811	-0.21948	-0.06447	0.01751	0.89674	1.45443	ac6
NM_015711	GLTSCR1	0.05704	-0.26529	-0.18304	-0.40733	1.12622	1.45260	ac6
NM_015401	HDAC7	0.23279	-0.75499	-1.35194	-1.05887	1.35304	1.45160	ac6
NM_016584	IL23A	0.20451	0.60708	0.98821	0.86361	1.30975	1.45087	ac6
NM_001135917	DOLPP1	-0.80994	0.30410	0.89803	-1.33404	0.92371	1.44831	ac6
NM_001122956	DBNL	-0.46012	-0.71370	-0.72146	0.18577	1.25704	1.44674	ac6
NM_032714	INF2	-0.02448	0.22398	1.14482	1.06873	1.05799	1.44609	ac6
NM_024731	KLHL36	0.25664	0.28217	0.59439	0.40697	1.13387	1.44225	ac6
NM_016332	SEPX1	0.11738	-0.23872	-0.13205	-0.06384	0.90068	1.44008	ac6
NM_005345_1	HSPA1A	0.11683	-0.29839	-0.57336	-0.86094	1.07449	1.43630	ac6
NM_001174100	PCBP4	-0.59712	-0.21852	-0.06858	0.03880	0.51406	1.43285	ac6
NM_001015002	LLGL2	0.20376	-0.19542	-0.24832	0.05062	0.85777	1.43276	ac6
NM_001195227	TRPM4	0.09481	-0.04068	0.18784	0.25053	0.97732	1.43202	ac6
NM_032409	PINK1	0.02275	-0.23149	-0.02072	0.00762	1.12235	1.43133	ac6
NM_001185077	ARHGDIA	0.28945	0.46815	0.62783	0.46337	0.48991	1.42804	ac6
NM_207122	EXT2	0.74248	0.52501	0.81256	0.86075	1.13822	1.42544	ac6
NM_022152	TMBIM1	-0.01242	-0.15290	0.17063	0.12730	1.00541	1.42517	ac6
NM_001100619	CABLES1	-0.12221	0.16494	0.96261	0.89160	1.06397	1.42451	ac6
NR_003016	SNORA26	0.65794	-0.12878	0.54874	0.48035	0.89756	1.42378	ac6
NM_020428	SLC44A2	-0.19151	-0.43910	0.11897	0.00883	0.82035	1.42272	ac6
NM_022737	LPPR2	-0.14944	0.27650	-0.01537	-0.44434	1.05839	1.42151	ac6
NR_039810	MIR4664	-0.81706	-0.30324	-0.61475	-0.95297	-0.52732	1.41970	ac6
NM_001146069	MFSD10	0.14635	0.12537	0.22931	0.20478	0.98589	1.41954	ac6
NM_152925	CPNE1	-1.04165	-0.04593	-1.80901	-0.31175	0.58187	1.41935	ac6
NM_007267	TMC6	0.10578	0.05824	-0.32437	0.04813	1.07010	1.40691	ac6
NM_018174	MAP1S	0.11589	0.40298	0.01059	-0.28603	1.13454	1.40557	ac6
NM_002587	PCDH1	-0.18964	-0.57425	-0.98776	-0.99815	1.02757	1.40371	ac6
NM_002281	KRT81	0.28303	0.10971	0.34936	0.68815	0.86964	1.40322	ac6
NM_005785	RNF41	-0.59691	-0.82085	-0.89121	0.30636	0.67100	1.40262	ac6
NM_178508	C6orf1	-0.20110	0.53462	0.69879	0.93373	1.36669	1.40183	ac6
NM_001975	ENO2	0.02537	-0.04718	0.18876	-0.08779	0.70851	1.39947	ac6
NM_006076	AGFG2	-0.02333	-0.24395	-0.18467	-0.10236	1.22967	1.39903	ac6
NM_006986	MAGED1	0.37452	-0.92817	0.41003	-0.76667	0.75385	1.39863	ac6
NM_152221	CSNK1E	0.19296	0.59358	0.76759	0.59013	0.93722	1.39650	ac6
NM_001204911	C16orf57	0.03251	0.24945	0.81414	0.72720	1.13871	1.39636	ac6
NR_003045	SNORD17	0.38023	-0.64795	0.28599	0.60061	0.80527	1.39534	ac6
NM_018067	MAP7D1	0.01814	0.20723	0.64480	0.32512	1.00101	1.39503	ac6
NM_148414	ATXN2L	-0.09157	0.46054	-0.00326	0.50466	-0.43390	1.39330	ac6
NM_002744	PRKCZ	0.07212	0.02168	0.20634	-0.09898	1.01983	1.39080	ac6
NM_015260	SIN3B	0.12067	0.06436	0.05387	-0.14345	1.12967	1.39012	ac6
NM_032017	STK40	0.08794	0.46262	0.83684	0.61697	1.00617	1.38972	ac6
NM_030819	GFOD2	-0.13030	-0.23850	0.26083	0.09128	1.04994	1.38840	ac6
NM_181671	PITPNC1	0.09819	-0.02354	0.21322	0.39212	1.24926	1.38649	ac6
NM_006834	RAB32	0.55128	0.68591	1.01312	0.53277	1.03409	1.38445	ac6

NM_001003692	ZMAT5	0.09365	-0.28779	-0.19158	-0.06969	0.87065	1.38258	ac6
NM_182538	SPNS3	0.09921	0.13231	0.24692	0.09911	1.08510	1.38230	ac6
NM_004761_1	RGL2	-0.11699	-0.31230	-0.11200	-0.30422	0.95157	1.38073	ac6
NM_002282	KRT83	0.26026	-0.02672	0.34186	0.65994	0.87431	1.38014	ac6
NM_024107	TMUB2	0.72808	1.29708	0.51223	0.73323	1.05677	1.37887	ac6
NM_001627	ALCAM	-0.23757	0.19746	0.39128	0.54643	0.88374	1.37867	ac6
NM_001178108	ZNF185	-0.18559	-0.34269	-0.41983	-0.31397	0.95982	1.37790	ac6
NM_001012426	FOXP4	0.12087	0.34308	0.73689	0.20692	1.20454	1.37719	ac6
NM_001199944	SH3GL1	0.00315	-0.02653	0.31705	0.31904	0.77331	1.37683	ac6
NM_005873	RGS19	0.95181	-0.01642	0.85899	-0.40507	1.30014	1.37565	ac6
NM_002283	KRT85	0.24776	0.13812	0.38137	0.49722	0.81150	1.37555	ac6
NM_000509	FGG	-0.22850	-0.06293	-0.75266	-0.14892	0.84649	1.37510	ac6
NM_000267	NF1	0.71596	0.19451	0.27219	0.45177	0.86883	1.37402	ac6
NM_001130144	LTBP3	0.41579	0.68747	0.52709	0.77871	1.25730	1.37175	ac6
NM_024681	KCTD17	-0.15678	-0.22731	0.24331	0.13769	1.06746	1.37112	ac6
NM_133635	POFUT2	0.47308	0.31816	0.30059	0.03204	1.06301	1.37089	ac6
NM_001098785	FAM89B	0.06653	0.16063	-0.07108	0.28976	1.01941	1.36973	ac6
NM_003225	TFF1	0.14876	-0.00542	0.52070	0.87086	1.06733	1.36895	ac6
NM_004494	HDGF	0.12986	0.16533	0.39222	0.22037	1.04174	1.36326	ac6
NM_004359	CDC34	0.01116	0.17633	0.75776	0.63473	1.06907	1.36199	ac6
NM_001177358	GPR137	0.39900	0.36488	0.89035	0.01175	0.45652	1.36181	ac6
NM_002131	HMGA1	0.15813	0.01069	0.39946	0.73739	1.10377	1.36099	ac6
NM_001128613	NUDT22	-0.03070	0.00089	0.10089	0.28165	0.63807	1.36028	ac6
NM_025049	PIF1	-0.12619	-0.35595	-0.50907	-0.55154	0.71089	1.35861	ac6
NM_022059	CXCL16	-0.22718	-0.06472	0.77736	0.61054	1.29808	1.35674	ac6
NM_000149	FUT3	-0.31401	0.15886	-1.15074	-0.03219	0.82129	1.35495	ac6
NM_001033044	GLUL	0.15122	-0.26132	0.32296	0.16055	1.13999	1.35490	ac6
NM_014164	FXYD5	-0.02497	-0.30800	-0.06804	0.02490	0.82964	1.35485	ac6
NM_012339	TSPAN15	0.05007	-0.13439	0.17061	0.36770	0.94162	1.35255	ac6
NM_000966	RARG	0.22964	0.38358	0.49587	0.17168	1.09580	1.35121	ac6
NM_198593	C1QTNF1	-0.49300	-1.07638	-0.42011	-0.16450	1.09306	1.35056	ac6
NR_002576	SNORA21	-0.84876	0.19315	-2.37117	-1.39313	-2.14399	1.34978	ac6
NM_001178008	CBS	-0.06058	-0.20506	-0.01829	-0.67557	0.52722	1.34870	ac6
NM_002835	PTPN12	-0.43255	0.07112	-0.17508	-0.46754	1.30678	1.34563	ac6
NM_018173	PLEKHG6	0.05004	0.20858	-0.55818	-1.07727	1.27803	1.34517	ac6
NR_030717	SNAPC2	-0.25146	0.22601	0.66513	0.26882	0.57782	1.34324	ac6
NR_038103	TECR	0.45824	-0.68556	0.36776	0.57356	0.52013	1.33815	ac6
NM_001199867	MARK4	-0.07370	-0.02556	-0.11885	-0.36867	1.10763	1.33763	ac6
NM_182639	HPS1	-0.05579	0.15652	0.47666	0.74887	1.25341	1.33627	ac6
NM_001127621	GALE	-0.33320	-1.04033	-0.30176	0.86608	0.18336	1.33136	ac6
NM_001001852	PIM3	0.59468	0.39157	-0.06345	0.02562	1.06311	1.33117	ac6
NM_002414	CD99	-0.08785	-0.32313	-0.02498	-0.04603	0.85903	1.33085	ac6
NM_001566	INPP4A	0.09385	0.24639	0.73483	0.48595	1.18488	1.32868	ac6
NM_032527	ZGPAT	-1.17474	0.10832	0.14244	0.88345	0.93807	1.32854	ac6
NM_005346	HSPA1B	0.06983	-0.39700	-0.55909	-0.75490	1.01898	1.32642	ac6
NM_030926	ITM2C	-0.03124	0.20765	0.54769	0.53128	1.15010	1.32460	ac6
NM_079423	MYL6	0.08706	-0.21001	0.03381	0.15429	0.84823	1.32338	ac6

NM_005273	GNB2	0.08150	-0.03677	0.09979	-0.09860	0.82764	1.32305	ac6
NM_001080434	LMTK3	0.09404	-0.03901	-0.09808	-0.22053	0.98540	1.32060	ac6
NM_024328	THTPA	0.43734	-0.12793	-0.89352	1.09598	0.69517	1.31911	ac6
NM_002031	FRK	0.51377	0.39031	0.74113	0.65324	1.19679	1.31898	ac6
NM_025250	TTYH3	0.05720	-0.08734	-0.21478	-0.52483	0.87871	1.31837	ac6
NM_052897	MBD6	0.03284	0.10538	-0.05179	-0.37350	1.04682	1.31707	ac6
NM_001242824	FDPS	-0.12404	-0.32709	0.35860	0.61865	-0.15817	1.31694	ac6
NM_017432	PTOV1	0.05205	-0.15948	-0.20033	-0.05680	0.93315	1.31673	ac6
NM_004957	FPGS	0.22424	0.06697	0.35196	0.48750	0.58587	1.31398	ac6
NM_001010972	ZYX	0.19281	0.53033	1.02015	0.99033	0.96759	1.31242	ac6
NM_001206799	PKM2	-1.24311	-3.89115	-0.36711	-0.52362	-0.75246	1.30986	ac6
NM_032140	C16orf48	0.02080	0.30523	-0.08969	-0.29503	0.65912	1.30867	ac6
NM_001242817	DEF8	1.15095	0.27294	0.67307	0.53293	-0.25380	1.30813	ac6
NM_007260	LYPLA2	-0.00806	-0.09806	0.19137	0.19925	0.85589	1.30514	ac6
NM_024707	GEMIN7	0.20534	-0.28018	-0.08841	0.33868	0.59884	1.30368	ac6
NM_001122823	GTF3C5	-1.33942	-0.40930	0.49284	-0.31529	0.66675	1.30325	ac6
NM_001102596	DTX2	0.00999	-0.29250	-0.31731	-0.42396	0.82202	1.30314	ac6
NM_023948	MOSPD3	-0.43378	-0.75661	-0.29955	-0.64785	0.44574	1.30226	ac6
NM_003116	SPAG4	0.63052	-0.06458	0.51777	0.82307	1.12433	1.30219	ac6
NM_001042483	NUPR1	-0.18225	-0.23514	-0.07030	-0.34023	0.94646	1.30186	ac6
NM_000445	PLEC	0.20331	0.31321	0.52913	-1.27530	0.74762	1.30139	ac6
NM_012478	WBP2	0.09133	-0.15391	-0.12133	-0.34068	0.98089	1.30091	ac6
NM_013443	ST6GALNAC6	-0.18384	-0.19817	-0.12255	-0.24469	1.00297	1.29836	ac6
NM_002918	RFX1	0.01312	0.33824	0.30409	-0.14535	0.94791	1.29832	ac6
NM_198850	PHLDB3	0.40876	0.18124	-0.01514	-0.23090	1.04361	1.29652	ac6
NM_015330	SPECC1L	1.00633	0.87560	0.37280	0.21993	0.04733	1.29485	ac6
NM_002201	ISG20	0.30273	0.48952	0.79006	1.11344	1.10891	1.29352	ac6
NM_005660	SLC35A2	0.39872	0.46401	0.51548	0.44295	1.00434	1.29315	ac6
NM_001124758	SPNS2	0.06806	0.08283	0.65048	0.79580	0.91234	1.29295	ac6
NM_007311	TSPO	0.16166	0.06945	-0.01494	-0.24888	0.81864	1.29198	ac6
NR_002207	CSNK2A1P	-0.46141	0.55238	0.60567	-0.22255	0.70522	1.29198	ac6
NM_001146157	FAM25A	0.13121	-0.13544	0.10156	0.46367	0.60662	1.28867	ac6
NM_202468	GIPC1	-0.66365	-0.68966	-0.19204	-0.34941	0.45655	1.28802	ac6
NM_014275	MGAT4B	0.02321	-0.06821	0.12091	0.01723	0.87067	1.28789	ac6
NM_033250	PML	-0.14229	0.71824	0.25478	0.71404	0.93472	1.28658	ac6
NM_017572	MKNK2	0.05255	-0.73379	-0.35191	-0.55801	0.88109	1.28497	ac6
NM_001013841	STAP2	-0.05300	-0.13203	0.18497	-0.16752	1.00582	1.28404	ac6
NM_199444	COPE	-0.20683	0.02727	0.12594	-0.25485	0.70728	1.28254	ac6
NM_005178	BCL3	0.13533	-0.28300	-0.45780	-0.01686	0.94885	1.28087	ac6
NM_013321	SNX8	-0.00846	0.09603	0.89956	0.94446	0.85192	1.28024	ac6
NM_016539	SIRT6	0.22136	0.15626	-0.22401	-0.28650	0.80439	1.27915	ac6
NM_002730	PRKACA	0.13069	0.27363	0.55147	-0.25930	0.90250	1.27729	ac6
NM_002359	MAFG	0.22544	0.74171	0.41123	0.17539	0.87459	1.27711	ac6
NM_014750	DLGAP5	0.48243	0.63830	0.55613	0.89527	1.12273	1.27678	ac6
NM_001142646	TPRA1	0.43361	0.69666	-0.39464	-0.23588	-0.32449	1.27667	ac6
NM_001077621	VPS37D	-0.03504	-0.39691	0.01374	0.12641	1.22873	1.27524	ac6
NR_030171	MIR492	0.24229	0.09167	0.32522	0.15512	0.76261	1.27465	ac6

NM_001085375	C1orf226	-0.09916	-0.28871	0.45746	0.68216	1.10064	1.27443	ac6
NM_001031717	CRELD1	-0.27612	-0.39047	-0.25496	-0.72181	0.79280	1.27312	ac6
NM_177526	PPAP2C	-0.19449	-0.06273	-0.06053	-0.57426	0.74916	1.26715	ac6
NM_001042353	FAM110A	-0.08034	-0.48411	0.15715	0.33956	0.88444	1.26684	ac6
NM_017550	MIER2	0.46259	0.27191	0.19326	-0.01140	0.83583	1.26658	ac6
NM_138402	SP140L	0.29388	-0.10815	0.04106	0.74487	1.25748	1.26530	ac6
NM_001130517	MPST	0.11839	-1.15857	-0.64968	-1.70325	0.88167	1.26250	ac6
NR_002556_1	LOC388242	0.19335	0.40628	0.62004	0.83391	0.67336	1.26210	ac6
NM_015492	C15orf39	0.00028	0.72635	0.72432	0.35866	0.99578	1.26037	ac6
NM_002961	S100A4	0.03663	-0.27248	0.12800	0.21641	0.80501	1.25940	ac6
NM_001018000	KAZN	0.62670	0.90393	0.99874	-0.49480	1.18451	1.25770	ac6
NM_006086	TUBB3	0.06880	0.06069	0.28594	0.28934	0.84164	1.25764	ac6
NM_004095	EIF4EBP1	0.13020	-0.21540	0.09590	0.26217	1.00575	1.25676	ac6
NM_024329	EFHD2	0.02527	0.13916	0.86373	0.97386	0.81394	1.25653	ac6
NM_024535	CORO7	0.26417	0.37929	0.55808	0.41080	0.86421	1.25397	ac6
NM_002593	PCOLCE	-0.35164	-0.06069	-0.18117	-0.37237	0.78542	1.25348	ac6
NM_032732	IL17RC	0.48807	0.09355	-0.13795	0.14932	0.86947	1.25310	ac6
NM_058195	CDKN2A	0.21278	0.04624	0.37898	0.59038	0.89991	1.25285	ac6
NM_014286	NCS1	0.09415	0.03163	1.05459	1.10491	0.86100	1.25203	ac6
NM_006663	PPP1R13L	0.00796	0.59625	0.89042	0.28853	0.97739	1.25190	ac6
NM_003461	ZYX	0.33740	0.40620	0.68668	1.11255	1.01603	1.24989	ac6
NM_001143781	FKBP11	-0.67345	0.06391	0.74655	0.88637	0.77031	1.24968	ac6
NM_001099784	FBXL19	-0.13379	0.13979	0.26526	-0.07148	0.75310	1.24858	ac6
NM_001042368	RALGDS	0.76855	0.74309	0.93147	-0.84578	0.39125	1.24738	ac6
NM_002668	PLP2	-0.00980	-0.19793	0.13822	0.20570	0.93841	1.24707	ac6
NM_015164	PLEKHM2	0.07599	0.11448	0.22593	0.00898	0.78537	1.24704	ac6
NM_006912	RIT1	-0.18851	0.18304	0.20049	0.22338	1.13003	1.24207	ac6
NM_001077498	C17orf63	0.15276	0.15489	0.41426	0.24593	1.01858	1.24158	ac6
NR_003005	SCARNA4	-1.18526	-0.40891	-0.15541	-1.14445	0.65673	1.24119	ac6
NM_015063	SLC8A2	0.10917	0.13657	0.57879	0.18662	0.97131	1.24082	ac6
NM_033033	KRT82	0.08259	0.03413	0.14681	0.42397	0.68848	1.23869	ac6
NM_001002295	GATA3	0.38808	-0.65398	0.03153	0.26527	0.31818	1.23835	ac6
NM_016823	CRK	0.20110	0.94927	1.12859	1.19584	0.69118	1.23751	ac6
NM_013974	DDAH2	-0.19527	-0.16384	-0.23497	-0.33866	1.03100	1.23625	ac6
NM_030973	MED25	0.05396	-0.04947	0.04981	0.03556	0.89321	1.23601	ac6
NM_001190452	MTRNR2L1	-0.08628	-0.29812	-0.28063	-0.25831	1.05574	1.23490	ac6
NM_001256139	CAPG	0.02086	-0.38105	-0.08284	-0.23341	0.63171	1.23304	ac6
NM_199242	UNC13D	0.01903	-0.06348	0.07086	-0.18265	0.66515	1.23303	ac6
NM_001126340	ORAI2	-0.16288	-0.42996	-0.30248	0.03608	0.40557	1.23294	ac6
NM_002140	HNRNPK	-0.15629	0.50650	0.02410	0.48540	-0.20518	1.23272	ac6
NM_006589	FAM189B	-0.04935	-0.06918	-0.10639	-0.19504	0.76062	1.23047	ac6
NM_001206790	TMPRSS13	-0.14689	-0.37854	-0.12384	-0.12523	1.18538	1.23022	ac6
NM_006483	DYRK1B	0.25410	-0.53339	-0.42843	-0.86124	1.08484	1.22967	ac6
NR_003606	ZNFX1-AS1	0.62030	0.31678	0.87519	0.22742	0.84500	1.22679	ac6
NM_002963	S100A7	0.57452	0.15824	-0.15325	0.49679	1.11512	1.22541	ac6
NM_183057	VPS28	-0.02792	0.31717	0.10180	0.21233	0.84753	1.22443	ac6
NM_006404	PROCR	-0.22824	-0.13901	0.15408	-0.02732	1.03686	1.22412	ac6

NM_004324	BAX	-0.12388	-0.55798	0.04564	-0.15256	0.75201	1.22334	ac6
NM_001065	TNFRSF1A	-0.05412	-0.11250	0.38159	0.34085	0.96298	1.22218	ac6
NM_003141	TRIM21	-0.00560	0.00658	0.53051	0.33138	0.88491	1.22174	ac6
NM_006354	TADA3	0.58592	-0.05955	-0.15444	0.23601	0.86713	1.22100	ac6
NM_017843	BCAS4	0.79081	-0.52495	-0.76575	-0.08397	-0.56965	1.22037	ac6
NM_005730	CTDSP2	-0.04653	0.02425	0.10537	-0.16574	1.06779	1.21952	ac6
NM_001199723	CRABP2	-0.07091	0.01089	0.39418	-0.06705	1.14543	1.21908	ac6
NM_017514	PLXNA3	-0.13884	0.05099	0.31232	0.06562	0.89903	1.21904	ac6
NM_001134774	KLC2	0.04879	-1.44892	0.48998	0.13716	0.78945	1.21876	ac6
NM_205835	LSR	0.05769	0.02961	0.18466	-0.34801	0.65814	1.21841	ac6
NM_002230	JUP	0.08437	0.25501	0.39987	-0.01980	0.73609	1.21698	ac6
NM_001039550	DNAJB2	0.07613	0.09226	0.18531	0.16529	0.76750	1.21629	ac6
NM_213618	ST5	0.08411	-0.18025	-0.75903	-1.25633	0.76294	1.21624	ac6
NM_153477	UXT	0.77326	0.25221	0.22044	1.08348	0.38495	1.21357	ac6
NM_207012	AP3M1	0.51303	0.70112	0.61779	0.10998	1.15058	1.21344	ac6
NM_130900	RAET1L	-0.17519	-0.16500	0.44698	0.94608	1.04453	1.21326	ac6
NM_017585	SLC2A6	0.50488	0.31778	0.40041	-0.02399	0.82994	1.21306	ac6
NM_198043	ZDHHC16	-0.15982	-0.00235	-0.98520	-0.21905	0.74939	1.21219	ac6
NM_031232	NECAB3	0.34530	0.66079	0.25486	-0.30488	0.86391	1.21072	ac6
NM_001024213	S100A13	0.38518	-0.42037	0.07577	-0.04163	0.75961	1.20951	ac6
NM_001136113	POMT1	0.81847	-0.12372	0.28038	0.19833	0.20780	1.20901	ac6
NR_028040	GATS	0.29797	0.17556	0.01234	-0.39930	0.73591	1.20901	ac6
NM_053004	GNB1L	0.28515	0.23816	-0.18222	-0.37866	0.69754	1.20771	ac6
NM_138575	PGAM5	0.73734	0.19009	0.54570	-0.24709	0.50252	1.20730	ac6
NM_005990	STK10	-0.06845	-0.04299	1.20413	0.94625	0.96642	1.20664	ac6
NM_001098576	TMBIM6	-0.35167	0.29449	0.78333	0.66471	0.92823	1.20508	ac6
NR_003083	SLC6A10P	0.07085	0.01100	0.06942	0.02805	0.88682	1.20485	ac6
NM_013333	EPN1	0.04107	-0.14326	0.02094	-0.20287	0.72790	1.20297	ac6
NR_046344	CALHM2	-0.28024	-0.95697	-0.28617	-0.00046	0.83729	1.20218	ac6
NM_001142298	SQSTM1	-1.71096	-2.11714	-0.73216	0.18616	-0.10140	1.20193	ac6
NM_018569	AP1AR	0.00387	0.86107	0.59458	0.89312	0.99414	1.20143	ac6
NM_005980	S100P	0.27828	-0.11960	0.20627	0.60293	1.02080	1.20017	ac6
NM_147202	C9orf25	-0.35693	0.65988	-0.28226	-1.75566	1.19368	1.19925	ac6
NM_031412	GABARAPL1	0.35151	0.81208	1.03315	0.71327	1.19498	1.19870	ac6
NM_015683	ARRDC2	0.44185	0.52427	0.94308	0.67295	1.18852	1.19835	ac6
NM_130465	TSPAN17	-0.01276	-0.09570	-0.14287	-0.06378	0.76567	1.19819	ac6
NM_017644	KLHL24	-0.04864	0.04439	-0.49547	-0.30945	1.19459	1.19783	ac6
NM_006532	ELL	0.16334	0.77238	0.49466	0.12922	0.88674	1.19748	ac6
NM_138471	C11orf84	0.07264	-0.27168	-0.16951	-0.29561	0.82549	1.19603	ac6
NM_005302	GPR37	0.10692	0.65790	0.61571	0.15768	0.96093	1.19486	ac6
NM_032310	C9orf89	0.08148	0.09935	0.36874	0.30615	0.80017	1.19428	ac6
NM_020859	SHROOM3	-0.01821	0.01391	0.43321	0.38956	1.19101	1.19292	ac6
NM_014297	ETHE1	0.14025	-0.16712	0.02926	0.18923	0.83791	1.19092	ac6
NM_031937	TBC1D10A	0.17398	0.31469	0.29511	-0.25984	0.79503	1.18988	ac6
NM_145804	ABTB2	-0.00709	0.21485	0.50967	-0.08065	0.86505	1.18758	ac6
NM_152264	SLC39A13	-0.32190	-0.01509	0.14047	0.00693	0.58626	1.18736	ac6
NM_001166114	PNPLA6	0.08910	-0.13962	-0.04488	-0.10427	0.63085	1.18639	ac6

NM_001242769	MTHFD1L	-0.80649	-0.09818	0.70891	1.17679	1.10139	1.18473	ac6
NM_202469	GIPC1	-0.80869	-0.81184	-0.70076	-0.10977	0.01523	1.18420	ac6
NM_138392	SHKBP1	0.06117	-0.14722	-0.08077	-0.09189	0.74723	1.18381	ac6
NR_001434_7	HLA-H	0.27675	-0.09830	0.45420	0.52244	1.03159	1.18359	ac6
NM_001174087	NCOA3	-0.46318	0.49398	-1.44984	-0.95108	1.05162	1.18299	ac6
NM_025240	CD276	-0.24599	-0.22930	0.75546	0.64215	0.63888	1.18180	ac6
NM_005527	HSPA1L	-0.17217	-0.61254	-0.60850	-0.86118	0.72736	1.18083	ac6
NM_017766	CASZ1	0.07044	-0.13568	0.07430	-0.25471	0.80575	1.18074	ac6
NM_198216	SNRPB	-0.01584	-0.08663	0.41998	0.48315	0.85410	1.18071	ac6
NM_014501	UBE2S	0.45634	0.42807	0.63351	0.51602	0.82244	1.18037	ac6
NM_001304	CPD	0.12726	0.09059	0.14268	0.07020	1.12928	1.17903	ac6
NM_178580	HM13	0.10323	0.39265	0.61774	0.50538	0.57226	1.17887	ac6
NM_147184	TP53I3	0.15622	0.44087	-0.26010	-0.43161	1.11704	1.17881	ac6
NM_001172219	SCMH1	0.87747	0.55810	-0.20574	-1.40089	0.85104	1.17852	ac6
NR_037709	TEN1-CDK3	0.11633	-0.13143	0.40022	0.10583	-0.17030	1.17623	ac6
NR_033990	LOC10012913	0.63261	-0.74714	0.20891	0.10602	0.69786	1.17557	ac6
NM_024676	SH3D21	-0.04868	-0.02150	0.42979	0.23661	0.46190	1.17356	ac6
NM_033025	SYDE1	0.36558	0.19950	-0.78950	-1.10141	0.56423	1.17279	ac6
NM_012229	NT5C2	-0.17199	0.02045	-0.43349	0.85024	1.05441	1.17272	ac6
NM_001024465	SOD2	-0.17716	-0.11840	0.00548	0.28929	0.93696	1.17226	ac6
NM_001178133	SUFU	-0.04352	-0.76166	-0.53448	0.07092	0.81063	1.17225	ac6
NM_001190487	MTRNR2L6	0.01996	-0.19428	-0.14094	-0.54533	1.02910	1.17166	ac6
NM_000714	TSPO	0.31065	-0.25167	-0.01965	0.33368	0.73931	1.17124	ac6
NM_015872	ZBTB7B	-0.01759	-0.05078	-0.54658	-0.79977	0.71098	1.17072	ac6
NM_021260	ZFYVE1	-0.01228	-0.00615	0.09363	0.10821	0.94988	1.17033	ac6
NM_016579	CD320	0.20025	0.09543	0.28831	0.36260	0.77702	1.16770	ac6
NM_201566	SLC16A13	0.50825	0.08122	0.25446	-0.16168	1.16735	1.16761	ac6
NM_001080412	ZBTB38	-0.08440	0.48041	0.33668	0.66615	1.03906	1.16750	ac6
NM_177441	TMUB2	0.24577	-0.01409	-0.55012	-0.74561	0.80743	1.16580	ac6
NM_001122	PLIN2	-0.32500	-0.45765	0.13993	0.28185	1.16476	1.16495	ac6
NR_033874	FLJ20021	0.44418	0.31149	0.38168	0.73208	0.77790	1.16491	ac6
NM_198433	AURKA	-0.62034	-0.69438	-1.24571	-0.57827	0.42388	1.16431	ac6
NM_018833_3	TAP2	-0.07939	-0.25296	0.19643	0.21241	0.67707	1.16408	ac6
NM_014045	LRP10	-0.02378	-0.04040	0.09968	-0.03181	0.80018	1.16282	ac6
NM_003255	TIMP2	-0.06051	-0.22774	-0.04279	-0.21439	0.81769	1.16281	ac6
NM_004428	EFNA1	0.61213	0.57598	0.79316	0.43487	0.72462	1.16227	ac6
NM_001014794	ILK	0.13704	0.00391	0.25462	-0.05750	0.77390	1.16091	ac6
NR_033465	NT5C3L	0.09092	-0.10068	0.26171	0.13055	0.66210	1.15962	ac6
NM_020831	MKL1	-0.06499	0.02551	1.01670	0.47162	0.74346	1.15931	ac6
NM_001242840	GUK1	0.23275	-0.26830	0.43920	0.84513	0.55577	1.15897	ac6
NM_004120	GBP2	-0.03634	0.44096	0.08422	0.71945	1.05361	1.15793	ac6
NM_000366	TPM1	0.48516	0.51613	1.03788	0.95985	0.70142	1.15652	ac6
NM_001113348	ECE1	-0.48957	-0.68891	0.35065	0.16043	0.67282	1.15607	ac6
NM_033045	KRT84	0.13862	0.18380	0.40324	0.68663	0.75210	1.15507	ac6
NM_001201576	PLSCR3	0.39703	0.28057	0.48047	-0.23797	0.75030	1.15420	ac6
NR_033250	LOC388499	-0.05328	-0.30143	0.07145	0.08940	0.79544	1.15414	ac6
NM_006700	TRAFD1	0.12837	0.12353	-0.37946	-0.25491	0.77679	1.15378	ac6

NM_001174102	PRR7	0.00588	-0.28323	0.35368	0.33382	0.62274	1.15254	ac6
NM_012181	FKBP8	0.00805	-0.25369	-0.25215	-0.26474	0.72217	1.15215	ac6
NM_022552	DNMT3A	-0.62526	0.07191	1.00879	1.03785	0.83343	1.15200	ac6
NM_207121	FAM110A	-0.05581	-0.17696	0.02549	-0.52877	0.86381	1.15174	ac6
NM_174983	MFSD12	0.15202	0.25298	0.59987	0.72861	0.70985	1.15104	ac6
NM_015470	RAB11FIP5	0.03129	0.20680	0.57913	0.27085	0.82676	1.15052	ac6
NM_001252386	TNIP1	-0.92072	0.13916	0.89900	0.77327	0.72231	1.14997	ac6
NM_005572	LMNA	0.08229	-0.11792	0.12713	0.11775	0.80834	1.14979	ac6
NM_002342	LTBR	0.03856	-0.01052	0.10244	0.08215	0.83065	1.14965	ac6
NM_001010854	TTC7B	-0.10449	-0.16899	-0.02106	-0.02258	1.07683	1.14864	ac6
NM_003040	SLC4A2	-0.11014	-0.22177	-0.02501	-0.43465	0.68902	1.14803	ac6
NM_023076	UNKL	0.53746	-0.03250	0.95695	0.44078	0.33768	1.14744	ac6
NM_003302	TRIP6	-0.01886	0.05365	0.24422	-0.27522	0.61422	1.14733	ac6
NM_001207062	C22orf23	-0.34408	-0.11263	-0.42897	-0.55962	0.86103	1.14699	ac6
NM_213560	PKN1	0.08316	-0.17303	-0.46684	-0.24453	1.06954	1.14697	ac6
NM_024667	VPS37B	0.44895	0.84853	0.64075	0.22698	0.78837	1.14614	ac6
NR_036515	LOC284454	0.64700	1.03407	-0.04995	0.23875	0.79405	1.14426	ac6
NM_001122957	BCKDK	-0.13159	0.13031	0.12456	-0.38653	0.43921	1.14305	ac6
NM_002503	NFKBIB	0.19096	0.39588	0.75379	0.78738	0.26621	1.14272	ac6
NM_001112726	KIAA0284	0.35116	0.59417	0.82664	0.64334	0.64433	1.14210	ac6
NM_001130848	PITPNM1	-0.38186	-0.10216	-1.79873	-0.53831	-1.08365	1.14101	ac6
NM_003595	TPST2	0.14054	0.49717	0.91723	0.57130	0.83209	1.14017	ac6
NR_027765	SBF1P1	0.20464	0.16878	0.15293	0.05790	0.83161	1.14000	ac6
NR_037929	SLMO2-ATP5E	0.03804	0.48316	0.69997	0.91582	0.98590	1.13769	ac6
NM_002972	SBF1	0.05061	0.05097	0.20528	-0.01748	0.74023	1.13712	ac6
NM_004263	SEMA4F	0.03809	-0.34591	-0.13485	-0.32498	0.93472	1.13521	ac6
NM_018064	AKIRIN2	0.32161	0.89989	0.99522	0.85702	1.06025	1.13457	ac6
NM_021991	JUP	0.04153	0.03643	0.24970	0.14326	0.76985	1.13372	ac6
NR_002312	RPPH1	0.21812	0.65104	0.07539	0.50200	0.85746	1.13299	ac6
NM_001131025	PEX5	0.34319	0.50155	0.89881	0.26481	0.44665	1.13143	ac6
NM_001098207	HNRNPF	1.04492	-0.36884	-0.65193	-0.40472	-0.07378	1.13133	ac6
NM_178511	PRR24	-0.01922	-0.25722	0.60916	0.61243	0.49886	1.13011	ac6
NM_005781	TNK2	0.10062	0.10356	0.32865	-0.47390	0.76650	1.13006	ac6
NM_004481	GALNT2	-0.15175	-0.30295	0.04578	0.29595	0.93840	1.12988	ac6
NM_182533	C1orf86	0.34100	0.08990	0.22084	0.43785	0.78338	1.12827	ac6
NM_006745	MSMO1	0.46553	0.19090	0.64847	0.73785	0.91151	1.12825	ac6
NM_013332	HILPDA	-0.10175	0.06356	0.50808	0.44887	0.86191	1.12713	ac6
NM_182557	BCL9L	0.01064	0.25393	0.24353	0.05564	0.81129	1.12392	ac6
NM_052972	LRG1	-0.07404	-0.23932	0.11616	-0.18558	0.54932	1.12361	ac6
NM_000245	MET	0.00349	0.10155	0.16972	0.30131	1.01691	1.12099	ac6
NR_002556	LOC388242	0.02945	0.44499	0.58177	0.67085	0.66429	1.12033	ac6
NM_023083	CAPN10	0.05475	0.12888	0.22491	0.02867	0.61026	1.11495	ac6
NM_001102605	IMPDH1	0.25606	-0.21890	-0.08607	0.44390	0.72661	1.11467	ac6
NM_001164094	COPS7A	-0.07070	-1.05571	-0.21892	0.21660	0.79824	1.11356	ac6
NM_198042	PDLIM2	0.20370	0.54181	0.94331	0.93146	0.64773	1.11249	ac6
NM_006227	PLTP	0.77203	0.27159	-0.25373	0.23393	0.98889	1.11204	ac6
NM_001080401	PPM1N	0.11681	-0.20118	-0.07415	-0.50591	0.53207	1.11092	ac6

NM_014963	SBNO2	0.19947	-0.05770	0.12332	0.04667	0.69741	1.10666	ac6
NM_001195479	TFG	-0.05459	0.17098	0.57991	0.52169	0.94570	1.10559	ac6
NM_020904	PLEKHA4	-0.06948	-0.18640	-0.38658	-0.58425	0.89230	1.10438	ac6
NM_025233	COASY	-0.26690	-0.53152	-0.10793	-0.06075	0.67600	1.10326	ac6
NM_001010982	AFMID	0.03258	-0.24879	-0.23532	-0.84514	0.63926	1.10278	ac6
NM_021933	MIIP	0.21734	0.19743	-0.13560	0.00637	0.70614	1.10161	ac6
NM_001206665	IGSF8	0.26851	0.17089	-0.17191	-0.25297	0.76701	1.10155	ac6
NM_006871	RIPK3	-0.04353	-0.13495	-0.05477	-0.91691	0.46409	1.10038	ac6
NM_053275	RPLP0	0.35927	-0.12758	-0.56535	0.32804	0.48158	1.09598	ac6
NM_018284	GBP3	-0.34984	0.09533	0.01646	0.20041	0.97469	1.09594	ac6
NR_028491	TMEM41B	0.22211	0.15904	-0.13121	0.87761	0.30659	1.09580	ac6
NM_020433	JPH2	0.09054	0.17175	0.05093	0.00756	0.59543	1.09489	ac6
NM_002859	PXN	-0.11391	-0.05072	0.47581	0.66476	0.68581	1.09425	ac6
NM_020912	FLYWCH1	0.09755	0.42320	-0.35174	-0.88585	0.60856	1.09289	ac6
NM_001002252	ARL6IP4	0.00143	-0.14608	0.35491	0.06206	0.36603	1.09239	ac6
NM_025225	PNPLA3	0.02846	0.08755	0.59200	0.49548	0.77172	1.09233	ac6
NM_000499	CYP1A1	-0.21558	-0.11239	-0.55757	-0.53718	1.08233	1.09189	ac6
NM_001136202	ISOC2	-1.02950	-0.42133	-0.09516	-0.33199	-0.32527	1.09178	ac6
NM_014994	MAPKBP1	0.34390	0.28326	0.56770	0.56900	0.84670	1.09122	ac6
NM_001009944	PKD1	0.22393	0.54400	0.75184	0.52208	0.78881	1.08973	ac6
NM_006357	UBE2E3	0.16240	0.09156	0.27166	0.64576	1.04218	1.08875	ac6
NR_003262	FDPSL2A	-0.16196	-0.38028	0.23472	0.27399	0.89942	1.08718	ac6
NM_001103175	CCDC64B	0.27558	0.29777	0.12943	-0.39361	0.69873	1.08717	ac6
NM_002319	LRCH4	0.08273	0.14786	-0.13179	-0.46627	0.70274	1.08646	ac6
NM_138689	PPP1R14B	0.08047	-0.18437	-0.05425	0.00912	0.61535	1.08489	ac6
NM_001188	BAK1	-0.13143	-0.27729	0.22617	0.20331	0.71089	1.08469	ac6
NR_028038	GATS	0.06691	-0.32203	-0.34607	-0.50394	0.98432	1.08453	ac6
NM_014798_1	PLEKHM1	-0.36547	-0.52968	-0.29378	-0.69756	0.55795	1.08397	ac6
NM_006640	42987	-0.25923	-0.35693	-0.03999	0.28968	0.82459	1.08295	ac6
NM_001195733	PIP5K1C	-0.08169	-0.07484	-0.46542	-1.28307	0.63989	1.08269	ac6
NM_006643	SDCCAG3	0.11177	0.33521	-0.57987	0.15284	0.66676	1.08217	ac6
NM_021242	MID1IP1	0.14804	-0.43787	-0.33876	-0.31250	0.52444	1.08162	ac6
NM_000735	CGA	0.50696	0.23975	0.55377	0.83428	0.93270	1.08136	ac6
NR_036623	DBF4B	-0.09457	-0.19235	-0.14708	-0.26254	0.76580	1.07977	ac6
NM_024735	FBXO31	0.31965	0.69188	0.40331	0.24389	0.60292	1.07899	ac6
NM_001099673	C8orf59	0.35533	0.03676	0.23263	1.07295	0.74368	1.07774	ac6
NM_005641	TAF6	-0.02824	-0.35015	-0.11749	-0.57919	0.77271	1.07682	ac6
NM_021975	RELA	0.11914	0.19850	0.34652	0.06495	0.67275	1.07629	ac6
NM_024070	PVRIG	0.08125	0.61299	0.24291	0.13960	0.70926	1.07543	ac6
NM_001032279	RCE1	-1.08415	0.49296	0.26727	0.57559	-0.40787	1.07540	ac6
NM_173209	TGIF1	1.02845	0.52810	0.09773	0.14948	0.89798	1.07533	ac6
NM_138440	VASN	0.32185	0.68985	0.46028	0.11189	0.69916	1.07447	ac6
NM_002898	RBMS2	-0.07585	-0.16512	-0.12777	-0.11363	0.97898	1.07372	ac6
NM_004427	PHC2	-0.10284	-0.12106	0.18896	0.15996	0.71168	1.07370	ac6
NM_003789	TRADD	0.12078	-0.16644	-0.28302	-0.25310	0.62178	1.07363	ac6
NM_001146019	TJAP1	-1.18932	-0.75174	-1.27371	-1.98034	-0.30679	1.07330	ac6
NM_003780	B4GALT2	0.02114	0.03730	0.55700	0.10282	0.53798	1.07264	ac6

NM_052880	PIK3IP1	-0.65512	-0.76276	-0.65693	-0.62679	0.92346	1.07237	ac6
NM_001747	CAPG	0.82320	0.28074	0.26094	0.55050	-0.83349	1.07229	ac6
NM_001790	CDC25C	-0.14982	-0.61273	-0.65746	-1.23687	0.83971	1.07123	ac6
NM_152926	CPNE1	0.41427	-1.02801	-0.74797	-3.58974	0.30252	1.06989	ac6
NM_001099409	EHBP1L1	-0.17179	-0.13812	0.23209	0.05428	0.91483	1.06900	ac6
NM_138457	FOXP4	0.30315	0.27699	0.61999	0.47181	0.64733	1.06847	ac6
NM_001139457	BCAP31	0.15739	-0.04021	0.34691	0.57884	0.70336	1.06822	ac6
NM_001135055	TKT	-0.06006	-0.15468	-0.08225	0.02234	0.58183	1.06660	ac6
NM_014940	MON1B	0.10504	0.41755	0.63076	0.23710	0.73693	1.06659	ac6
NM_001145526	AFMID	-0.05266	0.21936	-0.33388	-0.20943	0.96794	1.06637	ac6
NM_130781	RAB24	-0.16178	-0.06479	-0.16707	-0.63843	0.17547	1.06584	ac6
NM_001069	TUBB2A	0.19052	0.23726	0.71076	0.66775	0.77538	1.06459	ac6
NM_031459	SESN2	0.64056	0.94332	1.04213	0.69687	0.73582	1.06451	ac6
NM_001042388	PPP4R1	-0.99489	-0.17809	0.74167	0.95157	0.96975	1.06401	ac6
NM_001110792	MECP2	0.02859	0.08509	0.07117	-0.28258	0.69306	1.06388	ac6
NM_001085365	MZT2A	0.21133	-0.12619	-0.03409	0.04170	0.75207	1.06329	ac6
NM_207380	C15orf52	-0.09918	-0.22927	0.07553	0.35998	0.92988	1.06209	ac6
NM_182517	C1orf210	0.05282	-1.81546	-0.70825	-0.06372	0.00024	1.06008	ac6
NM_001083601	NAA60	0.12675	-0.37072	0.74719	0.30812	0.76426	1.05991	ac6
NM_005393	PLXNB3	0.12233	0.15712	0.05860	-0.13888	0.54694	1.05820	ac6
NM_054013	MGAT4B	-0.06517	0.54013	0.68745	0.46757	0.88814	1.05815	ac6
NM_001134337	RNF24	0.29544	0.16193	0.88379	0.43653	1.02336	1.05770	ac6
NM_130786	A1BG	0.28452	0.16208	-0.29147	0.12710	0.61905	1.05758	ac6
NM_014772	CTIF	0.35814	-0.55027	0.03586	-0.28724	-0.03240	1.05635	ac6
NM_005745	BCAP31	0.53914	0.34678	0.62447	-0.02972	0.49142	1.05585	ac6
NM_001135944	MADD	0.91239	-0.95992	-1.04564	-0.82165	0.77586	1.05510	ac6
NM_152600	ZNF579	-0.04218	-0.04082	-0.04002	-0.04461	0.76476	1.05497	ac6
NM_182924	MICALL2	0.17450	0.33821	0.45895	0.64627	0.74681	1.05484	ac6
NM_001014795	ILK	0.69773	-0.27436	0.80979	-0.13527	0.35861	1.05422	ac6
NM_007121	NR1H2	0.06003	-0.04700	0.00005	-0.16854	0.63266	1.05369	ac6
NM_001242713	LOC10028956	0.32407	0.16643	0.59907	0.63297	0.04271	1.05302	ac6
NR_027713	KRT8P41	-0.04397	0.03712	0.40008	0.06923	0.71853	1.05282	ac6
NM_002868	RAB5B	-0.59587	-0.53938	-0.46893	-0.63845	-0.31491	1.05244	ac6
NM_003942	RPS6KA4	-0.00140	-0.31557	0.09928	0.14536	0.39868	1.05202	ac6
NR_023383	DTX2P1-UPK3	0.09281	-0.41898	-0.00320	0.04373	0.83041	1.05133	ac6
NM_001130099	KIFC3	0.05387	0.02730	-0.78467	-1.08914	0.64860	1.05062	ac6
NR_004407	RNU11	-1.80293	-2.31581	-1.57146	-1.63500	-0.25016	1.05050	ac6
NM_016578	RSF1	0.10927	0.30455	0.13784	0.49183	1.02386	1.04896	ac6
NM_001040668	BCL2L12	-0.84001	0.09341	-0.24169	-0.28107	0.79373	1.04842	ac6
NM_080388	S100A16	0.12783	0.01874	0.35191	0.70226	0.74844	1.04840	ac6
NM_014400	LYPD3	0.17391	0.15131	0.27631	0.29699	0.87561	1.04820	ac6
NM_019089	HES2	-0.31132	-0.38691	-0.99457	-0.97848	0.91971	1.04816	ac6
NM_003564	TAGLN2	0.03275	-0.15741	-0.04359	-0.11319	0.47421	1.04724	ac6
NM_005167	PPM1J	0.18435	-0.09802	0.58739	0.54187	0.73748	1.04669	ac6
NM_014316	CARHSP1	0.03248	-0.25122	0.08552	0.16693	0.82308	1.04563	ac6
NM_174919	ARHGAP27	0.27958	0.14923	0.27615	0.08171	0.51625	1.04558	ac6
NM_001145023	SCRN2	-0.23419	-0.21764	0.30559	0.30762	0.21714	1.04439	ac6

NM_024309	TNIP2	-0.10910	-0.07523	0.20789	0.18914	0.75370	1.04429	ac6
NM_001256120	NAA10	-0.01546	-0.33014	0.15327	-0.33222	0.57362	1.04265	ac6
NM_002654	PKM2	0.08767	-0.92132	0.06520	0.17708	0.58382	1.04164	ac6
NM_004383	CSK	0.15492	0.23374	0.15366	-0.17058	0.68801	1.04093	ac6
NM_015889	MED15	-0.18424	-0.02126	0.47960	0.00112	0.73456	1.03978	ac6
NM_018411	HR	-0.79925	-0.00338	0.45119	-0.19836	0.54980	1.03848	ac6
NM_032037	TSSK6	0.13671	-0.21734	-0.21785	-0.25753	0.71469	1.03777	ac6
NM_001102	ACTN1	0.05170	0.14907	0.68304	0.58709	0.86450	1.03690	ac6
NM_175055	HIST3H2BB	-0.43619	-0.41412	0.08657	-0.09386	0.30907	1.03626	ac6
NM_002890	RASA1	-0.38645	-0.58608	-0.80706	-0.50312	-0.24199	1.03398	ac6
NM_198317	KLHL17	0.09629	0.19247	0.07313	-0.29740	0.74910	1.03356	ac6
NM_014931	PPP6R1	-0.08660	-0.09133	0.16998	0.10836	0.66008	1.03118	ac6
NM_001256265	FLII	0.45619	0.09506	0.48283	0.35167	0.58808	1.03091	ac6
NM_000358	TGFBI	0.02920	0.03382	0.20373	-0.05765	0.90581	1.03065	ac6
NM_015577	RAI14	-0.20179	-0.53913	0.34722	0.40495	0.96230	1.03036	ac6
NM_014603	CDR2L	-0.06984	-0.31360	-0.48310	-0.92023	0.62447	1.02981	ac6
NM_001271	CHD2	-0.15584	0.38689	-0.06297	0.19540	0.96232	1.02903	ac6
NM_138763	BAX	0.11909	0.06126	0.11676	-0.27196	0.47682	1.02835	ac6
NM_207397	CD164L2	0.71649	0.32442	0.79084	0.87775	0.64509	1.02818	ac6
NM_003025	SH3GL1	0.07605	-0.12609	0.27441	0.42870	0.57459	1.02747	ac6
NM_182981	OSGIN1	-0.18798	-0.13743	0.48938	0.19103	0.58505	1.02742	ac6
NM_138383	MTSS1L	0.08846	0.06023	-0.30246	-0.76982	0.60747	1.02645	ac6
NM_001001414	NCCRP1	0.11784	-0.46105	0.04992	0.42146	0.88692	1.02611	ac6
NM_005558	LAD1	-0.07215	0.01309	0.59078	0.60064	0.68906	1.02588	ac6
NM_002415	MIF	0.30608	-0.24699	-0.29079	0.26295	0.75542	1.02488	ac6
NM_016535	ZNF581	-0.43219	0.12663	0.46241	0.33354	0.89093	1.02414	ac6
NM_001141969	DAXX	-0.73815	-0.76303	0.35648	-0.26240	0.17347	1.02343	ac6
NM_001084	PLOD3	-0.10836	-0.15285	0.02209	-0.19913	0.61283	1.02331	ac6
NM_001039877	STRN4	0.54574	0.55686	0.61245	0.69642	0.47047	1.02293	ac6
NM_012068	ATF5	-0.41356	-0.28587	0.14636	0.38762	0.53290	1.02268	ac6
NM_001113756	TYMP	-0.40660	-0.36209	-0.16855	-0.33733	0.91574	1.02265	ac6
NM_018677	ACSS2	0.20084	-0.19326	0.13788	0.36267	0.78740	1.02231	ac6
NM_004517	ILK	-0.69817	-0.77355	-0.86489	0.15047	0.82317	1.02205	ac6
NM_006509	RELB	0.27797	0.58903	0.84755	0.52625	0.74629	1.02179	ac6
NM_003560	PLA2G6	-0.46425	0.42999	0.26110	-0.25326	0.83848	1.02149	ac6
NM_007144	PCGF2	0.09386	-0.11364	-0.43272	-0.48044	0.76544	1.02082	ac6
NM_001130448	C15orf62	-0.59210	-0.53621	-0.46015	-0.39012	0.65812	1.02036	ac6
NM_012142	CCNDBP1	0.05296	-0.09614	0.04847	0.44838	0.95956	1.02016	ac6
NM_014718	CLSTN3	0.06485	-0.19720	-0.34571	-0.34163	0.89658	1.01987	ac6
NM_001008712	RBPMS	0.11661	-0.26461	-0.04085	0.12665	0.77352	1.01958	ac6
NR_024019	FAM193B	0.00632	0.04771	0.26006	-0.21615	0.67731	1.01954	ac6
NM_001145338	ZBTB22	0.17543	0.59022	-0.59567	-1.03424	0.18080	1.01778	ac6
NM_021932	RIC8A	-0.00829	-0.15544	0.00742	-0.15742	0.55244	1.01516	ac6
NM_002011	FGFR4	-0.05158	0.17349	0.24506	-0.19573	0.53476	1.01389	ac6
NM_002198	IRF1	0.85478	0.63981	0.40812	0.25880	0.68161	1.01383	ac6
NM_014938	MLXIP	-0.01024	0.00315	0.37238	0.41303	0.62670	1.01348	ac6
NM_001142444	EDC3	-1.02090	0.33983	-0.29937	0.15018	0.43265	1.01201	ac6

NM_032819	ZNF341	-0.14074	-0.50403	-0.52169	-0.27947	0.74338	1.01184	ac6
NM_152557	ZNF746	0.84868	0.52956	0.84166	0.68147	0.50746	1.00978	ac6
NM_002149	HPCAL1	0.08691	-0.08995	0.26646	0.44302	0.63307	1.00932	ac6
NM_001146702	KDM5C	0.19588	-0.09101	0.35522	0.16506	0.58955	1.00915	ac6
NM_001961	EEF2	0.04604	-0.05592	0.06536	-0.08040	0.69804	1.00810	ac6
NM_025204	TRABD	0.11468	0.09440	0.05456	0.08486	0.68611	1.00803	ac6
NM_006779	CDC42EP2	0.38222	0.89861	0.58728	0.11282	0.78846	1.00661	ac6
NM_001042423	SLC16A3	0.44917	0.08977	-0.56034	-0.39192	0.53762	1.00654	ac6
NM_004286	GTPBP1	0.05899	0.15051	0.33587	0.29466	0.83605	1.00573	ac6
NM_003765	STX10	-0.04411	-0.29996	-0.11100	-0.09761	0.52363	1.00509	ac6
NM_030916	PVRL4	-0.09727	-0.25654	-0.47955	-0.78934	0.69847	1.00477	ac6
NM_006218	PIK3CA	0.01806	0.45715	0.50745	0.84932	0.93594	1.00382	ac6
NM_018216	PANK4	-0.08328	-0.16161	-0.21278	-0.30178	0.87365	1.00257	ac6
NM_021978	ST14	-0.04921	-0.03706	0.19155	0.07622	0.50997	1.00230	ac6
NM_001134776	KLC2	-0.04442	0.14553	0.28520	-0.13518	0.78499	1.00228	ac6
NM_014064	METTL11A	0.04897	-0.18511	-0.01818	0.20861	0.71130	1.00207	ac6
NM_002446	MAP3K10	0.17610	0.32210	0.20538	-0.07549	0.73813	1.00172	ac6
NM_005526	HSF1	0.09941	0.04567	0.13189	0.03178	0.64525	1.00094	ac6

* Activated genes cluster
