# Supplementary material for: Transcriptomic and ChIP-sequence interrogation of EGFR signaling in HER2+ breast cancer cells reveals a dynamic chromatin landscape and S100 genes as targets
Source: BMC Med Genomics. 2019 Feb 8;12:32. doi: 10.1186/s12920-019-0477-8 (PMC6368760; doi:10.1186/s12920-019-0477-8)
Supplement: Supplementary file 3 — Table S2. Log2 ratios of repressed genes listed by cluster. (PDF 2231 kb) [file 12920_2019_477_MOESM3_ESM.pdf]

Table S2. Log2 ratios of repressed genes listed by cluster

test_id	gene_id	1h/unt	2h/unt	4h/unt	6h/unt	16h/unt	24h/unt	cluster*
NM_001199199	AK2	-3.05304	0.234966	0.226558	-0.9268	-0.87761	-2.87964	rc1
NR_000008	SNORD22	-2.95184	-0.40018	-1.27784	-0.3308	-1.10902	0.02296	rc1
NM_001889	CRYZ	-2.68063	-0.04456	-0.23167	-2.35488	-0.18389	-0.04392	rc1
NM_022367	SEMA4A	-2.59939	-0.34549	-0.52332	-1.66621	0.093899	0.27263	rc1
NM_001195736	FAM213B	-2.34475	-0.95786	-2.02382	-2.09484	-0.64078	-0.04530	rc1
NM_001128833	ZBTB4	-2.33878	-0.57585	0.243796	-0.86203	1.290492	1.51034	rc1
NM_001128848	SMARCA4	-2.27289	-0.35204	-0.33715	-0.59695	-0.45497	-0.30098	rc1
NM_000920	PC	-2.19463	-1.53703	-1.69419	-0.93247	-0.09985	-0.02007	rc1
NM_145165	CHURC1	-2.13851	-0.06056	-1.00667	0.77729	0.271294	-1.14443	rc1
NM_001199262	UCHL5	-2.11119	-0.61008	-0.54876	0.392365	-1.70621	-1.53525	rc1
NM_001083113	ZGPAT	-2.07696	0.357156	1.414933	1.100655	-0.02954	-1.29505	rc1
NM_018320	RNF121	-2.06797	-1.89217	0.100873	-0.51037	-1.92726	-0.10879	rc1
NM_001024211	S100A13	-2.06085	-0.13515	0.092998	0.629522	0.218691	0.29017	rc1
NR_027391	SLC15A3	-2.05059	-0.00629	-1.09033	-0.38677	-0.95112	-0.60850	rc1
NM_001242488	KIAA0913	-2.0137	-1.70639	-0.21909	-0.13335	0.175537	0.32555	rc1
NM_001203263	IL17RC	-1.95726	-1.2544	-0.69828	-1.27717	-0.66397	0.01789	rc1
NM_001002878	THOC5	-1.88897	-1.14657	-1.44969	-1.31211	-0.38479	0.12823	rc1
NM_001130850	CAB39	-1.88896	0.802759	2.081179	2.485794	0.362118	-1.22369	rc1
NM_019862	ABCC1	-1.83437	0.438629	0.474881	0.402619	-1.22632	-0.01485	rc1
NM_001011668	CHCHD7	-1.80771	-0.9937	0.237118	0.200668	-1.25569	0.00678	rc1
NR_045607	UXS1	-1.80392	0.069253	-0.62355	-0.59247	-0.00875	-1.61569	rc1
NM_181359	IL6R	-1.7815	0.062178	0.826658	1.443113	-1.36908	0.89990	rc1
NM_001184794	PARD3	-1.77575	-0.35888	-0.35992	-0.64735	0.603886	0.16664	rc1
NM_001242481	EIF1AD	-1.7429	-0.88743	1.375451	-0.60734	0.450801	0.06404	rc1
NM_015604	DCAF4	-1.73568	0.260548	0.742173	0.713873	-1.01347	-0.27092	rc1
NM_013448	BAZ1A	-1.73385	0.354145	0.509233	0.629548	-0.50456	-0.08994	rc1
NM_001099670	C8orf59	-1.72845	-0.31399	-0.97924	-0.24813	-1.04176	0.64175	rc1
NM_001163323	CCDC120	-1.70605	1.247163	1.157557	0.340663	0.659414	0.73773	rc1
NM_016335	PRODH	-1.65591	-0.28294	-0.23281	-1.55944	-1.22545	-0.60943	rc1
NM_001005741	GBA	-1.65353	-1.47183	0.38562	1.233336	0.528011	0.12827	rc1
NM_003347	UBE2L3	-1.64744	-0.80692	-0.59156	0.651972	-0.84211	-1.01166	rc1
NM_001142587	NFYC	-1.61496	0.625049	0.517978	-0.25057	0.133021	0.48395	rc1
NM_001007269	GEMIN7	-1.61392	-1.14591	-1.40971	0.078149	-0.87749	0.70203	rc1
NM_001136158	OTUD5	-1.59703	-0.89236	0.544016	0.482634	0.622267	0.75922	rc1
NM_001199534	C20orf24	-1.59622	-1.00687	-0.88494	-0.30461	-0.72521	0.03094	rc1
NM_001004722	NCK2	-1.57906	0.278562	-0.32253	-0.01595	-0.19809	-0.98096	rc1
NM_001198615	MAP7	-1.57682	0.101015	0.396238	-0.10246	0.453643	0.27526	rc1
NM_017652	ZNF586	-1.55774	0.230219	0.860331	0.564362	-0.74686	0.08575	rc1
NM_001039467	RGS19	-1.55541	-0.35952	-1.01455	-0.06943	0.090519	0.18358	rc1
NM_001170804	LARP4	-1.53537	0.376544	-0.34399	1.100062	0.1121	-0.79754	rc1
NM_032647	CBX2	-1.53389	-0.23159	-0.23399	0.081426	0.316174	0.16476	rc1
NR_002450	SNORD68	-1.5176	-0.28722	0.460992	-0.41442	-0.74246	-0.71252	rc1
NM_153818	PEX10	-1.51274	0.278522	-0.9375	-0.19517	-0.55092	-0.56495	rc1
NM_182913	SYNE2	-1.50166	-0.08291	-0.48327	-0.93115	-1.01292	-0.21831	rc1
NM_001197126	IRF3	-1.49719	1.256765	0.906291	0.538143	0.642249	2.53361	rc1
NM_201437	TCEA1	-1.48837	0.771782	-0.23641	0.695876	-0.67368	-0.54277	rc1

NM_001195628	MLLT10	-1.48301	-0.13769	0.134591	-0.59281	-0.55364	0.24848	rc1
NM_147777	SNX15	-1.45743	-0.6445	-0.69695	-1.42326	-0.95357	-0.55351	rc1
NM_001012732	DCTD	-1.45604	0.342425	1.011697	1.477739	0.383941	1.17036	rc1
NR_004858	POFUT2	-1.45415	-0.37141	-0.5531	-0.02799	-1.22142	-0.84156	rc1
NM_130472	MADD	-1.45377	0.036445	0.011925	-0.83513	-0.78892	-0.75171	rc1
NM_031361	COL4A3BP	-1.435	-0.19643	0.184188	0.10248	-0.6403	-0.48678	rc1
NM_145288	ZNF296	-1.41951	-0.85519	-0.73552	-0.51544	-0.68891	-0.40571	rc1
NM_006372	SYNCRIP	-1.41074	-0.4544	-0.00375	0.123436	-0.09322	-0.03536	rc1
NM_001025237	TSPAN4	-1.39609	0.913336	-0.25788	1.548031	-0.8603	1.32076	rc1
NM_182580	CYB561D1	-1.38945	-1.28778	-0.11276	0.406604	-0.24204	0.79777	rc1
NM_001001520	HDGFRP2	-1.37486	-0.08412	-0.69858	-0.96977	-1.26888	0.41718	rc1
NM_033306	CASP4	-1.37193	-0.77984	0.553094	0.592402	-0.42332	0.84750	rc1
NM_001196	BID	-1.37146	-0.00272	0.887805	0.655245	1.064938	1.72373	rc1
NM_198310	TTC8	-1.34228	-0.77422	-1.06392	-0.2999	-0.37786	-1.29488	rc1
NM_001122823	GTF3C5	-1.33942	-0.4093	0.492837	-0.31529	0.66675	1.30325	rc1
NM_001142397	CTIF	-1.33924	0.823725	0.62324	-0.1149	1.179285	0.67476	rc1
NM_183043	RNF6	-1.32077	-0.25954	0.301404	0.385022	-0.90038	-0.27890	rc1
NM_001252269	FAM69A	-1.32069	0.208093	0.872076	0.15324	0.30173	0.31559	rc1
NM_178221	ATG4C	-1.27954	-0.88419	0.197174	0.185211	0.381313	-1.00473	rc1
NM_024755	SLTM	-1.25863	-0.15437	0.232376	0.484707	0.438397	0.35176	rc1
NM_006482	DYRK2	-1.2538	-0.87463	0.965606	0.616418	0.397456	-0.87357	rc1
NR_002821	LOC400927	-1.24919	0.300695	-0.0482	0.359957	0.970815	0.80198	rc1
NM_014888	FAM3C	-1.24621	-0.01409	1.106937	1.046164	1.762663	2.04138	rc1
NM_017991	KANSL3	-1.22777	-0.17756	-0.33691	-0.38031	-0.07776	0.40668	rc1
NM_181659	NCOA3	-1.22326	-0.68999	-0.76159	0.439243	0.86999	0.01567	rc1
NM_198046	ZDHHC16	-1.22094	-0.73776	-0.15395	-1.05214	0.644757	0.74500	rc1
NM_002512	NME2	-1.2126	0.928394	1.805028	-0.75955	2.412471	3.90498	rc1
NM_001012339	DNAJC21	-1.20943	-0.06682	-0.37271	-1.06617	-0.65179	-0.79584	rc1
NM_014283	C1orf9	-1.20267	0.419713	-0.38848	-0.89026	-0.08833	0.19386	rc1
NM_005186	CAPN1	-1.1966	-0.4478	-0.3481	-0.07754	-0.04886	0.91577	rc1
NM_000883	IMPDH1	-1.19285	-0.06524	-0.49685	-0.58948	0.313406	-0.05531	rc1
NM_001142618	STRA6	-1.18752	-0.59969	-0.302	1.109922	0.919118	0.77801	rc1
NR_003005	SCARNA4	-1.18526	-0.40891	-0.15541	-1.14445	0.656733	1.24119	rc1
NM_032527	ZGPAT	-1.17474	0.10832	0.142443	0.883452	0.938067	1.32854	rc1
NM_000874	IFNAR2	-1.16282	-0.46926	-0.52328	-0.41628	-0.65544	0.45344	rc1
NM_002434	MPG	-1.15431	0.114653	-0.55561	0.110755	0.961598	1.50347	rc1
NR_028352	PIGP	-1.14892	-0.67999	-0.87319	-0.11934	-0.10599	-0.56721	rc1
NM_001253794	LOC440335	-1.14717	-0.08929	0.006407	0.601862	0.302517	0.43655	rc1
NM_198490	RAB43	-1.14582	-0.05815	1.038091	0.105771	0.903692	0.89331	rc1
NM_001018055	BRCC3	-1.14379	0.286082	0.163625	0.005016	-1.09399	-0.71018	rc1
NM_005638_1	VAMP7	-1.13824	0.190771	-0.73143	0.383401	-0.72906	-0.06830	rc1
NM_001204467	RBM10	-1.13623	-1.09453	-0.49323	-0.87297	-0.66269	-0.62614	rc1
NM_001913	CUX1	-1.13083	-0.66709	-0.27262	-0.05073	-0.15001	0.16587	rc1
NM_022356	LEPRE1	-1.12537	-1.07236	-0.23558	-0.67147	-0.79707	-0.57608	rc1
NM_021117	CRY2	-1.12498	-0.91926	-0.40958	-0.34471	0.510232	-0.21184	rc1
NR_002746	SNORD47	-1.123	0.361086	-0.08111	0.374896	0.082597	-0.94072	rc1
NM_001171202	UBAP1	-1.12192	-0.21873	-0.05322	-0.1525	-0.90711	0.34568	rc1

NM_033355	CASP8	-1.09122	0.354362	-0.95716	-0.46001	-0.30438	-0.98766	rc1
NM_001135650	EEF1E1	-1.0904	0.181183	-0.04738	-1.07554	-0.26671	-0.58889	rc1
NM_133484	TANK	-1.08744	-0.37658	-0.03748	0.663738	0.480548	-0.46984	rc1
NM_001032279	RCE1	-1.08415	0.492964	0.267269	0.575587	-0.40787	1.07540	rc1
NM_001204062	COMMD3-BM	-1.07493	-0.16012	-0.1407	0.358035	-0.61636	-0.62665	rc1
NM_017768	LRRC40	-1.07236	-0.22184	0.011775	-0.06002	-0.89642	-0.61436	rc1
NR_027382	RDH13	-1.06806	-0.07397	-0.23032	-0.18634	-0.35649	-1.06387	rc1
NM_001025252	TPD52	-1.06739	-0.45263	0.662897	-0.09917	2.70422	2.63757	rc1
NM_001100588	RC3H2	-1.06396	-0.42942	0.092926	0.068091	0.270231	-0.22789	rc1
NM_001242837	AP2A2	-1.05519	-0.87359	-0.89135	-0.86432	-0.26854	-0.17222	rc1
NM_001100409	SENP6	-1.0495	-0.58262	-0.44159	-0.01929	0.154069	-0.34271	rc1
NM_005930	CTAGE5	-1.03865	0.223194	-0.88407	-0.16574	-0.34116	-0.85163	rc1
NM_001136202	ISOC2	-1.0295	-0.42133	-0.09516	-0.33199	-0.32527	1.09178	rc1
NM_001190987	SRSF11	-1.02623	-0.18306	-0.26262	0.118644	0.400781	0.38727	rc1
NM_001142444	EDC3	-1.0209	0.339834	-0.29937	0.150178	0.432649	1.01201	rc1
NM_001008485	SLC41A3	-1.01061	-0.22566	-0.14803	1.128894	-0.69069	-0.01068	rc1
NM_003003	SEC14L1	-1.00847	-0.72924	-0.70478	-0.35307	0.669761	-0.64620	rc1
NM_001199341	RPL17	0.358849	-4.50399	-3.53879	0.472291	-4.40104	0.30603	rc2
NM_001206799	PKM2	-1.24311	-3.89115	-0.36711	-0.52362	-0.75246	1.30986	rc2
NM_001135635	C11orf68	-0.72274	-3.50963	-1.78731	-2.41936	-2.02722	-1.55363	rc2
NR_003001	SCARNA7	-0.81563	-3.39987	-0.55685	-0.53278	-0.05102	0.48727	rc2
NM_001256533	MSTO1	-0.50772	-3.07352	-0.80971	-2.43067	-1.00626	-2.55653	rc2
NM_001025603	RFX5	-1.04488	-3.03836	-0.10396	-0.88679	-0.87317	-0.69071	rc2
NM_022105	DIDO1	-1.54474	-3.00667	0.352881	0.033896	-1.3838	-1.31219	rc2
NM_001099285	PTMA	0.612014	-2.80123	2.002397	-0.57242	0.134497	1.57311	rc2
NM_000546	TP53	-1.96969	-2.76909	-0.67048	-2.37633	-0.42497	-0.58718	rc2
NM_206861	TACC2	-1.00881	-2.71474	-0.98944	-1.66946	-1.68756	-1.54523	rc2
NM_007283	MGLL	-1.01414	-2.71378	0.218763	-0.09779	0.075243	0.44897	rc2
NM_052988	CDK10	-1.24167	-2.6647	-0.38749	-2.29623	-0.52914	-0.22456	rc2
NM_198045	ZDHHC16	-0.55981	-2.59577	-0.92267	-0.93316	-2.08333	-1.99724	rc2
NM_033486	CDK11B	1.277055	-2.52913	0.104409	0.781202	-0.69493	0.96860	rc2
NM_001184762	CLTA	0.821828	-2.44703	-0.612	-0.40229	-0.83415	-0.55235	rc2
NM_181455	MRPL55	-0.27981	-2.33638	-1.09571	-0.69266	0.5626	0.49446	rc2
NR_004407	RNU11	-1.80293	-2.31581	-1.57146	-1.635	-0.25016	1.05050	rc2
NM_022769	CRTC3	-0.22699	-2.31284	0.144216	-0.65995	0.913114	0.42842	rc2
NM_001256402	ELOVL1	-0.2502	-2.27663	-0.57832	-0.53498	-1.34063	-0.26626	rc2
NM_001042546	ATPAF1	-1.20829	-2.1865	-1.44696	-0.62125	-1.63564	-1.43216	rc2
NM_001204856	SCNM1	-0.46865	-2.16919	-0.02653	-0.32113	-0.68557	-0.63777	rc2
NM_001142298	SQSTM1	-1.71096	-2.11714	-0.73216	0.186163	-0.1014	1.20193	rc2
NR_045557	URI1	0.143289	-2.0913	0.934459	-0.01526	-0.21041	-1.66824	rc2
NM_001195752	THAP3	0.275676	-2.0777	-0.32529	0.200768	0.866991	0.89073	rc2
NR_003003	SCARNA17	-0.66042	-2.04272	-0.28471	-0.75701	-0.07315	0.77183	rc2
NM_001199319	PEX26	-0.98609	-2.04085	-1.20856	-0.29015	-0.72187	-0.51808	rc2
NM_014397	NEK6	-0.40038	-2.01547	-0.48105	0.889243	-0.71161	-0.00290	rc2
NR_039840	MIR4691	-0.16833	-2.00408	-1.53347	0.248214	-1.91478	-0.94422	rc2
NM_032797	AIFM2	-0.51083	-1.98956	-0.15232	-0.61152	0.425357	0.16253	rc2
NM_001039671	YIF1B	1.176765	-1.98747	-1.56696	1.972258	0.92846	-0.34552	rc2

NM_001199822	MDP1	-0.54872	-1.96081	-0.34899	-0.18268	-1.1695	-0.60727	rc2
NM_032471	PKIB	-0.16086	-1.93193	-0.10225	-0.92547	-0.38851	-0.67979	rc2
NM_001129765	NSDHL	0.092294	-1.91109	0.428332	0.93146	-0.77393	0.15395	rc2
NM_001256321	THTPA	-0.79577	-1.90926	-1.70185	0.668506	-1.03745	0.29064	rc2
NR_003025_1	SNORA59A	0.106027	-1.90808	-0.50004	-1.20568	0.259191	0.84406	rc2
NM_198970	AES	-0.03265	-1.8929	-0.52058	-0.80659	-1.24131	0.46164	rc2
NM_201281	MTMR2	-1.48697	-1.89154	0.252993	0.686322	-1.35485	-1.48022	rc2
NM_001017535	VDR	-0.23886	-1.86689	0.462674	-0.33201	-0.41227	-0.00846	rc2
NM_006472	TXNIP	-0.98006	-1.86429	-0.02934	0.033318	0.901879	0.79897	rc2
NM_001130065	MYO9B	-1.49157	-1.81608	-1.50821	-0.2626	-0.8007	-0.54554	rc2
NM_182517	C1orf210	0.052817	-1.81546	-0.70825	-0.06372	0.00024	1.06008	rc2
NM_001199281	CABIN1	-1.05064	-1.7741	-0.08589	-1.21994	-0.01199	-0.07850	rc2
NM_006312	NCOR2	-0.08029	-1.75753	-0.26909	-0.92383	-0.13886	-0.16751	rc2
NM_138777	MRRF	-1.22726	-1.75114	0.369065	0.221231	-1.16429	-1.08641	rc2
NM_001144037	TMEM25	-0.48268	-1.70331	-1.17134	-0.80847	-1.17144	-0.75247	rc2
NM_001134738	BCL6	-0.20045	-1.70277	-0.14902	0.169818	0.998117	0.66107	rc2
NM_001467	SLC37A4	0.168097	-1.66224	0.040769	0.544518	-0.80039	-1.27718	rc2
NR_045631	NFU1	-0.6683	-1.6546	-0.42518	-0.57046	0.023207	-1.35318	rc2
NM_032951	MLXIPL	-0.09018	-1.65271	-0.46559	-1.42641	-0.12725	-0.23107	rc2
NM_173717	ELAC2	-0.4681	-1.63627	0.110692	0.454854	-1.47566	0.23157	rc2
NM_001222	CAMK2G	-1.02137	-1.63557	-0.81104	-0.60076	-0.04285	0.30992	rc2
NM_001195556	CLINT1	-1.54057	-1.59819	-0.3674	-0.58377	-0.03846	0.31733	rc2
NM_080752	ZSWIM3	0.129824	-1.56498	-0.33638	-0.28597	0.67281	0.68083	rc2
NM_001005743	NUMB	-0.94639	-1.55502	-1.01998	-0.23342	0.329804	-0.80198	rc2
NM_001145662	GATA2	-0.41742	-1.55343	-0.31632	-0.38073	-0.99737	-0.75925	rc2
NM_001040443	PHF11	-1.29112	-1.53963	0.003288	-0.51757	0.310712	0.05358	rc2
NM_018107	RBM23	0.402316	-1.53299	-1.28536	-0.88576	0.567779	0.77110	rc2
NM_178000	PPP2R4	-0.01285	-1.52735	-0.92542	0.151509	0.732125	0.53540	rc2
NM_006756	TCEA1	0.430103	-1.5237	-0.16116	-0.68212	-0.1554	-1.19076	rc2
NM_003538	HIST1H4A	-0.59558	-1.52349	-1.15451	-1.41764	-1.34956	-0.71973	rc2
NM_001098507	ZNF207	-0.45495	-1.51978	0.616563	0.709368	0.316033	0.37619	rc2
NM_002561	P2RX5	-0.21302	-1.51737	0.145481	-0.04699	1.112574	0.72571	rc2
NR_036576	APTX	0.697808	-1.50755	0.790434	0.242085	-1.46296	-0.49591	rc2
NM_001164758	PRKAR1B	-1.2242	-1.50667	-0.54125	-0.62894	-0.89899	0.07686	rc2
NM_001099672	C8orf59	0.083633	-1.50323	0.246158	0.119644	-0.60707	0.90623	rc2
NM_001706	BCL6	0.338029	-1.48761	0.292303	-0.23343	1.55802	1.57964	rc2
NR_036565	CCT3	-1.20888	-1.4722	0.120455	-0.76387	-0.84751	-0.10494	rc2
NR_000007	SNORD73A	-0.67456	-1.45599	1.317314	-0.50013	-1.28019	0.77902	rc2
NM_001134774	KLC2	0.048793	-1.44892	0.489977	0.137155	0.789454	1.21876	rc2
NM_021131	PPP2R4	0.054831	-1.44429	-0.45012	0.059666	-0.01216	0.97750	rc2
NM_001014443	USP21	0.249409	-1.44351	-0.18085	-0.21768	-0.34819	0.72822	rc2
NM_183237	RNF7	-0.07766	-1.43485	0.161933	-0.90672	-0.27021	-0.07546	rc2
NM_024075	TSEN34	-0.85031	-1.42663	-0.46456	-0.86085	-1.05932	0.02221	rc2
NM_021944	C14orf93	0.306094	-1.42458	-0.60681	-0.58767	-1.20296	0.67028	rc2
NM_016362	GHRL	-0.06803	-1.41669	-0.27427	0.609779	0.093794	-0.70498	rc2
NM_001097590	RPP38	-0.99244	-1.39482	0.408343	-0.14107	0.009273	-0.07989	rc2
NM_002574	PRDX1	0.37086	-1.37763	-0.01905	0.491684	1.24608	1.48023	rc2

NM_015911	ZNF691	-0.26764	-1.37233	-0.89467	-0.46955	-1.06945	-1.01489	rc2
NM_003450	ZNF174	-0.40209	-1.36291	-0.12773	-0.00962	-0.14436	-0.22228	rc2
NM_001145773	GPR56	-0.3958	-1.35538	0.536641	0.350069	0.8988	2.21360	rc2
NM_032667	BSCL2	0.205875	-1.35078	-0.74774	1.015041	-0.0209	0.87552	rc2
NM_001190849	CNOT4	-0.11593	-1.34539	-1.29521	-0.34037	0.224285	0.73406	rc2
NM_015365	AMMECR1	-0.23803	-1.33884	-0.3008	0.940646	-0.01741	-0.79797	rc2
NR_029415	SHMT2	-0.21218	-1.33654	-0.82825	0.701871	-0.91757	-0.47178	rc2
NM_020860	STIM2	-0.22009	-1.33342	-0.49467	-0.35339	-0.61261	-1.14166	rc2
NM_005227	EFNA4	-0.25692	-1.32379	-0.61955	-0.54639	0.339413	0.56179	rc2
NM_018379	FAM63A	-0.22637	-1.32129	-1.14345	-0.79891	-0.62169	-0.89106	rc2
NM_001127585	ING4	1.273437	-1.31734	-0.513	0.001236	-0.61866	-0.41052	rc2
NM_016576	GMPR2	-0.00204	-1.31706	-0.11868	-0.04797	0.407494	0.25254	rc2
NM_005338	HIP1	-0.36004	-1.316	-0.27138	-0.58255	0.249149	0.31163	rc2
NM_001100119	XRCC3	1.14723	-1.31368	1.171895	-0.88722	0.384508	-0.39686	rc2
NM_152832	FAM89B	-0.03431	-1.30726	-0.13819	-0.84753	0.061832	0.67776	rc2
NM_001199877	SERF2	-0.00157	-1.30714	-0.37664	-0.12935	-0.38005	-0.83734	rc2
NM_138621	BCL2L11	-0.27334	-1.3068	-1.24064	-0.80744	-0.78897	-0.64968	rc2
NM_012237	SIRT2	-0.23441	-1.304	0.020966	0.173369	-0.44256	-0.39686	rc2
NM_004145	MYO9B	0.256732	-1.29998	0.194363	-0.72673	0.05748	0.20912	rc2
NM_012322	LSM5	-0.5371	-1.29886	-0.21722	0.407967	-0.45936	-0.73207	rc2
NM_003072	SMARCA4	-1.03259	-1.29587	-0.77233	-1.06453	0.044129	-0.15432	rc2
NM_001097640	FUT3	0.257385	-1.2953	0.224231	-0.30941	0.807353	0.77651	rc2
NR_037188	CPNE1	-0.26785	-1.29409	0.30288	0.075648	-0.35323	-0.45526	rc2
NM_001006639	TCEAL1	0.725589	-1.29034	0.619328	0.558602	0.678273	0.19616	rc2
NM_001100419	C19orf60	0.154803	-1.28521	-1.19887	0.400522	-0.01692	0.34651	rc2
NM_004927	MRPL49	-0.20677	-1.26684	-0.09802	-0.01525	-0.07244	0.11287	rc2
NM_032926	TCEAL3	-0.78485	-1.26025	-0.37262	-0.71272	-0.41084	-0.36875	rc2
NM_000404	GLB1	-0.99986	-1.25909	-0.27331	0.375015	-0.14956	-0.26196	rc2
NM_181746	CERS2	-0.42289	-1.25115	0.385258	0.660062	1.297325	1.95148	rc2
NM_001242777	TMEM139	-0.42801	-1.24543	-0.68846	-0.40825	-1.04371	-0.69189	rc2
NM_203500	KEAP1	-0.72373	-1.24124	-1.06942	-0.37579	-0.93604	-0.31280	rc2
NM_000655	SELL	-0.7667	-1.23906	-0.59206	0.023725	-0.84782	-1.13530	rc2
NM_017789	SEMA4C	-0.01817	-1.22111	-0.34808	-0.49406	0.729799	0.88383	rc2
NM_194358	RNF41	-1.01657	-1.21638	-0.17699	-0.9426	0.552194	-0.98520	rc2
NM_152268	PARS2	-0.12964	-1.21603	-0.46881	-0.33576	0.237564	0.38187	rc2
NM_001081003	COMMD5	-0.96771	-1.19965	-0.33079	-1.05738	-0.66295	0.22078	rc2
NM_001256169	APOM	-0.26105	-1.19687	-0.41221	0.627557	-0.06799	0.13198	rc2
NM_145112	MAX	0.281088	-1.19221	0.142883	-0.13116	0.109256	0.05764	rc2
NR_002918	SNORA48	0.568763	-1.19104	-0.37418	-0.61904	-0.93036	-0.52901	rc2
NM_003223	TFAP4	-0.26565	-1.18902	0.170086	0.439463	-1.03686	-1.04338	rc2
NM_006242	PPP1R3D	-0.14336	-1.18745	-0.78276	-0.31832	-0.42587	-0.68555	rc2
NM_030803	ATG16L1	-0.03903	-1.18365	1.143089	0.700757	-0.20928	-1.01270	rc2
NM_178120	DLX1	-0.01606	-1.18359	-0.99399	-0.8333	-0.23458	-0.44422	rc2
NM_001100598	ZNF707	-1.00636	-1.17793	-0.49391	-0.91882	-0.81804	-0.16622	rc2
NM_005585	SMAD6	0.179398	-1.17589	-0.99098	-0.52253	-0.71672	-0.96191	rc2
NM_022089	ATP13A2	-0.52523	-1.16215	-0.28955	-1.00315	-0.21364	0.95869	rc2
NM_152640	DCP1B	-0.02241	-1.16144	-0.42165	-0.112	-0.07284	0.05297	rc2

NM_001206916	CACNB3	-0.3025	-1.14973	0.293378	0.345146	-0.07008	-0.73355	rc2
NM_001165937	STARD3	0.665038	-1.14696	0.694361	0.350857	0.467677	0.51703	rc2
NR_028450	BCAT2	-0.33718	-1.13945	-0.61201	-0.5823	-0.04635	0.33583	rc2
NM_001167856	SBNO1	-0.99433	-1.13849	0.270553	0.186098	0.33833	-0.08922	rc2
NM_001136218	TMEM51	-0.76432	-1.13576	0.918919	1.428211	0.210261	1.47866	rc2
NM_018081	WRAP53	-0.9993	-1.13398	-0.02403	0.511126	-0.18681	-0.17987	rc2
NM_001077241	SLC25A45	-0.6753	-1.13115	1.288344	-0.08776	1.327265	0.46154	rc2
NR_000024	SNORD46	1.366999	-1.1135	0.844584	-0.74097	1.886968	-0.09978	rc2
NM_001173534	DGCR2	1.873844	-1.1133	1.214571	2.263548	-0.17215	0.56602	rc2
NM_001136036	ZNF692	-0.62006	-1.10948	-0.26542	-0.71208	-0.1919	-0.60617	rc2
NM_033375	MYO1C	-0.18579	-1.10638	0.047108	0.334972	-0.96338	-0.39092	rc2
NM_201434	RAB5C	-0.61115	-1.10534	0.006421	-0.40023	0.250502	0.88510	rc2
NM_022474	MPP5	-1.02812	-1.09684	-0.60605	-0.29017	0.244169	-0.36209	rc2
NM_001142418	MORF4L2	0.795541	-1.0786	1.067084	1.19712	0.62696	-0.42613	rc2
NM_198593	C1QTNF1	-0.493	-1.07638	-0.42011	-0.1645	1.093063	1.35056	rc2
NM_194317	LYPD6	0.304368	-1.07144	-0.21084	0.300305	-0.27782	-0.59821	rc2
NM_005189	CBX2	-0.2248	-1.06922	-0.36084	-0.36471	0.159478	0.49251	rc2
NM_003434	ZNF133	-0.03986	-1.06729	-0.08065	-0.05544	-0.07093	-0.04960	rc2
NM_001130964	PLCD1	-0.37189	-1.05885	-0.06604	-0.55566	0.397024	0.76081	rc2
NM_001164094	COPS7A	-0.0707	-1.05571	-0.21892	0.216601	0.798239	1.11356	rc2
NM_020649	CBX8	0.026981	-1.0552	-0.55658	-0.42602	-0.18511	0.16191	rc2
NM_001039457	ATP6V0B	0.199131	-1.05071	-0.1387	0.577033	0.72966	0.22677	rc2
NM_184231	NCKIPSD	0.016795	-1.04572	-0.79768	-0.79026	-0.42817	-0.07607	rc2
NM_198700	CELF1	-0.41939	-1.04294	0.175465	1.062359	0.188788	-0.30616	rc2
NM_001162384	ARHGEF2	0.467699	-1.04211	0.880933	-0.50324	-0.19064	0.74259	rc2
NM_175868	MAGEA6	0.46459	-1.0415	0.91095	-0.11887	0.47768	-0.57296	rc2
NM_001127621	GALE	-0.3332	-1.04033	-0.30176	0.866077	0.183362	1.33136	rc2
NM_002449	MSX2	-0.04618	-1.02648	-0.76245	-0.20187	-0.59388	-0.66997	rc2
NM_001171907	CXorf40A	0.8628	-1.01516	0.465367	-0.2597	0.518449	0.01002	rc2
NM_012486	PSEN2	0.057001	-1.01015	-0.06312	0.273778	-0.81708	-0.34642	rc2
NM_001206951	SLC16A3	0.384723	-1.00623	-0.72646	-0.70331	-0.09903	0.72048	rc2
NM_178126	FAM134C	-0.64367	-1.00035	-0.57476	-0.46558	-0.27259	0.33609	rc2
NR_000018	SNORD35A	-0.08876	-1.23211	-2.61116	-2.20543	0.27363	-1.39246	rc3
NM_080833	C20orf151	0.110276	-0.61701	-2.55579	-2.45953	-0.99256	-0.43953	rc3
NM_001164095	COPS7A	-1.68842	-1.47366	-2.5323	-1.49696	0.357472	0.04787	rc3
NM_001163034	RPTOR	0.164609	-1.10291	-2.50822	-1.60565	-1.08361	-0.80954	rc3
NM_006114	TOMM40	0.342798	-0.31608	-2.49674	-0.90378	-0.71651	-0.79802	rc3
NM_001083885	DFNB31	-0.71966	-0.55194	-2.47672	-2.00022	-1.86595	-1.81476	rc3
NM_001201338	SAFB	-0.86745	-2.02708	-2.39669	-0.28623	-1.79094	-2.13152	rc3
NR_002576	SNORA21	-0.84876	0.193145	-2.37117	-1.39313	-2.14399	1.34978	rc3
NM_001172412	VANGL1	-0.31925	-0.63756	-2.3384	-0.5372	-0.08879	-0.36317	rc3
NM_017697	ESRP1	-1.92404	-0.48586	-2.27155	-0.89611	0.207723	0.20262	rc3
NM_138619	GGA3	-1.07222	-0.58336	-2.21026	-0.77294	-0.1283	0.19123	rc3
NM_001242776	TMEM139	0.735626	0.282189	-2.16553	0.115206	0.646937	0.00581	rc3
NR_037591	AK2	1.027627	-0.41101	-2.16068	0.391239	-1.10992	0.03151	rc3
NR_045658	CREB3L4	-1.01007	-0.69909	-2.12474	-1.21476	-2.004	-1.59544	rc3
NM_001252294	SPDEF	-1.38495	-0.64102	-2.11712	-1.831	0.106912	-0.16031	rc3

NM_001242881	CARKD	0.312873	-0.95138	-2.08556	-0.5702	-1.36244	-0.17199	rc3
NM_001017369	MSMO1	-0.76983	1.073843	-2.0532	-0.05277	0.663779	-1.27025	rc3
NM_016169	SUFU	-0.06381	-0.69945	-2.01559	-1.18779	-0.08765	-0.00026	rc3
NM_173177	C1D	-0.52974	-1.86658	-1.98618	-1.52103	-0.18855	-1.27014	rc3
NM_052943	FAM46B	0.495139	0.209686	-1.97781	-1.26183	-0.50541	-0.15449	rc3
NM_001184781	DGCR2	-0.47266	-0.29278	-1.95117	-1.16103	-0.8468	-0.12824	rc3
NM_130795	RGS3	-0.3767	-0.80835	-1.94735	-1.17116	-1.01327	-0.60026	rc3
NR_000025	SNORD15B	0.033798	0.074333	-1.94361	-1.08711	-0.23539	-1.22518	rc3
NM_001145318	DSN1	-0.26408	0.803585	-1.93502	0.775826	-0.03936	-0.98777	rc3
NM_002611	PDK2	0.12063	-0.38939	-1.92142	-0.71902	-1.11106	-0.01818	rc3
NR_030775	SHISA4	-0.60891	-0.24441	-1.90667	-0.41498	0.743573	1.50432	rc3
NM_178040	ERC1	-0.32117	-0.38114	-1.89603	0.364295	0.014216	-1.13664	rc3
NM_014850	SRGAP3	-0.02506	-0.15297	-1.89525	-1.04057	-0.75431	-0.82361	rc3
NM_001008710	RBPMS	0.023696	-0.49373	-1.85838	-0.6846	-0.82689	-1.13876	rc3
NM_001142621	TGFBRAP1	-1.78466	-0.22709	-1.8463	-0.42835	-0.40634	-0.55848	rc3
NM_001177702	IFT27	0.085218	-0.18672	-1.84572	0.676916	-1.22607	-1.61298	rc3
NM_001135750	PSMG4	0.992415	0.303661	-1.81434	-0.31575	-0.45712	0.68443	rc3
NM_152925	CPNE1	-1.04165	-0.04593	-1.80901	-0.31175	0.581871	1.41935	rc3
NM_001130848	PITPNM1	-0.38186	-0.10216	-1.79873	-0.53831	-1.08365	1.14101	rc3
NM_002221	ITPKB	-0.17469	-0.86837	-1.77832	-1.2725	0.508285	0.53877	rc3
NM_173587	RCOR2	-0.16182	-1.01187	-1.76271	-1.49485	0.181184	0.06285	rc3
NM_001024808	BCL7A	-0.19489	-0.99935	-1.74373	-0.28709	-0.46305	-0.91324	rc3
NM_004915	ABCG1	-1.03158	-1.15173	-1.73454	-1.44301	-1.25119	-0.63911	rc3
NM_001040165	C16orf13	-0.78707	-0.34887	-1.7124	-0.3184	-1.48243	-1.34207	rc3
NM_207005	USF1	-0.77167	-1.01943	-1.70874	-0.35749	-0.90293	-0.10437	rc3
NM_016569	TBX3	0.094615	-0.39953	-1.70263	0.437345	-0.21389	-0.67557	rc3
NM_001035005	C18orf32	-0.16251	-0.32513	-1.67781	0.024984	-0.35361	-0.75911	rc3
NM_001199121_6	RPP21	0.092838	0.090012	-1.67545	-0.38894	-0.46032	0.07855	rc3
NM_001199282	LRBA	-1.56733	-0.96936	-1.66439	-0.37979	-0.36407	-1.53511	rc3
NM_080738	EDARADD	0.638667	-0.23172	-1.65066	-0.04465	-0.08861	-1.10444	rc3
NM_133639	RHOV	0.831682	0.456631	-1.6484	-0.98627	-0.24233	-0.11821	rc3
NM_019013	FAM64A	0.252065	0.110208	-1.63672	-0.22597	-0.28376	0.84008	rc3
NM_001168344	RREB1	-0.78081	0.288029	-1.63359	-1.05181	0.089654	0.31593	rc3
NM_001005914	SEMA3B	-0.09764	0.218091	-1.62625	-0.27368	-0.28648	-0.29279	rc3
NM_015436	RCHY1	-0.29072	0.135033	-1.62257	-1.342	-0.44508	-1.37210	rc3
NM_006105	RAPGEF3	-0.35398	-1.48686	-1.61678	-0.94156	-1.04854	-1.23628	rc3
NM_016291	IP6K2	-1.43059	-0.61205	-1.6036	-0.99616	-0.5929	-0.51810	rc3
NM_031300	MXD3	-0.02029	-0.70405	-1.59927	-0.82244	0.350614	0.63765	rc3
NM_001243088	FOXM1	-0.70101	-0.96596	-1.59532	-1.15546	0.549579	-0.35778	rc3
NM_173060	CAST	-0.40351	-0.51881	-1.57137	-0.08011	-0.71891	-1.53809	rc3
NM_001007027	ALG8	-0.67029	-0.31489	-1.5701	0.317649	-1.15648	-0.60159	rc3
NM_006670	TPBG	0.089374	-0.28779	-1.56242	-0.45779	0.813986	0.18156	rc3
NM_004381_3	ATF6B	-0.82757	-1.49996	-1.55004	-1.0021	-0.74885	0.64234	rc3
NM_001009933	DNASE1L1	-1.10437	-0.22411	-1.54237	-0.06352	0.355615	0.74471	rc3
NM_021922	FANCE	-0.18146	-0.51925	-1.54174	-1.26214	-1.00548	-1.28277	rc3
NR_023358	SCARNA9L	-0.83094	-1.52439	-1.53392	-1.41867	-0.67447	0.05532	rc3
NM_078471	MYO18A	-0.15599	-0.28249	-1.51562	-0.75945	-1.03441	-0.23569	rc3

NM_198217	ING1	-0.54978	-1.30788	-1.5117	-0.61876	-0.59286	-1.10493	rc3
NM_020536	CSRP2BP	-0.2647	-0.48778	-1.50884	-1.02986	-0.56365	-1.41885	rc3
NR_002590	SNORA41	1.039623	-0.86925	-1.5055	0.871033	-1.27832	-0.23473	rc3
NM_001242900	PPP6R2	0.494965	-0.33123	-1.50478	0.004577	0.543641	-0.31323	rc3
NM_001011667	CHCHD7	-0.60994	-0.78408	-1.50088	-0.10758	-0.14256	-1.15472	rc3
NM_001048174	MUTYH	0.049786	-0.6158	-1.48854	-0.77849	-0.9613	-1.14804	rc3
NM_006024	TAX1BP1	-1.47318	-0.84894	-1.482	-0.55312	0.778636	0.37428	rc3
NM_001003827	TRIM34	0.093103	-0.11029	-1.47123	-0.01234	0.577238	0.40294	rc3
NM_000282	PCCA	0.598612	0.258039	-1.46611	0.356435	0.274631	-0.00104	rc3
NM_001174087	NCOA3	-0.46318	0.493979	-1.44984	-0.95108	1.051618	1.18299	rc3
NR_029427	WDR13	0.368677	0.026786	-1.43892	-0.18605	-0.39689	-0.45945	rc3
NM_001130440	SRP9	-0.66934	-0.80553	-1.4341	-0.53958	-0.71304	-1.15396	rc3
NR_033839	FLJ39051	-0.1471	-0.59612	-1.38072	-1.12431	-0.63573	-0.80359	rc3
NM_007171	POMT1	-0.48415	-0.39843	-1.37	-0.94381	-0.68811	-0.89556	rc3
NM_172349	NSD1	-0.3008	-0.38912	-1.35739	-0.45244	-0.49188	-0.85242	rc3
NM_015401	HDAC7	0.232788	-0.75499	-1.35194	-1.05887	1.353042	1.45160	rc3
NM_032636	PSRC1	-1.08323	-0.01503	-1.35044	-0.59689	0.340208	-0.13152	rc3
NM_001042476	CARHSP1	-0.04737	-0.0841	-1.34975	0.141208	0.67746	0.14509	rc3
NM_003475	RASSF7	-0.15651	-0.06387	-1.34855	0.045215	-0.9024	-0.30508	rc3
NM_001445	FABP6	-0.60528	-1.23726	-1.33643	-0.9564	-0.55219	-0.51011	rc3
NR_028582	G6PC3	-0.25403	-0.22115	-1.3358	-0.93069	-0.78284	0.35828	rc3
NM_001256568	PPFIBP2	-0.05117	-0.70547	-1.32595	-0.71846	-1.13232	-1.30730	rc3
NM_020719	PRR12	-0.06195	-0.97322	-1.31659	-0.98755	-0.19748	-0.05744	rc3
NM_024507	KREMEN2	-0.60371	-0.02368	-1.31653	-0.94329	-0.92199	-0.58561	rc3
NM_145647	WDR67	-0.36121	-0.56768	-1.31233	-0.32899	-0.50536	-0.51133	rc3
NM_001012727	AGPAT2	0.467498	-0.08682	-1.31167	0.298728	1.214594	1.57228	rc3
NR_028514	LOC10030695	-0.06429	-0.79483	-1.3115	-0.2195	-0.16713	-0.25491	rc3
NM_005600	NIT1	-0.95771	-0.36482	-1.31114	-0.47633	0.182201	0.28706	rc3
NM_001144062	COPB1	0.641899	1.173743	-1.30956	0.76673	0.031717	-0.28243	rc3
NM_001195737	FAM213B	-0.84383	-0.97046	-1.30926	-0.5593	-0.78541	-1.24983	rc3
NM_001144962_4	NFKBIL1	0.06283	0.506008	-1.30675	-1.08367	0.631487	-0.35065	rc3
NM_006053	TCIRG1	-0.45608	0.326681	-1.30654	-0.38703	-0.15186	0.10636	rc3
NM_004628	XPC	0.026787	-0.16329	-1.30456	-0.63419	-1.28831	-1.21404	rc3
NM_001079539	XBP1	0.010901	-0.52294	-1.30178	-1.06081	-1.19183	-1.22162	rc3
NM_032305	POLR3GL	-0.39965	-1.04698	-1.29845	-0.79928	-0.72885	-1.23249	rc3
NM_004067	CHN2	-0.03391	-0.00181	-1.29667	-1.20663	0.279899	0.09476	rc3
NR_045033	APH1A	-0.28368	-0.34563	-1.29367	-0.60757	-0.70702	-1.04756	rc3
NM_001166103	SPINT2	-0.04374	-0.89132	-1.29154	-0.84849	-0.15953	-0.36930	rc3
NM_004443	EPHB3	-0.25634	-0.98461	-1.28572	-1.09949	-0.39869	-0.10555	rc3
NM_017817	RAB20	-0.08265	-1.02767	-1.261	-0.85672	-0.55645	-0.16959	rc3
NR_038257	TRPM2	0.457901	-0.81017	-1.25891	0.474996	-1.24639	-1.20759	rc3
NM_024922	CES3	-1.15039	-0.25277	-1.25618	-0.74386	-0.18772	-0.23414	rc3
NM_144608	HEXIM2	-0.18227	-1.22106	-1.2546	-1.00934	-0.60641	-0.42974	rc3
NM_018204	CKAP2	-0.40088	-0.86766	-1.24986	0.092107	0.194663	-0.52987	rc3
NM_003392	WNT5A	-0.23727	-0.25838	-1.24663	-0.27542	-0.67872	-0.47072	rc3
NM_004567	PFKFB4	0.041838	-0.53086	-1.24617	-1.00505	-0.17965	0.08032	rc3
NM_198433	AURKA	-0.62034	-0.69438	-1.24571	-0.57827	0.423877	1.16431	rc3

NM_001039661	TIRAP	-0.3751	-0.44331	-1.24385	-0.76566	-0.43467	-0.80291	rc3
NR_036495	LOC646938	-0.37629	-0.17881	-1.24231	-0.83386	0.135354	-0.10083	rc3
NR_027383	AKAP17A	-0.89861	0.568853	-1.23773	-0.06481	-1.13279	0.82199	rc3
NM_014676	PUM1	-0.51756	0.19555	-1.23461	0.187106	-0.14042	-0.28249	rc3
NM_152658	THAP8	-0.08489	-0.32027	-1.22814	-1.22065	-0.79576	-0.42869	rc3
NM_052854	CREB3L1	-0.10363	-0.04767	-1.22533	-0.7201	0.078624	0.26863	rc3
NM_001171908	CXorf40A	-0.50319	-0.36191	-1.22384	-0.91419	-0.99177	-0.00079	rc3
NM_001128228	TPRN	-0.11667	-0.39968	-1.22331	-1.20718	-0.26346	0.35924	rc3
NM_001207051	CLASP1	-0.53933	-0.25622	-1.22322	-0.20989	0.692634	-0.66118	rc3
NM_001002257	LCLAT1	-0.36165	-0.05356	-1.22105	-0.04702	-0.77826	-0.77809	rc3
NM_148179	C9orf23	-0.0572	-1.02185	-1.21914	-0.07801	-0.40368	0.47700	rc3
NM_020455	GPR126	-0.67535	0.272747	-1.21842	0.650509	0.326203	-0.13831	rc3
NM_012398	PIP5K1C	-0.24079	-0.40842	-1.21498	-0.8824	-0.39262	-0.16872	rc3
NM_025069	ZNF703	0.394064	-0.47986	-1.20761	-0.74785	0.273186	0.49229	rc3
NM_001199379	GBF1	-0.75213	-1.19556	-1.2035	-1.0231	-0.0386	0.19552	rc3
NM_134424	RAD52	-0.24606	-0.69881	-1.19787	-0.66058	0.109307	0.18683	rc3
NM_178568	RTN4RL1	-0.11757	-0.78419	-1.197	-1.07137	-1.01185	-1.01680	rc3
NM_004500	HNRNPC	-0.72024	0.029102	-1.19617	0.160345	-0.62026	-0.28007	rc3
NM_001173539	BANP	0.021125	-0.76813	-1.19562	-0.53757	-0.89628	0.28530	rc3
NM_001242312	FAM124A	-0.4377	-0.92493	-1.19297	-0.61958	-0.87736	-0.84860	rc3
NM_020319	ANKMY2	-0.14698	0.023249	-1.18578	-0.86855	-0.09287	-0.46123	rc3
NM_018337	ZNF444	0.106737	-0.86173	-1.18489	-0.89327	-0.46315	-0.06157	rc3
NM_001166261	MTIF3	-0.11797	-0.64579	-1.18454	-0.56657	-0.03468	-0.68724	rc3
NM_133374	ZNF618	-0.06934	-0.21237	-1.1725	-0.71404	-0.79083	-0.67091	rc3
NM_001256585	MGLL	-0.65451	0.413906	-1.16828	0.06378	0.134828	0.62342	rc3
NM_015373	CBY1	-0.1296	-0.5153	-1.16312	-1.0894	-0.62433	-0.47905	rc3
NM_021222	PRUNE	-0.03386	-0.67444	-1.15988	-0.6737	-0.21215	-0.37894	rc3
NM_014791	MELK	-0.17621	-0.36512	-1.15988	0.021617	0.133548	-0.60024	rc3
NM_001014765	FBXO44	0.031818	-0.45068	-1.15556	-0.20834	0.220315	0.41878	rc3
NR_002575	SNORA27	1.103968	1.781314	-1.15541	-0.14518	2.216273	1.19579	rc3
NM_000149	FUT3	-0.31401	0.158857	-1.15074	-0.03219	0.821287	1.35495	rc3
NM_024784	ZBTB3	-0.82902	-0.98176	-1.15013	-1.11237	-0.44381	-0.24773	rc3
NM_021732	AVPI1	0.411673	0.204758	-1.14966	-1.06874	0.064955	0.06555	rc3
NM_173547	TRIM65	-0.23867	-1.07111	-1.14941	-0.82736	-0.43535	-0.24501	rc3
NM_006498	LGALS2	-0.36357	0.078394	-1.14675	-0.64026	-0.21463	0.38847	rc3
NM_001256605	C1orf85	-0.44309	-0.80307	-1.1437	-1.01357	-0.53605	0.43834	rc3
NM_183422	TSC22D1	-0.24337	-0.78541	-1.14098	-0.7251	-0.57167	-0.66786	rc3
NM_005853	IRX5	0.250036	-0.02424	-1.13716	0.113546	-0.2554	-0.31092	rc3
NM_020423	SCYL3	-0.10364	-0.47718	-1.1369	-0.66911	-0.15272	-0.37738	rc3
NM_018073	TRIM68	-0.0246	-0.77382	-1.13348	-1.03764	-1.0899	-1.00521	rc3
NM_018421	TBC1D2	0.067547	-0.3066	-1.13038	-0.80038	-0.34051	-0.21872	rc3
NM_152504	C20orf196	-0.11773	-0.44841	-1.12495	-0.44564	0.046566	-0.12263	rc3
NM_001031681	CTNS	-0.1463	-0.65841	-1.12495	-0.26557	-0.66542	-0.67894	rc3
NM_031968	NARF	-0.49958	-0.90123	-1.12421	-1.02153	-0.78916	-0.79324	rc3
NM_001256799	GAPDH	-0.35858	-0.46148	-1.11999	-0.60041	-0.44583	0.60600	rc3
NM_015291	DNAJC16	0.128891	-0.24503	-1.11859	-0.58395	-0.30463	-0.42632	rc3
NR_028049	MTERFD2	-0.18723	-0.66315	-1.11766	-0.501	-0.8682	-1.06629	rc3

NM_080659	C11orf52	0.054394	-0.63859	-1.11766	-0.50062	-0.12002	-0.32823	rc3
NM_016270	KLF2	3.439357	1.606056	-1.11743	-0.67142	0.387204	0.49273	rc3
NM_032127	FAM160A2	0.205934	0.355407	-1.11505	-1.10249	0.391246	0.32587	rc3
NM_001172688	GGA1	-0.38137	0.239753	-1.11293	0.000982	0.291376	0.32979	rc3
NR_031695	MIR1282	-0.8226	-1.10384	-1.1096	-1.10326	-0.34219	-0.36254	rc3
NR_026650	BPHL	-0.94947	-0.44851	-1.10647	-0.28343	-0.20411	-0.59364	rc3
NM_152493	ZNF362	-0.00214	-0.30949	-1.10002	-0.97209	-0.80346	-1.00959	rc3
NM_001258	CDK3	-0.35559	0.003091	-1.09889	-1.04516	-0.09474	-0.87410	rc3
NM_001010883	FAM102B	-0.40815	-0.10217	-1.09783	-1.02245	-0.66592	-0.67895	rc3
NR_038357	LOC10050733	-0.4224	0.35788	-1.09647	-0.26872	-0.88296	-0.82835	rc3
NM_000319	PEX5	-0.84531	-0.46702	-1.09559	-0.30485	-0.33673	0.18878	rc3
NM_001204466	RBM10	0.755292	0.143573	-1.09411	-0.59028	-0.30069	0.19421	rc3
NR_003695	SNORD12B	0.443887	-0.22394	-1.09282	-0.2054	-0.60383	-0.09592	rc3
NM_001244682	POU2F3	-0.91392	0.2194	-1.08362	-0.73928	0.467252	0.18083	rc3
NM_001142623	GHDC	-0.36878	-0.91214	-1.07754	-0.85529	0.279337	0.67995	rc3
NM_207293	MBNL1	-1.00565	-0.22736	-1.07751	-0.02766	-0.10982	0.15142	rc3
NM_020338	ZMIZ1	-0.17346	-0.34879	-1.07504	-0.94969	-0.97029	-0.89569	rc3
NM_202467	GIPC1	0.265231	-0.01094	-1.07496	0.812881	0.229622	0.38127	rc3
NM_019619	PARD3	-0.76119	-0.88915	-1.07276	0.210503	0.64686	-0.58286	rc3
NM_001203258	MTA1	1.748304	1.41135	-1.07212	1.432861	1.826881	0.57565	rc3
NM_182526	TMEM229B	-0.14197	-0.44781	-1.06537	-0.92357	-0.48147	-0.81048	rc3
NM_033446	FAM125B	-0.14165	-0.44675	-1.06422	-0.48206	-0.26994	-0.05002	rc3
NM_001042410	ANKZF1	-0.21373	-0.41132	-1.05963	-0.97538	0.484391	0.79115	rc3
NM_152667	NANP	-0.21738	-0.18883	-1.05679	-0.32666	-0.75869	-0.62714	rc3
NM_001173512	MRRF	0.405607	0.135407	-1.05657	0.004398	-0.60099	-0.36163	rc3
NM_001135944	MADD	0.912392	-0.95992	-1.04564	-0.82165	0.775862	1.05510	rc3
NM_001031694	SCMH1	-0.29872	-0.48872	-1.04557	-0.35293	-0.07006	0.09109	rc3
NM_024702	ZNF750	0.360248	-0.20127	-1.04187	-0.79533	0.51456	0.43224	rc3
NM_001168320	TSPAN9	0.209299	0.010353	-1.04052	-0.80396	-0.27725	-0.29421	rc3
NM_015111	N4BP3	-0.05868	-0.71063	-1.03929	-0.92616	0.576875	0.74270	rc3
NM_007170	TESK2	0.215563	-0.11654	-1.03446	-0.83649	0.31908	0.45198	rc3
NM_001024674	LIN52	0.248263	-0.07774	-1.03193	-0.52373	-0.41687	-0.73525	rc3
NM_001017368	RFFL	-0.68503	-0.38604	-1.02867	0.041162	-0.18324	0.36751	rc3
NM_001081559	CPSF4	-0.08404	-0.28337	-1.02316	-0.4378	-0.62588	-0.27318	rc3
NM_016567	BCCIP	0.018646	-0.25044	-1.02291	-0.40269	-0.70898	-0.96842	rc3
NM_014920	ICK	-0.42043	-0.18727	-1.0197	-0.30282	-0.47544	-0.71251	rc3
NM_006054	RTN3	0.129283	-0.63736	-1.01936	-0.39301	-0.56464	-0.68234	rc3
NM_001024936	WASF1	-0.51506	-0.05701	-1.01867	-0.63338	-0.89874	-0.24284	rc3
NM_001130020	ATP6V0A1	1.970754	-0.50251	-1.01789	0.742192	0.71018	1.19207	rc3
NM_033034	TRIM5	0.226275	-0.10375	-1.01386	0.022051	0.285877	0.13367	rc3
NM_002646	PIK3C2B	-0.1773	-0.59638	-1.00844	-0.70908	-0.41155	-0.25692	rc3
NM_018027	FRMD4A	0.038961	-0.48321	-1.00719	-0.52091	-0.40982	-0.38797	rc3
NM_032303	HSDL2	-0.09931	-0.57759	-1.00257	-0.24135	-0.38979	-0.08868	rc3
NM_152926	CPNE1	0.414272	-1.02801	-0.74797	-3.58974	0.302519	1.06989	rc4
NM_001206797	PKM2	-0.89347	-0.18095	-1.56047	-3.50209	-3.07839	-2.16177	rc4
NR_027437	IP6K2	-1.16513	-1.99751	-1.83368	-3.15563	-1.11646	-1.02816	rc4
NM_022648	TNS1	-0.14987	-0.14775	-1.75198	-3.10035	-2.55208	-3.04761	rc4

NM_001199823	NEDD8-MDP1	-0.76444	-1.57568	-0.11635	-3.03353	-0.77393	-1.00180	rc4
NR_031576	MIR762	-0.49644	-0.05746	-0.13366	-2.98356	-0.46104	0.93906	rc4
NM_002592	PCNA	-0.62794	-0.46452	-0.00119	-2.92201	-2.20397	-1.16116	rc4
NM_021220	OVOL2	-0.1695	-0.52513	-1.91718	-2.88611	-0.47546	-0.41023	rc4
NM_001242773	TMEM139	-1.19795	-0.19744	-1.86019	-2.88521	-0.06649	-1.46760	rc4
NM_006058	TNIP1	0.829062	0.320204	0.669941	-2.87573	0.682419	0.81444	rc4
NM_173157	NR4A1	4.226002	4.151098	-2.01998	-2.8476	-0.48419	-0.41286	rc4
NR_037482	MIR3918	-1.16797	-1.57843	0.293023	-2.74247	-1.05095	-1.14658	rc4
NM_005080	XBP1	0.160306	-0.13629	-0.75209	-2.74236	-0.57229	-0.52841	rc4
NM_178581	HM13	-0.87057	-1.01581	-1.8139	-2.70873	-1.35016	-1.37260	rc4
NM_206840	NVL	-0.91918	0.753597	0.538584	-2.55643	-0.47107	-0.16531	rc4
NM_178427	ARNT	-0.21077	-0.54758	-0.70717	-2.55177	-0.19204	-1.78779	rc4
NR_003020	SNORA78	-1.15725	0.832906	0.250703	-2.54085	-0.66349	-1.44082	rc4
NM_006314	CNKSR1	0.104281	-0.84481	-0.37689	-2.47103	-0.22041	1.82165	rc4
NM_080702	BAG6	-0.24106	-0.40595	0.440836	-2.45986	-0.51867	0.04404	rc4
NM_001142353	PPP3CB	-0.20343	-0.18682	-1.90627	-2.43663	0.958167	-0.59169	rc4
NM_001145025	EDEM2	-0.91725	-1.13063	-1.77513	-2.41461	-1.11874	-0.94470	rc4
NM_148918	SHMT1	-0.13127	-0.30886	-0.55344	-2.39392	-1.63726	-1.88713	rc4
NR_036577	APTX	-1.11224	-0.10312	-1.91498	-2.3909	-1.13452	-0.49235	rc4
NR_037434	MIR3661	0.204245	0.621404	-0.8838	-2.35638	0.242879	0.56252	rc4
NM_032026	TATDN1	-0.5287	0.305156	0.220953	-2.31333	-0.26844	-0.05987	rc4
NM_199181	KIAA0889	-0.36216	-0.49315	-1.70044	-2.30226	-1.14049	-1.11339	rc4
NM_024636	STEAP4	0.153056	0.260941	0.318559	-2.30177	1.492552	1.53457	rc4
NM_001017957	OS9	-0.57869	-0.20129	0.142489	-2.28101	-0.41064	0.03708	rc4
NM_033086	FGD3	-0.01239	-0.64016	-1.93574	-2.26348	-1.76287	-1.34039	rc4
NM_033624	FBXO21	-0.40169	-0.08775	-0.92656	-2.21727	-0.35219	-0.68941	rc4
NM_145249	IFI27L1	-0.67722	-1.02137	0.297312	-2.18764	-0.66744	-0.90664	rc4
NM_004393	DAG1	0.121891	-0.61663	-1.37372	-2.17512	-0.12395	-0.44934	rc4
NM_002012	FHIT	-0.58336	-1.58379	-1.22816	-2.16773	-0.82274	-0.70906	rc4
NM_000116	TAZ	0.299127	0.358528	-0.06304	-2.14337	-0.99433	-2.10683	rc4
NR_024080	ACP1	0.136624	-0.50158	0.56095	-2.13392	-1.21702	-1.20229	rc4
NM_001142403	CD164	0.353404	0.788823	-0.58817	-2.12126	0.876697	-0.14836	rc4
NM_002616	PER1	0.874515	0.891707	-1.72586	-2.10166	-0.21671	-0.41274	rc4
NM_001144759	PHLDB1	-0.06144	-0.15247	-1.03695	-2.08455	0.111913	0.57456	rc4
NM_001015053	HDAC5	-0.05595	-1.16175	-0.26716	-2.06029	0.30726	0.29374	rc4
NM_032872	SYTL1	0.069536	-0.01771	-1.13021	-2.014	-0.95908	-0.89610	rc4
NM_001042424	WHSC1	-0.17161	0.315226	0.152433	-1.99669	-1.18799	-0.36342	rc4
NM_001039476	NPRL3	-0.30159	-0.64504	-0.47358	-1.99646	-0.21757	-0.05546	rc4
NM_001082486	ACD	0.010874	0.008344	-0.17548	-1.98967	0.003486	-0.18988	rc4
NR_033464	NT5C3L	-0.59682	-0.92407	-0.52194	-1.98667	-0.4085	0.32038	rc4
NM_001146019	TJAP1	-1.18932	-0.75174	-1.27371	-1.98034	-0.30679	1.07330	rc4
NM_001963	EGF	-0.03486	0.11501	-0.64766	-1.96449	0.183177	0.31915	rc4
NM_181505	PPP1R1B	-0.56331	-0.60145	-0.78238	-1.96216	-0.32842	-0.56989	rc4
NM_012161	FBXL5	-0.24161	0.264986	-0.02336	-1.95817	0.651273	0.12370	rc4
NM_018144	SEC61A2	-1.34215	-1.12876	0.074833	-1.93944	0.230728	0.15206	rc4
NM_024519	FAM65A	-0.1882	-0.15037	-1.31738	-1.93132	-0.17036	0.25374	rc4
NM_002673	PLXNB1	0.352644	0.635337	-0.18333	-1.91966	0.041173	0.84927	rc4

NM_001253792	ZNF444	-0.33388	0.482924	-0.57604	-1.91948	1.805337	2.12158	rc4
NM_001204063	CHURC1	0.685391	-0.15218	0.50216	-1.9111	-1.04974	-0.40760	rc4
NM_001243127	PACS2	-0.61518	-0.78585	-0.92142	-1.90654	-1.32318	-0.41751	rc4
NM_001184896	PHF8	-0.28163	-0.8536	-0.50222	-1.8996	-1.26846	-1.25857	rc4
NM_003597	KLF11	-1.22237	-0.27722	0.081772	-1.85399	-0.29025	0.30492	rc4
NM_016096	ZNF706	-0.42138	0.418977	0.619128	-1.84752	0.325408	-0.13267	rc4
NM_003535	HIST1H3J	-0.82633	-1.52782	-1.19712	-1.84195	-1.3325	-1.42928	rc4
NM_001251885	VIPR1	-1.6797	-0.66499	-0.10611	-1.83527	-0.84326	-1.03591	rc4
NM_001178079	STAT6	0.357964	-1.6993	0.254138	-1.83506	-0.00986	0.21504	rc4
NM_001606	ABCA2	-0.91271	-0.15804	-1.08338	-1.82159	-0.79703	-0.20499	rc4
NM_017920	URGCP	2.131174	1.497963	0.508003	-1.8211	-0.05979	0.17909	rc4
NM_003216	TEF	-0.12979	-0.50877	-1.73602	-1.81548	0.001987	0.00865	rc4
NM_203417	RCAN1	0.993146	1.242633	-0.91728	-1.7948	0.639435	-1.72497	rc4
NM_015127	CLCC1	-0.02027	-0.96239	-0.78725	-1.79316	-1.26617	-1.56043	rc4
NR_029702	MIR149	-0.62567	-0.05534	0.480906	-1.79225	0.090711	-0.91383	rc4
NM_198057	TSC22D3	-0.08452	-0.46765	-1.12512	-1.78229	-0.81508	-1.02715	rc4
NR_036517	C17orf62	-0.12337	-0.44126	-0.80422	-1.78101	-0.31167	-0.10552	rc4
NM_001243787	SMUG1	-1.29104	-1.30345	0.056631	-1.7764	-0.04071	0.32458	rc4
NM_001146258	PIP4K2C	-1.10673	0.390183	-0.85113	-1.77128	0.18351	-0.62605	rc4
NM_001143667	ZBED5	-1.16048	0.656686	-0.11277	-1.76464	-0.03419	-0.60655	rc4
NM_001025235	TSPAN4	0.057648	-1.46012	-0.91831	-1.76219	1.147973	-1.01724	rc4
NM_001114185	MVK	0.109891	-0.09244	-0.66029	-1.75713	-0.74167	-0.48407	rc4
NM_001077664	URGCP	0.121196	-0.00568	-0.97496	-1.75704	-0.28368	-0.49605	rc4
NM_147202	C9orf25	-0.35693	0.659881	-0.28226	-1.75566	1.193676	1.19925	rc4
NM_181311	TAZ	0.067526	-0.1228	-0.17969	-1.75521	-0.5348	-0.90103	rc4
NM_182811	PLCG1	1.005002	0.632662	-1.10312	-1.73687	0.473756	0.29245	rc4
NM_201599	ZMYM3	-0.16959	-0.41311	-1.49092	-1.73022	-0.31115	-0.39462	rc4
NR_038956	LOC10050671	0.069488	-0.64672	-0.74429	-1.72953	-0.31488	-0.31160	rc4
NM_001242547	ANAPC16	-0.18273	0.598287	-1.31751	-1.71612	-1.08972	-1.24678	rc4
NM_001130517	MPST	0.118391	-1.15857	-0.64968	-1.70325	0.881668	1.26250	rc4
NM_001007533	PPP1R27	-0.05113	-0.73343	-0.98946	-1.70235	-1.30046	-1.27302	rc4
NM_001143990	WRAP53	-0.03156	-0.1697	-1.67245	-1.69968	-1.37877	-1.66352	rc4
NM_001166349	SLC26A11	-0.166	-1.0696	-0.23711	-1.6852	-0.10287	-0.42544	rc4
NM_001143842	TMEM106C	0.383273	-0.82368	-0.93751	-1.64122	-0.67632	-1.23879	rc4
NR_024511	LOC728855	-0.42622	-0.74619	-0.45942	-1.6323	0.350503	0.43922	rc4
NM_152362	TNFAIP8L1	-0.83212	-0.78977	-1.26235	-1.6294	0.187074	0.29349	rc4
NM_032772	ZNF503	-0.48396	-0.93211	-0.80388	-1.62551	0.324245	0.34624	rc4
NM_001191006_1	SRSF10	-1.34819	-0.55911	-1.54257	-1.62418	-0.64032	-1.29140	rc4
NM_001788	42985	0.144223	0.463932	-1.60907	-1.62393	-1.37101	0.38213	rc4
NM_080627	KIAA0889	0.035534	-0.29878	-1.34806	-1.61839	-0.38393	-0.51173	rc4
NM_001143994	RASSF7	0.113999	-1.55552	0.20135	-1.60596	-1.09677	-0.41021	rc4
NM_001037806	NCKAP5L	0.290776	0.391386	-1.02929	-1.59328	0.147001	0.63692	rc4
NR_033183	MIB2	0.397826	0.152478	-0.88379	-1.58056	-0.30261	0.13009	rc4
NM_016733	LIMK2	0.064757	0.123953	-0.22116	-1.56194	-0.92056	-0.61280	rc4
NR_027283	LOC440461	0.030411	-0.41643	-1.38634	-1.5536	-0.98869	-1.53536	rc4
NM_001085454	GIT1	-1.11149	-0.77757	-0.09535	-1.55313	-1.23735	0.16324	rc4
NM_001163259	FAM63A	0.546608	0.525824	-0.30347	-1.55067	0.523449	-0.17649	rc4

NR_022007	PMS2P4	-0.14662	-1.0411	-0.3891	-1.53658	-1.20557	-1.31774	rc4
NM_001142651	NEURL1B	-0.26768	-0.58468	-1.28971	-1.53232	0.750333	0.60993	rc4
NM_001184888	NIPA2	0.200026	-0.4725	-0.03957	-1.53067	-1.42205	-0.35072	rc4
NM_024859	MAGIX	-0.09605	-0.7053	-0.45407	-1.52876	-0.12565	-0.49565	rc4
NM_020155	GPR137	-0.2465	0.593128	0.018104	-1.51814	-0.82898	0.43700	rc4
NM_022353	OSGEPL1	-0.06662	-0.35777	-1.09919	-1.51807	-0.99805	-1.01530	rc4
NM_001242412	AHRR	0.000944	-0.07813	-0.93421	-1.51332	-0.97905	-0.32185	rc4
NM_001128631	DCAKD	0.083731	-0.7832	-1.21283	-1.5096	-0.85935	-0.94520	rc4
NM_022166	XYLT1	-0.09609	0.021268	-0.76111	-1.50871	-0.37647	-0.30739	rc4
NM_134427	RGS3	1.057033	-0.31928	-1.45626	-1.50702	0.69006	0.98997	rc4
NM_001193655	C17orf62	-0.49189	-0.40179	-0.81758	-1.5014	0.248256	0.49325	rc4
NM_012121	CDC42EP4	-0.15019	-0.75756	-1.46107	-1.49409	-0.53269	-0.55215	rc4
NM_006702	PNPLA6	-0.3874	0.028801	0.019506	-1.49261	0.347748	0.79781	rc4
NM_001198993	NADK	-0.32746	-0.4075	-1.16611	-1.48867	-1.23045	-1.27046	rc4
NM_021205	RHOU	-0.01016	0.158696	0.014294	-1.48352	0.334029	0.16060	rc4
NM_001136012	FXYD3	-0.18297	-0.59917	-0.19307	-1.4824	-0.25176	0.18634	rc4
NM_001080482	C9orf172	0.165304	-0.27123	-0.92469	-1.48013	-0.30927	-0.20746	rc4
NR_002569	SCARNA9	-0.8987	-1.30976	-1.0034	-1.47854	-0.75568	-0.41612	rc4
NM_001142861	SMAD6	-0.29539	-0.56778	-0.94737	-1.47821	-1.28758	-0.97077	rc4
NM_153000	APCDD1	0.131907	-0.20789	-0.83874	-1.47652	-1.06436	-1.38442	rc4
NM_001135638	PIP5K1A	0.909724	1.875883	0.219937	-1.4763	-0.5388	0.91858	rc4
NM_152332	TC2N	-0.43246	-0.64377	-0.96522	-1.47472	-0.84086	-0.05986	rc4
NM_003532	HIST1H3E	-0.80987	0.218707	-0.60727	-1.46835	-0.62737	0.27199	rc4
NM_001034023	DMAP1	-0.34782	-1.16083	-0.76547	-1.464	-0.13876	0.23256	rc4
NM_017836	SLC41A3	-0.20066	-0.65592	-0.29073	-1.4628	1.248423	0.85415	rc4
NM_003628	PKP4	-0.473	-0.60643	-1.25063	-1.45306	-0.49847	-0.59381	rc4
NM_153347	TMEM86A	0.169187	-0.08152	-0.86227	-1.45014	-1.30072	-1.41870	rc4
NM_007312	HYAL1	-0.25536	-0.32862	-0.43834	-1.43127	-0.22036	0.21271	rc4
NM_001184796	ESYT1	-0.23847	0.037551	0.542793	-1.43033	0.282344	0.78571	rc4
NM_014434	NDOR1	0.026724	-0.3692	-0.30523	-1.42962	-0.14787	0.06848	rc4
NM_005419	STAT2	-0.47057	-0.28246	-1.31552	-1.42552	0.116701	0.19757	rc4
NM_001017958	OS9	-0.86552	-0.05639	0.147296	-1.4244	-0.15702	-0.19428	rc4
NM_001142370	PTPN18	-0.36694	-0.56396	-0.81777	-1.42238	-0.6903	-0.62810	rc4
NM_001256355	UBE2L3	1.838352	-0.73535	0.169727	-1.41772	0.836586	0.00729	rc4
NM_004938	DAPK1	-0.01333	0.072339	-1.11789	-1.41585	0.51897	0.37433	rc4
NM_001024227	ARF1	-0.79363	-0.75018	-0.70945	-1.41572	-1.36493	0.03920	rc4
NM_001032387	SUOX	-0.61062	-0.60287	-1.35828	-1.40573	-0.5672	-0.90751	rc4
NR_037577	TMEM222	0.009425	-0.47664	-0.43105	-1.40207	-0.88982	-0.85524	rc4
NM_001172219	SCMH1	0.877474	0.558101	-0.20574	-1.40089	0.851041	1.17852	rc4
NM_130853	PTPRS	-0.65392	-0.52569	-1.06183	-1.39407	-1.04163	-1.24536	rc4
NM_001242393	ACSS2	-0.28433	-0.32927	-0.71355	-1.39356	-0.33048	-0.33540	rc4
NM_014323	PATZ1	0.577534	-0.64359	-0.83575	-1.39285	0.03002	-1.25908	rc4
NM_001040146	CHTF8	-1.00674	-0.71735	-0.54129	-1.38491	-0.70775	0.90649	rc4
NM_001039469	MARK2	0.100884	-0.18266	-1.05742	-1.37442	0.376047	0.08078	rc4
NM_001253823	BLVRA	-0.11552	-0.20648	-0.3799	-1.35785	-1.06206	0.33874	rc4
NM_184043	ALDOA	0.919329	0.895264	-1.16142	-1.35621	-0.50964	-0.71187	rc4
NM_003545	HIST1H4E	-0.35536	-0.09188	-0.42029	-1.35011	0.070711	-0.24183	rc4

NM_152237	GAS2L1	-0.33496	-0.34282	-0.72974	-1.34416	-0.69974	0.38596	rc4
NM_012286	MORF4L2	-0.13564	0.82696	0.543311	-1.34365	1.404638	1.60493	rc4
NM_001142465	ECSIT	-0.52159	-0.94471	-1.07992	-1.34175	-0.49797	0.14307	rc4
NR_002604	SNORD10	0.388701	0.547208	-0.54848	-1.34035	0.474641	-0.01540	rc4
NM_001135917	DOLPP1	-0.80994	0.304099	0.898029	-1.33404	0.923711	1.44831	rc4
NM_001134473	KIAA0182	0.027078	-0.13063	-1.04942	-1.33257	-0.85147	-0.69824	rc4
NR_002449	SNORA65	-1.3297	-0.41901	-0.05558	-1.33171	-0.17058	-0.78929	rc4
NM_033010	PCBP4	0.096672	-0.50907	-0.41777	-1.32417	0.113128	-0.54989	rc4
NM_032222	FAM188B	-0.09573	-0.2601	-1.16628	-1.31706	-0.40217	-0.37492	rc4
NM_134263	SLC26A6	0.019233	-0.08537	0.123007	-1.31514	-0.60418	0.67113	rc4
NR_037872	LOC10023315	0.109639	-0.36725	-0.4517	-1.31197	0.085913	-0.08110	rc4
NM_014853	SGSM2	-0.13178	-0.45714	-0.20727	-1.31034	-0.03841	-0.30524	rc4
NR_023389	LINC00476	-0.11513	-0.18885	-0.00206	-1.30265	-1.0933	-0.92997	rc4
NM_001160407	TLCD1	-0.10799	-0.38316	-0.62073	-1.29214	-0.50522	-0.25508	rc4
NM_024335	IRX6	-0.19961	-0.39374	-0.56129	-1.28871	-1.2386	-1.15036	rc4
NM_001960	EEF1D	-0.5574	-0.59092	-0.76485	-1.28757	0.115942	0.68549	rc4
NM_139350	BIN1	-0.5821	-0.46036	-0.74335	-1.28724	-1.27391	-0.82070	rc4
NM_001195733	PIP5K1C	-0.08169	-0.07484	-0.46542	-1.28307	0.639895	1.08269	rc4
NM_001556	IKBKB	-0.49916	-0.79646	0.086684	-1.28056	-0.17579	0.44858	rc4
NR_026800	KIAA0125	-0.38854	-0.26828	-0.57527	-1.27957	-0.51381	-0.80300	rc4
NM_014770	AGAP2	-0.03328	-0.2747	-1.27169	-1.27875	-0.21938	-0.18995	rc4
NM_175875	SIX5	0.024821	-0.07853	-1.02376	-1.27838	-0.3262	-0.05769	rc4
NM_000445	PLEC	0.203315	0.313213	0.529135	-1.2753	0.74762	1.30139	rc4
NM_001199120_5	RPP21	-0.38655	0.01091	-0.63434	-1.26937	0.880672	0.07754	rc4
NM_212539	PRKCD	-0.1138	-0.3411	-0.87685	-1.25967	-0.81982	-0.77235	rc4
NM_213618	ST5	0.084109	-0.18025	-0.75903	-1.25633	0.762942	1.21624	rc4
NM_006478	GAS2L1	0.100302	-0.22719	-0.47589	-1.25499	-0.14782	-0.13478	rc4
NM_016457	PRKD2	0.406885	-0.16863	-0.9937	-1.25424	0.733766	0.70007	rc4
NR_026955	LINC00284	-0.18076	-0.12113	-0.20173	-1.24886	-0.38614	-1.14010	rc4
NM_001024683	ZNF688	-0.2132	-0.64718	-1.1287	-1.23856	-0.73474	-0.56181	rc4
NM_021005	NR2F2	0.217461	-0.87395	-0.2038	-1.23829	-0.57202	-0.51147	rc4
NM_001126339	THTPA	0.107361	0.048576	-0.05614	-1.2371	0.64645	0.64932	rc4
NM_001790	CDC25C	-0.14982	-0.61273	-0.65746	-1.23687	0.839707	1.07123	rc4
NM_212469	CHKA	0.555038	1.268555	0.722426	-1.23434	0.868926	0.81983	rc4
NM_015075	IQSEC2	-0.05842	-0.28038	-1.14482	-1.23053	0.351698	0.41925	rc4
NM_001164280	SLC37A4	-0.17863	0.049712	-0.63197	-1.23044	-0.95111	-0.75654	rc4
NM_012291	ESPL1	-0.21946	-0.39513	-0.82153	-1.22909	0.054936	-0.01443	rc4
NR_024584_1	LOC728875	-0.19884	-0.52043	-0.89898	-1.22816	0.073161	0.23724	rc4
NM_001160037	RHOBTB2	-0.09798	-0.44757	-0.92605	-1.22697	-0.94389	-0.82281	rc4
NM_020897	HCN3	-0.07213	-0.09148	-0.56598	-1.22317	-0.24206	-0.15528	rc4
NM_004630	SF1	0.304934	0.365227	0.367126	-1.22058	0.006814	0.18597	rc4
NM_001207009	ZNF497	-0.81167	-0.07363	-0.56639	-1.21694	-0.7788	-0.49530	rc4
NM_001166393	MTUS1	-0.49296	0.034952	-0.79366	-1.21524	-1.14602	-0.50447	rc4
NM_015219	EXOC7	-0.48052	0.223425	-0.48767	-1.21306	1.460039	1.56869	rc4
NM_148416	ATXN2L	-0.33354	-0.13017	-0.47779	-1.2104	-0.82954	-1.11929	rc4
NM_003101	SOAT1	-0.58663	-0.14033	-0.48578	-1.20782	-1.04255	-0.34749	rc4
NM_003655	CBX4	0.574663	-0.28163	-1.01704	-1.20516	-0.06108	0.19139	rc4

NM_017707	ASAP3	0.103805	-0.27788	-0.70006	-1.20489	-0.50249	-0.49025	rc4
NM_001114617	MGAT1	-0.26776	-0.54891	-1.13106	-1.20206	-0.172	-0.20275	rc4
NM_001198905	YY1AP1	-0.07295	-0.04432	0.30018	-1.19565	-0.46451	-0.88892	rc4
NM_014310	RASD2	-0.27881	-0.68955	-0.96426	-1.19537	0.027209	-0.13611	rc4
NM_001030001	RPS29	0.008916	-0.77062	-0.38566	-1.19404	0.964696	0.89457	rc4
NM_130802	MEN1	0.165625	-0.83366	-0.99177	-1.18999	-0.30814	0.49708	rc4
NM_000779	CYP4B1	0.621717	0.770517	0.807023	-1.18872	-0.21873	-0.87140	rc4
NM_080686_3	PRRC2A	-0.26238	0.029179	-0.50676	-1.18737	0.005286	0.02962	rc4
NM_003251	THRSP	0.072414	-0.5545	-1.05865	-1.18367	-0.81176	-0.74076	rc4
NM_001204159	SPHK2	-0.55663	-0.0435	-0.82659	-1.18303	-0.56756	0.33989	rc4
NM_016025	METTL9	-0.90174	0.895835	0.463138	-1.17979	-0.0938	0.37869	rc4
NM_001173988	C9orf86	-0.32616	-0.23058	0.258933	-1.17877	0.048283	0.20068	rc4
NM_198316	TENC1	-0.12193	-0.22704	-0.49537	-1.17585	-1.11412	-0.99920	rc4
NM_015503	SH2B1	-0.79653	-0.50988	-0.62703	-1.16848	-0.68851	-0.34922	rc4
NM_024824	ZC3H14	-0.45542	-0.51561	-1.00827	-1.16827	0.094438	-0.23072	rc4
NM_001198995	NADK	-0.38065	-0.02496	-0.31473	-1.16719	-0.06234	0.05162	rc4
NM_003512	HIST1H2AC	-0.00324	0.200051	-0.83288	-1.166	-0.19676	-0.01448	rc4
NM_001171948	KXD1	0.302911	-0.59787	0.18042	-1.1648	-0.02766	0.48007	rc4
NR_036513	LOC10013111	-0.1701	-1.11223	-0.80802	-1.16192	-0.72631	-0.97402	rc4
NM_017881	C9orf95	0.376209	0.109869	-0.51262	-1.16107	-0.99804	-0.84701	rc4
NM_153367	ZCCHC24	-0.168	-0.66219	-1.01291	-1.15865	-1.01384	-0.94853	rc4
NM_001012758	NUDT17	-0.44744	-0.37828	-0.45512	-1.15776	0.378976	0.62220	rc4
NM_182706	SCRIB	-0.66547	-0.12823	-0.06683	-1.15236	-0.16098	0.35150	rc4
NM_172088	TNFSF13	-0.33183	-0.83023	-1.06647	-1.14393	-0.12494	-0.22157	rc4
NM_001114752	CD55	-0.17503	-0.95373	0.171126	-1.14336	-0.35183	-0.03022	rc4
NM_014585	SLC40A1	-0.40044	-0.21726	-0.98668	-1.14089	-0.19509	-0.52657	rc4
NM_207343	RNF214	-0.39269	-0.14103	-0.50867	-1.13637	0.170636	0.03368	rc4
NM_005180	BMI1	-0.18708	-0.18115	-0.5787	-1.1363	0.003782	-0.34052	rc4
NM_201533	DGKZ	0.04812	0.009301	-0.62172	-1.13429	-0.61043	-0.65940	rc4
NM_032296	FLYWCH1	0.047215	-0.08838	-0.65206	-1.1337	-0.06261	0.45841	rc4
NM_031444	C22orf13	-0.16647	-0.50091	-0.9437	-1.1332	-0.76793	-0.64217	rc4
NM_001253764	SRA1	0.916203	0.87476	0.506494	-1.12124	-0.08449	0.60584	rc4
NM_001142450	SPNS1	-0.21242	-0.08793	-0.76177	-1.12085	-0.09438	-0.02703	rc4
NM_001110781	SLC35E2B	-0.18647	-0.20069	-0.76009	-1.12017	0.11403	0.20566	rc4
NM_139157	ST5	-0.07618	-0.4905	-0.77609	-1.12001	-0.56491	-0.35982	rc4
NM_182838	SLC35E2	-0.30933	-0.32561	-0.80377	-1.11945	-0.01675	0.07439	rc4
NM_001328	CTBP1	-0.60998	-0.22612	-1.0347	-1.11764	-1.09499	-0.75491	rc4
NM_004186	SEMA3F	-0.06553	-0.37751	-0.8598	-1.11617	-0.97643	-0.77449	rc4
NM_001242384	SYTL3	0.040953	-0.32913	-0.43867	-1.11574	-0.49349	-0.34242	rc4
NM_001674	ATF3	4.006445	3.568811	0.559998	-1.11532	0.83482	1.26626	rc4
NM_001172665	RAB40C	-0.43298	-0.63667	-0.39787	-1.11161	-0.52989	0.05452	rc4
NM_033025	SYDE1	0.365583	0.199501	-0.7895	-1.10141	0.564233	1.17279	rc4
NR_024510_1	LOC728855	-0.09783	-0.53397	-0.70565	-1.09786	0.042293	0.33524	rc4
NM_181800	UBE2C	-0.03839	-0.45513	-0.57018	-1.0957	-0.80923	-0.99714	rc4
NM_001964	EGR1	4.196052	1.676893	-0.39799	-1.09553	1.013872	1.06230	rc4
NM_001101676	SAMD12	-0.20352	-0.07019	-0.91342	-1.09184	-0.07855	-0.18926	rc4
NM_001130099	KIFC3	0.053873	0.027303	-0.78467	-1.08914	0.648597	1.05062	rc4

NM_148176	PPIL2	-0.14725	-0.10366	-0.39189	-1.08545	-0.62891	-0.37554	rc4
NM_001024401	SBK1	-0.0799	-0.13524	-0.57047	-1.0836	-0.11503	-0.05018	rc4
NM_001168222	TBC1D17	0.831961	0.709531	0.401709	-1.08276	0.895652	0.78985	rc4
NM_033418	METTL18	-0.68188	-0.1923	-0.22655	-1.08206	-0.21072	-0.16969	rc4
NR_024510	LOC728855	0.261073	-0.46068	-0.98515	-1.08008	-0.06728	0.28321	rc4
NM_013239	PPP2R3B	-0.07786	-0.05727	-0.54651	-1.07993	-0.58601	-0.54248	rc4
NM_014800	ELMO1	0.019123	-0.04575	-0.34077	-1.0785	-0.33306	-0.87317	rc4
NM_018173	PLEKHG6	0.050036	0.208575	-0.55818	-1.07727	1.278028	1.34517	rc4
NM_139034	MAPK7	0.117502	0.325345	-0.53625	-1.07694	-0.00614	0.29090	rc4
NM_013400	REPIN1	-0.48769	-0.64267	-0.78313	-1.07656	0.223704	0.26628	rc4
NM_001256435	PCYT2	-0.39048	-0.41518	-0.71276	-1.07433	-0.79961	-0.53042	rc4
NM_001127180	MYO7A	0.458917	0.301751	-0.07494	-1.07207	-0.03995	-0.68197	rc4
NM_001194990	MEPCE	0.063142	-0.33679	-0.7442	-1.07137	-1.02677	-0.83537	rc4
NM_001159576	SCNN1A	-0.76598	-1.00328	-0.8442	-1.06827	-1.00568	-0.35970	rc4
NM_001901	CTGF	4.952617	3.999998	-0.22628	-1.06682	0.311784	0.34731	rc4
NM_014428	TJP3	0.044011	-0.15435	-0.67366	-1.06594	0.243184	0.58595	rc4
NM_001204961	PBX1	-0.21472	-0.42202	-0.8808	-1.06502	-0.97093	-1.04258	rc4
NR_036519	C17orf62	-0.13185	-0.77239	-0.63933	-1.06305	-0.08492	0.52075	rc4
NM_005137	DGCR2	0.343766	-0.15987	0.778267	-1.06181	1.177052	0.92867	rc4
NM_178140	PDZD2	-0.12675	-0.08036	-0.99395	-1.06179	0.5919	0.38464	rc4
NM_177476	LYNX1	0.038929	-0.15617	-0.38385	-1.06118	0.225823	-0.25270	rc4
NM_013976	GCDH	-0.22837	-0.03517	0.664083	-1.05632	-0.3985	-0.16672	rc4
NM_001018078	FPGS	-0.24074	-0.1715	-0.74421	-1.05571	0.277365	0.23185	rc4
NM_018205	LRRC20	-0.16313	-0.13225	0.055592	-1.05162	0.637113	0.65232	rc4
NM_004302	ACVR1B	0.035678	-0.46087	-0.14234	-1.05047	0.071853	-0.19580	rc4
NM_006254	PRKCD	0.283526	-0.5107	-0.47265	-1.04878	-0.00562	0.31405	rc4
NM_016328	GTF2IRD1	0.043398	-0.06553	-0.73575	-1.04825	0.581889	0.61855	rc4
NM_006522	WNT6	0.410845	0.006546	-0.75856	-1.04634	-0.9987	-0.85824	rc4
NM_031215	CABLES2	-0.05674	-0.20541	-0.61801	-1.0413	-0.17176	-0.23232	rc4
NM_004961	GABRE	-0.61365	-0.38191	-0.41521	-1.03949	-0.55773	-0.27973	rc4
NM_001145338	ZBTB22	0.175425	0.590219	-0.59567	-1.03424	0.180797	1.01778	rc4
NM_001136039	NIF3L1	0.080265	-0.42486	-0.46019	-1.03263	-1.01399	-0.82711	rc4
NR_038924	LOC10050664	-0.24153	-0.82275	-0.92146	-1.03195	-0.80221	-0.66943	rc4
NM_001197246	BTN3A2	0.336381	0.277479	-0.06926	-1.03148	0.619375	0.31269	rc4
NM_024963	FBXL18	-0.07625	-0.20603	-0.79188	-1.02942	-0.15299	0.36083	rc4
NM_004952	EFNA3	-0.0451	-0.27928	-0.57582	-1.02927	-0.15445	-0.10558	rc4
NM_022479	WBSCR17	-0.12242	-0.21694	-0.50638	-1.02907	-0.89403	-0.55534	rc4
NM_004703	RABEP1	-0.3408	0.084501	-0.30936	-1.02441	-0.24734	-0.96409	rc4
NM_001243136	PELI3	0.340111	0.09914	-0.20014	-1.02186	-0.86278	-0.73192	rc4
NM_006492	ALX3	-0.2844	-0.31651	-0.75724	-1.02159	-0.85583	-0.54778	rc4
NM_003449	TRIM26	-0.32778	-0.4369	-0.73025	-1.02099	-0.59683	-0.33300	rc4
NM_058180	C21orf58	-0.06816	-0.00194	-0.43783	-1.01927	-0.67922	-0.97854	rc4
NM_052820	CORO2A	-0.10196	-0.19579	-0.60572	-1.01758	-0.15668	-0.26226	rc4
NM_001136191	KANK2	0.082796	-0.43544	-0.39374	-1.01732	-0.12468	-0.00837	rc4
NM_004689	MTA1	-0.2827	-0.42324	-0.30037	-1.01113	-0.54571	0.11605	rc4
NM_001079811	GLB1	0.235396	-0.02549	-0.37524	-1.01104	-0.15068	0.00736	rc4
NR_036518	C17orf62	1.239867	1.217656	-0.95885	-1.00972	1.118385	-0.34847	rc4

NM_015242	ARAP1	-0.26397	-0.61456	-0.8143	-1.00868	0.144371	0.45512	rc4
NM_001244937	MST1R	-0.47398	-0.13837	-0.78509	-1.00793	-0.06187	-0.21004	rc4
NM_001171251	C17orf53	-0.29654	-0.05714	-0.42093	-1.00722	-0.50764	-0.44955	rc4
NM_172056	KCNH2	0.056861	-0.48462	-0.48391	-1.0068	0.397899	-0.01238	rc4
NM_002957	RXRA	0.012003	-0.22358	-0.78321	-1.00436	-0.1255	0.06040	rc4
NM_001177639	DAG1	-0.27646	-0.45342	-0.68764	-1.00117	-0.55783	-0.24444	rc4
NM_005975	PTK6	0.235204	-0.99519	-0.38679	-1.00085	0.912211	0.89881	rc4
NM_024565	CCNJL	-0.20438	-0.98732	-1.00024	-1.00065	-0.10367	-0.06220	rc4
NM_001253389	ISYNA1	-0.03815	0.548862	-0.24522	-1.00062	0.15045	-0.28324	rc4
NR_046138	FAM91A2	-0.21418	-0.39798	-0.9372	-1.00004	0.302087	-0.35810	rc4
NM_002382	MAX	-0.74177	0.923344	0.00307	0.371962	-4.55031	-1.97639	rc5
NM_001142466	GPT2	-0.82473	-1.27213	-1.05645	-1.45933	-3.95666	-3.50771	rc5
NM_001171136	ZBED1	-3.19342	0.248751	-0.80589	-1.35923	-3.5989	-0.71652	rc5
NM_201627	TRIM41	-0.21012	-0.41913	-0.62312	-0.64893	-3.3577	-1.58817	rc5
NM_001171136_1	ZBED1	-0.03583	0.642966	0.100668	-1.03957	-3.24191	-1.40376	rc5
NM_201277	CNN2	-1.45024	0.041457	-0.35595	0.437081	-3.21289	-2.32656	rc5
NM_024861	C2orf54	-0.96434	-1.07987	-0.81702	-0.89972	-3.17691	-1.85050	rc5
NM_001164145	CHAMP1	0.050088	-0.26625	-0.84409	0.085421	-3.16376	-2.12494	rc5
NR_000021	SNORD32A	-0.87618	-0.48737	-0.40725	-0.29419	-3.15485	-0.94244	rc5
NM_001144030	NAT10	-0.59956	-0.02855	-0.35117	-0.79118	-3.14084	-0.76727	rc5
NM_001039589	DPH2	-1.83995	-1.46824	-1.44977	-0.83732	-3.09562	-1.87748	rc5
NR_000028	SNORD83B	-0.82967	0.579102	0.811787	0.395773	-3.01544	-1.74729	rc5
NM_031372	HNRPDL	-0.33096	0.632023	0.588164	-0.01303	-2.9884	-2.96843	rc5
NM_001135048	JDP2	0.200695	-0.6491	-1.8178	-1.66115	-2.94526	-1.05302	rc5
NM_178830	C19orf47	-1.34906	-0.40318	0.761005	-0.08124	-2.93242	0.57128	rc5
NM_178031	TMEM132A	0.08307	0.079721	-0.06492	-0.37676	-2.91768	-0.83442	rc5
NR_002579	SNORD74	-0.77509	-0.27439	-2.55328	-0.84445	-2.90403	-2.02255	rc5
NM_001956	EDN2	1.669989	0.861968	-0.49259	-0.97815	-2.90185	-2.68497	rc5
NM_001127687	ADIPOR1	0.01167	-0.07055	-0.14619	-0.09962	-2.87241	-0.53048	rc5
NM_001009998	SSBP4	-0.24011	-0.10635	-0.09839	-1.26973	-2.82721	0.70520	rc5
NM_001043351	TPM3	-0.64184	-0.04058	-1.10477	-0.2004	-2.81802	0.16893	rc5
NM_182678	UBE2E3	-1.52748	-2.50966	-1.60247	-1.27133	-2.80292	-2.75498	rc5
NR_039910	MIR4754	-0.18859	-0.61361	-1.53477	-2.54176	-2.79958	-2.08361	rc5
NM_001122898	CD99	-1.00755	-1.02344	-1.93511	-2.01999	-2.77299	-1.12683	rc5
NM_001256484	EPHX2	-0.20648	-0.51414	-0.62961	-0.91512	-2.76964	-2.64566	rc5
NM_145905	HMGA1	-1.48271	-0.80905	-0.87564	-1.34138	-2.75491	-1.95337	rc5
NM_001255979	CREB3L4	0.335312	-0.24155	-0.5578	-0.92336	-2.71022	-2.30123	rc5
NM_001177842	SRPR	-0.09183	0.093953	-0.54752	-0.19691	-2.69558	-1.84635	rc5
NM_080648	APEX1	2.168345	2.282854	1.043338	1.797867	-2.65447	0.48102	rc5
NM_001172223	MOB2	-0.03505	-0.5326	-0.65898	-1.69496	-2.63008	-1.99554	rc5
NM_001256618	TUBGCP2	0.191495	0.329501	-0.47974	-1.30063	-2.60533	-0.84225	rc5
NR_038431	LOC10050605	-0.26781	-0.8656	-0.1919	-1.0516	-2.56755	-2.15487	rc5
NM_001099696	REPIN1	-0.21793	-2.03042	-1.98545	-1.7468	-2.52482	0.07553	rc5
NM_001115156	GDI2	0.079641	-0.45762	-1.30301	-1.27632	-2.5216	-1.68786	rc5
NM_005808	CTDSPL	-0.90786	-0.50517	-0.56158	-0.84534	-2.51295	-1.22822	rc5
NM_001199957	NDUFB5	-0.99819	-1.3632	-0.40213	-0.37708	-2.50658	-0.81212	rc5
NM_001024957	BRMS1	0.238755	-0.5766	-1.24146	0.530916	-2.47291	-1.93962	rc5

NM_001145461	YIF1B	-1.09714	-1.87516	-0.70781	-1.62286	-2.46502	-2.16103	rc5
NM_001144036	TMEM25	-1.06994	-0.74907	-2.10927	-2.33223	-2.46356	-2.03744	rc5
NM_078626	CDKN2C	-0.17356	-0.96142	-1.94376	-0.82975	-2.44066	-2.42468	rc5
NR_028402	CSRP2BP	0.263188	-0.58251	-1.1566	-2.01544	-2.39831	-0.74965	rc5
NR_046373	P2RX4	-0.72086	-1.28926	-1.94549	-1.4676	-2.3924	-1.28056	rc5
NM_001242485	EIF1AD	-0.50613	-0.17513	-1.15205	-0.85836	-2.38455	-1.40843	rc5
NM_138439	FLYWCH2	0.12464	-0.08891	-0.63804	-0.6996	-2.36258	-0.47677	rc5
NM_001006115	IP6K1	-0.05011	-0.65864	-0.57334	-1.05328	-2.32582	-1.55376	rc5
NR_023344	RNU6ATAC	-0.05118	-1.26806	0.286317	0.167709	-2.32535	0.33301	rc5
NM_032034	SLC4A11	-1.02306	-0.46931	0.265176	-0.45297	-2.32342	-0.84626	rc5
NM_001204468	RBM10	-0.82294	0.472175	-0.24227	0.827232	-2.31629	-1.00901	rc5
NM_001206974	DHPS	-0.17773	-0.26814	-0.57683	-0.91062	-2.27675	-1.60383	rc5
NM_001032383	PQBP1	-1.53238	-0.74544	0.272068	-0.45997	-2.26431	-1.49603	rc5
NM_025078	PQLC1	-0.73831	-0.25603	-0.7916	0.169088	-2.24465	-0.54237	rc5
NM_012446	SSBP2	0.204452	0.10286	-0.25695	-0.25294	-2.23086	-0.84843	rc5
NM_013411	AK2	0.350299	-0.40733	-0.12985	0.46277	-2.22139	-1.45472	rc5
NM_001256532	MSTO1	0.177429	0.184935	0.405412	1.498412	-2.21711	0.56599	rc5
NR_004399	SNORD86	-0.33253	0.78003	-1.17633	0.692429	-2.2039	-1.05833	rc5
NM_001077366	POMT1	-1.91872	-0.71151	-0.86827	-1.51261	-2.20063	-0.91422	rc5
NM_001039614	C15orf59	-0.32885	-0.19732	-0.57677	-1.20222	-2.186	-2.11556	rc5
NM_001101312	TMEM176B	0.459574	-0.46381	-1.39279	-1.98388	-2.17734	-1.45442	rc5
NM_001001992	USP16	-0.30476	-1.16458	-1.28453	-0.33911	-2.17275	-1.75961	rc5
NM_001039548	KLHL35	0.037999	-0.17522	-0.51622	-0.17053	-2.16844	-1.81948	rc5
NR_046255	FGD5-AS1	0.085088	-0.30166	-0.59333	-0.19477	-2.16326	-2.12987	rc5
NM_024311	MFSD11	-0.15861	-0.43877	-0.61877	0.337688	-2.162	-0.79313	rc5
NM_001255981	CREB3L4	-0.208	-0.0553	-0.60043	-0.86538	-2.14142	-2.01212	rc5
NR_027889	TMEM189	-0.23582	0.083603	0.004513	0.534703	-2.13625	-0.23032	rc5
NM_004609	TCF15	0.166181	0.11454	-0.22961	-0.11593	-2.10865	-1.41962	rc5
NM_001253850	VTCN1	0.897615	1.138679	0.719545	0.644811	-2.09958	-1.35468	rc5
NM_001190791	ZNF317	0.230922	0.613188	0.447891	0.557377	-2.09498	-1.91158	rc5
NM_001039708	SDCCAG3	-0.16401	-0.31379	-0.40742	-1.04669	-2.08532	-1.27093	rc5
NR_002976	SNORA44	1.367001	0.968049	1.617943	1.329816	-2.08258	0.18722	rc5
NR_033675	VDAC2	1.75037	2.128805	0.06522	2.363551	-2.08249	1.12405	rc5
NM_001145396	ALDH16A1	0.583979	-0.35518	-0.8776	-0.20141	-2.07372	-1.07165	rc5
NM_017454	STAU1	-1.7085	-0.85182	-1.95445	-1.63743	-2.06756	-0.91176	rc5
NM_147148	GSTM4	0.290134	-0.07718	-0.25028	-0.80089	-2.02697	-1.69499	rc5
NM_001024226	ARF1	0.240891	-0.44179	-0.17035	0.180372	-2.02415	0.59524	rc5
NM_001174152	RABEPK	0.461056	-0.10915	0.292351	-0.09507	-2.02205	-1.05524	rc5
NM_022970	FGFR2	0.029045	-0.09254	-0.66214	-1.05563	-2.00893	-1.93077	rc5
NR_024580	LOC1001311	0.063707	0.038137	-0.46492	-0.84749	-2.00251	-1.20113	rc5
NM_001195731	CHPF	-0.49015	-0.59292	-1.29143	-0.7207	-2.00225	-1.12993	rc5
NM_001243885	TOB1	-0.07413	-0.45854	-1.96055	-1.74075	-1.9865	-1.17297	rc5
NR_046290	LOC1002890	0.669281	0.334588	-0.33647	0.047121	-1.95984	-0.78897	rc5
NM_001002812	PDE4DIP	0.000146	0.123076	-0.22408	-0.1926	-1.93147	-1.93146	rc5
NM_182826	SCARA3	0.247617	0.228517	-0.32917	-1.10164	-1.93136	-1.40530	rc5
NM_021830	C10orf2	-0.63114	-0.79704	0.194169	-0.00957	-1.92266	-0.54742	rc5
NM_147173	NUDT2	-0.2216	-1.11597	-0.49149	-0.4	-1.91086	-0.74891	rc5

NM_020820	PREX1	0.002141	-0.14399	-0.42988	-0.99719	-1.90689	-1.84858	rc5
NM_194429	FGFR1OP	-0.27978	-0.28124	-0.27599	-0.04773	-1.90377	-0.84600	rc5
NM_001160210	SLC25A13	-1.33257	-0.42677	-0.77325	-1.44161	-1.89908	-1.47740	rc5
NR_038396	LOC10050646	-0.18692	-0.01527	-0.53143	-0.62791	-1.8936	-1.60940	rc5
NM_001165031	DTYMK	-0.02175	-1.03754	-0.13786	-1.48023	-1.8924	-1.29679	rc5
NM_001127457	CRY2	0.409346	-0.76892	-0.84518	-1.23565	-1.89053	-0.06959	rc5
NM_001012664	SLC3A2	0.226326	-0.18373	-0.07495	-0.17892	-1.87989	-0.75633	rc5
NM_001035505	BOLA3	-0.14543	-0.41105	-0.13541	-0.25584	-1.87605	-0.99739	rc5
NM_001033522	CSTF1	-0.29315	-1.29005	-0.53077	0.134836	-1.86302	-1.41738	rc5
NM_024979	MCF2L	-0.28167	-0.02262	-0.29587	-1.28091	-1.85002	-1.82709	rc5
NM_033015	FASTK	-0.62507	-0.66504	-0.67286	-0.66362	-1.83329	-1.60102	rc5
NM_000260	MYO7A	-0.49028	-0.47921	-0.31728	-0.53733	-1.82569	-1.68674	rc5
NM_006538	BCL2L11	-0.70965	-0.48307	-0.80321	-0.89813	-1.82314	-1.55643	rc5
NM_176794	MRPL43	0.318418	-0.53446	-0.00834	0.160189	-1.81816	-0.77338	rc5
NM_001204492	CAMK2G	-0.17821	0.630271	-0.29451	-0.13	-1.81536	-1.04365	rc5
NM_006092	NOD1	-0.19388	-0.58803	-1.26998	-1.27581	-1.81509	-1.67046	rc5
NM_001023587	ABCC5	-0.0997	-0.04307	-0.35832	-0.43126	-1.80902	-1.74142	rc5
NM_016487	C6orf203	-0.32071	-0.39736	-0.61589	-0.60446	-1.80483	-1.43252	rc5
NM_001162895	KIAA0040	-0.23046	1.01937	-0.32609	-1.16647	-1.80115	0.46682	rc5
NM_032637	SKP2	0.116655	-0.43044	-1.29105	-0.82323	-1.79941	-1.79939	rc5
NM_001184883	PLCB3	-0.33434	-0.32659	-1.04934	-0.54865	-1.77949	-1.48630	rc5
NM_017964	SLC30A6	-0.24017	0.25986	-0.47324	-0.45767	-1.77591	-0.83251	rc5
NM_181515	MRPL21	-1.56962	0.094671	-0.45573	0.382297	-1.75761	-0.66298	rc5
NM_032050	PATZ1	-0.60324	-0.69842	-0.93506	-1.34131	-1.75661	-0.73968	rc5
NM_001161473	ALDH3B1	0.463901	-0.03686	-0.35308	0.790202	-1.75558	-0.42250	rc5
NM_002315	LMO1	-0.24135	-0.49909	-0.86932	-0.97288	-1.74877	-1.39713	rc5
NM_001144887	CITED1	-0.63968	0.196466	-0.08835	-0.43167	-1.74708	-1.31488	rc5
NM_001009877	BRD9	-0.07406	0.07483	-0.44353	-0.2266	-1.74024	-1.50724	rc5
NM_001098509	SGSM2	-0.10521	0.347862	-0.13287	-0.03762	-1.73839	-0.83803	rc5
NM_001145901	SARS2	-1.68124	-1.61976	-0.68384	-1.39902	-1.73475	-1.41248	rc5
NM_001102371	FOXRED2	0.242022	0.007082	-0.20249	-0.43737	-1.72372	-1.57667	rc5
NM_007155	ZP3	-0.17399	0.153504	0.199741	0.042206	-1.7213	-0.82015	rc5
NR_037669	GGCT	-0.14043	0.012692	-0.28427	-0.1357	-1.71789	-1.49269	rc5
NM_001131036	ZCCHC9	-1.68572	-0.60373	-0.31846	0.115385	-1.71241	-1.21240	rc5
NM_015590	GPATCH4	0.014207	-0.15224	0.00357	-0.26967	-1.7124	-1.33379	rc5
NM_205861	DHDDS	0.186271	-0.23977	0.06869	0.188543	-1.70751	0.02706	rc5
NM_153645	NUP50	0.039082	0.373157	-0.06039	0.588228	-1.69136	-0.73611	rc5
NM_181843	NUDT8	0.011149	-0.42944	-0.55073	0.658281	-1.67526	-0.57274	rc5
NM_001337	CX3CR1	0.034851	-0.2139	-0.93824	-0.71708	-1.6748	-1.54018	rc5
NR_027383_1	AKAP17A	-1.15428	0.161854	-1.18588	0.194635	-1.66975	0.08861	rc5
NM_001224	CASP2	-0.58105	-0.70805	-0.81788	-1.06119	-1.66504	-1.19779	rc5
NM_000296	PKD1	-0.12873	0.229604	-0.78309	-1.28124	-1.66486	-1.25464	rc5
NM_003869	CES2	-0.26512	-0.30073	-0.37325	-0.69283	-1.66218	-1.32384	rc5
NR_002448	SNORD36A	-0.54032	-1.30958	0.325822	0.175199	-1.65635	-1.36692	rc5
NM_001008749	RAB19	-0.23027	-0.68817	-0.95756	-1.44234	-1.65549	-1.28516	rc5
NM_033487	CDK11B	-0.35172	-0.32525	-0.07839	-0.38426	-1.64813	-0.75529	rc5
NR_002919	SNORA5A	0.088542	0.948138	0.325115	0.711965	-1.64472	0.26819	rc5

NR_036543	PPIE	-0.04088	-0.47406	-1.25918	0.056774	-1.64001	-0.00716	rc5
NR_033187	PIN4	-0.40627	-1.58216	-0.83753	-0.78615	-1.63211	-1.13851	rc5
NR_002586	SNORA63	-1.08196	0.244964	-0.44685	-0.68255	-1.63181	-1.37698	rc5
NM_003519	HIST1H2BL	0.276857	-1.57626	-0.56077	-0.47387	-1.6303	-0.62714	rc5
NR_037645	TMX2	-0.13923	-0.36731	-0.24949	-0.59878	-1.60719	-1.32936	rc5
NM_005700	DPP3	-0.93517	-0.12548	-0.12185	-0.16082	-1.6061	-0.13205	rc5
NM_001014839	NCDN	-0.50083	-0.28737	-0.3503	0.325371	-1.59933	-0.03764	rc5
NR_036627	RBM5	0.447727	-0.55061	-0.78353	-0.31436	-1.59785	-0.77063	rc5
NM_003531	HIST1H3C	-0.59779	-0.30999	0.177042	-0.4106	-1.59366	-1.30962	rc5
NM_032052	PATZ1	-0.87046	-0.81547	-1.18804	-0.57193	-1.59107	-1.21755	rc5
NM_033388	ATG16L2	-0.04434	0.637864	0.35392	0.165528	-1.58633	-0.84808	rc5
NM_001256676	STOML1	0.286216	-0.82448	-0.59646	-0.42022	-1.57866	-0.19549	rc5
NM_006624	ZMYND11	-0.34213	-0.09089	-0.70378	0.14504	-1.57347	-0.75870	rc5
NM_019021	C11orf71	-0.19572	-0.31572	-0.64488	-0.42393	-1.57236	-1.28569	rc5
NM_152856	RBM10	-0.81578	0.355716	0.95308	0.406784	-1.5657	-0.38684	rc5
NM_001134485	TOMM5	0.313123	0.410017	0.439286	0.728715	-1.56546	0.01781	rc5
NM_001163735	MYO19	-0.15899	-0.08144	-0.01392	-0.45145	-1.56021	-1.53543	rc5
NM_198242	EIF4G1	-0.28871	-0.67322	-0.09794	-0.1451	-1.55906	-0.82557	rc5
NM_002840	PTPRF	-0.03831	-0.05661	-0.35715	-0.55853	-1.55778	-1.00846	rc5
NM_014189	ADD1	0.046841	-0.4804	-0.31904	-0.5195	-1.55729	-1.21118	rc5
NR_015366	LOC388796	0.459382	0.535317	0.612021	0.4756	-1.53664	-0.45494	rc5
NM_001172819	PGM1	-0.00323	0.814147	0.301556	-0.74309	-1.5316	-0.17077	rc5
NM_153025	C16orf55	-0.07202	-0.23482	-0.63965	-0.5897	-1.51647	-0.76746	rc5
NM_001256378	MCMBP	-0.74463	0.068161	-0.08841	-0.40672	-1.51458	-1.17896	rc5
NM_001242659	C1orf233	0.003258	-0.25119	-0.33863	-0.21867	-1.51111	-1.32796	rc5
NM_001143819	TPCN1	-1.36781	-1.41805	-0.30602	-1.03369	-1.50979	0.29521	rc5
NM_001038618	NARF	-0.04982	-0.57278	-0.14177	-0.83686	-1.50831	-0.57999	rc5
NM_183415	UBE3B	-0.33386	-0.53865	-0.6103	-0.63909	-1.50519	-1.05613	rc5
NM_001172439	ENDOU	0.178903	0.199262	-0.05221	0.359947	-1.50497	-1.07679	rc5
NM_203387	RNH1	-0.42832	-0.97908	0.319158	-0.36436	-1.50188	0.03927	rc5
NM_001142545	ADCK1	0.681212	0.071625	-0.50279	-0.11893	-1.49283	-0.09717	rc5
NM_024660	IGFLR1	0.147803	-0.35404	-0.61089	-0.81387	-1.48942	-1.38517	rc5
NM_013430	GGT1	-0.53514	0.040548	-0.40791	-0.58542	-1.4889	-0.69537	rc5
NM_001190201	CES4A	-0.04776	-0.43853	-0.14813	0.339867	-1.48734	-0.78766	rc5
NM_198722	AMIGO3	0.09098	0.415668	0.186236	-0.54999	-1.48232	-0.98461	rc5
NM_032620	GTPBP3	-1.13555	-0.49422	-0.07629	-1.11841	-1.4819	-0.90497	rc5
NM_001082488	DHRS4L1	0.061718	-0.5008	-0.47755	-0.24328	-1.48143	-1.27314	rc5
NM_016373	WWOX	0.093141	-0.02621	-0.12102	0.065352	-1.48109	-1.33301	rc5
NM_001139467	TBL1X	0.040117	0.164664	-0.15538	-0.30915	-1.48062	-1.37539	rc5
NM_004332	BPHL	0.432228	-0.36409	0.502198	0.115767	-1.47554	-1.33878	rc5
NR_026874	LOC10013041	-0.29815	0.008827	0.014976	-0.61993	-1.4724	-0.93884	rc5
NM_001242787	BRF1	-0.00401	-0.58156	-0.48779	-0.66627	-1.47112	-1.45887	rc5
NM_001005862	ERBB2	-0.13178	-0.46088	-0.69653	-1.05533	-1.46942	-0.83682	rc5
NM_025256	EHMT2	-0.82248	0.530207	-0.20933	-0.59195	-1.46543	0.23252	rc5
NM_024324	CRELD2	-0.00657	-0.13505	-0.11217	-0.14821	-1.45939	-1.17586	rc5
NM_001719	BMP7	0.087238	-0.18144	-0.27863	-0.5904	-1.45933	-1.44661	rc5
NM_016499	TMEM216	-0.57998	0.221467	-0.24567	-0.36456	-1.45912	0.49806	rc5

NM_001134335	EIF2AK1	0.449182	0.152845	0.436385	-0.81736	-1.45887	-0.81784	rc5
NM_024848	MORN1	-0.38031	-0.32134	-0.86564	-0.58299	-1.45315	-1.39020	rc5
NM_006416	SLC35A1	-0.43313	0.238024	-0.69145	-0.87128	-1.45311	-1.20828	rc5
NM_001114600	C1orf144	-0.74578	-0.88933	-0.01848	0.039359	-1.45205	-0.94853	rc5
NM_007244	PRR4	-0.23593	-0.15954	-0.8917	-0.90052	-1.44979	-0.55257	rc5
NM_001008860	NIPA2	0.203411	-0.66641	-0.29817	0.072476	-1.44746	-1.01989	rc5
NR_027058	LINC00338	-0.12908	0.224206	0.337597	-0.13482	-1.44201	-0.96047	rc5
NM_145791	MGST1	0.622485	0.372223	-1.05724	1.370173	-1.44182	-0.67285	rc5
NM_001142677	CHID1	-0.11868	-0.69631	-0.70453	-0.16527	-1.4393	-0.05674	rc5
NM_001255980	CREB3L4	-1.01064	-0.52643	-0.59079	-1.3959	-1.43647	-0.30148	rc5
NM_001042632	SNX21	0.189094	-0.98518	-0.03658	-0.53535	-1.42889	0.10506	rc5
NM_024649	BBS1	-0.17886	-0.18967	-0.16438	-0.21657	-1.42872	-1.26772	rc5
NM_001003396	TPD52L1	-0.59951	-0.29017	-0.23154	-0.21004	-1.42842	-1.18153	rc5
NM_018697	LANCL2	0.11611	0.045215	0.299102	0.518824	-1.42746	-1.26242	rc5
NM_001184897	PHF8	0.086436	-0.54225	0.389963	-0.29132	-1.42085	-0.99167	rc5
NM_001113496	42987	0.15895	0.757294	1.422676	1.680908	-1.41932	-0.58694	rc5
NM_003343	UBE2G2	-0.26978	0.102225	0.254023	0.108928	-1.41737	-1.15962	rc5
NR_046347	USP39	-0.13449	-0.08478	-0.44058	-0.39024	-1.41417	-0.68875	rc5
NR_026723	SARNP	-0.06068	0.423767	0.389446	0.391198	-1.41386	-0.45560	rc5
NM_022492	TTC31	-0.55409	-0.97456	-0.88582	-0.88299	-1.41349	-1.25891	rc5
NR_040055	LOC10050668	1.146223	0.349404	-0.15764	0.255554	-1.41038	-0.77697	rc5
NM_018054	ARHGAP17	-0.16572	-0.80542	-0.24922	-0.38295	-1.40476	-1.15493	rc5
NM_001203247	EZH2	-0.58009	0.480158	-0.517	-0.88607	-1.40428	-1.23198	rc5
NR_046326	KDM5B-AS1	-0.40763	-0.03021	-0.86603	0.141853	-1.39701	-0.48018	rc5
NM_001178042	DBI	-0.22989	0.020369	0.054697	0.735182	-1.391	-0.63000	rc5
NM_006328	RBM14	0.043855	-0.03078	0.244345	0.474447	-1.39036	-1.16326	rc5
NM_022116	FIGNL1	0.45464	-0.06299	0.012235	-0.41906	-1.39028	-1.12426	rc5
NM_198573	ENHO	-0.09737	-0.34562	-0.39252	-0.53714	-1.38546	-1.31679	rc5
NM_212502	CDK18	0.185882	-0.01468	-0.69828	-0.77872	-1.38393	0.10894	rc5
NR_002977	SNORA45	-0.0801	0.963125	0.550776	0.656697	-1.38047	-0.90529	rc5
NM_022485	MTMR14	-0.40432	-0.88544	-0.81509	-0.4079	-1.37999	-0.40673	rc5
NM_001255978	CREB3L4	-0.313	-0.55751	-0.50831	-1.35275	-1.3769	0.32224	rc5
NR_039666	MIR4461	-1.29495	-0.6025	-0.95145	-0.03835	-1.37581	-0.89835	rc5
NM_138458	WDR92	-0.00587	-0.27969	-0.8892	-0.70878	-1.37472	-0.99993	rc5
NM_002660	PLCG1	-0.33655	-0.41369	-0.4295	-0.60841	-1.37464	-1.21790	rc5
NM_018262	IFT122	-0.29715	-0.53299	-0.46711	-0.8406	-1.37084	-1.04201	rc5
NM_130468	CHST14	0.074926	-0.06204	-0.27269	-0.332	-1.36958	-0.94793	rc5
NM_001013843	SLTM	-0.10228	-0.02983	-1.12776	-0.53458	-1.36647	-0.90793	rc5
NM_001172566	MYD88	-1.08103	-0.91814	-0.74098	-0.88473	-1.36403	-1.02958	rc5
NM_001135023	ELMOD3	-0.34169	-0.48448	1.193252	0.905039	-1.35546	-0.05171	rc5
NM_032494	ZC3H8	-0.10292	0.505706	0.065476	0.274591	-1.34524	-1.31718	rc5
NM_001171806	PAPD7	-0.40454	0.410711	0.140732	-0.10859	-1.34427	-1.28037	rc5
NM_181519	SYT15	-0.10551	-0.30561	-0.37251	-0.47888	-1.34301	-1.25897	rc5
NM_001165417	SLC25A11	0.446947	0.014697	-0.54037	-1.29033	-1.34063	-0.64623	rc5
NM_004584	RAD9A	0.022968	-0.19454	-1.16977	-1.05702	-1.33897	-1.10176	rc5
NM_197964	C7orf55	0.00276	-0.53929	-0.37642	-0.35901	-1.33495	-0.99795	rc5
NM_001184917	PCYT2	-0.0613	-0.38497	-0.58431	-0.8827	-1.33193	-0.87377	rc5

NR_024600	DIABLO	-1.27166	0.275632	-1.1183	-0.27486	-1.33091	-0.35271	rc5
NM_001256469	KAT6B	-0.90968	-0.39226	-1.17371	-1.09806	-1.32885	-1.07290	rc5
NM_001001936	AFAP1L2	-0.1786	-1.02931	-0.35907	-1.3222	-1.32624	-0.16678	rc5
NM_001244580	TRRAP	-0.95584	-0.32857	0.018206	0.201534	-1.32372	-0.19667	rc5
NM_007363	NONO	-0.64022	-0.87209	-0.54129	-1.00989	-1.32062	0.01907	rc5
NM_182915	STEAP3	-0.45871	-0.17314	-0.51831	-0.87245	-1.31924	-1.10643	rc5
NM_016830	VAMP1	0.695587	0.379131	0.552695	0.495792	-1.31751	-0.90217	rc5
NM_001033059	AMD1	-0.50248	1.260177	0.838661	-0.53511	-1.31483	-1.19713	rc5
NM_032711	MAFG	1.047994	-0.72366	-0.49529	-0.96348	-1.313	-0.88883	rc5
NM_018134	IQCC	0.014537	-0.23128	0.193244	0.227942	-1.31291	-1.08395	rc5
NM_001199803	CENPO	0.496456	0.117902	0.225793	0.145911	-1.30854	-0.87262	rc5
NM_001198973	KIAA1522	-0.4905	-0.87511	-0.8839	-0.84774	-1.3076	-0.08874	rc5
NM_001145445	SCAF4	0.299017	-0.56742	0.331119	0.614398	-1.30755	-0.67970	rc5
NR_002585	SNORA52	1.107651	0.202383	-0.59498	1.004159	-1.30145	0.16249	rc5
NM_021734	SLC25A19	-0.00078	-0.94803	-0.11213	-0.27585	-1.30026	-0.97576	rc5
NM_006724	MAP3K4	-0.36746	-0.74134	-1.15229	0.373807	-1.2996	-1.26689	rc5
NM_025215	PUS1	0.18023	-0.13204	-0.03751	-0.7443	-1.29836	-0.42065	rc5
NM_001037984	SLC38A10	-0.05855	0.022144	-0.15087	-0.6904	-1.2968	-0.86093	rc5
NM_001198904	YY1AP1	-0.48135	0.27569	0.908316	-0.08364	-1.29502	-0.69030	rc5
NM_173074	PIGF	1.137482	1.150445	0.627573	-0.49139	-1.29223	-0.68460	rc5
NM_005243	EWSR1	1.01668	0.760412	1.211332	2.683531	-1.29042	-0.71356	rc5
NM_001243526	HYI	0.367312	-0.46139	-0.69115	0.756716	-1.28776	0.67991	rc5
NR_046240	LOC10050610	-0.33735	-0.44965	0.041459	-0.44304	-1.28158	-0.85485	rc5
NR_024469	LOC10013098	-0.45632	-0.69309	-0.18298	-0.90685	-1.28063	-1.11986	rc5
NM_032442	NEURL4	0.064725	-0.3269	-0.19537	-0.6335	-1.27991	-0.05375	rc5
NM_145294	WDR90	0.290355	0.253207	-0.08537	-0.23665	-1.27877	-1.09936	rc5
NM_001042680	MFSD12	-0.20831	0.104802	-0.46604	-0.60046	-1.27876	0.16802	rc5
NM_173620	HEXDC	0.222752	0.522664	0.12865	-0.03092	-1.27682	-1.18659	rc5
NM_001256335	PTGES2	-0.15935	-0.77489	-1.16864	-1.16594	-1.27501	-0.01943	rc5
NM_001128591	PSMG4	0.576051	-0.3264	0.716422	0.683544	-1.27199	-0.57600	rc5
NM_000327	ROM1	-0.24417	-0.37589	-0.80381	-0.14995	-1.2719	-0.90879	rc5
NM_020703	AMIGO1	-0.15504	-0.4741	-0.44392	-0.42953	-1.27078	-1.22679	rc5
NM_012327	PIGN	-0.93052	-0.25781	-0.58833	-0.405	-1.26654	-0.62137	rc5
NM_001129828	CSAG3	-0.49522	-0.52984	-0.86794	-0.42415	-1.26603	-0.72980	rc5
NM_001206646	AKIP1	0.088151	-0.30848	-0.93126	-0.41274	-1.26515	-0.44877	rc5
NM_001193646	ATF5	0.372795	0.406948	0.522805	0.388948	-1.26449	-0.90618	rc5
NR_003697	SNHG15	0.347451	0.348224	0.305358	0.284437	-1.26053	-0.88902	rc5
NM_001036646	HHLA3	0.378657	-0.25067	0.577127	0.608888	-1.25615	-0.50223	rc5
NM_001254755	CYB5D2	-0.15242	-1.21717	-1.08713	-1.05343	-1.25595	-0.95506	rc5
NM_015029	POP1	0.148971	-0.04119	0.996164	0.692607	-1.25471	-1.07489	rc5
NM_001195150	LOC10013070	-0.39065	0.111945	-0.2567	-0.3012	-1.25343	-0.96671	rc5
NM_001017989	OPA3	0.152583	-0.04257	-0.28635	-0.04505	-1.25174	-0.98248	rc5
NM_001003688	SMAD1	-0.60227	-0.49185	-0.57874	-0.77664	-1.25149	-0.99342	rc5
NM_001159280	ADAL	0.068597	0.176536	0.023211	-0.26371	-1.25096	-1.24471	rc5
NM_001099286	FAM54A	-0.06155	-0.41554	-0.00964	-0.1438	-1.24988	-0.82805	rc5
NM_018956	C9orf9	-0.25554	-0.27008	-0.55955	-0.2095	-1.24938	-0.86053	rc5
NR_036260_1	MIR1184-3	2.177891	-0.17386	1.503596	3.985468	-1.24868	0.46433	rc5

NM_206862	TACC2	-0.19883	-0.03276	-0.33223	-1.13968	-1.2474	-1.12610	rc5
NM_004707	ATG12	-0.10947	-0.22249	-0.21777	0.366286	-1.2449	-0.04779	rc5
NM_002861	PCYT2	0.22973	0.093323	-0.56002	-0.20335	-1.2417	-1.11567	rc5
NR_003013	SCARNA16	-0.52484	-0.57971	0.232653	-0.21319	-1.23953	-0.47107	rc5
NM_006133	DAGLA	0.030917	-0.34529	0.249553	0.200497	-1.23233	-0.84990	rc5
NM_181508	HPS5	-0.10694	-0.04565	-0.00073	-0.15406	-1.23001	-0.84418	rc5
NM_198083	DHRS4L2	0.357757	0.08148	0.048393	0.2269	-1.22981	-0.36993	rc5
NM_152988	SPPL2B	-0.38474	-0.01509	0.021919	-0.52055	-1.22883	-0.71754	rc5
NR_026593	TMX2	-0.03935	-0.00835	0.048695	-0.64885	-1.22781	-0.94997	rc5
NM_001005464	HIST2H3A	-0.33586	-0.51057	-0.62105	-1.02854	-1.22709	-0.41977	rc5
NM_002138	HNRNPD	-0.14463	-0.26238	0.392215	0.679917	-1.2269	-0.96511	rc5
NM_013393	FTSJ2	-0.0981	-0.25833	-0.11788	0.06185	-1.22316	-1.19457	rc5
NM_007042	RPP14	-0.29041	-0.29957	0.045773	0.253675	-1.21964	-1.20812	rc5
NM_001099695	REPIN1	0.422027	0.442618	-0.23734	0.203444	-1.21725	0.27794	rc5
NM_181799	UBE2C	0.060893	0.195851	0.39035	-0.09182	-1.21635	-0.96236	rc5
NM_001252152	SLC39A9	0.352846	-0.4675	-0.44897	0.939038	-1.21415	0.11721	rc5
NM_001164816	ACIN1	-0.58068	0.220771	-0.26193	-1.03503	-1.21337	-0.73469	rc5
NM_001006941	ALG3	-0.29066	-0.63985	-0.2091	-0.16733	-1.21176	-0.35763	rc5
NM_001171182	CENPL	0.335494	-0.06943	-0.89664	-0.57787	-1.21101	-0.79003	rc5
NM_002836	PTPRA	-0.69643	0.163879	0.220231	0.999902	-1.20905	-0.23740	rc5
NM_000792	DIO1	0.137515	-0.25398	-0.15298	0.288444	-1.20855	-1.14637	rc5
NM_001161504	ALDH4A1	-0.11409	-0.32085	-0.3802	-0.2628	-1.20726	-1.01652	rc5
NM_182563	C16orf79	-0.63082	-0.12657	-0.84093	-0.64215	-1.20565	-0.73094	rc5
NM_198205	MLX	0.641999	0.209147	-0.58981	0.417367	-1.20227	0.15680	rc5
NM_176071	P2RY2	-1.13951	-0.24765	-0.32384	-0.42798	-1.19917	-0.40095	rc5
NM_001145714	SAE1	1.121166	-0.09336	0.054073	-0.25648	-1.19817	0.42809	rc5
NM_001039465	SRSF5	-0.06659	0.511082	0.176733	-0.09863	-1.19744	-0.73476	rc5
NR_003068	SNORD88B	1.422323	0.312089	0.562162	0.191785	-1.1973	0.54941	rc5
NM_002987	CCL17	0.167631	-0.56667	-0.64816	-0.6089	-1.19681	-0.82866	rc5
NM_178170	NEK8	-0.05041	0.072793	-0.10661	-0.27976	-1.19663	-0.97802	rc5
NM_139048	HLTF	-0.54285	-0.78147	-0.75298	-0.75496	-1.19572	-1.18063	rc5
NM_001007025	GOSR1	-0.06377	-0.0139	-0.17481	-0.1178	-1.1953	-0.54805	rc5
NM_005882	MAEA	0.652658	1.045933	0.766004	1.222309	-1.19236	-0.30188	rc5
NM_148896	NPB	0.094613	0.260286	-0.57712	-1.05212	-1.19146	-1.09333	rc5
NM_012213	MLYCD	-0.08084	-0.29263	-0.8872	-1.05085	-1.18758	-1.13501	rc5
NM_024301	FKRP	0.000459	0.133856	-0.97717	0.134984	-1.18757	0.08522	rc5
NM_014960	ARSG	-0.00123	-0.19126	-0.41605	-0.36604	-1.18717	-1.11292	rc5
NM_001243028	AKT2	-0.50341	-0.85875	-1.08145	-0.93378	-1.18707	-0.98709	rc5
NM_031370	HNRNPD	-0.23509	-0.15439	0.378328	0.217318	-1.18687	-0.92255	rc5
NM_001127203	PCID2	-0.33928	-0.29135	0.1893	0.041894	-1.18544	-0.28809	rc5
NM_001126051	HDGF	-0.43621	-0.87814	-0.73887	-0.68292	-1.18457	-0.61461	rc5
NR_038938	LOC10050576	-0.12913	-0.90973	-0.4095	-0.6482	-1.18332	-1.05657	rc5
NM_058219	EXOSC6	0.117371	0.128568	0.098977	0.058306	-1.18131	-0.94451	rc5
NM_000882	IL12A	0.492907	-0.68343	-0.77788	-0.71183	-1.18072	-1.17777	rc5
NM_173872	CLCN3	-0.27449	0.398144	-1.04718	0.264721	-1.1769	-0.57728	rc5
NM_001048212	CEMP1	-0.20826	-0.04134	-0.45074	-0.75858	-1.16923	-0.87220	rc5
NR_033686	PEF1	-0.03004	-0.08879	-0.27728	-0.10141	-1.16789	-1.01434	rc5

NM_207644	FAM211B	0.191843	-0.07567	-0.61181	-0.81566	-1.16718	-0.97020	rc5
NM_181463	MRPL55	0.175441	-0.40424	-0.38482	0.052658	-1.16714	-0.47753	rc5
NM_006651	CPLX1	-0.26876	-0.43655	-0.47425	-0.58965	-1.16586	-0.57318	rc5
NR_024250	LOC10012555	-0.88338	-0.24116	-1.07999	-0.33584	-1.16586	-0.85765	rc5
NM_001012270	BIRC5	-0.84172	-0.97439	-0.87185	-0.39685	-1.16461	-0.91603	rc5
NM_003534	HIST1H3G	-0.05388	-0.30016	-0.47446	0.375923	-1.16431	-0.04328	rc5
NM_013263	BRD7	-0.31238	-0.36754	-0.36032	-0.33625	-1.16317	-0.93736	rc5
NM_177996	EPB41L1	-0.20103	-0.29731	-0.63494	-0.75064	-1.16085	-0.96932	rc5
NM_199413	PRC1	-0.32739	-0.41704	0.108734	-0.24707	-1.15931	-0.79059	rc5
NR_003654	SCAND2	-0.09304	-0.40286	0.147132	0.066269	-1.15443	-0.59865	rc5
NM_014861	ATP2C2	-0.07396	-0.14888	-0.1481	-0.25539	-1.15114	-1.09088	rc5
NM_024308	DHRS11	-0.01679	-0.14771	0.002602	-0.08851	-1.15101	-1.14421	rc5
NM_001008222	ZDHHC9	0.044561	-0.48777	-0.23528	-0.3925	-1.15093	-0.51700	rc5
NM_014714	IFT140	0.013448	-0.16058	-0.49443	-1.00756	-1.15071	-0.84153	rc5
NM_001009941	ANKRD16	-0.03179	-0.18549	-0.43553	-0.01885	-1.14897	-0.81209	rc5
NM_001083612	AGPHD1	-0.27647	-0.35439	-0.22348	-0.17209	-1.14775	-1.13589	rc5
NM_014362	HIBCH	-0.10454	0.412984	0.066521	0.249324	-1.14683	-0.84163	rc5
NM_001855	COL15A1	0.088255	0.072279	-0.18818	-0.49198	-1.14031	-1.03424	rc5
NM_001174127	SLC11A2	0.302429	0.316106	-0.76045	-0.83596	-1.13918	-0.73184	rc5
NM_001126123	ENOSF1	0.385448	-0.39918	-0.48546	0.019378	-1.13765	-0.31093	rc5
NM_180982	MRPL52	-0.11348	-0.37372	-0.08834	0.115738	-1.13246	0.58171	rc5
NM_032125	TMEM222	-0.01298	-0.2661	0.046282	0.299986	-1.13096	-0.64545	rc5
NM_020702	KIAA1161	-0.0775	-0.19327	-0.44991	-0.8331	-1.1293	-1.08820	rc5
NM_170750	PSMD10	0.14401	0.527358	0.936437	-0.02047	-1.12794	-0.15813	rc5
NM_152531	XXYLT1	-0.12477	-0.32162	-0.63357	-0.68848	-1.12694	-0.92198	rc5
NM_017698	TMEM164	-0.15287	-0.50552	-0.72087	-0.55814	-1.12604	-0.91610	rc5
NM_001131007	KIAA0922	-0.57	-0.10226	-0.3445	0.372501	-1.12379	-0.08092	rc5
NM_031488	L3MBTL2	-0.08473	-0.37926	-0.59061	-0.83222	-1.12308	-0.87832	rc5
NM_006985	NPIP	-0.19295	0.352863	-0.15022	-0.16358	-1.11932	-1.04383	rc5
NM_001042498	SLC35A2	-0.21874	-0.28097	-0.17421	-0.57921	-1.1192	-0.78955	rc5
NM_001134758	ARFRP1	0.353851	0.842871	1.393549	0.38877	-1.11745	0.49577	rc5
NM_032499	C15orf41	-0.78495	-0.47459	-0.37973	-0.02884	-1.11668	-1.00246	rc5
NM_002028	FNTB	0.305104	-0.32055	-0.338	-0.99197	-1.11182	-0.54342	rc5
NM_001080503	CCDC159	0.086019	0.234456	-0.01534	-0.18814	-1.10976	-0.90149	rc5
NM_178448	SAPCD2	-0.05904	-0.10343	-0.4804	-0.84235	-1.10962	-0.75197	rc5
NM_013397	PRICKLE4	-0.23007	-0.25778	-0.72683	-0.84515	-1.10655	-0.85845	rc5
NM_015662	IFT172	-0.05096	-0.1922	-0.27042	-0.60119	-1.10604	-0.89548	rc5
NM_001099684	FAM156B	0.205004	-0.04076	-0.59524	-0.50718	-1.10432	-0.98200	rc5
NR_040252	ANKS3	0.362691	-0.93019	-0.35145	0.32333	-1.10085	-0.35550	rc5
NM_003624	RANBP3	-0.02209	0.04	-0.27122	-0.23217	-1.10035	-0.41298	rc5
NM_145867	LTC4S	0.436041	0.278417	0.1532	0.634885	-1.10024	-1.07576	rc5
NM_001160367	CDK10	0.124842	0.380686	-0.75127	-0.16896	-1.09971	-0.38794	rc5
NM_001128217	PSIP1	-0.17651	0.087867	-0.69637	-0.38048	-1.09958	-0.39128	rc5
NM_175066	DDX51	0.051485	-0.00894	0.082584	0.112974	-1.09859	-0.93120	rc5
NM_001101663	NBPF24	-0.36106	-0.23015	-0.46362	-0.15747	-1.09388	-0.89878	rc5
NM_001128852	SRRT	-0.14854	-0.10808	0.529132	0.017055	-1.09243	-0.89666	rc5
NM_032357	CCDC115	-0.0562	-0.24264	-0.27542	-0.57864	-1.09232	-0.88839	rc5

NM_001114382	TSC2	0.064048	-0.1082	-0.04545	-0.28501	-1.09083	-0.10357	rc5
NM_052813	CARD9	0.101487	0.164949	-0.56915	-0.84707	-1.0903	-0.94233	rc5
NR_038123	PSMA3	-0.36443	0.696048	-0.99839	-0.54692	-1.08583	-0.55127	rc5
NM_004711	SYNGR1	-0.17103	-0.32079	-0.54492	-0.85466	-1.08516	-1.04038	rc5
NM_001244897	PTBP3	-0.41684	0.111175	-0.23617	-0.32512	-1.08315	-0.08710	rc5
NM_015983	UBE2D4	-0.16501	-0.43348	-0.40786	-0.48367	-1.07996	-0.79718	rc5
NR_046176	NBPF3	-0.04527	-0.04098	-0.20866	-0.14556	-1.07699	-0.98574	rc5
NM_016434	RTEL1	-0.09987	-0.07251	-0.30134	-0.67613	-1.07531	-0.80958	rc5
NM_001039374	KIAA1984	0.089986	-0.00616	-0.19843	-0.87762	-1.07485	-0.67364	rc5
NM_001142281	ARCN1	0.470396	0.696029	0.274152	0.472587	-1.0678	-0.15209	rc5
NM_001143944	LEMD2	-0.40984	-0.58796	-0.87258	-0.64753	-1.06692	-1.04412	rc5
NM_080590	CAPS	0.142258	-0.05046	0.096383	-0.10346	-1.06607	-0.11994	rc5
NM_004041	ARRB1	0.154599	0.222355	-1.04037	-0.28518	-1.06533	0.45701	rc5
NM_015570	AUTS2	-0.40552	-0.49333	-0.17342	-0.18435	-1.06335	-0.69025	rc5
NM_015185	ARHGEF9	0.064457	0.100166	-0.18755	-0.61443	-1.06265	-0.83395	rc5
NM_058181	YBEY	0.020939	-0.26708	-0.45293	-0.10031	-1.06197	-0.47486	rc5
NR_024582	JPX	-0.36873	-0.3538	-0.53414	-0.02394	-1.05908	-0.53807	rc5
NM_001198832	PDE4DIP	-0.21146	-0.34548	-0.49576	-0.87519	-1.05802	-1.05237	rc5
NM_025207	FLAD1	-0.75561	-0.94078	-0.95215	-0.35596	-1.05614	-0.93926	rc5
NM_032350	C7orf50	-0.46835	-0.32191	-0.43397	-0.16463	-1.05611	0.13163	rc5
NM_003819	PABPC4	-0.84167	0.218286	0.245776	0.056077	-1.05272	-0.99866	rc5
NM_001135245	TCOF1	-0.282	0.137996	0.218805	0.282354	-1.05152	-0.86249	rc5
NM_001193544	ANXA6	-0.00595	-0.25316	-0.36097	-0.91766	-1.05095	-0.73480	rc5
NR_002174	CMAHP	-0.36196	0.170113	-0.05041	0.148731	-1.04897	-1.04837	rc5
NM_001170543	PGAM5	-0.3769	-0.59343	-0.25441	-0.35502	-1.0473	-0.56248	rc5
NM_001003694	BRPF1	0.052109	0.277191	0.009928	-0.17224	-1.04683	-0.80099	rc5
NM_002650	PI4KA	0.058535	0.132072	-0.15348	0.056806	-1.04382	-0.71364	rc5
NM_001130858	PPIP5K1	0.073808	0.247634	0.355682	-0.05825	-1.04068	-0.52655	rc5
NM_001185181	PFDN6	-1.00358	-0.28801	-0.24802	-0.40058	-1.03974	-0.70306	rc5
NM_018343	RIOK2	-0.75371	-0.31316	-0.3244	0.51377	-1.03918	-0.93638	rc5
NR_003579	FRG1B	-0.32418	-0.38803	-0.59392	-0.11545	-1.03784	-0.90030	rc5
NM_017619	RNPC3	-0.1643	-0.46936	-0.36966	-0.11295	-1.03735	-0.46868	rc5
NR_036263_3	MIR1244-3	0.065065	-0.34269	-0.26297	-0.45573	-1.03638	-0.52270	rc5
NR_024455	LOC10013361	-0.64898	-0.70267	-0.95598	-0.95037	-1.03555	-0.77276	rc5
NM_016286	DCXR	-0.03062	-0.5366	0.293239	0.441318	-1.03482	0.03008	rc5
NM_023078	PYCRL	0.111017	-0.05183	-0.20186	-0.20251	-1.03298	-0.85740	rc5
NM_001145815	AMDHD2	-0.49816	0.597675	-0.30116	-0.37956	-1.0286	-0.63840	rc5
NM_015665	AAAS	0.01629	-0.31757	-0.34368	-0.41055	-1.02532	-0.85448	rc5
NM_001172745	SEC23B	-0.24827	-0.3044	-0.1832	0.428946	-1.02394	-0.42384	rc5
NM_199327	SPRY1	0.024996	-0.64553	-0.23949	-0.1991	-1.02374	-0.74861	rc5
NM_001669	ARSD	-0.1675	-0.37678	-0.28278	-0.30286	-1.02268	-0.75638	rc5
NM_001198934	ABCC10	-0.16641	-0.21045	-0.35543	-0.53974	-1.02074	-0.44674	rc5
NM_001949	E2F3	0.245468	-0.30862	0.430614	0.425363	-1.01877	-0.64417	rc5
NM_002193	INHBB	-0.23641	-0.32118	-0.14779	-0.04025	-1.01727	-0.76442	rc5
NM_030812	ACTL8	-0.18116	-0.55128	-0.38078	-0.49909	-1.01717	-0.95039	rc5
NR_003010	SCARNA12	-0.73613	-0.13946	-0.30382	-0.61901	-1.01225	-0.29940	rc5
NR_033697	NDUFA2	0.484466	-0.18676	0.019501	-0.21347	-1.01225	0.13779	rc5

NM_170739	MRPL11	0.234478	-0.16196	-0.41133	0.086253	-1.00903	-0.62375	rc5
NM_001024628	NRP1	0.09825	-0.27684	-0.19773	-0.54015	-1.00896	-0.83659	rc5
NM_032683	MPV17L2	-0.0135	-0.21004	-0.42011	-0.54385	-1.00799	-0.67598	rc5
NM_199443	USP4	-0.12615	0.037659	0.030315	-0.17289	-1.00626	-0.85775	rc5
NM_199194	BRE	0.366624	-0.35316	0.334846	-0.76029	-1.00597	-0.21864	rc5
NM_152834	TMEM18	0.005934	-0.05851	0.185062	0.397586	-1.00521	-0.99685	rc5
NM_001199266	DGKZ	-0.14994	-0.46692	-0.47415	-0.12981	-1.00436	-0.78977	rc5
NM_001142560	RRNAD1	0.01761	-0.34828	-0.45054	-0.3867	-1.00317	-0.24913	rc5
NM_198508	KLRG2	0.100298	0.062601	-0.20596	-0.63691	-1.00182	-0.88490	rc5
NM_015840	ADAR	-0.07967	-0.42401	0.424335	-0.3603	-2.698	-4.54383	rc6
NM_001015881	TSC22D3	-0.00082	-0.29284	-1.48136	-2.76592	-3.13329	-4.46864	rc6
NM_007008	RTN4	-0.88036	-0.6252	-0.25991	0.934726	-0.94459	-4.26975	rc6
NM_007326	CYB5R3	1.698802	1.100727	0.867363	-3.87714	-2.92646	-4.14664	rc6
NM_178840	C1orf64	0.171295	0.046838	-0.17557	-0.7578	-3.15769	-4.08348	rc6
NM_001003799	TARP	0.089881	-0.17734	-0.2967	-0.23162	-2.72963	-4.04985	rc6
NM_001122898_1	CD99	-0.08076	-0.72324	-1.82703	-1.01691	-2.04489	-3.80690	rc6
NR_033500	PPP2R1A	0.081381	-0.53739	-1.56247	-1.99903	-1.64945	-3.66352	rc6
NR_045604	VTCN1	-0.2313	-0.06158	-0.15619	-0.72024	-2.91252	-3.45216	rc6
NM_173073	SLC35C2	-0.56588	-0.53905	-0.34018	-0.51498	-2.47367	-3.43602	rc6
NM_005949	MT1F	-0.02929	0.025024	-0.58467	-0.68299	-1.74651	-3.34538	rc6
NM_001077511_4	TCF19	-0.59182	-0.31089	-0.79982	-1.69188	-2.61081	-3.32794	rc6
NM_012410	SEZ6L2	-0.22356	-0.27756	-2.16098	-0.0321	-0.78756	-3.28216	rc6
NM_014507	MCAT	-1.07636	-0.48731	-0.60068	-1.99577	-1.97961	-3.24141	rc6
NM_001252075	SLC9A3R2	-0.6423	-0.99925	-0.82856	-0.60533	-1.72813	-3.23874	rc6
NM_001202522_4	DDR1	0.546602	0.514206	0.009146	0.073336	-1.22883	-3.21530	rc6
NM_001099772	CYP4B1	-0.39888	-0.57362	-0.85791	-0.1542	-2.69745	-3.07838	rc6
NM_016448	DTL	0.272237	0.034107	-0.72951	-0.54782	-1.91208	-3.05247	rc6
NM_000221	KHK	-0.08422	-0.39533	-0.53141	-0.5224	-2.65115	-3.03964	rc6
NM_001692	ATP6V1B1	-0.05744	-0.2912	-0.33198	-0.5192	-2.79277	-3.03151	rc6
NM_001948	DUT	-0.17912	-0.51633	0.033498	1.022827	-1.56535	-3.00555	rc6
NM_003905	NAE1	-1.35743	0.162587	-0.17643	-1.00865	-0.93813	-2.98177	rc6
NM_001144994	C2orf72	-0.05079	-0.54584	-1.33221	-1.50706	-2.55139	-2.96453	rc6
NM_024908	WDR76	0.015354	-0.21442	-0.51009	-0.33084	-1.98894	-2.93936	rc6
NM_031912	SYT15	0.006379	-0.27543	-1.04332	-1.40854	-2.49879	-2.91190	rc6
NM_004091	E2F2	-0.03874	-0.78091	-1.19052	-1.40334	-2.23506	-2.91173	rc6
NM_018095	KBTBD4	-1.72568	-1.91779	-2.02289	-0.73769	-0.72052	-2.87274	rc6
NM_001146152	CYP51A1	-0.71385	-0.0286	-0.19875	0.5316	-2.43596	-2.85603	rc6
NM_006198	PCP4	0.243207	0.213464	-0.2525	-0.49682	-1.67105	-2.81781	rc6
NM_001013839	EXOC7	0.224832	-2.01876	-0.86383	-1.64144	-2.34181	-2.81601	rc6
NM_080911	UNG	-0.06779	-0.19245	-0.26074	-0.50414	-2.08633	-2.79977	rc6
NM_007370	RFC5	0.072775	-0.12781	-0.24591	-0.34409	-2.37648	-2.79939	rc6
NM_004966	HNRNPF	-2.22952	-2.0483	-2.76848	-0.22649	-2.2173	-2.79295	rc6
NM_030629	CMIP	-0.51137	-0.59304	-1.55939	-0.28118	-1.62816	-2.79030	rc6
NM_004153	ORC1	-0.23886	-0.62273	-1.37763	-0.91728	-2.07578	-2.78703	rc6
NM_005915	MCM6	0.016091	-0.141	-0.11873	-0.01494	-1.9134	-2.75389	rc6
NM_198993	STAC2	-0.07565	-0.37814	-0.92263	-1.63924	-2.36387	-2.74993	rc6
NR_038900	DSCAM-AS1	0.376402	-0.19216	-0.27208	-0.45249	-2.14301	-2.72978	rc6

NM_002388	MCM3	-0.05344	-0.17335	-0.26937	-0.51213	-1.97085	-2.69255	rc6
NM_133174	APBB3	-0.14091	0.13605	-0.60131	-0.26897	-2.16766	-2.68698	rc6
NM_001254	CDC6	0.103353	0.007155	0.079601	0.191081	-1.71183	-2.61599	rc6
NM_002166	ID2	0.360066	-1.19646	-2.45668	-1.13992	-2.06484	-2.58533	rc6
NM_145638	OSBPL5	-0.61031	-0.75679	-1.59563	-1.36885	-0.57346	-2.57868	rc6
NM_001252151	SLC39A9	-0.97538	0.552238	-0.10922	0.249595	-1.22645	-2.56977	rc6
NM_001195248	APTX	0.072168	0.620962	0.512139	0.680893	-0.03924	-2.50744	rc6
NM_006055	LANCL1	-0.81154	-0.47239	-1.35365	-0.48282	-1.25487	-2.50610	rc6
NM_003633	ENC1	-0.09574	-0.10929	0.083652	-0.45168	-0.32387	-2.49761	rc6
NM_017730	QRICH1	0.306481	0.532929	0.358883	-0.07374	-0.57126	-2.48965	rc6
NM_001184721	GYG1	1.023794	-0.18405	-0.12083	-0.23846	-1.00209	-2.48923	rc6
NM_001204196	SRP19	-0.2737	-1.09615	-0.32845	0.424789	-0.20583	-2.48387	rc6
NM_001142272	RAB11FIP3	-0.71471	-0.63886	-1.14876	-1.47368	-1.70316	-2.47482	rc6
NM_003239	TGFB3	-0.17174	-0.40178	-0.92963	-1.32	-2.3676	-2.47210	rc6
NM_001244567	BID	0.68608	0.026649	-0.09011	0.489345	-0.21201	-2.47178	rc6
NR_038899	DSCAM-AS1	0.065532	-0.29004	-0.40388	-0.46709	-2.35749	-2.45720	rc6
NM_001204447	C20orf29	0.435109	0.022374	-0.39832	0.504144	-0.41038	-2.44873	rc6
NM_182649	PCNA	0.066401	0.043908	0.016847	0.169851	-1.50222	-2.43152	rc6
NM_001789	CDC25A	0.225898	0.240173	0.234778	-0.03321	-2.04168	-2.40835	rc6
NM_080841	PTPRA	-0.053	-0.74339	-0.65573	-1.49492	-1.04918	-2.40665	rc6
NM_001195226	PRODH	0.344433	0.043529	-0.02046	0.169836	-1.52258	-2.38070	rc6
NM_144635	FAM131A	0.076583	-0.53098	-1.24655	-0.87856	-2.09623	-2.37989	rc6
NM_024323	C19orf57	0.288221	0.166415	-0.44607	-1.3345	-1.56436	-2.37578	rc6
NM_001142428	MORF4L2	-0.00536	0.538756	0.07161	1.00201	0.243481	-2.36453	rc6
NM_006275	SRSF6	0.480939	0.885708	0.535657	-0.15859	-2.1931	-2.35820	rc6
NM_002916	RFC4	-0.35002	-0.04458	-0.48548	-1.08783	-1.72982	-2.34307	rc6
NR_002788	CYP4Z2P	-0.08085	-0.13132	-0.24552	-0.24276	-1.51525	-2.33129	rc6
NM_014317	PDSS1	-0.00245	0.012076	0.106201	0.309658	-1.75507	-2.32200	rc6
NM_001178115	ZNF185	-0.2321	-0.76911	-0.63224	-0.63492	-1.3637	-2.30875	rc6
NM_178134	CYP4Z1	0.137984	-0.0867	-0.14066	0.135895	-1.53244	-2.29243	rc6
NM_001146333	SUMF2	-0.22288	-0.19234	-0.69727	-1.20967	-0.88535	-2.29069	rc6
NM_016095	GINS2	-0.0243	-0.3445	-0.16463	-0.02684	-1.68638	-2.28513	rc6
NM_144999	LRRC45	-0.0742	-0.14269	-0.61614	-1.13124	-2.21838	-2.28227	rc6
NM_001142501	MON1A	0.650288	0.029102	0.343605	-0.03434	-1.86961	-2.27854	rc6
NM_170712	RASSF1	0.162972	0.727384	0.217845	-0.8404	-0.87438	-2.26131	rc6
NM_033128	SCIN	0.192952	-0.22493	-0.80357	-0.33167	-2.00944	-2.26080	rc6
NM_024626	VTCN1	0.175832	0.097296	0.083031	0.136191	-1.90197	-2.25676	rc6
NM_024028	PCYOX1L	-0.14759	-0.30517	-0.56252	-1.25701	-1.88331	-2.25582	rc6
NR_037943	STX16	-0.94175	-0.7624	-1.15657	-0.09197	-1.89363	-2.25161	rc6
NM_001199699	DHX33	-0.27696	-0.8309	-0.4434	0.890737	-0.98563	-2.23663	rc6
NM_001143966	TBC1D7	-0.1088	-0.06547	-1.50214	-1.31459	-1.31968	-2.23077	rc6
NM_175748	UBR7	-0.223	-0.27928	-0.14441	-0.25014	-1.58931	-2.22617	rc6
NM_012177	FBXO5	-0.71982	-0.40978	-0.06853	0.268669	-1.03292	-2.22461	rc6
NM_001037553	AGPAT3	0.075068	-0.20398	-0.91486	-0.64087	-0.85842	-2.22090	rc6
NM_178517	PIGW	0.023761	0.023511	0.016465	0.443153	-1.6399	-2.21651	rc6
NM_001077447	PPCS	-0.24848	-0.22811	-0.35567	-0.00619	-1.52287	-2.21203	rc6
NM_012307	EPB41L3	-0.08259	-0.17311	-0.22118	-0.53863	-1.3198	-2.19948	rc6

NM_181464	MRPL55	-0.33793	-0.2441	0.00967	-0.0919	-1.5899	-2.19279	rc6
NM_001003395	TPD52L1	0.386663	-0.17873	-1.23072	0.087399	-1.42373	-2.18377	rc6
NM_001350_4	DAXX	-0.10175	-0.1261	-0.73406	-0.0294	0.333898	-2.18372	rc6
NM_001198557	LMNB1	-0.36034	-0.2193	-0.50701	-0.29219	-1.18805	-2.18106	rc6
NM_001170637	SRGAP2	-0.2444	-0.86853	-2.0155	-1.82313	-1.29282	-2.16706	rc6
NR_038150	UBR7	0.784225	0.196775	-0.06236	0.405615	-1.27613	-2.16336	rc6
NM_145167	PIGM	-0.14126	-0.31989	-0.78656	-0.52442	-1.63284	-2.15637	rc6
NM_024900	PHF17	0.043032	-0.54409	-1.23203	-0.94553	-1.47804	-2.14990	rc6
NM_017975	ZWILCH	-0.32669	0.087276	-0.13769	0.067123	-1.6603	-2.14273	rc6
NM_001007270	GEMIN7	0.17275	-0.07207	-0.00797	0.334257	-0.27091	-2.13854	rc6
NR_045493	LSM1	-0.31588	-0.05936	-0.84456	-0.16361	-0.81504	-2.13391	rc6
NM_001018001	KAZN	-1.48803	-0.81471	-0.97674	-0.06023	-0.91676	-2.11323	rc6
NM_024642	GALNT12	-0.22105	0.072482	-0.13562	-0.21389	-1.68541	-2.11057	rc6
NM_001024937	MINK1	-1.19191	0.022121	0.080292	-1.64931	0.001205	-2.10698	rc6
NM_014573	TMEM97	-0.1216	-0.2671	-0.18262	-0.11703	-1.57196	-2.09914	rc6
NM_178190	ATPIF1	0.216652	-0.27263	-1.16021	0.114813	-2.08762	-2.09646	rc6
NM_004111	FEN1	-0.00102	-0.09984	-0.05262	-0.21275	-1.57535	-2.09405	rc6
NM_003504	CDC45	-0.08264	0.003754	-0.0667	-0.30769	-1.79477	-2.09404	rc6
NM_001002810	PDE4DIP	-0.08846	-0.11923	0.366564	0.183114	-1.22796	-2.08606	rc6
NM_006859	LIAS	0.005967	-0.20424	-1.29516	-0.43571	-1.55465	-2.07257	rc6
NM_199192	BRE	-0.45487	-0.26411	0.142788	-0.34024	0.213821	-2.05898	rc6
NM_033493	CDK11B	-0.88559	0.734409	0.10119	-1.47139	0.157172	-2.05209	rc6
NM_018571	STRADB	-0.06223	-0.18692	-0.15225	-0.11686	-0.68138	-2.05093	rc6
NM_007238	PXMP4	-0.13566	-0.59637	-0.92845	-1.02386	-1.75094	-2.04533	rc6
NM_017615	NSMCE4A	0.017543	-0.0146	0.011217	-0.17885	-1.11874	-2.04339	rc6
NM_001037333	CYFIP2	-0.0316	-0.22874	-0.34373	-0.65251	-1.41556	-2.04066	rc6
NM_002946	RPA2	-0.00996	-0.24302	-0.49668	-0.55097	-1.57369	-2.03973	rc6
NM_001955	EDN1	3.220127	1.91671	1.482596	1.254213	0.578087	-2.03529	rc6
NM_016240	SCARA3	-0.24483	-0.38611	-0.47061	-0.9786	-1.97362	-2.02526	rc6
NM_001253902	MEST	-0.51634	-0.56831	-0.69076	-0.52356	-1.05421	-2.01935	rc6
NM_000791	DHFR	0.049875	-0.18423	-0.02193	0.043069	-1.30882	-2.01352	rc6
NM_001134477	PARN	-0.42953	-0.54596	-0.55323	0.453268	-0.68164	-2.01326	rc6
NM_012392	PEF1	-0.32898	0.396857	0.483268	0.359222	-0.0375	-2.01151	rc6
NM_017709	FAM46C	-0.01608	-0.70267	-0.19791	0.059042	-1.57249	-2.00754	rc6
NM_005225	E2F1	0.009651	-0.19674	-0.481	-0.76708	-1.76942	-2.00215	rc6
NM_001100118	XRCC3	0.346926	0.110067	-0.93331	-1.31333	-0.68198	-2.00004	rc6
NM_001251990	GMNN	-0.04687	-0.0186	0.064664	0.211877	-0.87402	-1.99897	rc6
NM_130384	ATRIP	0.020011	-0.15162	-1.30818	-0.68071	-1.56398	-1.99796	rc6
NM_000234	LIG1	-0.03346	-0.18101	-0.17609	-0.57589	-1.69763	-1.99573	rc6
NM_020647	JPH1	-0.11254	-0.24102	-0.64866	-0.19741	-1.41827	-1.99554	rc6
NM_006739	MCM5	0.05224	-0.1904	-0.43447	-0.59905	-1.71706	-1.98982	rc6
NM_206967	C16orf74	0.041647	-0.12821	-0.38188	-1.00228	-1.92306	-1.98558	rc6
NR_026737	C6orf52	-0.48742	0.070987	-0.18594	0.159597	-1.32409	-1.98544	rc6
NM_001129995	KCTD15	0.67301	0.797697	-0.4836	-1.78946	-0.41068	-1.97582	rc6
NM_002823	PTMA	0.108999	0.622617	-0.7078	1.075284	-0.95856	-1.97394	rc6
NM_000535	PMS2	-0.03394	-0.16991	-0.56799	-0.27045	-0.84944	-1.96388	rc6
NM_130791	WWOX	-0.02863	-0.60864	-0.08379	-0.07186	-1.06484	-1.96163	rc6

NM_001161009	ARMC10	0.288025	-1.13597	-0.91107	0.103905	-0.6923	-1.95833	rc6
NR_024568	FBXO31	-0.45964	-1.51036	-0.30802	-0.88412	-0.53278	-1.95670	rc6
NM_015190	DNAJC9	0.016552	0.218762	0.342832	0.40255	-1.5105	-1.94837	rc6
NM_001195001	PTPRU	-0.11961	-0.73487	-1.21606	-1.16643	-1.24564	-1.94643	rc6
NM_153022	C12orf59	-0.2892	-0.58377	-0.79352	-1.43252	-1.79505	-1.94583	rc6
NM_199050	C2CD2	-0.47718	-0.19594	-0.97739	-0.67333	-0.7356	-1.93739	rc6
NM_198505	ATP13A5	0.060011	-0.15761	0.064851	0.105619	-1.6327	-1.93173	rc6
NM_000320	QDPR	-0.0753	-0.2556	-0.12999	-0.20845	-1.5804	-1.92973	rc6
NM_052903	TUBGCP5	-0.05694	-0.38602	-0.81416	-0.58272	-1.41474	-1.92746	rc6
NM_001172222	SCMH1	-0.45612	-0.26797	-0.79514	-0.93637	-0.6244	-1.91995	rc6
NM_007083	NUDT6	-0.11051	0.234298	-0.43135	-0.23088	-1.85056	-1.91978	rc6
NR_037716	RNASEK	0.138321	-1.16491	-1.72581	-0.74039	-0.23828	-1.91842	rc6
NM_001142604	PPT1	-0.07902	-0.12377	-0.39494	-0.51454	-1.87795	-1.91665	rc6
NM_178836	PLD6	0.151477	-0.66362	-0.44844	-0.46798	-1.37703	-1.91273	rc6
NM_145016	GLYATL2	-0.07813	0.411185	0.028321	-0.28448	-0.68403	-1.91122	rc6
NM_004366	CLCN2	0.420297	0.009294	0.058202	-1.4664	-0.36114	-1.90936	rc6
NM_001134479	LRRC8D	-0.06086	-0.20359	-0.16322	0.523028	-0.78421	-1.90672	rc6
NM_001025107	ADAR	-0.72609	-0.81082	-0.23778	-0.78666	-1.6331	-1.90434	rc6
NM_001034	RRM2	0.113568	0.044621	0.345948	0.405753	-1.00647	-1.90020	rc6
NM_145913	SLC5A8	-0.13791	-0.00453	-0.29745	-0.15323	-1.12214	-1.89230	rc6
NR_000027	SNORD83A	-0.41136	0.061782	0.028045	0.035897	-1.61075	-1.89028	rc6
NM_025133	FBXO11	-0.09905	-0.17336	-0.40842	-0.25499	-1.54234	-1.88743	rc6
NM_014703	VPRBP	-0.0362	0.38762	0.40679	0.282187	-0.80825	-1.88694	rc6
NM_172206	CAMKK1	-0.17393	-0.59143	-0.38609	-0.50715	-1.38468	-1.88464	rc6
NR_037182	LOC653513	0.041911	-0.14035	-0.31857	-0.26097	-1.39941	-1.88393	rc6
NM_058179	PSAT1	0.018727	-0.39791	-0.24218	0.352106	-1.07966	-1.88191	rc6
NM_001122634	CPS1	-0.65427	0.102123	-0.37398	-1.0034	-1.04423	-1.87804	rc6
NR_033248	LOC10032910	0.020505	-0.27647	-0.55916	-0.25955	-1.78346	-1.87642	rc6
NM_148907	OSBPL9	-0.25323	0.155691	-0.2492	-1.47168	-0.58828	-1.87624	rc6
NM_001085354	MORC4	0.201722	-0.86641	-1.83285	-0.44895	-1.23471	-1.87091	rc6
NM_198901	SRI	0.387569	0.091244	-0.33383	0.478731	-0.16581	-1.87049	rc6
NM_001136198	FZR1	-0.88729	-0.3626	-0.59321	-0.5346	-0.65275	-1.86979	rc6
NR_027763	GYS1	-0.35002	0.191232	0.121021	0.730613	0.222161	-1.86927	rc6
NM_001145355	CBWD1	0.189674	0.217826	0.168003	0.673708	0.056272	-1.86583	rc6
NM_203351	MAP3K3	-0.34873	-0.54965	-0.23269	-1.27003	-1.26366	-1.86139	rc6
NM_153338	GGT6	0.075004	0.139338	-1.21316	-0.72146	-1.36427	-1.85849	rc6
NM_001038603	MARVELD2	-0.03769	-0.36911	-0.52391	-0.23377	-0.28602	-1.85600	rc6
NM_007109_2	TCF19	0.402898	-0.10713	0.07582	0.078667	-1.13795	-1.85513	rc6
NM_002915	RFC3	-0.3425	-0.13576	-0.14595	0.149025	-1.07767	-1.85388	rc6
NM_001040452	RUFY1	-0.07857	-0.30106	-0.92384	0.205659	-1.336	-1.85092	rc6
NM_013302	EEF2K	-0.30979	-0.52267	-1.03275	-0.84628	-1.50469	-1.85023	rc6
NM_005983	SKP2	-0.16208	-0.75927	-0.63847	0.04204	-1.00979	-1.84534	rc6
NM_001122890	GGT6	-0.35194	-1.28514	0.012854	-1.18303	-1.77392	-1.84466	rc6
NM_002533	NVL	0.403583	-0.47435	-0.83172	0.229343	-1.18835	-1.84304	rc6
NM_001161452	CYBASC3	-0.11256	-0.58101	-0.69412	-0.4259	-1.46407	-1.84270	rc6
NM_001256692	MELK	0.02205	0.44508	-1.75311	0.191671	-0.15499	-1.83628	rc6
NM_004526	MCM2	0.007008	-0.33161	-0.38326	-0.66544	-1.57881	-1.83392	rc6

NM_003038	SLC1A4	-0.0624	-0.3505	-0.19894	-0.39347	-1.62506	-1.83343	rc6
NM_000922	PDE3B	-0.19045	0.12561	-0.11952	-0.24591	-1.82174	-1.83335	rc6
NM_001017416	USP1	-0.69401	0.436335	-0.56545	0.085058	-1.46714	-1.83002	rc6
NM_007357	COG2	0.132829	-0.13161	-0.16903	-0.16442	-1.57317	-1.82494	rc6
NM_001040260	DCLK2	0.188297	0.314943	0.013891	-1.0817	-0.9728	-1.81811	rc6
NM_178858	SFXN2	0.005434	-0.32104	-0.42405	-0.28056	-1.52298	-1.81714	rc6
NR_029710	MIR193A	-1.57072	0.238333	0.076799	0.899313	-1.28098	-1.81466	rc6
NR_046293	MSTO1	0.581063	0.503818	-1.44143	-0.36777	-1.68082	-1.81044	rc6
NM_024094	DSCC1	-0.22935	-0.15491	-0.18238	0.066106	-0.96356	-1.80728	rc6
NM_002689	POLA2	-0.13342	-0.26195	-0.57432	-0.7492	-1.2132	-1.80383	rc6
NM_000179	MSH6	0.163141	0.043213	-0.13266	-0.08312	-1.1328	-1.80135	rc6
NM_001142650	HNRPLL	-0.33634	0.197819	0.368783	0.886961	-0.34799	-1.79933	rc6
NR_002588	SNORA4	0.044748	0.774284	-0.0159	-0.5051	-0.87145	-1.79686	rc6
NM_001076680	C17orf108	0.116112	-0.25146	-0.53456	-0.31546	-1.79081	-1.79548	rc6
NR_027700	NOP56	0.085686	0.143495	0.364248	0.359112	-1.63473	-1.78825	rc6
NM_213620	ATP6V1H	0.025853	-0.40757	-0.51661	0.219123	-1.16833	-1.78810	rc6
NR_028105	DCAF8	0.280399	0.227804	-0.71038	-0.06824	-1.41786	-1.78719	rc6
NM_033001	GTF2I	0.414345	-0.16749	0.154252	0.029791	-1.2187	-1.78342	rc6
NM_153282	HYAL1	-0.81621	-0.47709	0.30816	0.411483	-0.64643	-1.77965	rc6
NM_004242	HMGN3	-0.97636	-1.22653	-1.01211	-1.66153	-1.25611	-1.77881	rc6
NR_026984	LOC90784	-0.26673	-0.23027	-0.48275	-0.40409	-1.69888	-1.77585	rc6
NM_144627	C1orf182	0.094745	-0.52739	-0.19672	-0.21228	-1.36935	-1.77583	rc6
NM_005483	CHAF1A	-0.0321	-0.09867	-0.57846	-0.61767	-1.26505	-1.77375	rc6
NM_021067	GINS1	0.162539	-0.17323	-0.33087	-0.26517	-0.9643	-1.76687	rc6
NM_004694	SLC16A6	-0.32719	-0.23689	-0.39175	-0.3832	-1.0405	-1.76156	rc6
NM_001172437	PEG10	-0.48788	-0.22133	0.220737	-0.58649	-0.97845	-1.75889	rc6
NM_152329	LRR1	-0.46254	-0.18594	-0.15202	-0.25097	-1.3095	-1.75817	rc6
NM_194436	LDHD	-0.41491	-0.14107	-0.0469	-0.2459	-0.81913	-1.75780	rc6
NM_016467	ORMDL1	0.205943	0.297676	0.049309	0.810136	-1.08269	-1.75486	rc6
NM_033259	CAMK2N2	-0.04446	-0.39307	-0.38909	-0.68431	-1.30241	-1.74683	rc6
NM_002073	GNAZ	-0.29453	-0.17967	-0.62351	-0.60767	-1.51564	-1.74410	rc6
NM_014666	CLINT1	-0.23688	-0.32255	0.293853	0.714239	-0.21185	-1.74277	rc6
NM_001160708	POLR3B	0.397762	0.103337	0.115245	0.345728	-0.83478	-1.74266	rc6
NM_001197220	PDE4D	0.253297	0.811265	-0.16366	-0.42134	-1.24084	-1.73831	rc6
NM_003846	PEX11B	0.213527	-0.79347	-0.52624	-0.12379	-0.74482	-1.73703	rc6
NM_014324	AMACR	-0.01821	-0.18606	-0.30124	-0.12375	-0.80826	-1.73642	rc6
NM_024629	MLF1IP	-0.29518	-0.0398	-0.05797	-0.04594	-1.14688	-1.73525	rc6
NM_000623	BDKRB2	0.664174	1.282706	-0.01477	-0.61616	-0.86494	-1.73352	rc6
NM_005175	ATP5G1	-1.51539	-0.53963	-0.75771	0.816207	-1.49837	-1.73262	rc6
NM_032775	KLHL22	-0.05127	-0.67005	-1.0099	-1.17253	-0.50882	-1.72792	rc6
NM_017940	NBPF1	-0.10943	-0.20649	-0.36622	-0.47339	-1.37379	-1.72743	rc6
NM_003017	SRSF3	0.293831	0.371593	0.395212	0.477074	-0.94435	-1.72725	rc6
NM_003505	FZD1	0.003121	-0.22957	-0.47467	-0.56894	-1.16532	-1.72713	rc6
NM_207584	IFNAR2	0.630393	-0.34764	0.494425	0.672292	0.860794	-1.72275	rc6
NM_001033572	AMZ2	-0.25045	-0.35441	-0.44042	-0.30639	-0.53645	-1.72246	rc6
NM_000788	DCK	-0.16238	-0.17871	-0.09562	0.067075	-0.86178	-1.72157	rc6
NM_001012456	SEC61G	-0.16877	-0.10185	-0.72445	-0.68532	-0.4982	-1.72062	rc6

NR_033142	TMEM48	-1.17109	-0.50789	-0.36209	0.015121	-1.54884	-1.71886	rc6
NM_014321	ORC6	-0.04019	0.040391	-0.02371	-0.15935	-1.40342	-1.71383	rc6
NM_181471	RFC2	0.016432	-0.21678	-0.12664	-0.14216	-1.41897	-1.71347	rc6
NM_203386	RNH1	0.17803	-0.15259	-0.36235	-0.20568	-1.59912	-1.70967	rc6
NM_012288	TRAM2	0.014211	-0.21408	-0.08982	0.295734	-1.38571	-1.70869	rc6
NM_001018115	FANCD2	0.130781	-0.13324	-0.33112	-0.15087	-1.00666	-1.70324	rc6
NR_015411	LOC254559	-0.0912	-0.24059	-0.9107	-0.84184	-1.09419	-1.70094	rc6
NM_001102368	C14orf159	0.048031	-0.05315	-0.155	-0.41443	-1.3415	-1.69882	rc6
NM_004953	EIF4G1	-0.25468	0.117489	-0.25814	0.420294	-0.9981	-1.69747	rc6
NM_207661	ZC3H14	-0.20385	0.035632	-0.00657	0.458	-1.61406	-1.69687	rc6
NM_001100426	RAP1GDS1	-0.67264	-0.07687	-0.85228	-0.68968	-1.09338	-1.69616	rc6
NR_023923	C14orf167	-0.34686	-0.48618	-1.24552	-0.25731	-0.79286	-1.69537	rc6
NM_203379	ACSL5	-0.01106	0.246695	-0.06621	0.608088	-0.19963	-1.69274	rc6
NM_017990	PDPR	-0.35723	-0.55041	-0.81022	-0.78425	-1.47964	-1.69132	rc6
NM_001144956	RIIAD1	0.141363	0.205312	0.275847	-0.19852	-0.80791	-1.69045	rc6
NM_001080450	BEND3	-0.07443	0.459208	1.262347	1.220423	-1.20587	-1.68682	rc6
NM_052928	SMYD4	0.076584	-0.53452	-0.96912	-0.73989	-1.25646	-1.68596	rc6
NM_001078175	SLC29A1	1.184311	0.958223	0.386056	0.90653	-0.50797	-1.68572	rc6
NM_014817	TRIL	-0.34483	-0.70829	-1.29559	-1.09418	-1.48596	-1.68434	rc6
NM_152298	NASP	0.178278	-0.03513	-0.3629	-0.39217	-1.45497	-1.67775	rc6
NM_015261	NCAPD3	0.084413	-0.08527	-0.14913	-0.34693	-1.08776	-1.67146	rc6
NM_183058	LYZL2	-0.36953	-0.50732	-0.38617	-0.28347	-1.50496	-1.67062	rc6
NM_016937	POLA1	-0.14858	-0.05524	-0.24516	-0.08004	-0.8131	-1.66845	rc6
NM_001142422	MORF4L2	0.180448	0.290986	-0.12198	0.855695	0.223501	-1.66839	rc6
NM_000189	HK2	0.120793	0.283854	0.253359	0.285771	-1.28036	-1.66731	rc6
NM_017522	LRP8	-0.75724	-0.20396	-0.81569	-0.57322	-0.95024	-1.66712	rc6
NM_001164501	EIF4ENIF1	-0.10782	-0.50674	-0.45045	-0.68492	-0.61156	-1.66685	rc6
NM_001031672	CYB5RL	-0.03087	-0.16209	-0.46827	-0.67552	-1.2195	-1.66217	rc6
NM_032336	GINS4	0.042514	-0.10844	0.116217	0.120132	-1.28665	-1.65868	rc6
NM_001159770	SLC39A11	-0.63905	-0.67668	-0.79146	0.258231	-0.71802	-1.65494	rc6
NM_182776	MCM7	-0.44323	-0.24866	-0.50171	-0.32461	-1.24641	-1.65271	rc6
NM_001076683	UBTF	-0.32171	-0.50335	-0.71256	-0.61014	-1.49181	-1.65100	rc6
NM_001164759	PRKAR1B	1.000477	0.643294	0.56856	-0.44472	-0.2723	-1.64455	rc6
NR_002605	DLEU1	-0.14379	-0.37409	-0.30179	-0.21675	-0.92336	-1.64056	rc6
NM_020685	C3orf14	0.033719	-0.14453	-0.12861	0.086699	-1.04004	-1.63939	rc6
NM_018129	PNPO	-0.01912	-0.39952	-0.50528	-0.60031	-1.60904	-1.63464	rc6
NR_046216	MGC27345	-0.13054	0.022909	-0.48086	0.008067	-1.58363	-1.63409	rc6
NM_024051	GGCT	-0.18999	-0.03769	-0.28378	-0.1059	-1.01907	-1.63045	rc6
NM_021965	PGM5	-0.08738	-0.06679	-0.05839	-0.44282	-1.19688	-1.62991	rc6
NR_003065_3	SNORD84	-1.27818	-0.14781	-1.21828	0.084612	-0.99111	-1.62949	rc6
NM_213645	WARS	0.074397	0.2397	0.00445	-0.95077	-1.30318	-1.62556	rc6
NM_004237	TRIP13	-0.05177	-0.08242	0.006892	0.113498	-1.02376	-1.62462	rc6
NM_181503	EXOSC8	-0.13554	-0.22157	-0.03016	-0.0033	-1.2319	-1.62254	rc6
NM_182746	MCM4	0.004719	0.039534	0.150877	0.230601	-1.09748	-1.62054	rc6
NR_034137	LOC729013	-0.17408	-0.55872	-0.35408	-0.06288	-1.25366	-1.61770	rc6
NM_001142784	IL11RA	-0.32792	-0.47763	-0.17082	-0.4734	-1.52815	-1.61276	rc6
NM_020689	SLC24A3	-0.01738	-0.12757	-0.40977	-0.56453	-1.34698	-1.61211	rc6

NM_182486	C1QTNF6	-0.19562	-0.76582	0.041392	-0.24752	-0.34403	-1.60757	rc6
NM_001135654	PABPC4	0.807711	-0.06052	0.58235	0.242507	-0.276	-1.59275	rc6
NM_001256512	SSBP1	-0.39762	-0.38168	0.265279	0.786824	-0.2786	-1.59209	rc6
NM_017951	SMPD4	-0.28993	-0.57368	-0.45405	-0.10194	-1.46835	-1.58837	rc6
NR_015376	LINC00200	-0.13778	-0.42443	-0.08638	-0.7395	-1.31447	-1.58825	rc6
NM_017512	ENOSF1	-0.42653	-0.35647	-0.0625	0.392295	-0.49851	-1.58645	rc6
NM_001139518	PRKRA	-1.10006	-0.32251	-0.56727	-0.69703	-1.35188	-1.58338	rc6
NM_001198840	RBM12	0.926946	-0.14389	0.553643	0.858846	-0.17617	-1.57968	rc6
NM_000251	MSH2	-0.41883	-0.03655	-0.15196	-0.02072	-0.81114	-1.57939	rc6
NM_001256678	KIAA0391	-1.28539	-1.06858	-0.6246	-0.43576	-0.06795	-1.57674	rc6
NM_001024594	C1orf53	0.483614	0.108383	0.0888	0.177388	-0.70575	-1.57515	rc6
NM_183323	PAIP1	0.084808	-0.22894	-0.43459	1.184445	-0.99226	-1.57327	rc6
NM_003720	PSMG1	0.130479	0.104576	0.238984	0.426761	-1.37215	-1.57312	rc6
NR_036638	FAM213B	0.235859	-0.30162	-0.14014	-0.13007	-1.05105	-1.57162	rc6
NM_175886	PRPS1L1	-0.00067	-0.1586	-0.11985	-0.27832	-0.78787	-1.56879	rc6
NM_014805	EPM2AIP1	-0.51453	-0.12489	-0.28427	-0.2003	-1.29506	-1.56564	rc6
NM_001134673	NFIA	-0.37854	-0.24508	-1.2211	-0.66938	-1.55185	-1.56318	rc6
NM_152858	WTAP	-0.41695	-0.70056	0.703488	1.466318	-0.82788	-1.55918	rc6
NR_027016	PGAP2	-2.6E-05	0.052602	-0.71214	-0.39169	-1.39398	-1.55764	rc6
NM_001137664	ANAPC7	-0.2509	-0.09281	-0.08167	0.008673	-1.54687	-1.55709	rc6
NM_006329	FBLN5	-0.12772	-0.25236	-0.52764	-0.6994	-1.02855	-1.55603	rc6
NM_001199984	NDUFS1	0.703653	0.449314	0.151722	0.595137	-0.6069	-1.55501	rc6
NM_052845	MMAB	0.050049	-0.16735	-0.23789	0.097019	-1.17924	-1.55336	rc6
NM_006925	SRSF5	0.37955	0.999369	0.899594	0.038918	-1.03277	-1.55075	rc6
NM_018273	TMEM143	-0.21317	-0.24022	-0.72195	-0.76619	-1.25213	-1.54917	rc6
NM_006796	AFG3L2	-0.03982	0.002409	0.024169	0.172199	-1.23066	-1.54740	rc6
NM_032490	C14orf142	0.12367	0.002004	0.20162	0.068142	-1.09969	-1.53885	rc6
NM_001077440	BCLAF1	-0.81602	-0.25509	0.114054	-0.3092	-1.37758	-1.53746	rc6
NM_002875	RAD51	-0.07002	-0.32103	-0.24271	-0.1333	-0.92621	-1.53600	rc6
NM_014967	FAN1	-0.07483	-0.07385	-0.61196	-0.4807	-1.31192	-1.53581	rc6
NR_034085	LOC648987	-0.10005	-0.45903	-1.01755	-1.09422	-1.36417	-1.53563	rc6
NM_000380	XPA	0.466663	0.326095	0.448185	0.21334	-1.22418	-1.53513	rc6
NR_036682	BCL7B	0.206729	-0.42569	-0.5703	-0.03815	-1.01357	-1.53367	rc6
NM_001167618	MLH1	0.417851	-0.02675	0.065752	-0.27853	-0.96871	-1.52895	rc6
NM_017790	RGS3	-0.28511	-1.52243	-1.03818	-1.40308	-0.78792	-1.52704	rc6
NM_033544	RCCD1	-1.21895	0.420972	-0.09914	-1.45587	-0.86675	-1.52618	rc6
NM_018706	DHTKD1	-0.06029	-0.18724	-0.22508	-0.32846	-1.0509	-1.52384	rc6
NM_006953	UPK3A	0.206355	-0.20521	-0.35021	0.4202	-1.31014	-1.52202	rc6
NM_001145317	DSN1	-0.29916	-0.73577	0.321593	-1.42075	-0.85496	-1.51856	rc6
NM_001030010	ALDH3B1	-0.14483	-0.4454	-0.30114	-0.76349	-1.39584	-1.51344	rc6
NM_001243728	ATPAF1	-0.26241	-0.92428	-0.99472	-0.52074	-0.96484	-1.50332	rc6
NM_199414	PRC1	-0.04322	0.204175	-0.46896	-0.41284	-0.4278	-1.50050	rc6
NM_152932	GLT8D1	0.036401	-0.30849	-0.65684	-0.17547	-0.74613	-1.50041	rc6
NM_138381	OXNAD1	0.072647	0.186763	0.032836	-0.06765	-1.39424	-1.50038	rc6
NM_005932	MIPEP	-0.08702	-0.30169	-0.23225	-0.18186	-1.30762	-1.49791	rc6
NM_001142605	EFTUD2	0.140918	0.634674	0.059309	-0.43258	-0.12943	-1.49716	rc6
NM_001193369	DIDO1	0.157625	-0.28808	-0.41636	-0.18805	-0.91198	-1.49693	rc6

NR_003614	PMS2L2	0.211289	-0.17463	-0.29881	0.214049	-1.19053	-1.49483	rc6
NM_005687	FARSB	0.140816	-0.15218	0.058873	0.341253	-0.99729	-1.49418	rc6
NM_144574	WDR20	0.204821	-0.25086	-0.33389	0.483577	-0.39726	-1.49214	rc6
NM_015954	DERA	0.130656	0.143221	0.123453	0.493094	-0.61659	-1.48947	rc6
NM_033276	XRCC6BP1	-0.21092	-0.22998	-0.34165	-0.0915	-1.36148	-1.48946	rc6
NM_001100170	IMMT	0.0589	0.151636	-0.05867	-0.06191	-0.87303	-1.48716	rc6
NM_005206	CRK	-0.087	-0.14625	-0.20186	-0.45907	-0.33476	-1.48468	rc6
NM_006025	ENDOU	0.085634	0.03637	-0.0103	-0.10121	-0.4723	-1.48338	rc6
NM_018048	MAGOHB	-0.05969	-0.17801	-0.05081	0.040365	-1.32183	-1.48328	rc6
NM_001146218	WRB	0.457722	0.020642	-0.50094	0.478649	-0.3392	-1.48286	rc6
NM_013235	DROSHA	-0.33671	-0.44694	-0.72049	-0.26524	-0.66896	-1.48218	rc6
NM_001039716	DIO1	-0.23366	0.045469	-0.02332	-1.05001	-0.71492	-1.47971	rc6
NM_017443	POLE3	-0.08989	-0.29811	-0.12616	-0.10455	-1.17532	-1.47509	rc6
NM_002945	RPA1	0.097133	-0.13183	-0.07127	-0.25667	-0.803	-1.47421	rc6
NM_025077	TOE1	-0.09934	-0.0948	-0.02205	-0.10749	-1.33964	-1.47351	rc6
NM_005956	MTHFD1	0.122297	-0.08866	-0.0307	-0.02975	-1.01093	-1.47335	rc6
NM_001166425	GNAI2	-0.16475	0.367298	-1.27619	-1.43151	-1.40659	-1.46974	rc6
NM_020236	MRPL1	0.148002	0.269826	0.04301	-0.10345	-0.91407	-1.46949	rc6
NM_015036	ENDOD1	-0.14164	-0.11706	-0.05261	0.445984	-0.96451	-1.46752	rc6
NM_020165	RAD18	-0.19462	0.222034	-0.12738	-0.02541	-1.01284	-1.46404	rc6
NR_024111	SBDSP1	0.252086	0.430148	0.227538	0.340723	-0.73155	-1.45886	rc6
NM_001184991	NDUFC1	0.367732	-0.53947	0.047069	-0.88389	-0.21161	-1.45601	rc6
NM_001134423	CDV3	-0.14631	0.300382	0.070574	1.121829	-1.0418	-1.45516	rc6
NM_003384	VRK1	-0.20436	-0.0059	-0.22366	0.118649	-0.79379	-1.45433	rc6
NM_001126121	SLC25A19	-0.37293	-0.43751	-0.42416	0.628566	-1.42613	-1.45430	rc6
NM_001137559	ANAPC5	-0.06245	0.059346	0.005217	-0.0119	-0.88097	-1.45370	rc6
NM_006638	RPP40	-0.0713	0.186775	0.190247	0.742063	-0.91524	-1.45317	rc6
NM_014353	RAB26	-0.07658	-0.2488	-0.40382	-0.66942	-1.18919	-1.45131	rc6
NM_017878	HRASLS2	0.341427	-0.39999	-0.39985	-0.63786	-1.32554	-1.45117	rc6
NM_003920	TIMELESS	-0.08565	-0.21994	-0.23303	-0.46846	-1.1085	-1.45032	rc6
NR_033242	C19orf79	-0.11432	-1.16222	-0.49075	0.473269	-0.58545	-1.44957	rc6
NM_001252129	LSM4	0.203818	0.326655	-0.75257	-0.78186	-0.56248	-1.44539	rc6
NM_000196	HSD11B2	-0.03267	-0.31961	-0.37522	-0.29902	-1.32137	-1.44505	rc6
NM_177560	CSNK2A1	0.339963	0.551865	0.331834	0.554142	-0.65802	-1.44497	rc6
NR_026920	MORC2-AS1	0.110253	0.455126	-0.11116	0.133968	-1.26829	-1.44314	rc6
NM_031243	HNRNPA2B1	0.18242	0.336698	0.356837	0.4698	-0.57062	-1.44267	rc6
NM_021203	SRPRB	-0.01925	-0.17682	0.060689	0.143713	-1.28651	-1.44168	rc6
NM_001001556	GALK2	-0.4411	-0.75414	-1.32599	-0.36216	-1.34873	-1.44099	rc6
NM_024766	CAMKMT	-0.14075	-0.36037	-0.29729	-0.15605	-1.1172	-1.44053	rc6
NM_153712	TTL	0.002434	-0.06276	0.000511	0.046717	-0.73201	-1.43975	rc6
NM_006479	RAD51AP1	-0.23497	-1.06696	-1.323	-0.20199	-1.11256	-1.43931	rc6
NM_030771	CCDC34	0.31227	-0.3683	-0.41579	0.076685	-1.1462	-1.43758	rc6
NM_001170536	ATAD3A	-0.02824	-0.06511	-0.32008	-0.32911	-1.03234	-1.43742	rc6
NM_032646	TTYH2	0.007651	-0.26213	-0.31209	-0.5254	-1.39129	-1.43685	rc6
NR_027786	SERHL	0.049322	-0.22183	-0.16157	0.042974	-1.23393	-1.43663	rc6
NR_033227	CHTF8	0.247971	-0.02617	-0.51227	-0.67283	-0.29631	-1.43558	rc6
NM_032359	C3orf26	-0.17624	0.158614	-0.20956	-0.07941	-0.66375	-1.43549	rc6

NM_012089	ABCB10	-0.53777	-0.04188	-0.15308	-0.05313	-1.14619	-1.43484	rc6
NM_001128839	CACNA1D	-0.00706	0.106252	-0.16572	-0.46556	-0.90783	-1.43386	rc6
NM_001170716	BCAR1	0.161663	-0.24351	0.337863	0.449444	0.691196	-1.43090	rc6
NM_016592	GNAS	-0.10737	-0.49295	-0.42174	-0.3004	-0.96318	-1.42920	rc6
NM_018265	C1orf106	0.651889	-1.23366	-0.63276	-0.30674	-0.66111	-1.42704	rc6
NM_000056	BCKDHB	-0.03234	0.166365	-0.24872	0.091764	-0.74463	-1.42560	rc6
NM_001071	TYMS	-0.04933	-0.09581	0.14422	0.064586	-0.7632	-1.42494	rc6
NM_018283	NUDT15	-0.05009	-0.10144	-0.12874	0.211355	-1.13685	-1.42471	rc6
NM_002954	RPS27A	-0.75037	-1.04068	-0.57274	-0.66937	-1.13236	-1.42326	rc6
NM_015434	INTS7	-0.71999	0.068874	0.013125	0.65619	-0.86942	-1.41872	rc6
NM_199249	C19orf48	-0.12825	-0.30442	0.370683	0.160963	-1.07719	-1.41682	rc6
NM_014165	NDUFAF4	-0.11377	-0.05609	0.182074	0.428763	-1.12945	-1.41681	rc6
NM_024120	C20orf7	0.340676	0.150034	0.449677	-0.01279	-0.76997	-1.41627	rc6
NM_152259	C15orf42	0.167943	-0.69344	-0.33926	-0.23135	-0.95191	-1.41596	rc6
NM_194313	KIF24	0.208358	-0.24779	-0.53213	-1.06689	-0.9556	-1.41177	rc6
NM_014233	UBTF	0.124438	-0.34535	-0.64962	-0.33284	-1.31789	-1.40996	rc6
NM_004298	NUP155	-0.74028	0.44195	-0.38281	0.096953	0.006197	-1.40941	rc6
NM_001099678	LRRC58	-0.68255	-0.37424	-0.20151	0.158593	-1.21371	-1.40922	rc6
NM_001165412	NFKB1	-0.51265	0.856511	1.327362	1.220638	-0.08092	-1.40911	rc6
NM_024320	PRR15L	-0.19377	-1.39289	-1.1747	-0.68271	-1.17504	-1.40586	rc6
NM_001100624	CENPN	0.09456	0.328625	0.228077	0.306206	-1.10788	-1.40564	rc6
NM_014509	SERHL2	0.088477	-0.21087	-0.15981	0.141048	-1.15656	-1.40544	rc6
NR_023919	LINC00339	-0.16353	-0.37359	-0.92601	-1.02628	-1.09208	-1.40506	rc6
NM_001618	PARP1	0.02623	-0.08066	-0.00144	0.057455	-0.91359	-1.40398	rc6
NR_038109	LOC10050724	-0.27942	-0.71657	-0.2129	0.283385	-0.60484	-1.40379	rc6
NM_207046	ENSA	-0.1127	-0.26629	-0.42382	-0.52878	-0.84832	-1.40044	rc6
NM_015144	ZCCHC14	-0.0576	-0.27594	-0.28007	-0.19917	-1.09648	-1.39975	rc6
NM_001214907	ZNF48	-0.34029	-1.18828	-0.05066	-1.10711	-0.30689	-1.39969	rc6
NM_001128161	UBP1	-0.12388	-0.02054	-0.01019	0.372444	-1.00886	-1.39943	rc6
NM_144706	C2orf15	-0.0652	-0.64543	-0.782	-0.2353	-1.03342	-1.39638	rc6
NM_001198541	ERAP1	-0.31907	-0.30135	0.081342	0.583185	-0.65357	-1.39475	rc6
NM_020362	PITHD1	-0.11732	-0.15971	0.131465	0.145142	-1.26094	-1.39390	rc6
NM_001112706	SCIN	0.068693	0.106611	0.082913	0.010528	-0.99624	-1.39178	rc6
NM_004706	ARHGEF1	0.51719	0.14382	0.573492	-1.24911	-0.77989	-1.39051	rc6
NM_001251969	SLC30A5	0.160647	-0.68548	-1.20174	-0.01386	-1.18731	-1.38749	rc6
NM_016089	ZNF589	-0.07414	-0.39109	-0.47187	-0.46245	-0.81014	-1.38646	rc6
NM_181900	STARD5	-0.10607	-0.29676	-0.60563	-0.79295	-1.34398	-1.38513	rc6
NM_001126122	SLC25A19	0.151892	0.334162	-0.06425	-0.64334	-1.15881	-1.38269	rc6
NM_000170	GLDC	-0.11805	-0.23198	-0.2545	-0.28031	-1.25668	-1.38015	rc6
NM_002585	PBX1	0.09545	0.056852	-0.65215	-0.32244	-0.94774	-1.37995	rc6
NM_001099755	SYBU	-0.13587	-0.25362	-0.35789	-0.32762	-1.29471	-1.37923	rc6
NM_001201572	SEC22C	-0.32691	-0.33407	-0.26772	-0.67755	-1.27096	-1.37848	rc6
NR_028090	LOC202781	-0.11666	0.325562	-0.0458	0.138972	-1.21508	-1.37519	rc6
NM_024619	FN3KRP	-0.09728	-0.20143	-0.20634	-0.48708	-1.26289	-1.37448	rc6
NM_018062	FANCL	0.338581	-0.64513	-0.63652	0.02631	-1.26912	-1.37415	rc6
NM_001102369	C14orf159	0.289027	-1.1545	-1.18928	-0.95369	-1.35876	-1.37189	rc6
NM_000135	FANCA	-0.1021	0.040482	-0.19566	-0.35209	-1.16271	-1.37184	rc6

NM_145061	SKA3	0.062909	0.018508	-0.59429	0.353772	-0.41743	-1.37139	rc6
NM_014177	TIMM21	0.034635	0.021573	-0.20817	-0.12842	-0.94252	-1.36828	rc6
NM_001243147	NVL	-1.17653	0.124338	-0.66338	-0.6513	-0.75205	-1.36808	rc6
NM_174955	ATP2A3	0.327138	0.725111	-0.19894	-0.09687	-0.78747	-1.36558	rc6
NM_001199642	ADCY5	-0.14676	-0.15403	-0.54069	-0.35821	-0.9482	-1.36507	rc6
NM_020899	ZBTB4	0.522	-0.03753	-0.86286	-0.38194	-1.03622	-1.36422	rc6
NR_024374	LOC642846	-0.15877	-0.07123	-0.33684	-0.48828	-0.95241	-1.36339	rc6
NM_003653	COPS3	-0.05852	-0.01229	0.518518	0.608991	-0.70939	-1.36331	rc6
NM_017861	PIGX	-0.10625	-0.16772	-0.22483	-0.13335	-0.90359	-1.36228	rc6
NM_173570	ZDHHC23	-0.2891	0.217221	0.211987	0.322399	-0.87261	-1.36122	rc6
NM_001280	CIRBP	-0.04448	-0.09141	0.023157	0.137381	-1.12765	-1.36089	rc6
NM_152308	RMI2	-0.07875	-0.39016	-0.49971	-0.73736	-0.72068	-1.35988	rc6
NM_133263	PPARGC1B	1.142652	-0.30682	0.556001	0.843512	-1.21604	-1.35742	rc6
NM_003944	SELENBP1	-0.0287	-0.29664	-0.22971	-0.21761	-0.88323	-1.35520	rc6
NM_001001661	ZNF425	-0.26451	-0.63483	-1.01214	-0.75383	-1.33208	-1.35318	rc6
NM_021194	SLC30A1	-0.23609	0.21062	0.352334	0.56002	-1.0351	-1.35254	rc6
NM_004323	BAG1	0.036382	-0.09181	-0.07646	-0.08806	-1.22852	-1.34993	rc6
NR_038457	NAPA	-0.14308	-0.21524	0.60361	-0.18435	0.118842	-1.34157	rc6
NM_001202464	ZMYND11	-0.34931	0.002523	0.016985	-0.16273	-0.45755	-1.34075	rc6
NM_053274	GLMN	-0.03097	-0.07806	-0.00408	0.620828	-1.13734	-1.34030	rc6
NM_016622	MRPL35	0.00884	-0.13558	-0.36172	-0.14275	-1.20986	-1.33885	rc6
NM_001007277	EI24	-0.83508	-0.86483	0.172712	-0.31739	-1.12624	-1.33437	rc6
NR_026591	RAD1	-0.10444	-0.24499	-0.21146	0.008383	-0.64324	-1.33383	rc6
NM_005387	NUP98	0.45159	0.650602	1.121437	1.128269	-0.20946	-1.33369	rc6
NM_018097	HAUS2	-0.28062	-0.03015	0.436225	0.650577	-0.92292	-1.33355	rc6
NM_012091	ADAT1	-0.09346	-0.199	0.072359	-0.10247	-1.14144	-1.33350	rc6
NM_002077	GOLGA1	0.45636	0.23643	0.001833	-0.08094	-1.1394	-1.33321	rc6
NM_001199463	PDCD2	-0.01193	-0.12341	0.452196	0.784438	-1.02535	-1.33228	rc6
NM_002764	PRPS1	-0.04265	-0.09234	-0.04247	0.031253	-0.93507	-1.33050	rc6
NM_000466	PEX1	-0.12352	-0.16129	-0.33234	-0.31206	-1.07709	-1.33037	rc6
NM_018469	TEX2	-0.02703	0.014826	0.024593	0.209432	-0.80631	-1.32927	rc6
NM_015141	GPD1L	0.072156	-0.07949	-0.32721	-0.31874	-1.01688	-1.32903	rc6
NM_001143787	PPHLN1	-0.0937	-0.44066	-0.13357	-0.30026	-0.16318	-1.32794	rc6
NM_001262	CDKN2C	-0.24467	-0.42082	-0.9552	-1.21957	-1.22857	-1.32786	rc6
NM_178044	SLX1B	-0.23275	-1.22588	-0.65222	-0.44231	-0.4373	-1.32602	rc6
NM_005244	EYA2	0.01616	-0.08157	-0.5213	-1.18041	-1.025	-1.32540	rc6
NR_003614_1	PMS2L2	0.464033	-0.19084	-0.04067	-0.00206	-1.21423	-1.32535	rc6
NM_198395	G3BP1	0.508672	0.730164	-0.3558	0.749937	-0.04522	-1.32247	rc6
NM_006591	POLD3	0.156722	-0.16596	-0.72958	-0.52348	-0.68969	-1.31900	rc6
NM_138421	SAAL1	0.017821	0.050797	0.159215	0.231552	-0.5506	-1.31843	rc6
NM_145244	DDIT4L	0.119597	-0.15142	-0.91947	-0.7324	-1.03154	-1.31837	rc6
NM_001113378	FANCI	-0.07957	-0.09488	0.051255	0.059781	-0.47624	-1.31642	rc6
NR_003949	FKBP9L	-0.06872	-0.21091	-0.45019	-0.41667	-1.03277	-1.31388	rc6
NM_080794	MRPL39	-0.56372	0.339763	0.402321	0.399269	-0.40476	-1.31388	rc6
NM_176806	MOCS2	0.103561	0.052775	0.023441	0.486862	-0.87584	-1.31373	rc6
NM_201443	TEAD4	-0.5252	-0.69116	-0.14068	-0.34732	-0.76816	-1.31241	rc6
NM_001033	RRM1	0.033188	-0.01881	0.114159	0.185216	-0.62654	-1.31165	rc6

NM_033261	IDI2	0.219223	0.831488	0.544337	0.574531	-1.16696	-1.31075	rc6
NR_028597	SRD5A1P1	-0.30169	-0.4045	-0.29734	-0.15329	-1.02533	-1.31007	rc6
NM_147188	FBXO22	0.991362	0.32276	0.070338	1.502185	0.33112	-1.30935	rc6
NM_014252	SLC25A15	-0.17038	-0.28169	-0.27178	-0.01563	-1.18988	-1.30915	rc6
NM_013412	RABL2A	-0.36565	-0.56016	-0.77246	-1.02752	-1.009	-1.30853	rc6
NR_046310	PCGF5	-0.28547	-0.15027	-0.02714	0.181867	-1.06166	-1.30822	rc6
NM_138635	H2AFV	-0.08795	0.048495	-0.099	0.117175	-0.86312	-1.30696	rc6
NM_001177716	KLF11	0.222421	-0.04408	-0.76934	0.232544	-0.17602	-1.30458	rc6
NM_001198961	ECHDC2	-0.31672	0.153094	-0.12443	0.235495	-1.01019	-1.30443	rc6
NM_001199775	CPD	-0.58094	-0.02808	-0.2788	-0.00217	-0.70567	-1.30438	rc6
NM_014175	MRPL15	0.094709	-0.04734	0.1435	0.286136	-0.89981	-1.30323	rc6
NM_183075	CYP2U1	-0.36124	-0.26238	-0.73935	-0.37523	-1.14412	-1.30319	rc6
NM_001332	CTNND2	-0.10983	-0.10207	-0.17386	-0.21968	-1.0408	-1.30283	rc6
NM_001146068	CAPN2	-0.15098	-0.3346	-0.14184	0.79486	-0.47232	-1.29609	rc6
NM_001136005	GART	0.009852	0.02877	-0.73247	0.543764	-0.39948	-1.29604	rc6
NM_152755	CNPY4	0.006621	-0.32691	-0.85115	-0.94569	-1.11397	-1.29425	rc6
NM_001048201	UHRF1	-0.07568	-0.04614	0.061213	0.315467	-0.77199	-1.29317	rc6
NM_007057	ZWINT	-0.01518	-0.04268	0.067019	0.026699	-0.93504	-1.28958	rc6
NM_019014	POLR1B	0.076584	-0.12927	0.278318	0.801764	-0.73875	-1.28935	rc6
NM_022371	TOR3A	-0.09073	-0.25597	-0.43013	-0.46055	-1.11914	-1.28853	rc6
NM_015442	CNOT10	0.196307	-0.06081	-0.34763	0.241645	-0.5932	-1.28850	rc6
NM_001127711	TMEM14B	-0.12954	-0.41902	0.019368	0.533783	-1.1047	-1.28656	rc6
NM_014636	RALGPS1	0.009361	-0.10695	-0.45724	-0.49578	-0.91692	-1.28639	rc6
NM_001195427	SRSF2	0.289864	0.37793	0.624889	0.728747	-1.15122	-1.28638	rc6
NM_001177591	PPP2R2A	0.211238	0.637927	0.013054	1.003457	-0.41407	-1.28629	rc6
NM_147161	ACOT11	0.11872	-0.09894	-0.44173	-0.49811	-1.28108	-1.28569	rc6
NM_022720	DGCR8	-0.10877	-0.08229	-0.04063	-0.27821	-1.23158	-1.28521	rc6
NM_018246	CCDC25	-0.21845	-0.16874	-0.55171	-0.08781	-1.05823	-1.28372	rc6
NM_001047	SRD5A1	-0.14407	-0.3436	-0.10594	-0.18407	-0.98008	-1.28268	rc6
NM_001195100	PTP4A2	-0.45854	-1.16273	-0.99155	-0.02117	0.191968	-1.28018	rc6
NM_020730	DLG3	-0.04251	-0.19406	-0.78923	-0.45092	-1.02478	-1.27890	rc6
NM_001042618	PARP2	0.068873	-0.01954	-0.41294	-0.36143	-0.74077	-1.27823	rc6
NM_201521	KLC4	0.102198	-0.33606	-0.42313	-0.82284	-0.91851	-1.27802	rc6
NM_006397	RNASEH2A	0.131347	-0.31812	-0.19191	-0.01648	-0.84634	-1.27608	rc6
NM_016489	NT5C3	-0.59017	0.058603	0.136076	0.700543	-1.22597	-1.27489	rc6
NM_001242648	PHACTR1	-0.13844	-0.23328	-0.86354	-0.96948	-0.87144	-1.27171	rc6
NM_033319	CENPL	-0.3716	-0.12682	-0.62003	0.289521	-0.42756	-1.27103	rc6
NR_003098	SNHG1	-0.54287	0.045182	0.201979	0.259618	-0.74418	-1.27099	rc6
NM_018944	MIS18A	0.016707	0.065598	-0.33882	-0.12948	-0.97932	-1.27066	rc6
NM_001143997	OGDHL	-0.04765	-0.46187	-0.54037	-0.97105	-1.08491	-1.27030	rc6
NM_020151	STARD7	-0.10769	-0.14318	-0.05084	0.023174	-1.00914	-1.27029	rc6
NM_017613	DONSON	-0.09884	0.123281	0.236241	0.360907	-0.69634	-1.26774	rc6
NM_006392	NOP56	0.012355	0.058162	0.450372	0.552161	-1.08562	-1.26723	rc6
NM_006306	SMC1A	-0.05534	-0.08806	-0.0972	0.102041	-0.7599	-1.26686	rc6
NM_145644	MRPL35	0.28709	-0.33887	-0.25707	0.178476	-0.84329	-1.26666	rc6
NM_001167924	C3orf26	0.360444	-0.17639	0.305965	0.317756	-1.17545	-1.26598	rc6
NM_032756	HPDL	-0.05098	0.070273	0.418268	0.430461	-1.23897	-1.26450	rc6

NR_045017	LETMD1	-0.18346	-0.47621	-1.12505	-0.60433	-0.38317	-1.26447	rc6
NM_144680	ZNF18	0.053037	-0.54511	-0.4186	-0.3843	-0.94728	-1.26373	rc6
NR_022014	HMGN2P46	0.0777	-0.26621	-0.1494	-0.1615	-0.71636	-1.26251	rc6
NM_001127394	TSEN15	0.301449	0.324601	0.252096	0.688521	-1.09797	-1.26240	rc6
NM_001005374	LRSAM1	-0.67338	-0.39857	-1.08148	-0.83055	-0.90821	-1.26185	rc6
NM_001166265	LTBP1	-0.13441	-0.33588	-0.29522	-0.29368	-1.19601	-1.26131	rc6
NM_001005476	PKP4	0.171406	0.44292	0.343335	0.471908	-0.16863	-1.26060	rc6
NM_138970	NRXN3	-0.83587	-0.17231	-0.41962	-0.58223	-1.06003	-1.26030	rc6
NM_030653	DDX11	-0.057	-0.16012	-0.55783	-0.89295	-0.97072	-1.26010	rc6
NM_001144928	NKIRAS2	-0.35353	-0.80918	-0.13766	-0.35478	-0.39999	-1.25901	rc6
NM_004958	MTOR	-0.07706	-0.16279	-0.21826	-0.42753	-0.87714	-1.25863	rc6
NM_001113513	ARHGEF7	-0.14378	-0.75553	-0.57642	-0.57012	-0.25305	-1.25748	rc6
NM_201598	FAM86A	0.422825	-0.15577	0.059387	-0.15438	-0.872	-1.25414	rc6
NR_002932	GSTM2P1	-0.00379	-0.66202	-0.23584	-0.46968	-1.19154	-1.25341	rc6
NM_002696	POLR2G	0.056817	-0.33976	-0.21758	0.159665	-1.05079	-1.25311	rc6
NM_002553	ORC5	0.00259	0.001613	-0.48216	-0.00826	-0.97389	-1.25266	rc6
NM_133335	WHSC1	-0.0358	-0.35068	-0.4524	0.021954	0.191344	-1.25241	rc6
NM_001130926	MEF2A	-0.32102	-0.93726	-0.18962	0.178437	0.117276	-1.25150	rc6
NM_032352	BRMS1L	-0.99528	-0.35707	-0.05483	0.32985	-0.468	-1.25137	rc6
NM_031965	GSG2	0.095171	0.154057	0.079586	-0.2716	-0.67312	-1.25110	rc6
NM_020421	ADCK1	-0.34059	-0.33561	-0.35385	-0.53517	-0.77768	-1.24990	rc6
NM_001249	ENTPD5	-0.00225	-0.01648	-0.27649	-0.22692	-0.91036	-1.24959	rc6
NM_001516	GTF2H3	0.155673	-0.09208	0.015021	0.564397	-0.97282	-1.24946	rc6
NM_018297	NGLY1	-0.30814	-0.31807	-0.56384	-0.1334	-0.28427	-1.24891	rc6
NM_144677	MGAT5B	0.60971	0.792475	0.400669	0.530384	-0.1054	-1.24885	rc6
NM_001167733_2	VARS2	-0.01265	-0.11018	-0.52256	-0.3478	-0.93766	-1.24698	rc6
NM_018394	ABHD10	-0.14896	-0.22119	-0.7629	-0.25657	-1.1373	-1.24654	rc6
NR_001285	SNORD35B	0.434255	0.555343	0.523642	0.307005	-0.47126	-1.24600	rc6
NM_001129888	CYHR1	-0.89493	0.36359	-0.19598	-0.53858	-0.92895	-1.24567	rc6
NM_005879	TRAIP	-0.00246	-0.40713	-0.76532	-0.84054	-1.08753	-1.24540	rc6
NM_177974	CASC4	-0.57055	-0.37143	-0.30302	0.44588	-0.21599	-1.24500	rc6
NM_001098502	CHCHD4	-0.07798	-0.2561	-0.33746	-0.00296	-0.83431	-1.24447	rc6
NM_001008708	CHAC2	0.362845	0.107291	0.193059	0.169777	-1.14085	-1.24309	rc6
NM_003579	RAD54L	-0.15792	-0.26315	-0.26543	-0.56429	-0.56876	-1.24237	rc6
NM_182984	TRMT2A	-0.09837	-0.34514	-0.99401	-0.47754	-1.10564	-1.24210	rc6
NM_014793	LCMT2	0.433611	0.215057	0.081342	0.118932	-1.01592	-1.24091	rc6
NR_027334	LOC10013169	-0.34298	-0.90236	-1.15759	-0.88888	-1.05343	-1.23988	rc6
NM_001012993	C9orf152	-0.09	-0.62469	-1.15313	-0.98677	-1.06967	-1.23961	rc6
NM_003621	PPFIBP2	0.15095	-0.18038	-0.79872	-1.09236	-0.51055	-1.23831	rc6
NM_002821	PTK7	-0.8383	-0.55127	-0.56946	-0.88563	-1.22208	-1.23793	rc6
NM_014435	NAAA	-0.15011	-0.12288	-0.04311	0.277158	-0.86978	-1.23671	rc6
NM_014736	KIAA0101	-0.06818	-0.10346	-0.1882	0.052042	-0.40424	-1.23355	rc6
NM_021144	PSIP1	-0.51954	-0.55905	-0.96946	-0.33539	-1.04741	-1.23298	rc6
NM_001008662	CCBL2	-0.18478	-0.24708	-0.55063	-0.37497	-0.2646	-1.23266	rc6
NM_001905	CTPS	0.097236	0.442343	0.829289	0.563342	-0.79778	-1.23206	rc6
NM_015341	NCAPH	0.110536	-0.13411	-0.23432	-0.26328	-0.56134	-1.23170	rc6
NM_152857	WTAP	-0.08326	0.158082	-0.04586	0.077841	-0.63753	-1.23151	rc6

NM_018154	ASF1B	0.003	-0.2024	-0.29019	-0.52409	-0.81041	-1.23085	rc6
NM_031426	AIF1L	-0.02219	-0.3568	-0.28831	-0.44568	-1.07852	-1.22913	rc6
NM_020379	MAN1C1	-0.01495	-0.12045	-0.66427	-0.98142	-1.06383	-1.22764	rc6
NM_022909	CENPH	-0.17626	-0.36805	-0.2462	0.272334	-0.5843	-1.22761	rc6
NM_001512	GSTA4	-0.13097	-0.12404	-0.11638	0.113919	-0.81514	-1.22632	rc6
NM_203399	STMN1	-0.06132	-0.31483	-0.23813	-0.51044	-0.65576	-1.22532	rc6
NM_014426	SNX5	0.027754	0.106398	0.097244	0.333482	-0.95614	-1.22377	rc6
NR_040772	LOC10050566	-0.05951	0.162685	-0.41323	-0.22449	-1.07561	-1.22300	rc6
NM_001242926	ZNF410	-0.27984	-0.10697	0.022793	-0.51197	-0.49642	-1.22133	rc6
NM_024039	MIS12	0.116494	-0.15519	-0.51298	-0.49584	-0.96699	-1.22093	rc6
NM_003594	TTF2	0.04559	0.081351	0.004744	0.123207	-0.69468	-1.22039	rc6
NM_152379	C1orf131	0.167257	-0.25409	-0.10054	0.053422	-0.85298	-1.21966	rc6
NM_058182	FAM165B	-0.04723	-0.2499	-0.29292	-0.05975	-0.84633	-1.21902	rc6
NM_006606	RBBP9	0.100865	-0.17567	-0.1612	0.141538	-1.01965	-1.21902	rc6
NM_018386	PCID2	1.241672	1.074962	-0.06598	-0.50925	1.035832	-1.21892	rc6
NM_199162	ADPRHL1	-0.22267	-0.53068	-0.6967	-0.86546	-1.10744	-1.21871	rc6
NM_005226	S1PR3	-0.20118	-0.46545	-1.11474	-1.11347	-0.81705	-1.21517	rc6
NR_024451	LOC10013422	0.212247	-0.0712	-0.6757	-0.51135	-0.81916	-1.21409	rc6
NM_001244926	PRPF4	0.080606	-0.36451	0.033797	0.115014	-0.62594	-1.20913	rc6
NM_006134	TMEM50B	0.234754	0.155552	0.167758	0.225657	-0.5071	-1.20793	rc6
NM_004117	FKBP5	-0.14885	0.003228	0.317705	0.837383	-0.39332	-1.20747	rc6
NM_001145375	ALKBH2	0.190693	0.135958	-0.56135	-0.07761	-1.01409	-1.20737	rc6
NR_033759	ATP5L	0.411274	-0.52268	-0.10824	0.871719	-1.05748	-1.20736	rc6
NM_003032	ST6GAL1	0.056422	-0.03356	-0.05069	-0.35706	-0.96817	-1.20690	rc6
NM_016053	CCDC53	-0.16683	0.063484	-0.21974	-0.15663	-0.87923	-1.20597	rc6
NM_006876	B3GNT1	-0.20491	-0.7203	-1.06825	-0.87366	-1.19722	-1.20590	rc6
NM_025247	ACAD10	-0.094	-0.19986	-0.48529	-0.55758	-0.94468	-1.20454	rc6
NM_024625	ZC3HAV1	0.269861	-0.17801	0.214325	0.065114	-0.46547	-1.20453	rc6
NM_020662	MRS2	0.073722	-0.06494	0.095265	0.413621	-0.83599	-1.20437	rc6
NM_022786	ARV1	0.138224	0.34706	-0.12766	0.201604	-0.86407	-1.20385	rc6
NM_017775	TTC19	-0.11461	-0.08121	0.092625	0.483522	-1.088	-1.20368	rc6
NM_152945	RBM45	-0.32229	0.138759	0.064013	0.220228	-0.90207	-1.20331	rc6
NM_015294	TRIM37	-0.0816	-0.06363	-0.49543	-0.32487	-0.82259	-1.20247	rc6
NM_001193357	NUP62	0.530651	0.524066	0.106205	0.293657	-0.09546	-1.19968	rc6
NM_005780	LHFP	-0.18999	-0.10049	-0.1843	0.26886	-0.82757	-1.19943	rc6
NR_037177_7	LOC10029414	-0.1382	0.02348	-0.61533	-0.33672	-0.76009	-1.19770	rc6
NM_001085471	FOXN3	-0.72965	-0.93109	-0.84883	-0.60939	-0.63938	-1.19630	rc6
NM_170725	PGBD2	0.410504	0.009978	-0.24097	-0.14059	-0.93835	-1.19599	rc6
NM_005916	MCM7	0.033075	-0.05345	0.065754	-0.10918	-0.84669	-1.19562	rc6
NM_152424	FAM123B	-0.11387	-0.03213	0.210929	0.309344	-0.83033	-1.19542	rc6
NM_001143841	TMEM106C	-0.1276	-0.30953	-0.35498	-0.41651	-0.83021	-1.19520	rc6
NR_033399	DDX12P	-0.07563	-0.10259	-0.23103	-0.57362	-0.98192	-1.19477	rc6
NM_017812	CHCHD3	-0.04072	-0.13811	-0.0433	0.048035	-0.78702	-1.19464	rc6
NM_001136130	APP	-0.06787	-0.27433	-0.55115	-0.21593	-1.11664	-1.19445	rc6
NM_024622	FASTKD1	-0.30604	0.089589	-0.60884	-0.85662	-0.73384	-1.19410	rc6
NM_001083961	WDR62	0.024856	-0.39283	-0.86872	-0.75374	-0.79082	-1.19389	rc6
NM_006527	SLBP	0.064313	-0.13614	-0.14629	0.141076	-1.04671	-1.19259	rc6

NM_014859	ARHGAP44	-0.09413	-0.05244	-0.23853	-0.74973	-1.09935	-1.19108	rc6
NR_024249	FAM86C2P	-0.11398	-0.47211	-0.75747	-0.54401	-0.99054	-1.19082	rc6
NM_001003722	GLE1	-0.12112	-0.25642	-0.36036	-0.53822	-1.07483	-1.19079	rc6
NM_015135	NUP205	0.123108	0.030357	-0.01801	-0.08752	-0.62595	-1.18563	rc6
NR_027052	THAP7-AS1	0.540575	-0.04679	-0.7414	0.1257	-0.97242	-1.18552	rc6
NM_018137	PRMT6	-0.05244	-0.05624	-0.21196	-0.05717	-0.85901	-1.18516	rc6
NM_016647	C8orf55	-0.02812	-0.30441	-0.40018	-0.26938	-1.16615	-1.18464	rc6
NM_018463	ITFG2	-0.24995	-0.43297	-0.36166	-0.28103	-1.17489	-1.18296	rc6
NM_001243750	NUDT8	0.101352	-0.11928	-0.26597	-0.121	-0.9172	-1.18252	rc6
NM_014503	UTP20	-0.06909	0.201226	-0.01136	0.419698	-0.717	-1.18165	rc6
NM_000848	GSTM2	0.151915	-0.40853	-0.18255	0.062614	-1.04918	-1.18065	rc6
NM_014285	EXOSC2	0.075008	-0.11526	0.030674	0.184397	-0.97735	-1.18009	rc6
NM_017945	SLC35A5	0.001417	0.274686	-0.30312	-0.54129	-1.03251	-1.17607	rc6
NM_181656	C17orf58	-0.31567	-0.72264	-0.86705	-0.12545	-0.73381	-1.17600	rc6
NM_012437	SNAPIN	-0.05641	-0.286	-0.23798	0.006261	-1.08614	-1.17569	rc6
NM_033109	PNPT1	-0.29468	0.11629	-0.0468	0.505966	-0.58506	-1.17466	rc6
NM_014033	METTL7A	-0.22476	-0.35045	-0.40283	-0.39482	-0.99403	-1.17386	rc6
NM_003514	HIST1H2AM	-0.60407	-0.07927	-0.1233	-0.60307	-0.53253	-1.17157	rc6
NM_177989	ACTL6A	0.170838	0.330126	0.004981	0.575968	-0.21817	-1.17140	rc6
NM_007194	CHEK2	-0.48448	-0.18418	-0.61619	-0.28835	-0.86966	-1.17083	rc6
NM_001080449	DNA2	-0.5097	0.091848	-0.0941	0.162522	-0.65595	-1.17060	rc6
NM_016101	NIP7	-0.13615	0.200006	0.630313	0.70014	-0.93729	-1.17008	rc6
NM_138443	HAUS1	-0.2586	0.040905	-0.19942	-0.29089	-0.79855	-1.16879	rc6
NM_012192	FXC1	0.026579	-0.18276	-0.1624	-0.05814	-0.92682	-1.16837	rc6
NR_002324	SNORA62	-0.2857	0.516871	-0.64902	-1.0615	0.175241	-1.16818	rc6
NM_000411	HLCS	-0.33169	-0.13191	-0.25897	0.071927	-0.96253	-1.16718	rc6
NM_015056	RRP1B	-0.00804	-0.07992	0.014046	0.422665	-0.86178	-1.16562	rc6
NM_001048194	RCC1	-0.2857	-0.39335	-0.04419	-0.18621	-0.75136	-1.16517	rc6
NM_018332	DDX19A	0.036349	-0.16489	-0.11072	-0.11074	-1.14685	-1.16504	rc6
NM_005441	CHAF1B	0.007133	-0.1909	-0.30114	-0.4837	-0.99252	-1.16400	rc6
NR_029377	C20orf7	-0.83016	0.015151	-0.77578	0.54216	-0.55712	-1.16396	rc6
NM_005573	LMNB1	0.47959	-0.21255	-0.16892	0.185751	-1.06226	-1.16245	rc6
NM_001178080	STAT6	-0.52315	1.487824	0.985355	1.300187	1.848402	-1.16186	rc6
NM_001048265	C9orf116	0.150277	0.300622	0.160213	-0.11451	-0.85396	-1.16150	rc6
NR_002325	SNORA6	-0.69049	-0.13096	-0.27273	-0.10647	-0.43024	-1.15993	rc6
NM_199177	MRRF	-0.05126	-0.01899	-0.65383	-0.35829	-0.49096	-1.15963	rc6
NM_182757	RNF144B	-0.08625	-0.54047	-0.68299	-0.48127	-0.96625	-1.15896	rc6
NM_182789	PAIP1	-0.55515	-0.46671	-0.57189	-1.14995	-0.32202	-1.15797	rc6
NM_198476	C19orf54	-0.14314	-0.03579	-0.49512	-0.64163	-1.03339	-1.15711	rc6
NM_030929	KAZALD1	-0.16863	-0.04069	-0.38171	-0.39256	-1.03345	-1.15633	rc6
NM_001037305	THYN1	-0.1085	0.296585	-0.15698	-0.11249	-0.85339	-1.15577	rc6
NM_004249	RAB28	0.353692	0.123778	-0.32992	0.188874	-0.04887	-1.15557	rc6
NM_024675	PALB2	-0.25031	-0.34503	0.072952	0.150686	-0.82738	-1.15510	rc6
NM_001238	CCNE1	0.211525	-0.06713	-0.0421	-0.12443	-1.08342	-1.15476	rc6
NM_153269	C20orf96	-0.01087	-0.34486	-0.2247	-0.66133	-0.86571	-1.15191	rc6
NM_001005413	ZWINT	-0.67107	-0.17235	-0.33739	-0.53762	-1.02525	-1.15012	rc6
NM_002566	P2RY11	0.157047	0.205198	0.028512	0.387366	-1.12101	-1.14975	rc6

NM_003804	RIPK1	-0.13971	0.116994	0.142248	-0.01197	-0.84541	-1.14935	rc6
NM_020775	KIAA1324	-0.08518	-0.27719	-0.31491	-0.22144	-0.69975	-1.14796	rc6
NM_015697	COQ2	-0.0385	-0.13374	-0.24285	0.33382	-0.93573	-1.14616	rc6
NM_022836	DCLRE1B	-0.1588	-0.07283	0.01273	-0.1438	-0.65111	-1.14521	rc6
NM_006904	PRKDC	-0.11151	-0.02669	-0.09189	0.335315	-0.41257	-1.14512	rc6
NM_016376	ANKFY1	-0.08668	-0.23815	-0.30146	-0.10949	-0.71122	-1.14506	rc6
NM_000292	PHKA2	-0.02854	-0.07205	-0.04991	-0.26844	-1.0393	-1.14480	rc6
NM_001079524	PAICS	0.118095	0.037588	0.016928	0.353542	-0.73859	-1.14451	rc6
NM_006822	RAB40B	0.225383	0.179785	-0.02363	0.255205	-0.78606	-1.14416	rc6
NM_145738	SYNGR1	0.016934	0.266201	-0.58835	-0.55431	0.523058	-1.14346	rc6
NM_015907	LAP3	0.15327	0.089069	0.051347	0.22146	-0.80121	-1.14322	rc6
NM_018342	TMEM144	-0.26828	-0.04191	-0.15138	-0.02391	-0.92498	-1.14005	rc6
NM_001012716	C18orf56	-0.65009	-0.42256	-0.33293	-0.2233	-0.52839	-1.14000	rc6
NM_018322	SAYSD1	0.0017	-0.419	-0.53505	-0.2632	-0.79785	-1.13874	rc6
NM_018092	NETO2	-0.19478	-0.24486	-0.59733	0.008902	-0.51895	-1.13820	rc6
NR_015389	LOC339290	-0.07698	0.204957	-0.26146	-0.01844	-1.12369	-1.13783	rc6
NM_012405	ICMT	-0.1823	-0.32948	-0.25965	-0.26023	-0.93955	-1.13646	rc6
NM_145048	PACRGL	-0.13443	-0.14774	-0.11283	-0.11365	-0.50259	-1.13625	rc6
NM_025125	FAM213A	-0.07242	-0.2604	-0.30847	-0.07801	-0.98633	-1.13602	rc6
NR_024282	LOC113230	0.038208	0.043343	-0.40332	-0.55417	-0.96763	-1.13565	rc6
NM_005033	EXOSC9	0.293476	0.354065	0.300909	0.345271	-0.36972	-1.13559	rc6
NM_138395	MARS2	0.085738	0.048612	0.334475	0.467568	-1.03345	-1.13444	rc6
NM_016126	HSPB11	-0.00313	-0.11881	-0.23111	0.001123	-0.83459	-1.13365	rc6
NM_020675	SPC25	0.132296	-0.02768	0.24689	0.377901	-0.60248	-1.13355	rc6
NR_028473	TMEM138	0.641173	0.541175	0.076719	0.430801	-0.58765	-1.13354	rc6
NM_012106	ARL2BP	-0.2103	-0.27938	-0.20695	-0.1627	-0.87337	-1.13233	rc6
NM_003705	SLC25A12	-0.0433	-0.09275	-0.33742	-0.43912	-0.79693	-1.13142	rc6
NM_014498	GOLIM4	-0.36589	-0.19282	-0.34302	-0.00264	-0.92752	-1.13127	rc6
NM_133642	LARGE	-0.3491	-0.42525	-0.9835	-0.94726	-0.74651	-1.13042	rc6
NR_024538	GSTM4	-0.06811	-0.94582	0.189629	-0.42152	-0.90013	-1.13018	rc6
NM_001163474	ZNF746	-1.01232	-0.56105	-0.36658	-0.67991	-0.4392	-1.12955	rc6
NM_003562	SLC25A11	0.047211	-0.31671	-0.25554	0.123512	-0.77975	-1.12942	rc6
NR_034072	SARS	0.023174	0.121212	0.477859	0.964452	-0.65301	-1.12750	rc6
NM_032730	RTN4IP1	0.071924	-0.11413	-0.09529	-0.18224	-0.62589	-1.12749	rc6
NM_001042559	EIF4G2	-0.22175	0.146331	0.980091	1.310544	0.04763	-1.12653	rc6
NM_001039362	ATP6V1C2	-0.12071	0.072812	0.198254	-0.15848	-0.67872	-1.12604	rc6
NM_001078176	SLC29A1	-0.34058	-0.47616	-0.10537	-0.25252	-1.05424	-1.12573	rc6
NM_001031701	NT5DC3	0.235037	0.641746	0.637836	0.541626	-0.93596	-1.12358	rc6
NR_003584	SNHG8	0.184905	0.030228	0.031398	0.134225	-0.61104	-1.12344	rc6
NM_006231	POLE	-0.07782	-0.08277	-0.04525	-0.3824	-0.78962	-1.12243	rc6
NM_001921	DCTD	0.353393	-0.44652	-0.62249	-0.53664	0.106238	-1.12109	rc6
NM_145799	42984	-0.08586	-0.42501	-0.11542	0.156935	-0.88203	-1.12022	rc6
NM_003276	TMPO	0.024731	0.038816	0.210854	0.460664	-0.40765	-1.11840	rc6
NM_001379	DNMT1	0.055714	-0.03222	-0.03947	-0.07258	-0.52645	-1.11806	rc6
NM_001098813	42986	-0.3864	-0.37283	-0.0812	-0.13487	-0.66358	-1.11715	rc6
NM_001080414	CCDC88C	-0.10073	-0.14846	-0.13437	0.072107	-1.01856	-1.11656	rc6
NM_001142936	DAGLB	-0.48765	0.128787	-0.12271	-0.00289	-1.09192	-1.11524	rc6

NR_024158	PP7080	-0.04286	-0.02708	0.048678	0.03379	-0.9439	-1.11453	rc6
NM_015235	CSTF2T	-0.04033	-0.32734	-0.58462	-0.24722	-0.89834	-1.11362	rc6
NM_030621	DICER1	-0.64336	-0.32504	-0.39016	0.050271	-0.29453	-1.11343	rc6
NM_144607	CYB5D1	0.013184	-0.0937	-0.20431	0.427312	-0.87194	-1.11246	rc6
NM_015361	R3HDM1	0.015416	-0.15408	-0.28743	-0.21229	-0.82117	-1.11166	rc6
NM_001243137	PDE8A	0.310915	-0.59544	-0.32028	0.185904	-0.87055	-1.11098	rc6
NM_003630	PEX3	-0.13236	0.137364	0.05411	0.414135	-0.80101	-1.10978	rc6
NM_152416	C8orf38	0.138557	-0.141	-0.04879	0.383424	-1.03434	-1.10807	rc6
NR_002911	SNORA71A	-0.09084	-0.19521	0.246262	-0.10006	0.039163	-1.10689	rc6
NR_045992	ETF1	0.674274	0.365077	-0.51266	0.936172	0.35261	-1.10548	rc6
NM_001195218	DCXR	0.338621	0.100398	-0.63874	-0.19938	-0.04723	-1.10530	rc6
NM_002453	MTIF2	-0.51301	0.009822	-0.20281	-0.2486	-0.7394	-1.10440	rc6
NM_001143843	TMEM106C	0.422334	-0.89884	1.583007	1.078812	-0.63928	-1.10376	rc6
NM_080650	ATPBD4	0.26648	-0.43929	-0.2387	-0.50088	-0.54123	-1.10300	rc6
NM_004741	NOLC1	0.14632	0.205467	0.574774	0.808317	-0.73135	-1.10283	rc6
NM_016059	PPIL1	0.111005	-0.00545	0.165747	0.25336	-0.6176	-1.10131	rc6
NM_013367	ANAPC4	-0.36574	-0.07565	0.032341	-0.23096	-0.62317	-1.10130	rc6
NM_170784	MKKS	0.02565	-0.08911	-0.25906	-0.02508	-0.8557	-1.10012	rc6
NM_138779	TEX30	0.082561	0.355941	-0.00372	0.099472	-0.71324	-1.09927	rc6
NM_001242674	AHCYL1	-0.01392	-0.09178	-0.08414	0.185522	-0.3798	-1.09823	rc6
NM_052865	C20orf72	-0.10929	-0.04352	-0.41473	-0.04887	-0.65087	-1.09811	rc6
NM_001256438	CABLES1	0.17105	-0.34525	-0.10404	0.156983	-0.28198	-1.09782	rc6
NM_032590	KDM2B	-0.02438	0.169786	0.067228	0.013156	-1.03827	-1.09759	rc6
NM_005662	VDAC3	0.145538	0.041628	0.148992	0.245707	-0.72246	-1.09377	rc6
NM_021953	FOXM1	-0.23994	-0.40824	0.086814	-0.86185	-0.66572	-1.09253	rc6
NM_005517	HMGN2	-0.07865	-0.35749	-0.22909	-0.13907	-0.83336	-1.09246	rc6
NM_032726	PLCD4	-0.20271	-0.01157	-0.20781	-0.07763	-0.97794	-1.09184	rc6
NM_002610	PDK1	0.127621	0.090516	0.015891	0.715902	-0.98281	-1.09087	rc6
NM_015226	CLEC16A	-0.14642	-0.14594	-0.26391	-0.48787	-0.97688	-1.09036	rc6
NM_019042	PUS7	-0.08115	0.191503	0.321985	0.809738	-0.55646	-1.09014	rc6
NM_001206984	METTL23	-0.15949	-0.35002	-0.5443	-0.47683	-0.80492	-1.08918	rc6
NM_007270	FKBP9	-0.06567	-0.1321	-0.21219	-0.30515	-0.78382	-1.08774	rc6
NM_001190329	ATP5G3	-0.00475	-0.08225	0.099796	0.425476	-0.59896	-1.08712	rc6
NR_038279	LOC10050693	-0.06432	-0.0408	-0.14942	-0.13975	-0.95408	-1.08710	rc6
NM_031922	REPS1	-0.1093	-0.17903	0.22967	0.098104	-0.4464	-1.08648	rc6
NR_023345	CNKSR1	-0.21148	0.619156	-0.1544	0.800207	1.695577	-1.08384	rc6
NM_001029991	METTL17	0.093242	-0.16035	0.292147	-0.03148	-0.70534	-1.08373	rc6
NM_001105570	NUDT19	-0.06654	0.031259	-0.06128	0.184331	-1.05537	-1.08345	rc6
NM_001077351	RBM23	-0.03372	0.05097	-0.27511	-0.70326	-1.08026	-1.08223	rc6
NR_027322	LOC283070	-0.23606	0.105166	-0.36664	-0.60469	-0.84502	-1.08149	rc6
NM_174928	N6AMT2	0.376263	-0.24018	-0.07774	-0.6899	-0.75979	-1.08070	rc6
NM_001173473	ASMTL	0.234058	0.164561	0.002991	-0.0894	-0.20348	-1.07939	rc6
NM_177973	SULT2B1	0.206454	-0.4999	-0.39763	-0.24119	-0.93873	-1.07777	rc6
NM_032822	FAM136A	-0.07162	-0.20262	-0.08539	0.146929	-0.71091	-1.07744	rc6
NM_022810	SLC25A14	-0.01776	-0.06204	-0.71284	0.363959	-0.5264	-1.07743	rc6
NM_033119	NKD1	0.03805	-0.05673	-0.75551	-0.93489	-0.60491	-1.07701	rc6
NM_181842	ZBTB12	-0.19676	-0.42122	-1.04835	-0.42906	-0.84856	-1.07611	rc6

NM_080668	CDCA5	-0.07144	-0.22822	-0.19017	-0.25544	-0.67664	-1.07420	rc6
NM_003488	AKAP1	-0.16178	-0.24636	-0.47627	-0.24713	-0.93708	-1.07397	rc6
NM_201647	STAMBP	-0.04268	-0.38986	-0.55924	-0.05435	-0.71117	-1.07248	rc6
NM_018025	GPATCH1	0.057651	-0.40627	-0.51829	-0.50542	-0.81223	-1.07221	rc6
NR_038830	PIN1	0.814376	0.921947	0.059332	1.885433	0.071624	-1.07185	rc6
NM_001039349	EFEMP1	0.161732	-0.04217	0.119598	-0.22389	-0.9088	-1.07075	rc6
NM_021075	NDUFV3	0.071364	-0.33258	-0.56215	-0.17929	-0.9329	-1.07028	rc6
NR_003054	SNORD65	-0.23081	-0.70229	-0.03973	0.299304	0.119379	-1.07009	rc6
NM_031220	PITPNM3	0.058469	-0.15449	0.043221	-0.68739	-0.85605	-1.06468	rc6
NM_012140	SLC25A10	0.054083	-0.19629	-0.30919	-0.35194	-1.04053	-1.06456	rc6
NM_032382	COG8	-0.02722	-0.66714	-0.79743	-0.7037	-0.97307	-1.06348	rc6
NM_014424	HSPB7	0.100827	-0.08813	-0.10364	-0.13338	-0.74445	-1.06259	rc6
NM_015084	MRPS27	-0.00463	-0.24788	-0.32146	-0.37322	-0.78695	-1.06153	rc6
NM_002417	MKI67	0.47758	0.503763	-0.12568	0.842967	-0.29952	-1.05997	rc6
NM_016052	RRP15	-0.20122	0.18989	0.414858	0.807748	-0.60784	-1.05982	rc6
NM_004477	FRG1	-0.24212	-0.0836	0.120758	0.287768	-0.74938	-1.05971	rc6
NM_020700	PPM1H	0.000451	-0.06109	-0.79102	-0.98711	-0.71086	-1.05957	rc6
NM_005327	HADH	0.060504	-0.24974	-0.17383	-0.11556	-0.69501	-1.05836	rc6
NM_139235	NOL6	-0.62438	-0.69838	0.025553	-0.36387	-0.83629	-1.05765	rc6
NM_001204827	XPNPEP3	-0.14959	-0.34564	-0.40188	0.307933	-0.78322	-1.05666	rc6
NM_022061	MRPL17	0.102878	-0.30207	-0.09066	0.403508	-0.8206	-1.05663	rc6
NM_024551	ADIPOR2	0.070419	0.244747	0.283232	0.089057	-0.85791	-1.05632	rc6
NM_000140	FECH	-0.00478	-0.09347	-0.04591	0.080018	-0.88061	-1.05622	rc6
NM_003016	SRSF2	-0.19593	0.575389	0.377923	0.299374	-0.60662	-1.05530	rc6
NM_017906	PAK1IP1	0.097449	0.183548	0.118183	0.697601	-0.83073	-1.05529	rc6
NM_020806	GPHN	0.09346	-0.01515	-0.05595	-0.0675	-0.71841	-1.05506	rc6
NM_001142471	INTS12	0.185453	0.298191	0.218053	0.427816	-0.77903	-1.05471	rc6
NM_001164811	PET117	-0.118	-0.13792	-0.15344	0.083026	-0.63456	-1.05394	rc6
NR_001456	SNORD38A	0.782037	1.757045	0.84529	2.054046	2.254399	-1.05343	rc6
NM_000268	NF2	0.744381	0.38145	-0.47243	0.212162	0.21965	-1.05198	rc6
NM_018198	DNAJC11	-0.02592	-0.11077	-0.08178	-0.25279	-1.0278	-1.05136	rc6
NM_001039583	PICK1	-0.01991	-0.0214	-0.28133	0.915081	-0.85532	-1.04984	rc6
NM_001006656	ZNF473	0.154613	-0.40865	0.107704	0.082702	-0.61069	-1.04945	rc6
NM_001242534	MFSD11	-0.2633	0.309354	0.132611	-0.6158	-0.1167	-1.04901	rc6
NM_032638	GATA2	0.171323	-0.39233	-0.51457	-0.49416	-0.63782	-1.04835	rc6
NM_015396	ARMC8	-0.1669	0.105851	-0.27908	0.028973	-0.48652	-1.04785	rc6
NM_001077527	JRK	-0.4671	-0.83122	-0.31738	-0.2545	-0.86596	-1.04783	rc6
NR_033805	LOC220906	-0.09539	-0.03111	0.064718	0.43133	-0.82915	-1.04704	rc6
NR_002473_1	LOC440354	-0.24429	-0.09855	0.278333	0.339386	-0.57698	-1.04587	rc6
NM_032276	RHBDD1	-0.29208	-0.15912	-0.42826	-0.26177	-0.66218	-1.04567	rc6
NM_003432	ZNF131	-0.18462	0.290053	-0.0941	-0.0763	-0.63378	-1.04519	rc6
NM_013300	FAM216A	-0.25614	0.206658	-0.08834	0.48687	-0.46204	-1.04514	rc6
NM_033480	FBXO9	-0.07098	-0.06995	0.02915	0.198254	-0.71467	-1.04179	rc6
NR_003040	RPL23AP64	-0.55339	-0.26016	-0.15143	0.189684	-0.15815	-1.04149	rc6
NM_001039844	ACBD7	-0.29546	-0.28436	-0.21103	-0.15508	-0.70777	-1.04072	rc6
NM_006460	HEXIM1	0.129816	0.155573	-0.77247	-0.78486	-0.99081	-1.04020	rc6
NM_001315	MAPK14	-0.24194	-0.15092	-0.90693	-0.01452	-0.16362	-1.03970	rc6

NM_001080542	FBF1	0.009673	0.0361	-0.20616	-0.8429	-0.62529	-1.03951	rc6
NM_001013672	C17orf97	0.018161	-0.76639	-0.69121	-0.69824	-0.84491	-1.03906	rc6
NM_024657	MORC4	-0.10676	0.354806	0.510599	0.333176	-0.82906	-1.03754	rc6
NM_016940	RWDD2B	0.207728	-0.19369	-0.41151	-0.07982	-0.49724	-1.03662	rc6
NM_001145971	RDH13	0.249595	0.190834	-0.22403	-0.25717	-0.9532	-1.03485	rc6
NM_152490	B3GALNT2	-0.13949	-0.1014	-0.13008	0.413222	-0.52472	-1.03363	rc6
NM_001025579	NDEL1	0.11626	1.739352	1.399281	1.436198	-0.09762	-1.03290	rc6
NM_015908	SRRT	-0.26448	-0.09038	0.129232	0.107803	-0.78231	-1.03254	rc6
NM_199250	C19orf48	0.184825	0.343549	0.251346	0.142101	-0.92436	-1.02845	rc6
NM_001190440	NCOR1	0.291786	-0.1395	-0.59361	0.352118	-0.28412	-1.02823	rc6
NM_001146160	TATDN1	0.318846	-0.85639	-0.80833	1.200316	-0.48907	-1.02810	rc6
NM_006542	SPHAR	-0.09031	0.304056	-0.26426	0.046085	0.081016	-1.02798	rc6
NM_014345	ZNF318	-0.02635	-0.34383	-0.6546	-0.29021	-0.55265	-1.02758	rc6
NR_002952	SNORA9	-0.89423	-0.08106	0.749312	0.544515	-0.61248	-1.02697	rc6
NR_026950	LOC283922	-0.22053	-0.34545	-0.36454	-0.60343	-0.73262	-1.02668	rc6
NM_001826	CKS1B	-0.02419	-0.25552	-0.09161	0.072851	-0.54678	-1.02520	rc6
NM_022482	GZF1	0.040723	-0.17769	-0.36298	-0.48974	-0.81269	-1.02502	rc6
NM_138391	TMEM183A	0.102759	-0.49121	-0.45144	-0.11564	-0.82897	-1.02475	rc6
NR_024252	FAM86HP	0.00437	-0.23108	-0.40419	-0.20096	-0.97158	-1.02465	rc6
NM_004085	TIMM8A	-0.21905	0.057775	0.178424	0.345075	-0.77179	-1.02397	rc6
NM_018984	SSH1	-0.20017	-0.58759	-0.24126	0.019467	-0.92095	-1.02327	rc6
NM_173584	EFCAB4A	0.099205	0.201949	0.012963	-0.11701	-0.9228	-1.02269	rc6
NM_030928	CDT1	-0.10855	-0.41825	-0.4164	-0.4751	-0.59513	-1.02158	rc6
NM_001161705	BFSP1	-0.11312	-0.66969	-0.39335	-0.87326	-0.98559	-1.02146	rc6
NM_000143	FH	0.125739	-0.03931	0.194558	0.407133	-0.68157	-1.02088	rc6
NM_001142597	CLP1	-0.03985	0.180782	-0.34797	0.030578	0.472889	-1.02042	rc6
NM_152707	SLC25A16	0.04641	0.226264	0.053474	0.083278	-0.72962	-1.01957	rc6
NM_080664	C14orf126	0.049529	0.098938	-0.06761	0.163865	-0.75338	-1.01945	rc6
NM_018361	AGPAT5	0.055198	0.130263	0.053989	0.821773	-0.71479	-1.01942	rc6
NM_000808	GABRA3	-0.16683	-0.32658	-0.584	-0.51646	-0.80782	-1.01905	rc6
NM_203283	RBPJ	0.193261	0.209585	-0.9658	0.377727	-0.31726	-1.01888	rc6
NM_014659	PPIP5K1	-0.2669	-0.16816	-0.21185	-0.34106	-0.2285	-1.01851	rc6
NM_002504	NFX1	0.052069	0.120095	-0.00226	-0.06684	-0.52904	-1.01743	rc6
NM_001282	AP2B1	-0.11541	-0.33347	0.243705	-0.28843	-0.1804	-1.01687	rc6
NR_037702	RNF7	-0.67479	-0.46484	-0.34985	0.34206	-0.28263	-1.01540	rc6
NM_001197323	HIRIP3	0.352999	-0.1006	0.316405	0.252771	-0.48379	-1.01475	rc6
NM_001097616	GPR89C	0.005786	0.168254	0.075872	0.411186	-0.47572	-1.01338	rc6
NM_203288	RP9	-0.121	-0.38046	-0.09621	0.114566	-0.90235	-1.01064	rc6
NM_024960	PANK2	-0.19555	-0.39142	0.031289	0.206073	-0.57962	-1.01055	rc6
NM_023940	RASL11B	-0.01262	0.339273	1.744173	0.889755	-0.75676	-1.00842	rc6
NM_005646	TARBP1	-0.12528	0.153528	-0.41365	-0.23096	-0.7256	-1.00784	rc6
NM_003193	TBCE	0.194407	-0.00934	-0.1114	0.185869	-0.91933	-1.00754	rc6
NM_001018109	PIR	0.302261	0.192459	-0.11481	-0.16117	-0.38374	-1.00715	rc6
NM_016551	TM7SF3	-0.03233	-0.21022	-0.0959	-0.004	-0.51789	-1.00713	rc6
NR_028500	LDHA	-0.58325	-0.01021	0.043556	0.759773	-0.72945	-1.00697	rc6
NM_018323	PI4K2B	-0.67524	-0.14839	-0.2036	-0.11167	-0.76392	-1.00686	rc6
NM_014976	PDCD11	-0.0426	-0.03833	0.232319	0.19607	-0.88456	-1.00616	rc6

NM_006973	ZNF32	0.090711	-0.10458	-0.29328	-0.05321	-0.66245	-1.00602	rc6
NM_032319	PRADC1	0.102458	-0.29289	-0.27457	-0.21319	-0.99384	-1.00479	rc6
NM_001079538_1	XAGE2B	0.35183	0.0264	0.188139	0.21486	-0.34699	-1.00394	rc6
NM_198066	GNPNAT1	-0.0576	0.051867	0.092931	0.502604	-0.60012	-1.00335	rc6
NM_002510	GPNMB	0.006867	-0.08213	-0.00381	0.106797	-0.64884	-1.00262	rc6
NM_004322	BAD	0.299984	-0.54971	-0.48611	0.427445	-0.72428	-1.00257	rc6
NM_181581	DUS4L	0.086634	-0.03157	-0.46303	0.075787	-0.83616	-1.00234	rc6
NM_001104629	C4orf19	0.220306	-0.27042	-0.30183	0.398938	-0.07078	-1.00143	rc6
NM_080821	FAM210B	-0.05135	-0.21179	-0.10433	0.253666	-0.55739	-1.00026	rc6

*rc: Repressed genes cluster
