# Supplementary figures and images for: Transcriptomic and ChIP-sequence interrogation of EGFR signaling in HER2+ breast cancer cells reveals a dynamic chromatin landscape and S100 genes as targets
Source: BMC Med Genomics. 2019 Feb 8;12:32. doi: 10.1186/s12920-019-0477-8 (PMC6368760; doi:10.1186/s12920-019-0477-8)

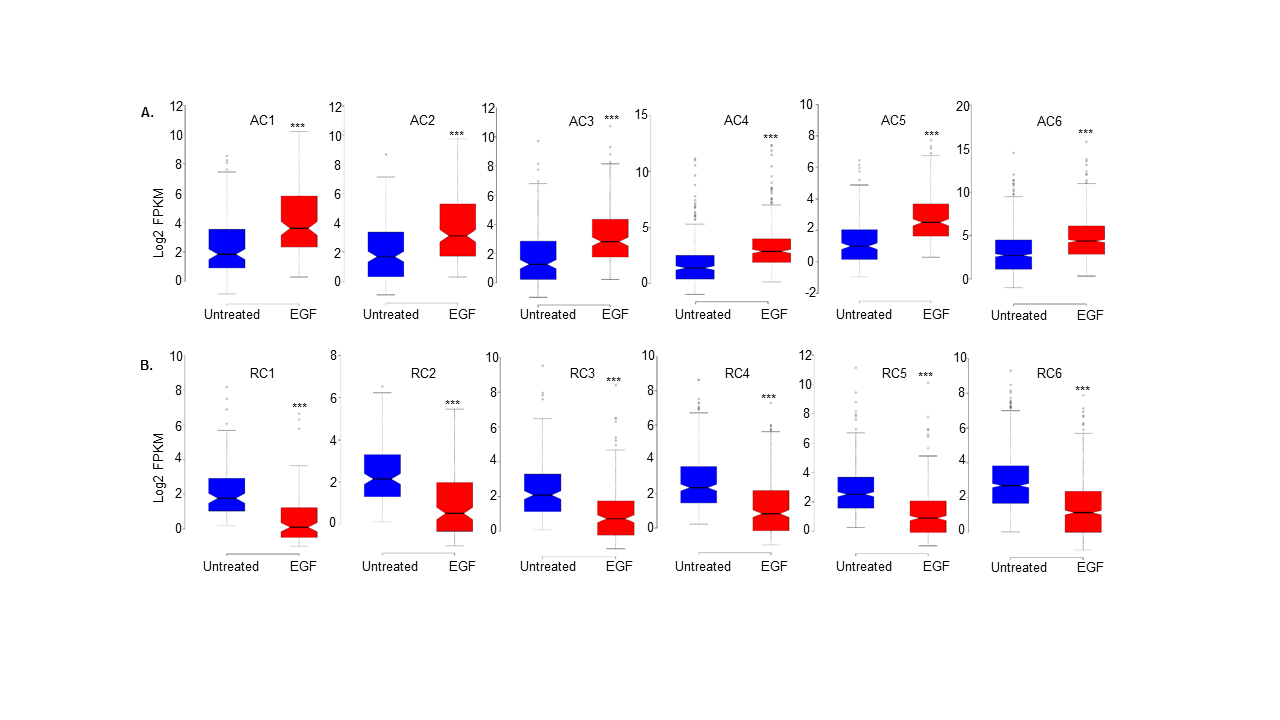

Supplement: Supplementary file 4 — Figure S1.Genes differentially expressed by cluster. A. Distributions of FPKMs plotted as boxplots for transcripts induced 2-fold (AC = activated cluster) B. Distributions of FPKMs plotted as boxplots for transcripts repressed 2-fold or more (RC = repressed cluster). ***p-value < .001 two-sided t-test for significant difference between Untreated and EGF treated samples. (TIF 158 kb) [file 12920_2019_477_MOESM4_ESM.tif]

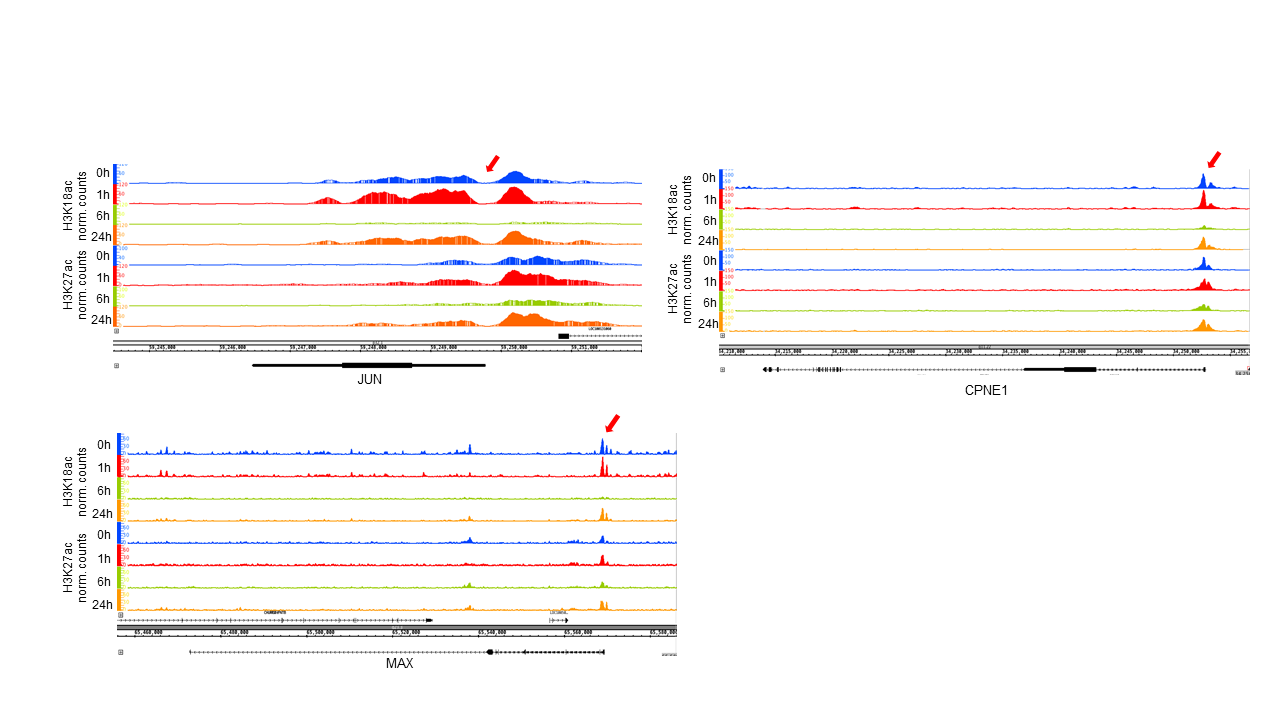

Supplement: Supplementary file 5 — Figure S2. EGFR signaling modulates chromatin at activated and repressed genes. IGB browser views of ChIP-seq H3K18ac and H3K27ac data at loci containing JUN, CPNE1 and MAX. Red arrows indicate annotated TSS. (TIF 213 kb) [file 12920_2019_477_MOESM5_ESM.tif]

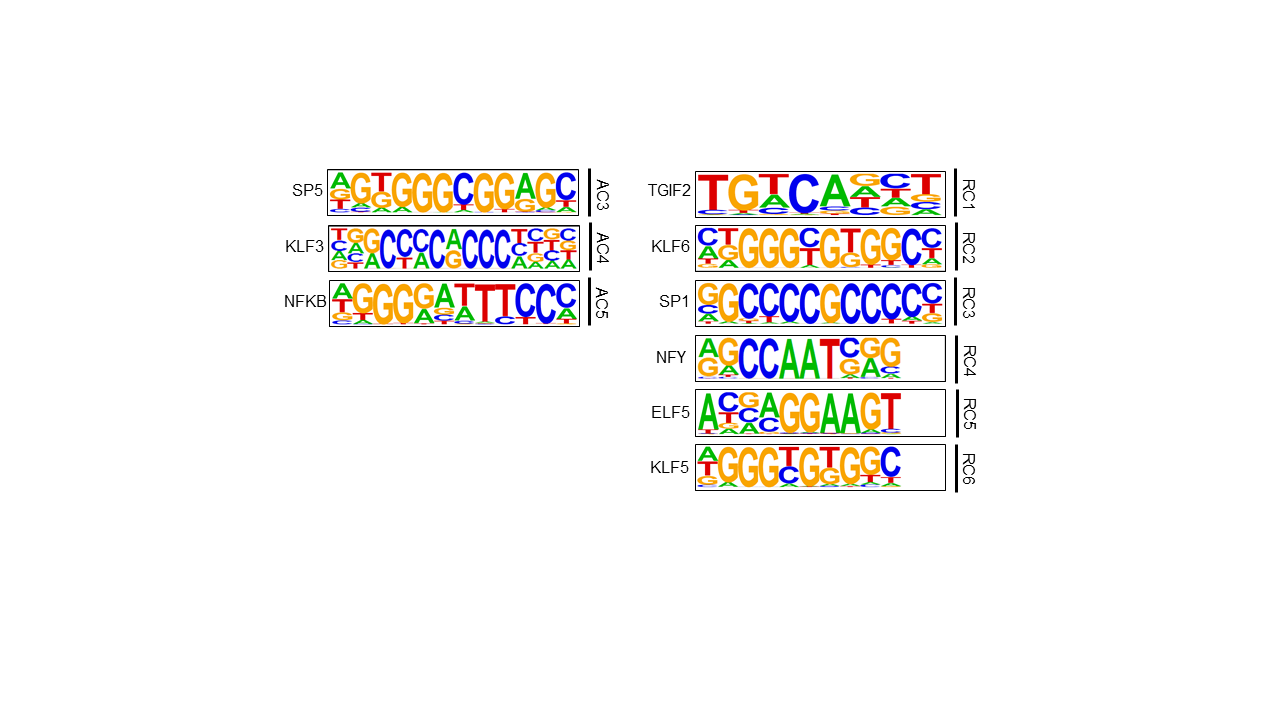

Supplement: Supplementary file 6 — Figure S3. Clusters contain motifs for TF known to be downstream of EGFR signaling. Motifs results as indicated by HOMER search from -300 bp to + 50 bp of each cluster. All motifs shown were enriched at p-value <.01. (TIF 230 kb) [file 12920_2019_477_MOESM6_ESM.tif]
